# Supplementary material for: Nickel-catalyzed allylic carbonylative coupling of alkyl zinc reagents with tert-butyl isocyanide
Source: Nat Commun. 2020 Jan 20;11:392. doi: 10.1038/s41467-020-14320-1 (PMC6971256; doi:10.1038/s41467-020-14320-1)
Supplement: Supplementary file 2 — Supplementary Information [file 41467_2020_14320_MOESM2_ESM.pdf]

Supporting Information for：

Nickel-Catalyzed Allylic Carbonylative Coupling of alkyl zinc reagents with *tert*-Butyl Isocyanide

Chen *et al*

correspondence to: yifengchen@ecust.edu.cn

This PDF Files includes:

Supplementary Methods

Supplementary Notes

Supplementary Figure 1-116

Supplementary References

Supplementary Methods

General information

All reactions were carried out under nitrogen atmosphere and anhydrous conditions unless otherwise indicated. THF were distilled from sodium:benzophenone. DCM was distilled over CaH_2. $NiCl_2 \cdot DME$ (CAS 29046-78-4) was purchased from Sigma Aldrich; $ZnCl_2$ (CAS 7646-85-7) was purchased from Strem; *N*,*N*-Dimethylacetamide (DMA, CAS 127-19-5) was purchased from Adamas (99.8%, SafeDry, with molecular sieves, Water ≤ 50 ppm (by K.F.), SafeSeal); *N,N*-Dimethylformamide (DMF, CAS 68-12-2) was purchased from Adamas (99.8%, SafeDry, with molecular sieves, Water ≤ 50 ppm (by K.F.), SafeSeal); ^{n}BuLi (CAS 109-72-8, 2.4 M in hexane) was purchased from Acros and Aldrich; Methylmagnesium bromide (CAS 75-16-1, 3.0 M in 2-Me-THF) and Ethylmagnesium bromide (CAS 925-90-6, 3.0 M in Et_2O) were purchased from Energy Chemical; Cyclopropylmagnesium bromide (CAS 23719-80-4, 0.5 M in THF) was purchased from Adamas; Vinylmagnesium bromide (CAS 1826-67-1, 1.0 M in THF) was purchased from Adamas; Zinc dust (325 mesh) and LiCl were purchased from Greagent. All Negishi reagents were titrated according to Knochel's procedure.[1] Reactions were monitored by thin-layer chromatography (TLC) carried out on 0.20 mm Huanghai silica gel plates (HSGF 254) using UV light as the visualizing agent and an acidic solution of Phosphomolybdic Acid (PMA) and basic solution of Potassium permanganate ($KMnO_4$) with heat as the developing agent. All new compounds were characterized by means of ^{1}H-NMR, ^{13}C-NMR, and HR-MS. GC analysis was performed on Agilent Technologies 7820A GC system. GC runs were performed with the following method: GC; HP-5 column; inlet temperature 100 °C; column temperature 100 °C for 1 min, then 50 °C:min to 280 °C, then 280 °C for 6 min. NMR spectra were recorded using a Bruker AVANCE III 400 MHz NMR spectrometer and can be found at the end of the paper. High-resolution mass spectra (HRMS) were recorded on a Waters GCT Premier mass spectrometer using EI-TOF (electron ionization-time of flight). Melting points were obtained for all crystalline solids on a X-4 Melting-Point Apparatus with microscope (from Shanghai Jingsong company). All ^{1}H-NMR data are reported in δ units, parts per million (ppm), and were calibrated relative to the signals for residual chloroform (7.26 ppm) in deuterochloroform ($CDCl_3$). All ^{13}C-NMR data are reported in ppm relative to $CDCl_3$ (77.16 ppm) and were obtained with ^{1}H decoupling. The following abbreviations or combinations thereof were used to explain the multiplicities: s = singlet, d = doublet, t = triplet, q = quartet, quin = quintet, sext = sextet, sep = septet.

Compounds **1u**,[2] **1v**,[3] **1y**,[4] **1z**,[3] **1aa**,[3] **1ab**,[5] **1ac**,[6] **1ad**,[5] **1ae**,[7] **1af**,[8] **1ah**,[9] were synthesized according to the published procedures.

General procedure for the synthesis of the allylic acetates.

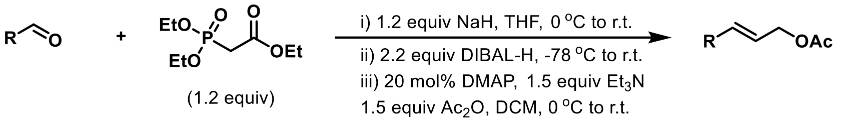

To a suspension of NaH (60% in mineral oil, 1.2 equiv) in THF (0.2 M) at 0 °C was added dropwise triethyl phosphonoacetate (1.2 equiv) and the reaction mixture was stirred for 0.5 h at 0 °C cold bath. A solution of the aldehyde (1.0 equiv) in THF (1.0 M) was then added to the reaction mixture, which was allowed to warm to room temperature and stirred until complete consumption of the aldehyde. The reaction was then quenched with sat. aq. NH_4Cl, the aqueous layer was extracted three times with EtOAc and separated organic layer was washed with brine, dried over Na_2SO_4 and concentrated under reduced pressure. The crude α,β-unsaturated ester was used in the next step without further purification.

To a solution of the above obtained α,β-unsaturated ester (1.0 equiv) in DCM (0.2 M) at -78 °C was added DIBAL-H (2.2 equiv) and the reaction mixture was stirred until complete consumption of the α,β-unsaturated ester. The reaction was then allowed to warm to room temperature and aq. NaOH (1.0 M) was added and the reaction mixture was stirred at room temperature for 1 h. The aqueous layer was extracted three times with DCM and separated organic layer was washed with brine, dried over Na_2SO_4 and concentrated under reduced pressure. The crude allylic alcohol was used in the next step without further purification.

To a 0 °C solution of the allylic alcohol (1.0 equiv) in DCM (0.5 M) was added Et_3N (1.5 equiv) and DMAP (20 mol%) and acetic anhydride (1.5 equiv), the reaction mixture was allowed to warm to room temperature and stirred overnight. The reaction mixture was quenched with sat. aq. NH_4Cl, the aqueous layer was extracted three times with DCM and separated organic layer was washed with brine, dried over Na_2SO_4 and concentrated under reduced pressure. The crude product was purified by column chromatography with PE:EtOAc as eluent to afford the desired allylic acetate.

(*E*)-3-(4-iodophenyl)allyl acetate (1w)

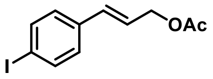

General procedure was followed on 3 mmol scale and purification by flash column chromatography on silica gel (PE:EtOAc = 100:1–20:1 v/v) afforded **1w** as a white solid (734 mg, 81%, 3 steps), mp: 72–73 °C, TLC (PE:EtOAc, 20:1 v/v): R_f= 0.40; ^{1}H NMR (400 MHz, $CDCl_3$): δ 7.63 (d, J = 8.0 Hz, 2H), 7.11 (d, J = 8.0 Hz, 2H), 6.55 (d, J = 15.6 Hz, 1H), 6.27 (dt, J = 16.0, 6.4 Hz, 1H), 4.70 (d, J = 6.4 Hz, 2H), 2.10 (s, 3H); ^{13}C NMR (100 MHz, $CDCl_3$): δ 170.8, 137.7, 135.7, 132.9, 128.4, 124.2, 93.6, 64.9, 21.1; HRMS (EI): Calcd for $C_{11}H_{11}IO_2$: 301.9804; found: 301.9806.

(*S*,*E*)-5,9-dimethyldeca-2,8-dien-1-yl acetate (1ai)

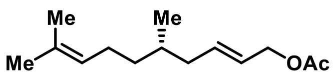

General procedure was followed on 10 mmol scale and purification by flash column chromatography on silica gel

(PE:EtOAc = 30:1–20:1 v/v) afforded **1ai** as a colorless oil (1.68 g, 75%, 3 steps), TLC (PE:EtOAc, 20:1 v/v): R_f = 0.60; ^{1}H NMR (400 MHz, $CDCl_3$): δ 5.70 (dt, *J* = 15.2, 7.2 Hz, 1H), 5.52 (dtd, *J* = 15.6, 6.4, 1.2 Hz, 1H), 5.07–5.03 (m, 1H), 4.47 (d, *J* = 6.4 Hz, 2H), 2.01 (d, *J* = 2.4 Hz, 3H), 1.97–1.89 (m, 2H), 1.87–1.82 (m, 1H), 1.64 (s, 3H), 1.56 (s, 3H), 1.47 (sext, *J* = 6.8 Hz, 1H), 1.34–1.19 (m, 2H), 1.15–1.06 (m, 1H), 0.83 (dd, *J* = 6.8, 1.2 Hz, 3H); ^{13}C NMR (100 MHz, $CDCl_3$): δ 170.3, 134.7, 130.8, 125.1, 124.6, 65.0, 39.6, 36.5, 32.3, 25.5, 25.4, 20.7, 19.2, 17.4; HRMS (EI): Calcd for $C_{14}H_{24}O_2$: 224.1776; found: 224.1777.

(*E*)-3-(4-(4,4,5,5-tetramethyl-1,3,2-dioxaborolan-2-yl)phenyl)allyl acetate (1x)

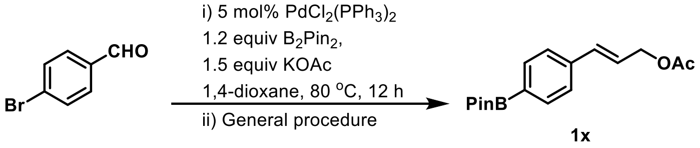

The aldehyde synthesized according to the published procedures[10] with some modifications. An oven-dried Schlenk tube containing a stirring bar was charged with $PdCl_2(PPh_3)_2$ (350 mg, 0.5 mmol, 5 mol%), KOAc (1.47 g, 15 mmol, 1.5 equiv), 4-bromobenzaldehyde (1.85 g, 10 mmol, 1.0 equiv), B_2Pin_2 (3.05 g, 12 mmol, 1.2 equiv). Then the Schlenk tube was evacuated and backfilled with N_2 (This process was repeated for three times). 1,4-dioxane (30 mL, 0.3 M) was then added and the tube was equipped with a balloon filled with N_2 at 80 °C for 12 h. The reaction mixture was quenched with sat. aq. NH_4Cl, the aqueous layer was extracted three times with EtOAc and separated organic layer was washed with brine, dried over Na_2SO_4 and concentrated under reduced pressure. The crude aldehyde was used in the next step without further purification.

General procedure was followed on 10 mmol scale and purification by flash column chromatography on silica gel (PE:EtOAc = 100:1–20:1 v/v) afforded **1x** as a white solid (2.40 g, 79%, 4 steps), mp: 62–63 °C, TLC (PE:EtOAc, 20:1 v/v): R_f = 0.23; ^{1}H NMR (400 MHz, $CDCl_3$): δ 7.76 (d, *J* = 8.0 Hz, 2H), 7.39 (d, *J* = 8.4 Hz, 2H), 6.66 (d, *J* = 16.0 Hz, 1H), 6.35 (dt, *J* = 16.0, 6.4 Hz, 1H), 4.73 (dd, *J* = 6.4, 1.2 Hz, 2H), 2.10 (s, 3H), 1.34 (s, 12H); ^{13}C NMR (100 MHz, $CDCl_3$): δ 171.0, 139.0, 135.2, 134.1, 126.0, 124.3, 84.0, 65.1, 25.0, 21.1; HRMS (EI): Calcd for $C_{17}H_{23}BO_4$: 302.1689; found: 302.1693.

(*R*,*E*)-4-(6-methoxynaphthalen-2-yl)pent-2-en-1-yl acetate (1aj)

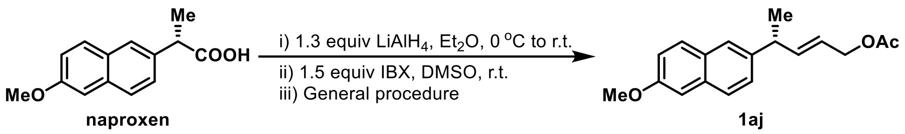

The alcohol was synthesized according to the published procedures[11] on 10 mmol scale and then used in the next step without further purification. To a solution of alcohol in DMSO (30 mL, 0.3 M) was added IBX (4.20 g, 15 mmol, 1.5 equiv) and the reaction mixture was stirred at room temperature until complete consumption of the alcohol substrate. The reaction mixture was quenched with H_2O and was filtered through Celite,

the aqueous layer was extracted three times with EtOAc and separated organic layer was washed with brine, dried over Na_2SO_4 and concentrated under reduced pressure. The crude aldehyde was used in the next step without further purification.

General procedure was followed on 10 mmol scale and purification by flash column chromatography on silica gel (PE:EtOAc = 100:1–20:1 v/v) afforded **1aj** as a colorless oil (852 mg, 30%, 5 steps), TLC (PE:EtOAc, 20:1 v/v): R_f = 0.58; ^{1}H NMR (400 MHz, $CDCl_3$): δ 7.70 (d, *J* = 8.4 Hz, 2H), 7.56 (d, *J* = 1.2 Hz, 1H), 7.31 (dd, *J* = 8.4, 2.0 Hz, 1H), 7.15–7.11 (m, 2H), 6.01 (ddt, *J* = 15.2, 6.4, 1.2 Hz, 1H), 5.63 (dtd, *J* = 15.6, 6.4, 1.2 Hz, 1H), 4.57 (d, *J* = 6.4 Hz, 2H), 3.92 (s, 3H), 3.63 (quin, *J* = 6.8 Hz, 1H), 2.07 (s, 3H), 1.46 (d, *J* = 7.2 Hz, 3H); ^{13}C NMR (100 MHz, $CDCl_3$): δ 171.0, 157.5, 140.5, 140.3, 133.4, 129.2, 129.2, 127.1, 126.7, 125.2, 123.0, 118.9, 105.7, 65.2, 55.4, 42.0, 21.2, 21,0; HRMS (EI): Calcd for $C_{18}H_{20}O_3$: 284.1412; found: 284.1411.

(*E*)-4-(4-isobutylphenyl)pent-2-en-1-yl acetate (1ak)

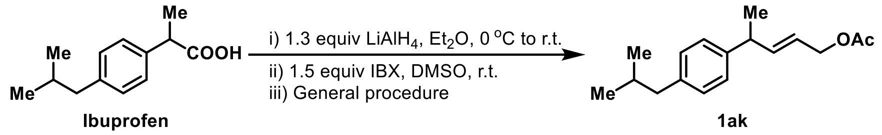

The alcohol was synthesized according to the published procedures[12] on 10 mmol scale and then used in the next step without further purification. To a solution of alcohol in DMSO (30 mL, 0.3 M) was added IBX (4.20 g, 15 mmol, 1.5 equiv) and the reaction mixture was stirred at room temperature until complete consumption of the alcohol substrate. The reaction mixture was quenched with H_2O and was filtered through Celite, the aqueous layer was extracted three times with EtOAc and separated organic layer was washed with brine, dried over Na_2SO_4 and concentrated under reduced pressure. The crude aldehyde was used in the next step without further purification.

General procedure was followed on 10 mmol scale and purification by flash column chromatography on silica gel (PE:EtOAc = 100:1–20:1 v/v) afforded **1ak** as a colorless oil (1.04 g, 37%, 5 steps), TLC (PE:EtOAc, 20:1 v/v): R_f = 0.54; ^{1}H NMR (400 MHz, $CDCl_3$): δ 7.14–7.09 (m, 4H), 5.95 (ddt, *J* = 15.2, 6.4, 1.2 Hz, 1H), 5.60 (dtd, *J* = 15.2, 6.4, 1.2 Hz, 1H), 4.56 (d, *J* = 6.4 Hz, 2H), 3.47 (quin, *J* = 6.8 Hz, 1H), 2.46 (d, *J* = 7.2 Hz, 2H), 2.07 (s, 3H), 1.87 (sep, *J* = 6.8 Hz, 1H), 1.38 (d, *J* = 6.8 Hz, 3H), 0.92 (d, *J* = 6.4 Hz, 6H); ^{13}C NMR (100 MHz, $CDCl_3$): δ 170.9, 142.4, 140.7, 139.7, 129.3, 127.0, 122.6, 65.2, 45.1, 41.7, 30.3, 22.5, 21.1, 21.1; HRMS (ESI): $[M+Na]^+$ calcd for $C_{17}H_{24}NaO_2$: 283.1674; found: 283.1680.

(2*E*,11*Z*)-icosa-2,11-dien-1-yl acetate (1al)

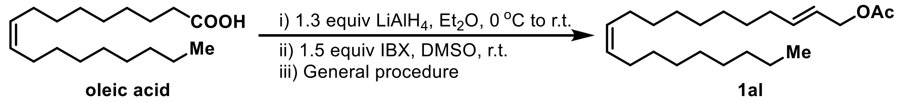

The alcohol was synthesized according to the published procedures[13] on 10 mmol scale and then used in the next step without further purification. To a solution of alcohol in DMSO (30 mL, 0.3 M) was added IBX (4.20 g, 15 mmol, 1.5 equiv) and the reaction mixture was stirred at room temperature until complete consumption of the alcohol

substrate. The reaction mixture was quenched with H_2O and was filtered through Celite, the aqueous layer was extracted three times with EtOAc and separated organic layer was washed with brine, dried over Na_2SO_4 and concentrated under reduced pressure. The crude aldehyde was used in the next step without further purification.

General procedure was followed on 10 mmol scale and purification by flash column chromatography on silica gel (PE:EtOAc = 100:1–20:1 v/v) afforded **1al** as a colorless oil (1.50 g, 45%, 5 steps), TLC (PE:EtOAc, 20:1 v/v): R_f = 0.63; ^{1}H NMR (400 MHz, $CDCl_3$): δ 5.76 (dt, *J* = 15.2, 6.8 Hz, 1H), 5.55 (dtt, *J* = 15.2, 6.8, 1.2 Hz, 1H), 5.38–5.29 (m, 2H), 4.50 (dd, *J* = 6.4, 0.8 Hz, 2H), 2.05 (s, 3H), 2.06–1.98 (m, 6H), 1.38–1.25 (m, 22H), 0.87 (t, *J* = 7.2 Hz, 3H); ^{13}C NMR (100 MHz, $CDCl_3$): δ 171.0, 136.9, 130.1, 128.0, 123.8, 65.5, 32.4, 32.0, 31.7, 29.9, 29.9, 29.7, 29.5, 29.5, 29.3, 29.3, 29.0, 27.3, 25.8, 22.8, 21.2, 14.2; HRMS (EI): Calcd for $C_{22}H_{40}O_2$: 336.3028; found: 336.3032.

(*R*,*E*)-6-((3*R*,5*R*,6*S*,8*S*,9*S*,10*R*,13*R*,14*S*,17*R*)-3,6-dimethoxy-10,13-dimethylhexadecahydro-1H-cyclopenta[a]phenanthren-17-yl)hept-2-en-1-yl acetate (1am)

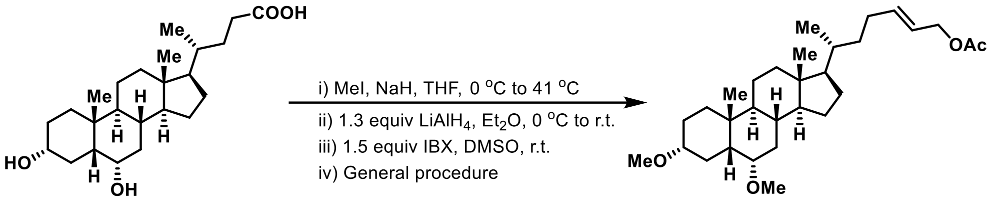

hyodeoxycholic acid 1am

To a solution of hyodeoxycholic acid (3.92 g, 10 mmol, 1.0 equiv) in THF (100 mL, 0.1 M) at 0 °C was added NaH (2.4 g, 60 mmol, 6.0 equiv, 60 % dispersion in mineral oil). After allowing the reaction mixture to stir for 1 h at room temperature, it was cooled to 0 °C. MeI (6.40 mL, 100 mmol, 10 equiv) was slowly added to the mixture and it was allowed to stir at 41 °C for 26 hours by TLC monitor. A second portion of NaH (2.40 g, 60 mmol, 6.0 equiv, 60 % dispersion in mineral oil) and MeI (3.0 mL, 47 mmol) were added to the reaction mixture and the reaction was allowed to stir at 41 °C for by TLC monitor. A third portion of NaH (2.4 g, 60 mmol, 6.0 equiv, 60 % dispersion in mineral oil) and MeI (3.0 mL, 47 mmol) were added to the reaction mixture and the reaction was allowed to stir at 41 °C for another 24 h. The reaction mixture was quenched with sat. aq. NH_4Cl at 0 °C. the aqueous layer was extracted three times with EtOAc and separated organic layer was washed with brine, dried over Na_2SO_4 and concentrated under reduced pressure. The crude product was purified by flash column chromatography on silica gel (PE:EtOAc = 20:1–5:1 v/v) afforded acid as a yellow oil (3.70 g, 88%), TLC (PE:EtOAc, 20:1 v/v): R_f = 0.65.

To a solution of acid (3.70 g, 8.8 mmol, 1.0 equiv) in Et_2O (30 mL, 0.3 M) at 0 °C was added $LiAlH_4$ (434 mg, 11.4 mmol, 1.3 equiv) slowly and the reaction mixture was allowed to warm to room temperature and stirred until complete consumption of the acid (~ 2 h). The reaction mixture was quenched with aq. 1 M NaOH and was filtered through Celite. The aqueous layer was extracted three times with EtOAc and separated organic layer was washed with brine, dried over Na_2SO_4 and concentrated under reduced pressure. The crude product was purified by flash column chromatography on

silica gel (PE:EtOAc = 20:1–5:1) afforded alcohol as a colorless oil (2.87 g, 80%), TLC (PE:EtOAc, 5:1 v/v): R_f = 0.23.

To a solution of alcohol (2.02 g, 5.0 mmol, 1.0 equiv) in DMSO (20 mL, 0.25 M) was added IBX (2.10 g, 7.5 mmol, 1.5 equiv) and the reaction mixture was allowed to warm to room temperature and stirred until complete consumption of the alcohol (~ 2 h). The reaction mixture was quenched with H_2O and was filtered through Celite, the aqueous layer was extracted three times with EtOAc and separated organic layer was washed with brine, dried over Na_2SO_4 and concentrated under reduced pressure. The crude aldehyde was used in the next step without further purification.

General procedure was followed on 5 mmol scale and purification by flash column chromatography on silica gel (PE:EtOAc = 50:1–30:1 v/v) afforded **1am** as a colorless oil (560.9 mg, 24%, 3 steps), TLC (PE:EtOAc, 5:1 v/v): R_f = 0.54; ^{1}H NMR (400 MHz, $CDCl_3$): δ 5.74 (dt, *J* = 15.6, 6.4 Hz, 1H), 5.54 (dt, *J* = 15.6, 6.4 Hz, 1H), 4.49 (d, *J* = 6.4 Hz, 2H), 3.50 (dt, *J* = 12.0, 4.8 Hz, 1H), 3.35 (s, 3H), 3.29 (s, 3H), 3.17–3.08 (m, 1H), 2.05 (s, 3H), 1.96–1.88 (m, 3H), 1.86–1.77 (m, 2H), 1.72–1.65 (m, 3H), 1.59–1.53 (m, 1H), 1.50–1.44 (m, 1H), 1.40–1.34 (m, 4H), 1.31–1.15 (m, 5H), 1.13–0.97 (m, 7H), 0.89 (d, *J* = 6.4 Hz, 3H), 0.89 (s, 3H), 0.62 (s, 3H); ^{13}C NMR (100 MHz, $CDCl_3$): δ 171.0, 137.3, 123.5, 80.3, 77.3, 65.5, 56.3, 56.2, 55.8, 55.7, 45.0, 43.0, 40.2, 40.1, 36.1, 35.8, 35.5, 35.2, 34.8, 31.8, 29.1, 28.3, 27.1, 25.6, 24.3, 23.8, 21.2, 20.9, 18.6, 12.1; HRMS (EI): Calcd for $C_{30}H_{50}O_4$: 474.3709; found: 474.3706.

1-(1-(4-chlorobenzoyl)-5-methoxy-2-methyl-1H-indol-3-yl)but-3-en-2-yl acetate (1an)

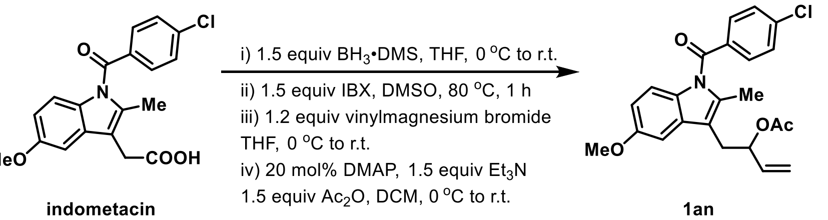

To a solution of indomethacin (1.8 g, 5.0 mmol, 1.0 equiv) in THF (20 mL, 0.25 M) at 0 °C was added dropwise a solution of BH_3·DMS (10.0 M in DMS, 0.75 mL, 7.5 mmol, 1.5 equiv). The yellow solution was warmed up to room temperature and then stirred at this temperature for 24 h. The reaction mixture was quenched by the slowly addition of MeOH (1.0 mL) at 0 °C. The solvent was removed under reduced pressure. The crude material was dissolved in DCM, washed with sat. aq. $NaHCO_3$, water, 3 M HCl aq., water and brine, dried with Na_2SO_4 and concentrated under reduced pressure. The crude product was purified by flash column chromatography on silica gel (PE:DCM = 1:1) afforded alcohol as a yellowish solid.

To a solution of the corresponding alcohol (1.0 g, 3.0 mmol, 1.0 equiv) in DMSO (20 mL, 0.15 M) was added IBX (1.26 g, 4.5 mmol, 1.5 equiv) at room temperature and the resulting mixture was heated at 80 °C in open flask for 1 h. The mixture was diluted with EtOAc and quenched with H_2O, then the mixture was filtered through Celite, the aqueous layer was extracted three times with EtOAc and separated organic layer was washed with brine, dried over Na_2SO_4 and concentrated under reduced pressure. The

crude product was purified by flash column chromatography on silica gel (PE:DCM = 1:1) afforded aldehyde as an orange solid.

To a solution of aldehyde (1.59 g, 2.53 mmol, 1.0 equiv) in THF (20 mL, 0.13 M) at 0 °C was added dropwise vinylmagnesium bromide (0.8 M, 3.8 mL, 3.04 mmol, 1.2 equiv), the reaction mixture was allowed to warm to room temperature and stirred until complete consumption of the aldehyde. The reaction mixture was quenched with sat. aq. NH_4Cl, the aqueous layer was extracted three times with EtOAc and separated organic layer was washed with brine, dried over Na_2SO_4 and concentrated under reduced pressure. The crude product was purified by flash column chromatography on silica gel (PE:DCM = 2:1) afforded allylic alcohol as an orange oil.

To a 0 °C solution of the allylic alcohol (369 mg, 1.0 mmol, 1.0 equiv) in DCM (2 mL, 0.5 M) was added Et_3N (0.21 mL, 1.5 mmol, 1.5 equiv) and DMAP (25.0 mg, 0.2 mmol, 20 mol%) was added dropwise acetic anhydride (0.14 mL, 1.5 mmol, 1.5 equiv), the reaction mixture was allowed to warm to room temperature and stirred overnight. The reaction mixture was quenched with sat. aq. NH_4Cl, the aqueous layer was extracted three times with DCM and separated organic layer was washed with brine, dried over Na_2SO_4 and concentrated under reduced pressure. The crude product was purified by flash column chromatography on silica gel (PE:EtOAc = 50:1–20:1–10:1 v/v) afforded **1an** as a yellow solid (239 mg, 58%), mp: 83–84 °C, TLC (PE:EtOAc, 5:1 v/v): R_f = 0.58; ^{1}H NMR (400 MHz, $CDCl_3$): δ 7.63 (dt, *J* = 8.8, 2.0 Hz, 2H), 7.46 (dt, *J* = 8.8, 2.0 Hz, 2H), 7.07 (d, *J* = 2.4 Hz, 1H), 6.88 (d, *J* = 8.8 Hz, 1H), 6.67 (dd, *J* = 8.8, 2.4 Hz, 1H), 5.87 (ddd, *J* = 17.2, 10.4, 6.4 Hz, 1H), 5.48 (q, *J* = 6.4 Hz, 1H), 5.25 (dt, *J* = 17.2, 1.2 Hz, 1H), 5.18 (dt, *J* = 10.4, 1.2 Hz, 1H), 3.85 (s, 3H), 3.06 (dd, *J* = 14.4, 6.4 Hz, 1H), 2.91 (dd, *J* = 14.4, 6.8 Hz, 1H), 2.34 (s, 3H), 2.04 (s, 3H); ^{13}C NMR (100 MHz, $CDCl_3$): δ 170.2, 168.4, 156.1, 139.2, 135.9, 135.7, 134.2, 131.3, 131.2, 130.9, 129.2, 117.1, 115.0, 111.6, 101.7, 74.4, 55.8, 29.7, 21.4, 13.7; HRMS (EI): Calcd for $C_{23}H_{22}ClNO_4$: 411.1237; found: 411.1233.

(*E*)-6-(((*R*)-2,5,7,8-tetramethyl-2-((4*R*,8*R*)-4,8,12-trimethyltridecyl)chroman-6-yl)oxy)hex-2-en-1-yl acetate (1ao)

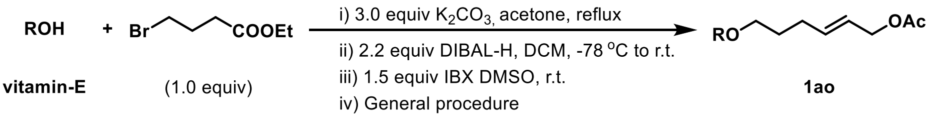

R =

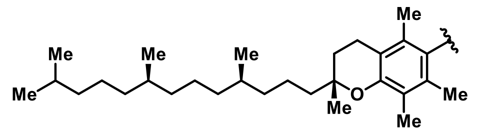

To a solution of K_2CO_3 (4.14 g, 30 mmol, 3.0 equiv) in acetone (20 mL, 0.5 M) was added Vitamin-E (4.5 mL, 10 mmol, 1.0 equiv) and ethyl 4-bromobutanoate (1.43 mL, 10 mmol, 1.0 equiv). The reaction was then allowed to stir at 80 °C by TLC monitor until complete consumption of the substrate. The reaction mixture was quenched with sat. aq. NH_4Cl, the aqueous layer was extracted three times with EtOAc and separated organic layer was washed with brine, dried over Na_2SO_4 and concentrated under reduced pressure. The crude ester was used in the next step without further purification.

To a solution of ester in DCM (30 mL, 0.3 M) at -78 °C was added DIBAL-H (14.7 mL, 22 mmol, 2.2 equiv) and the reaction mixture was stirred at this temperature overnight. The reaction mixture was quenched with aq. 1 M NaOH and was filtered through Celite. The aqueous layer was extracted three times with DCM and separated organic layer was washed with brine, dried over Na_2SO_4 and concentrated under reduced pressure. The crude product was purified by flash column chromatography on silica gel (PE:EtOAc = 20:1–5:1) afforded alcohol as a white solid (927 mg, 18%), TLC (PE:EtOAc, 5:1): R_f = 0.30.

To a solution of alcohol (927 mg, 1.8 mmol, 1.0 equiv) in DMSO (15 mL, 0.07 M) was added IBX (773 mg, 2.76 mmol, 1.5 equiv) and the reaction mixture was stir at 50 °C and until complete consumption of the alcohol. The reaction mixture was quenched with H_2O and was filtered through Celite, the aqueous layer was extracted three times with EtOAc and separated organic layer was washed with brine, dried over Na_2SO_4 and concentrated under reduced pressure. The crude aldehyde was used in the next step without further purification.

General procedure was followed on 1.84 mmol scale and purification by flash column chromatography on silica gel (PE:EtOAc = 100:1–50:1–20:1 v/v) afforded **1ao** as a colorless oil (589.9 mg, 56%, 3 steps), TLC (PE:EtOAc, 10:1 v/v): R_f = 0.47; ^{1}H NMR (400 MHz, $CDCl_3$): δ 5.85 (dt, *J* = 15.6, 6.4 Hz, 1H), 5.66 (dt, *J* = 15.6, 6.4 Hz, 1H), 4.54 (d, *J* = 6.4 Hz, 2H), 3.65 (t, *J* = 6.4 Hz, 2H), 2.58 (t, *J* = 6.8 Hz, 2H), 2.33 (dt, *J* = 7.2, 7.2 Hz, 2H), 2.17 (s, 3H), 2.13 (s, 3H), 2.09 (s, 3H), 2.08 (s, 3H), 1.90 (quin, *J* = 6.8 Hz, 2H), 1.85–1.73 (m, 2H), 1.61–1.51 (m, 3H), 1.45–1.38 (m, 2H), 1.39–1.24 (m, 8H), 1.24 (s, 3H), 1.18–1.07 (m, 8H), 0.88 (d, *J* = 6.8 Hz, 6H), 0.86 (d, *J* = 6.8 Hz, 6H); ^{13}C NMR (100 MHz, $CDCl_3$): δ 170.9, 148.3, 147.8, 135.8, 127.9, 125.9, 124.5, 122.9, 117.6, 74.9, 72.2, 65.3, 40.2 (d, *J* = 3.0 Hz), 39.5, 37.7, 37.6, 37.5, 37.4, 32.9, 32.8, 31.4 (d, *J* = 4.2 Hz), 29.6, 29.1, 28.1, 24.9, 24.6, 24.0, 22.9, 22.8, 21.1, 20.8, 19.9, 19.8, 19.8, 12.9, 12.0, 11.9. HRMS (EI): Calcd for $C_{37}H_{62}O_4$: 570.4648; found: 570.4649.

cyclopropyl-3-phenylallyl acetate (1ar)

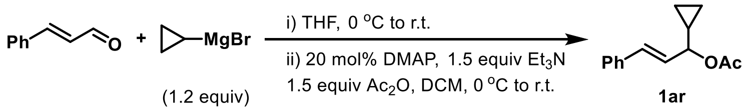

To a solution of cinnamaldehyde (1.25 mL, 10 mmol, 1.0 equiv) in THF (20 mL, 0.5 M) at 0 °C was added dropwise cyclopropylmagnesium bromide (0.5 M in THF, 24 mL, 12 mmol, 1.2 equiv), the reaction mixture was allowed to warm to room temperature and stirred until complete consumption of the cinnamaldehyde. The reaction mixture was quenched with sat. aq. NH_4Cl, the aqueous layer was extracted three times with EtOAc and separated organic layer was washed with brine, dried over Na_2SO_4 and concentrated under reduced pressure. The crude alcohol was used in the next step without further purification.

General procedure was followed on 5 mmol scale and purification by flash column chromatography on silica gel (PE:EtOAc = 100:1–20:1 v/v) afforded **1ar** as a colorless oil (756 mg, 70%), TLC (PE:EtOAc, 20:1 v/v): R_f = 0.65; ^{1}H NMR (400 MHz, $CDCl_3$):

δ 7.40 (d, *J* = 7.2 Hz, 2H), 7.33 (t, *J* = 7.6 Hz, 2H), 7.26 (t, *J* = 6.8 Hz, 1H), 6.63 (d, *J* = 16.0 Hz, 1H), 6.24 (dd, *J* = 16.0, 6.8 Hz, 1H), 4.90 (t, 7.2 Hz, 1H), 2.11 (s, 3H), 1.23–1.14 (m, 1H), 0.64–0.57 (m, 2H), 0.49 (dt, *J* = 9.2, 4.4 Hz, 1H), 0.39 (dt, *J* = 8.8, 4.4 Hz, 1H); ^{13}C NMR (100 MHz, $CDCl_3$): δ 170.5, 136.4, 132.2, 128.6, 128.0, 126.9, 126.7, 78.7, 21.5, 15.1, 3.8, 2.8; HRMS (EI): Calcd for $C_{14}H_{16}O_2$: 216.1150; found: 216.1151.

Preparation of $ZnCl_2$ Solution (1.0 M in THF)

Zinc chloride (1.0 M in THF): Finely powdered anhydrous $ZnCl_2$ (0.68 g, 5.0 mmol) was weighed into a 25-mL, flame-dried Schlenk tube in the glove box. The tube was taken out of the glove box and heated with a heat gun for 2 min under vacuum and then back filled with nitrogen (This process was repeated 3 times.). After cooling down to room temperature, the flask was backfilled with N_2, and THF (anhydrous, 5.0 mL) was added. The suspension was vigorously stirred for 30 min before it was used.

Preparation of ^{n}BuZnCl

To a solution of $ZnCl_2$ (1.0 M, 1.0 mL, 1.0 mmol) in THF (0.6 mL) was added ^{n}BuLi (2.4 M in hexane, 0.42 mL, 1.0 mmol) at 0 °C. The mixture was vigorously stirred at the same temperature for 15 min. The concentration of ^{n}BuZnCl is from 0.29 M to 0.45 M.

Preparation of MeZnBr

To a solution of $ZnBr_2$ (675 mg, 3.0 mmol, 1.0 equiv) in THF (5.0 mL) was added dropwise MeMgBr solution (3.0 M in THF, 1.0 mL, 3.0 mmol, 1.0 equiv) at 0 °C. The mixture was stirred at room temperature for 1 h, cooled to 0 °C, and the stirring was stopped to allow the Mg salts to settle for > 1 h. The concentration of MeZnBr is 0.33 M to 0.36 M.

General procedure for the preparation of organozinc reagents via direct insertion of zinc

This method is modified from the reported literature.[14] Anhydrous LiCl (10 mmol, 2.0 equiv) was placed in an N_2-flushed sealed tube and dried for 10 min under the vacuum with heat gun. Zinc powder (10 mmol, 2.0 equiv, 325 mesh) was added under N_2 and the heterogeneous mixture of Zn and LiCl was dried again for 10 min under the vacuum with heat gun. The sealed tube was backfilled with N_2 (This process was repeated for three times). THF (5 mL) was added and the Zn was activated with $BrCH_2CH_2Br$ (5 mol%) under the reflux condition for 20 min. After cooling, Me_3SiCl (1 mol%) and I_2 (0.5 mol%) was added under N_2, then the mixture was refluxed for another 20 min. Alkyl halide (5 mmol) was then added neat at room temperature and the reaction mixture was stirred at 65 ºC for 12–72 h (checked by GC analysis of reaction aliquots, the conversion was higher than 98%). The concentration of Negishi reagents range from 0.40 M to 0.68 M.

General procedure A for the Ni-Catalyzed carbonylative Negishi reaction

An oven-dried Schlenk tube charged with $NiCl_2$·DME (10 mol%) was evacuated and backfilled with N_2 (This process was repeated for three times). DMA (0.1 M) was added into the reaction mixture. To this solution was subsequently added allylic acetate (1.0 equiv), ^{t}BuNC (1.5 equiv) and Negishi reagent (1.5 equiv). The tube was equipped with a balloon filled with N_2 at 25 ºC until complete consumption of the starting material. The mixture was added 1 M HCl aq. and stirred at room temperature for 0.25 h. The

mixture was then extracted with EtOAc and separated organic layer was washed with brine, dried over anhydrous Na_2SO_4, and concentrated under reduced pressure to yield the crude product, which was purified by silica gel flash column chromatography.

General procedure B for the Ni-Catalyzed carbonylative Negishi reaction

An oven-dried Schlenk tube charged with Ni(cod)$_2$ (10 mol%) (weighed in the glove box), DMA (0.1 M) was then added into the reaction mixture. To this solution was subsequently added allylic acetate (1.0 equiv), ^{t}BuNC (1.5 equiv) and Negishi reagent (1.5 equiv). The tube was equipped with a balloon filled with N_2 at 50 °C until complete consumption of the starting material. The mixture was added 1 M HCl aq. and stirred at room temperature for 0.25 h. The mixture was then extracted with EtOAc and separated organic layer was washed with brine, dried over anhydrous Na_2SO_4, and concentrated under reduced pressure to yield the crude product, which was purified by silica gel flash column chromatography.

(*E*)-1-phenyldodec-1-en-4-one (4a)

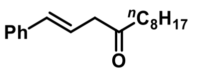

General procedure A was followed with cinnamyl acetate (34 μL, 0.2 mmol, 1.0 equiv), ^{t}BuNC (34 μL, 0.3 mmol, 1.5 equiv) and *n*-octylzinc(II) bromide (0.59 M, 0.51 mL, 0.3 mmol, 1.5 equiv) prepared according to the general procedure used 1-bromooctane with a reaction time of 0.5 h. The reaction mixture was purified by flash column chromatography (silica gel, PE:EtOAc = 200:1–150:1 v/v) to afford **4a** as a pale yellow oil (46.3 mg, 90%), TLC (PE:EtOAc, 20:1 v/v): R_f = 0.49; ^{1}H NMR (400 MHz, $CDCl_3$): δ 7.39–7.36 (m, 2H), 7.33–7.29 (m, 2H), 7.23 (tt, *J* = 7.2, 1.2 Hz, 1H), 6.47 (d, *J* = 16.0 Hz, 1H), 6.32 (dt, *J* = 16.0, 7.2 Hz, 1H), 3.32 (dd, *J* = 7.2, 1.2 Hz, 2H), 2.48 (t, *J* = 7.2 Hz, 2H), 1.59 (quin, *J* = 7.2 Hz, 2H), 1.28 (m, 10H), 0.88 (t, *J* = 7.2 Hz, 3H); ^{13}C NMR (100 MHz, $CDCl_3$): δ 209.1, 137.1, 133.7, 128.7, 127.6, 126.4, 122.3, 47.1, 42.6, 31.9, 29.5, 29.3, 29.3, 23.9, 22.8, 14.2; The spectral data are consistent with those reported in the literature.[15]

(*E*)-6-phenylhex-5-en-3-one (4b)

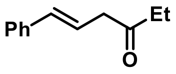

General procedure A was followed with cinnamyl acetate (34 μL, 0.2 mmol, 1.0 equiv), ^{t}BuNC (34 μL, 0.3 mmol, 1.5 equiv) and diethylzinc (1.0 M, 0.4 mL, 0.4 mmol, 2.0 equiv) with a reaction time of 0.5 h. The reaction mixture was purified by flash column chromatography (silica gel, PE:EtOAc = 200:1–150:1 v/v) to afford **4b** as a colorless oil (29.2 mg, 84%), TLC (PE:EtOAc, 20:1 v/v): R_f = 0.46; ^{1}H NMR (400 MHz, $CDCl_3$): δ 7.37 (d, J = 7.2 Hz, 2H), 7.31 (t, J = 7.2 Hz, 2H), 7.23 (t, J = 7.2 Hz, 1H), 6.47 (d, J = 16.0 Hz, 1H), 6.32 (dt, J = 16.0, 7.2 Hz, 1H), 3.33 (d, J = 7.2 Hz, 2H), 2.52 (q, J = 7.2 Hz, 2H), 1.08 (t, J = 7.2 Hz, 3H); ^{13}C NMR (100 MHz, $CDCl_3$): δ 209.5, 137.0, 133.7, 128.7, 127.6, 126.4, 122.3, 46.7, 35.8, 7.9; The spectral data are consistent with those reported in the literature.[16]

(*E*)-5-phenylpent-4-en-2-one (4c)

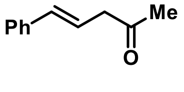

General procedure A was followed with cinnamyl acetate cinnamyl acetate (34 μL, 0.2 mmol, 1.0 equiv), ^{t}BuNC (34 μL, 0.3 mmol, 1.5

equiv) and methylzinc(II) bromide (0.36 M, 0.83 mL, 0.3 mmol, 1.5 equiv) prepared according to the procedure above with a reaction time of 0.5 h. The reaction mixture was purified by flash column chromatography (silica gel, PE:EtOAc = 200:1–100:1 v/v) to afford **4c** as a colorless oil (23.9 mg, 75%), TLC (PE:EtOAc, 20:1 v/v): R_f = 0.30; ^{1}H NMR (400 MHz, $CDCl_3$): δ 7.39–7.37 (m, 2H), 7.33–7.29 (m, 2H), 7.23 (tt, *J* = 7.2, 1.2 Hz, 1H), 6.48 (d, *J* = 16.0 Hz, 1H), 6.31 (dt, *J* = 16.0, 7.2 Hz, 1H), 3.34 (dd, *J* = 7.2, 0.8 Hz, 2H), 2.22 (s, 3H); ^{13}C NMR (100 MHz, $CDCl_3$): δ 207.0, 136.9, 133.9, 128.7, 127.7, 126.4, 122.0, 47.9, 29.8; The spectral data are consistent with those reported in the literature.[17]

(*E*)-1,5-diphenylpent-4-en-2-one (4d)

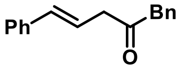

General procedure A was followed with cinnamyl acetate (34 μL, 0.2 mmol, 1.0 equiv), ^{t}BuNC (34 μL, 0.3 mmol, 1.5 equiv) and benzylzinc(II) bromide (0.60 M, 0.5 mL, 0.3 mmol, 1.5 equiv) prepared according to the general procedure used (bromomethyl)benzene with a reaction time of 0.5 h. The reaction mixture was purified by flash column chromatography (silica gel, PE:EtOAc = 200:1–150:1–100:1 v/v) to afford **4d** as a pale yellow oil (40.0 mg, 85%), TLC (PE:EtOAc, 20:1 v/v): R_f = 0.47; ^{1}H NMR (400 MHz, $CDCl_3$): δ 7.36–7.32 (m, 5H), 7.29 (d, *J* = 8.0 Hz, 2H), 7.25–7.21 (m, 3H), 6.42 (d, *J* = 16.0 Hz, 1H), 6.27 (dt, *J* = 16.0, 7.2 Hz, 1H), 3.77 (s, 2H), 3.36 (d, *J* = 6.8 Hz, 2H); ^{13}C NMR (100 MHz, $CDCl_3$): δ 206.3, 136.9, 134.0, 134.0, 129.6, 128.9, 128.7, 127.7, 127.2, 126.4, 121.9, 49.8, 46.1; The spectral data are consistent with those reported in the literature.[18]

(*E*)-1,6-diphenylhex-5-en-3-one (4e)

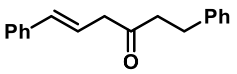

General procedure A was followed with cinnamyl acetate (34 μL, 0.2 mmol, 1.0 equiv), ^{t}BuNC (34 μL, 0.3 mmol, 1.5 equiv) and phenethylzinc(II) bromide (0.58 M, 0.52 mL, 0.3 mmol, 1.5 equiv) prepared according to the general procedure used (2-bromoethyl)benzene with a reaction time of 0.5 h. The reaction mixture was purified by flash column chromatography (silica gel, PE:EtOAc = 200:1–150:1–100:1 v/v) to afford **4e** as a pale yellow solid (37.4 mg, 75%), mp: 59–60 °C, TLC (PE:EtOAc, 20:1 v/v): R_f = 0.40; ^{1}H NMR (400 MHz, $CDCl_3$): δ 7.36–7.33 (m, 2H), 7.31–7.27 (m, 3H), 7.26–7.21 (m, 2H), 7.20–7.17 (m, 3H), 6.43 (d, *J* = 16.0 Hz, 1H), 6.27 (dt, *J* = 16.0, 7.2 Hz, 1H), 3.29 (dd, *J* = 7.2, 1.2 Hz, 2H), 2.93–2.89 (m, 2H), 2.84–2.79 (m, 2H); ^{13}C NMR (100 MHz, $CDCl_3$): δ 207.9, 141.0, 136.9, 133.9, 128.7, 128.6, 128.5, 127.7, 126.4, 126.3, 121.9, 47.3, 44.1, 29.8; The spectral data are consistent with those reported in the literature.[19]

(*E*)-1-phenylocta-1,7-dien-4-one (4f)

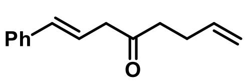

General procedure A was followed with cinnamyl acetate (34 μL, 0.2 mmol, 1.0 equiv), ^{t}BuNC (34 μL, 0.3 mmol, 1.5 equiv) and but-3-en-1-ylzinc(II) bromide (0.59 M, 0.51 mL, 0.3 mmol, 1.5 equiv) prepared according to the general procedure used 4-bromobut-1-ene with a reaction time of 0.5 h. The reaction mixture was purified by flash column chromatography

chromatography (silica gel, PE:EtOAc = 200:1–150:1–100:1 v/v) to afford **4f** as a pale yellow oil (34.0 mg, 85%), TLC (PE:EtOAc, 20:1 v/v): R_f = 0.52; ^{1}H NMR (400 MHz, $CDCl_3$): δ 7.39–7.36 (m, 2H), 7.33–7.29 (m, 2H), 7.23 (tt, *J* = 7.2, 1.2 Hz, 1H), 6.48 (d, *J* = 15.6 Hz, 1H), 6.31 (dt, *J* = 16.0, 7.2 Hz, 1H), 5.82 (ddt, *J* = 17.2, 10.4, 6.4 Hz, 1H), 5.05 (ddt, *J* = 17.2, 1.6, 1.6 Hz, 1H), 4.99 (ddt, *J* = 10.0, 1.6, 1.6 Hz, 1H), 3.33 (dd, *J* = 6.8, 1.2 Hz, 2H), 2.60 (t, *J* = 7.2 Hz, 2H), 2.35 (ddt, *J* = 14.4, 6.8, 1.2 Hz, 2H); ^{13}C NMR (100 MHz, $CDCl_3$): δ 208.0, 137.1, 137.0, 133.9, 128.7, 127.7, 126.4, 122.1, 115.5, 47.1, 41.6, 27.8; HRMS (EI): Calcd for $C_{14}H_{16}O$: 200.1201; found: 200.1202.

(*E*)-7-methyl-1-phenylocta-1,6-dien-4-one (4g)

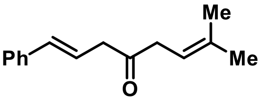

General procedure A was followed with cinnamyl acetate (34 μL, 0.2 mmol, 1.0 equiv), ^{t}BuNC (34 μL, 0.3 mmol, 1.5 equiv) and (3-methylbut-2-en-1-yl)zinc(II) bromide (0.44 M, 0.68 mL, 0.3 mmol, 1.5 equiv) prepared according to the general procedure used 1-bromo-3-methylbut-2-ene which stirred at room temperature for 26 h with a reaction time of 0.5 h. The reaction mixture was purified by flash column chromatography (silica gel, PE:EtOAc = 200:1–150:1 v/v) to afford **4g** as a pale yellow oil (30.3 mg, 71%), TLC (PE:EtOAc, 20:1 v/v): R_f = 0.47; ^{1}H NMR (400 MHz, $CDCl_3$): δ 7.38–7.36 (m, 2H), 7.33–7.29 (m, 2H), 7.23 (tt, J = 7.2, 1.2 Hz, 1H), 6.47 (d, J = 15.6 Hz, 1H), 6.31 (dt, J = 16.0, 7.2 Hz, 1H), 5.35–5.30 (m, 1H), 3.34 (dd, J = 7.2, 1.2 Hz, 2H), 3.19 (d, J = 6.8 Hz, 2H), 1.77 (s, 3H), 1.64 (s, 3H); ^{13}C NMR (100 MHz, $CDCl_3$): δ 207.4, 137.0, 136.2, 133.8, 128.7, 127.6, 126.4, 122.2, 115.8, 46.4, 42.4, 25.9, 18.2; HRMS (EI): Calcd for $C_{15}H_{18}O$: 214.1358; found: 214.1357.

(*E*)-9-fluoro-1-phenylnon-1-en-4-one (4h)

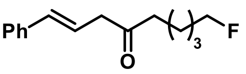

General procedure A was followed with cinnamyl acetate (34 μL, 0.2 mmol, 1.0 equiv), ^{t}BuNC (34 μL, 0.3 mmol, 1.5 equiv) and (5-fluoropentyl)zinc(II) bromide (0.64 M, 0.47 mL, 0.3 mmol, 1.5 equiv) prepared according to the general procedure used 1-bromo-5-fluoropentane with a reaction time of 0.5 h. The reaction mixture was purified by flash column chromatography (silica gel, PE:EtOAc = 150:1–100:1–100:1.5 v/v) to afford **4h** as a pale yellow solid (40.0 mg, 85%), mp: 40–41 °C, TLC (PE:EtOAc, 20:1 v/v): R_f = 0.43; ^{1}H NMR (400 MHz, $CDCl_3$): δ 7.38–7.36 (m, 2H), 7.33–7.29 (m, 2H), 7.23 (tt, *J* = 7.2, 1.2 Hz, 1H), 6.48 (d, *J* = 16.0 Hz, 1H), 6.31 (dt, *J* = 16.0, 7.2 Hz, 1H), 4.49 (t, *J* = 6.0 Hz, 1H), 4.37 (t, *J* = 6.0 Hz, 1H), 3.32 (dd, *J* = 7.2, 1.2 Hz, 2H), 2.52 (t, *J* = 7.2 Hz, 2H), 1.77–1.67 (m, 2H), 1.66–1.61 (m, 2H), 1.45–1.37 (m, 2H); ^{13}C NMR (100 MHz, $CDCl_3$): δ 208.6, 137.0, 133.8, 128.7, 127.7, 126.4, 122.2, 84.0 (d, J_{C-F} = 163.4 Hz), 47.1, 42.3, 30.3 (d, J_{C-F} = 19.4 Hz), 25.0 (d, J_{C-F} = 5.2 Hz), 23.3; ^{19}F NMR (376 MHz, $CDCl_3$): -218.4 (tt, *J* = 46.9, 25.6 Hz, 1F); HRMS (EI): Calcd for $C_{15}H_{19}FO$: 234.1420; found: 234.1418.

(*E*)-10-chloro-1-phenyldec-1-en-4-one (4i)

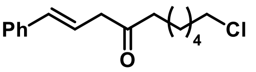

General procedure A was followed with cinnamyl acetate (34 μL, 0.2 mmol, 1.0 equiv), ^{t}BuNC (34 μL, 0.3 mmol, 1.5 equiv) and

(6-chlorohexyl)zinc(II) bromide (0.68 M, 0.44 mL, 0.3 mmol, 1.5 equiv) prepared according to the general procedure used 1-bromo-6-chlorohexane with a reaction time of 1 h. The reaction mixture was purified by flash column chromatography (silica gel, PE:EtOAc = 200:1–150:1–100:1 v/v) to afford **4i** as a colorless oil (33.0 mg, 62%), TLC (PE:EtOAc, 20:1 v/v): R_f = 0.46; ^{1}H NMR (400 MHz, $CDCl_3$): δ 7.38–7.36 (m, 2H), 7.33–7.29 (m, 2H), 7.23 (tt, *J* =7.2, 1.2 Hz, 1H), 6.47 (d, *J* = 16.0 Hz, 1H), 6.31 (dt, *J* = 16.0, 7.2 Hz, 1H), 3.52 (t, *J* = 6.8 Hz, 2H), 3.31 (dd, *J* = 7.2, 1.2 Hz, 2H), 2.50 (t, *J* = 7.2 Hz, 2H), 1.76 (quin, *J* = 6.8 Hz, 2H), 1.61 (quin, *J* = 7.6 Hz, 2H), 1.48–1.41 (m, 2H), 1.36–1.28 (m, 2H); ^{13}C NMR (100 MHz, $CDCl_3$): δ 208.8, 137.0, 133.8, 128.7, 127.7, 126.3, 122.2, 47.1, 45.1, 42.3, 32.5, 28.5, 26.7, 23.5; HRMS (EI): Calcd for $C_{16}H_{21}ClO$: 264.1281; found: 264.1280.

(*E*)-6-oxo-9-phenylnon-8-enenitrile (4j)

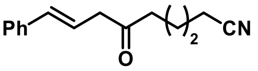

General procedure A was followed with cinnamyl acetate (34 μL, 0.2 mmol, 1.0 equiv), ^{t}BuNC (34 μL, 0.3 mmol, 1.5 equiv) and (4-cyanobutyl)zinc(II) bromide (0.40 M, 0.75 mL, 0.3 mmol, 1.5 equiv) prepared according to the general procedure used 5-bromopentanenitrile with a reaction time of 0.5 h. The reaction mixture was purified by flash column chromatography (silica gel, PE:EtOAc = 30:1–10:1–5:1 v/v) to afford **4j** as a pale yellow solid (33.2 mg, 73%), mp: 46–47 ºC, TLC (PE:EtOAc, 10:1 v/v): R_f = 0.35; ^{1}H NMR (400 MHz, $CDCl_3$): δ 7.38–7.36 (m, 2H), 7.33–7.29 (m, 2H), 7.24 (tt, *J* = 7.2, 1.2 Hz, 1H), 6.48 (d, *J* = 16.0 Hz, 1H), 6.29 (dt, *J* = 16.0, 7.2 Hz, 1H), 3.32 (dd, *J* = 7.2, 1.2 Hz, 2H), 2.56 (t, *J* = 6.8 Hz, 2H), 2.35 (t, *J* = 6.8 Hz, 2H), 1.79–1.72 (m, 2H), 1.69–1.63 (m, 2H); ^{13}C NMR (100 MHz, $CDCl_3$): δ 207.7, 136.8, 134.1, 128.7, 127.8, 126.4, 121.8, 119.6, 47.1, 41.2, 24.9, 22.7, 17.3; HRMS (EI): Calcd for $C_{15}H_{17}NO$: 227.1310; found: 227.1311.

ethyl (*E*)-5-oxo-8-phenyloct-7-enoate (4k)

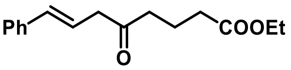

General procedure A was followed with cinnamyl acetate (34 μL, 0.2 mmol, 1.0 equiv), ^{t}BuNC (34 μL, 0.3 mmol, 1.5 equiv) and (4-ethoxy-4-oxobutyl)zinc(II) bromide (0.48 M, 0.63 mL, 0.3 mmol, 1.5 equiv) prepared according to the general procedure used ethyl 4-bromobutanoate with a reaction time of 0.5 h. The reaction mixture was purified by flash column chromatography (silica gel, PE:EtOAc = 50:1–30:1–20:1 v/v) to afford **4k** as a pale yellow oil (40.6 mg, 78%), TLC (PE:EtOAc, 5:1 v/v): R_f = 0.43; ^{1}H NMR (400 MHz, $CDCl_3$): δ 7.38–7.35 (m, 2H), 7.32–7.28 (m, 2H), 7.22 (tt, *J* = 7.2, 1.2 Hz, 1H), 6.47 (d, *J* = 16.0 Hz, 1H), 6.29 (dt, *J* = 15.6, 7.2 Hz, 1H), 4.11 (q, *J* = 7.2 Hz, 2H), 3.31 (dd, *J* = 7.2, 1.2 Hz, 2H), 2.57 (t, *J* = 7.2 Hz, 2H), 2.33 (t, *J* = 7.2 Hz, 2H), 1.91 (quin, *J* = 7.2 Hz, 2H), 1.23 (t, *J* = 7.2 Hz, 3H); ^{13}C NMR (100 MHz, $CDCl_3$): δ 208.0, 173.3, 136.9, 133.9, 128.7, 127.7, 126.4, 122.0, 60.5, 47.1, 41.3, 33.3, 18.9, 14.3; The spectral data are consistent with those reported in the literature.[20]

(*E*)-7-oxo-10-phenyldec-9-en-1-yl pivalate (4l)

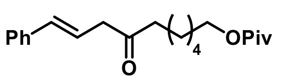

General procedure A was followed with cinnamyl acetate (34 μL, 0.2 mmol, 1.0 equiv), ^{t}BuNC (34 μL, 0.3 mmol, 1.5 equiv) and (6-(pivaloyloxy)hexyl)zinc(II) bromide (0.42 M, 0.71 mL, 0.3 mmol, 1.5 equiv) prepared according to the general procedure used 6-bromohexyl pivalate with a reaction time of 0.5 h. The reaction mixture was purified by flash column chromatography (silica gel, PE:EtOAc = 100:1–50:1–30:1 v/v) to afford **4l** as a pale yellow oil (57.2 mg, 87%), TLC (PE:EtOAc, 20:1 v/v): R_f = 0.41; ^{1}H NMR (400 MHz, $CDCl_3$): δ 7.37–7.35 (m, 2H), 7.32–7.28 (m, 2H), 7.22 (tt, J = 7.2, 1.2 Hz, 1H), 6.46 (d, J = 16.0 Hz, 1H), 6.30 (dt, J = 16.0, 7.2 Hz, 1H), 4.03 (t, J = 6.8 Hz, 2H), 3.31 (dd, J = 6.8, 1.2 Hz, 2H), 2.48 (t, J = 7.2 Hz, 2H), 1.64–1.56 (m, 4H), 1.38–1.31 (m, 4H), 1.18 (s, 9H); ^{13}C NMR (100 MHz, $CDCl_3$): δ 208.9, 178.7, 137.0, 133.7, 128.6, 127.6, 126.3, 122.2, 64.3, 47.1, 42.4, 38.8, 28.8, 28.5, 27.3, 25.8, 23.6; HRMS (EI): Calcd for $C_{21}H_{30}O_3$: 330.2195; found: 330.2193.

(*E*)-15-((*tert*-butyldimethylsilyl)oxy)-1-phenylpentadec-1-en-4-one (4m)

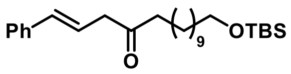

General procedure A was followed with cinnamyl acetate (34 μL, 0.2 mmol, 1.0 equiv), ^{t}BuNC (34 μL, 0.3 mmol, 1.5 equiv) and (11-((*tert*-butyldimethylsilyl)oxy)undecyl)zinc(II) bromide (0.57 M, 0.53 mL, 0.3 mmol, 1.5 equiv) prepared according to the general procedure used ((11-bromoundecyl)oxy)(*tert*-butyl)dimethylsilane with a reaction time of 0.5 h. The reaction mixture was purified by flash column chromatography (silica gel, PE:EtOAc = 200:1–150:1–100:1 v/v) to afford **4m** as a pale yellow oil (48.6 mg, 56%), TLC (PE:EtOAc, 20:1 v/v): R_f = 0.50; ^{1}H NMR (400 MHz, $CDCl_3$): δ 7.38–7.36 (m, 2H), 7.33–7.29 (m, 2H), 7.23 (tt, *J* = 7.2, 1.2 Hz, 1H), 6.47 (d, *J* = 15.6 Hz, 1H), 6.31 (dt, *J* = 16.0, 7.2 Hz, 1H), 3.59 (t, *J* = 6.8 Hz, 2H), 3.32 (dd, *J* = 7.2, 1.2 Hz, 2H), 2.48 (t, *J* = 7.6 Hz, 2H), 1.60–1.57 (m, 2H), 1.50 (quin, *J* = 6.8 Hz, 2H), 1.29–1.25 (m, 14H), 0.89 (s, 9H), 0.05 (s, 6H); ^{13}C NMR (100 MHz, $CDCl_3$): δ 209.1, 137.0, 133.7, 128.7, 127.6, 126.4, 122.3, 63.5, 47.1, 42.6, 33.0, 29.7, 29.7, 29.6, 29.6, 29.5, 29.3, 26.1, 25.9, 23.9, 18.5, -5.1; HRMS (EI): Calcd for $C_{27}H_{46}O_2Si$: 430.3267; found: 430.3268.

(*E*)-1-(6-chloropyridin-3-yl)-5-phenylpent-4-en-2-one (4n)

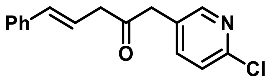

General procedure A was followed with cinnamyl acetate (34 μL, 0.2 mmol, 1.0 equiv), ^{t}BuNC (34 μL, 0.3 mmol, 1.5 equiv) and ((6-chloropyridin-3-yl)methyl)zinc(II) chloride (0.47 M, 0.64 mL, 0.3 mmol, 1.5 equiv) prepared according to the general procedure used 2-chloro-5-(chloromethyl)pyridine with a reaction time of 0.5 h. The reaction mixture was purified by flash column chromatography (silica gel, PE:EtOAc = 50:1–20:1–10:1 v/v) to afford **4n** as a pale yellow solid (38.2 mg, 70%), mp: 72–73 °C, TLC (PE:EtOAc, 5:1 v/v): R_f = 0.35; ^{1}H NMR (400 MHz, $CDCl_3$): δ 8.21 (d, *J* = 2.0 Hz, 1H), 7.51 (dd, *J* = 8.0, 2.0 Hz, 1H), 7.37–7.29 (m, 5H), 7.26–7.23 (m, 1H), 6.50 (d, *J* = 16.0 Hz, 1H), 6.28 (dt, *J* = 15.6, 7.2, 1H), 3.79 (s, 2H), 3.42 (d, *J* = 6.8 Hz, 2H); ^{13}C NMR (100 MHz, $CDCl_3$): δ 204.3, 150.4, 150.3, 140.2, 136.6, 134.7, 128.7, 128.5, 127.9, 126.4, 124.2, 121.0, 46.9, 45.1; HRMS (EI): Calcd for $C_{16}H_{14}ClNO$: 271.0764; found: 271.0762.

(*E*)-6-methyl-1-phenylhept-1-en-4-one (4o)

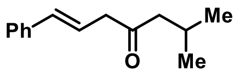

General procedure A was followed with cinnamyl acetate (34 μL, 0.2 mmol, 1.0 equiv), ^{t}BuNC (34 μL, 0.3 mmol, 1.5 equiv) and isobutylzinc(II) bromide (0.51 M, 0.59 mL, 0.3 mmol, 1.5 equiv) prepared according to the general procedure used 1-bromo-2-methylpropane with a reaction time of 0.5 h. The reaction mixture was purified by flash column chromatography (silica gel, PE:EtOAc = 200:1–150:1 v/v) to afford **4o** as a pale yellow oil (23.4 mg, 58%), TLC (PE:EtOAc, 20:1 v/v): R_f = 0.48; ^{1}H NMR (400 MHz, $CDCl_3$): δ 7.39–7.36 (m, 2H), 7.33–7.29 (m, 2H), 7.23 (tt, *J* = 7.2, 1.2 Hz, 1H), 6.47 (d, *J* = 16.0 Hz, 1H), 6.31 (dt, *J* = 16.0, 7.2 Hz, 1H), 3.30 (dd, *J* = 7.2, 1.2 Hz, 2H), 2.37 (d, *J* = 6.8 Hz, 2H), 2.17 (sep, *J* = 6.8 Hz, 1H), 0.93 (d, *J* = 6.8 Hz, 6H); ^{13}C NMR (100 MHz, $CDCl_3$): δ 208.7, 137.0, 133.7, 128.7, 127.6, 126.4, 122.2, 51.6, 47.6, 24.6, 22.7; The spectral data are consistent with those reported in the literature.[15]

(*E*)-1-cyclopentyl-4-phenylbut-3-en-1-one (4p)

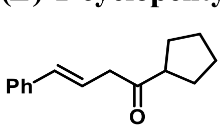

General procedure B was followed with cinnamyl acetate (34 μL, 0.2 mmol, 1.0 equiv), ^{t}BuNC (34 μL, 0.3 mmol, 1.5 equiv) and cyclopentylzinc(II) bromide (0.55 M, 0.55 mL, 0.3 mmol, 1.5 equiv) prepared according to the general procedure used bromocyclopentane with a reaction time of 1 h. The reaction mixture was purified by flash column chromatography (silica gel, PE:EtOAc = 150:1–100:1 v/v) to afford **4p** as a pale yellow oil (26.1 mg, 61%), TLC (PE:EtOAc, 20:1 v/v): R_f = 0.50; ^{1}H NMR (400 MHz, $CDCl_3$): δ 7.39–7.36 (m, 2H), 7.31 (t, *J* = 8.0 Hz, 2H), 7.22 (tt, *J* = 7.2, 1.2 Hz, 1H), 6.47 (d, *J* = 16.0 Hz, 1H), 6.34 (dt, *J* = 16.0, 7.2 Hz, 1H), 3.38 (dd, *J* = 7.2, 1.2 Hz, 2H), 2.98 (quin, *J* = 8.0 Hz, 1H), 1.88–1.74 (m, 4H), 1.70–1.54 (m, 4H); ^{13}C NMR (100 MHz, $CDCl_3$): δ 211.2, 137.1, 133.4, 128.7, 127.6, 126.3, 122.7, 51.2, 46.1, 29.0, 26.2; HRMS (EI): Calcd for $C_{15}H_{18}O$: 214.1358; found: 214.1359.

(*E*)-1-(4-bromophenyl)-5-phenylpent-4-en-2-one (4q)

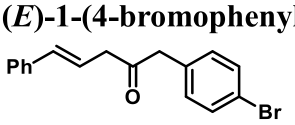

General procedure A was followed with cinnamyl acetate (34 μL, 0.2 mmol, 1.0 equiv), ^{t}BuNC (34 μL, 0.3 mmol, 1.5 equiv) and (4-bromobenzyl)zinc(II) bromide (0.40 M, 0.75 mL, 0.3 mmol, 1.5 equiv) prepared according to the general procedure used 1-bromo-4-(bromomethyl)benzene with a reaction time of 0.5 h. The reaction mixture was purified by flash column chromatography (silica gel, PE:EtOAc = 200:1–150:1 v/v) to afford **4q** as a pale yellow solid (51.9 mg, 83%), mp: 95–96 °C, TLC (PE:EtOAc, 20:1 v/v): R_f = 0.33; ^{1}H NMR (400 MHz, $CDCl_3$): δ 7.37 (dt, *J* = 8.0, 1.6 Hz, 2H), 7.28–7.20 (m, 4H), 7.17–7.13 (m, 1H), 6.99 (d, *J* = 8.4 Hz, 2H), 6.36 (d, *J* = 16.0 Hz, 1H), 6.18 (dt, *J* = 16.0, 7.2 Hz, 1H), 3.64 (s, 2H), 3.27 (dd, *J* = 7.2, 1.2 Hz, 2H); ^{13}C NMR (100 MHz, $CDCl_3$): δ 205.5, 136.8, 134.3, 132.9, 131.9, 131.4, 128.7, 127.8, 126.4, 121.6, 121.3, 48.8, 46.4; HRMS (EI): Calcd for $C_{17}H_{15}BrO$: 314.0306; found: 314.0303.

(*E*)-1-(3-bromophenyl)-5-phenylpent-4-en-2-one (4r)

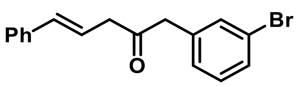

General procedure A was followed with cinnamyl acetate (34 μL, 0.2 mmol, 1.0 equiv), ^{t}BuNC (34 μL, 0.3 mmol, 1.5 equiv) and (3-bromobenzyl)zinc(II) bromide (0.58 M, 0.52 mL, 0.3 mmol, 1.5 equiv) prepared according to the general procedure used 1-bromo-3-(bromomethyl)benzene with a reaction time of 0.5 h. The reaction mixture was purified by flash column chromatography (silica gel, PE:EtOAc = 200:1–150:1 v/v) to afford **4r** as a pale yellow solid (57.2 mg, 91%), mp: 91–92 °C, TLC (PE:EtOAc, 20:1 v/v): R_f = 0.33; ^{1}H NMR (400 MHz, $CDCl_3$): δ 7.33–7.31 (m, 1H), 7.29–7.28 (m, 1H), 7.28–7.26 (m, 2H), 7.22 (t, *J* = 7.2 Hz, 2H), 7.17–7.14 (m, 1H), 7.12 (t, *J* = 7.6 Hz, 1H), 7.05 (d, *J* = 7.6 Hz, 1H), 6.36 (d, *J* = 16.0 Hz, 1H), 6.18 (dt, *J* = 16.0, 7.2 Hz, 1H), 3.65 (s, 2H), 3.28 (dd, *J* = 6.8, 1.2 Hz, 2H); ^{13}C NMR (100 MHz, $CDCl_3$): δ 205.3, 136.8, 136.1, 134.3, 132.7, 130.4, 130.3, 128.7, 128.3, 127.8, 126.4, 122.8, 121.5, 48.9, 46.4; HRMS (EI): Calcd for $C_{17}H_{15}BrO$: 314.0306; found: 314.0299.

(*E*)-1-(2-fluorophenyl)-5-phenylpent-4-en-2-one (4s)

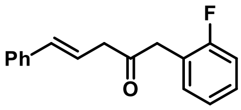

General procedure A was followed with cinnamyl acetate (34 μL, 0.2 mmol, 1.0 equiv), ^{t}BuNC (34 μL, 0.3 mmol, 1.5 equiv) and (2-fluorobenzyl)zinc(II) bromide (0.55 M, 0.55 mL, 0.3 mmol, 1.5 equiv) prepared according to the general procedure used 1-(bromomethyl)-2-fluorobenzene with a reaction time of 0.5 h. The reaction mixture was purified by flash column chromatography (silica gel, PE:EtOAc = 200:1–150:1 v/v) to afford **4s** as a pale yellow oil (30.8 mg, 61%), TLC (PE:EtOAc, 20:1 v/v): R_f = 0.47; ^{1}H NMR (400 MHz, $CDCl_3$): δ 7.29–7.27 (m, 2H), 7.24–7.20 (m, 2H), 7.18–7.09 (m, 3H), 7.05–6.97 (m, 2H), 6.38 (d, *J* = 16.0 Hz, 1H), 6.22 (dt, *J* = 16.0, 7.2 Hz, 1H), 3.73 (s, 2H), 3.32 (dd, *J* = 7.2, 1.2 Hz, 2H); ^{13}C NMR (100 MHz, $CDCl_3$): δ 204.9, 161.1 (d, J_{C-F} = 244.4 Hz), 137.0, 134.1, 131.8 (d, J_{C-F} = 4.2 Hz), 129.2 (d, J_{C-F} = 8.1 Hz), 128.7, 127.7, 126.4, 124.4 (d, J_{C-F} = 3.6 Hz), 121.7, 121.5 (d, *J* = 16.2 Hz), 115.6 (d, J_{C-F} = 21.7 Hz), 46.4, 42.7 (d, J_{C-F} = 2.3 Hz); ^{19}F NMR (376 MHz, $CDCl_3$): δ -117.0; HRMS (EI): Calcd for $C_{17}H_{15}FO$: 254.1107; found: 254.1108.

(*E*)-1-phenyloct-1-en-4-one (4t)

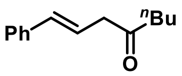

General procedure A was followed with cinnamyl acetate (34 μL, 0.2 mmol, 1.0 equiv), ^{t}BuNC (34 μL, 0.3 mmol, 1.5 equiv) and *n*-butylzinc chloride (0.38 M, 0.79 mL, 0.3 mmol, 1.5 equiv) with a reaction time of 0.5 h. The reaction mixture was purified by flash column chromatography (silica gel, PE:EtOAc = 200:1–150:1 v/v) to afford **4t** as a colorless oil (38.4 mg, 95%), TLC (PE:EtOAc, 20:1 v/v): R_f = 0.53; ^{1}H NMR (400 MHz, $CDCl_3$): δ 7.39–7.37 (m, 2H), 7.31 (t, *J* = 7.2 Hz, 2H), 7.23 (tt, *J* = 7.2, 1.2 Hz, 1H), 6.47 (d, *J* = 16.0 Hz, 1H), 6.32 (dt, *J* = 16.0, 7.2 Hz, 1H), 3.32 (dd, *J* = 7.2, 1.2 Hz, 2H), 2.49 (t, *J* = 7.2 Hz, 2H), 1.58 (quin, *J* = 7.6 Hz, 2H), 1.33 (sext, *J* = 7.6 Hz, 2H), 0.91 (t, *J* = 7.2 Hz, 3H); ^{13}C NMR (100 MHz, $CDCl_3$): δ 209.1, 137.1, 133.7, 128.7, 127.6, 126.4, 122.3, 47.1, 42.3, 25.9, 22.4, 14.0; The spectral data are consistent with those reported in the literature. [21]

(*E*)-1-(naphthalen-2-yl)oct-1-en-4-one (4u)

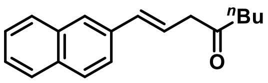

General procedure A was followed with (E)-3-(naphthalen-2-yl) allyl acetate (45.2 mg, 0.2 mmol, 1.0 equiv), ^{t}BuNC (34 µL, 0.3 mmol, 1.5 equiv) and *n*-butylzinc chloride (0.42 M, 0.71 mL, 0.3 mmol, 1.5 equiv) with a reaction time of 0.5 h. The reaction mixture was purified by flash column chromatography (silica gel, PE:EtOAc = 200:1–150:1–100:1 v/v) to afford **4u** as a pale yellow solid (48.2 mg, 96%), mp: 61–62 ºC, TLC (PE:EtOAc, 20:1 v/v): R_f = 0.47; ^{1}H NMR (400 MHz, $CDCl_3$): δ 7.81–7.78 (m, 3H), 7.72 (s, 1H), 7.61 (dd, *J* = 8.4, 1.6 Hz, 1H), 7.49–7.42 (m, 2H), 6.63 (d, *J* = 16.0 Hz, 1H), 6.45 (dt, *J* = 16.0, 7.2 Hz, 1H), 3.37 (dd, *J* = 7.2, 1.2 Hz, 2H), 2.52 (t, *J* = 7.2 Hz, 2H), 1.61 (quin, *J* = 7.2 Hz, 2H), 1.34 (sext, *J* = 7.6 Hz, 2H), 0.93 (t, *J* = 7.2 Hz, 3H); ^{13}C NMR (100 MHz, $CDCl_3$): δ 209.1, 134.5, 133.8, 133.7, 133.1, 128.3, 128.1, 127.8, 126.4, 126.2, 125.9, 123.6, 122.7, 47.1, 42.4, 25.9, 22.4, 14.0; HRMS (EI): Calcd for $C_{18}H_{20}O$: 252.1514; found: 252.1513.

(*E*)-1-(2-bromophenyl)oct-1-en-4-one (4v)

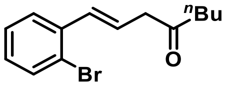

General procedure A was followed with (E)-3-(2-bromophenyl) allyl acetate (36 µL, 0.2 mmol, 1.0 equiv), ^{t}BuNC (34 µL, 0.3 mmol, 1.5 equiv) and *n*-butylzinc chloride (0.43 M, 0.70 mL, 0.3 mmol, 1.5 equiv) with a reaction time of 0.5 h. The reaction mixture was purified by flash column chromatography (silica gel, PE:EtOAc = 200:1–150:1–100:1 v/v) to afford **4v** as a pale yellow oil (40.8 mg, 73%), TLC (PE:EtOAc, 20:1 v/v): R_f = 0.44; ^{1}H NMR (400 MHz, $CDCl_3$): δ 7.46 (dd, *J* = 8.4, 1.6 Hz, 2H), 7.19 (t, *J* = 6.8 Hz, 1H), 7.02 (td, *J* = 8.0, 1.6 Hz, 1H), 6.73 (d, *J* = 15.6 Hz, 1H), 6.21 (dt, *J* = 16.0, 7.2 Hz, 1H), 3.31 (dd, *J* = 7.2, 1.2 Hz, 2H), 2.43 (t, *J* = 7.2 Hz, 2H), 1.52 (quin, *J* = 7.6 Hz, 2H), 1.26 (sext, *J* = 7.6 Hz, 2H), 0.84 (t, *J* = 7.2 Hz, 3H); ^{13}C NMR (100 MHz, $CDCl_3$): δ 208.8, 136.9, 133.0, 132.4, 129.0, 127.6, 127.2, 125.5, 123.5, 47.0, 42.5, 25.9, 22.5, 14.0; HRMS (EI): Calcd for $C_{14}H_{17}BrO$: 280.0463; found: 280.0457.

(*E*)-1-(4-iodophenyl)oct-1-en-4-one (4w)

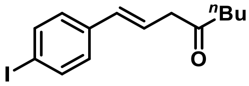

General procedure A was followed with (E)-3-(4-iodophenyl) allyl acetate (60.4 mg, 0.2 mmol, 1.0 equiv), ^{t}BuNC (34 µL, 0.3 mmol, 1.5 equiv) and *n*-butylzinc chloride (0.42 M, 0.71 mL, 0.3 mmol, 1.5 equiv) with a reaction time of 0.5 h. The reaction mixture was purified by flash column chromatography (silica gel, PE:EtOAc = 200:1–150:1–100:1 v/v) to afford **4w** as a pale yellow solid (53.3 mg, 81%), mp: 55–56 ºC, TLC (PE:EtOAc, 20:1 v/v): R_f = 0.45; ^{1}H NMR (400 MHz, $CDCl_3$): δ 7.62 (dt, *J* = 8.4, 2.0 Hz, 2H), 7.09 (dt, *J* = 8.4, 2.0 Hz, 2H), 6.38 (d, *J* = 16.0 Hz, 1H), 6.31 (dt, *J* = 16.0, 6.4 Hz, 1H), 3.30 (d, *J* = 6.0 Hz, 2H), 2.47 (t, *J* = 7.2 Hz, 2H), 1.57 (quin, *J* = 7.6 Hz, 2H), 1.31 (sext, *J* = 7.2 Hz, 2H), 0.90 (t, *J* = 7.2 Hz, 3H); ^{13}C NMR (100 MHz, $CDCl_3$): δ 208.7, 137.7, 136.6, 132.6, 128.1, 123.4, 92.8, 46.8, 42.4, 25.9, 22.4, 14.0; HRMS (EI): Calcd for $C_{14}H_{17}IO$: 328.0324; found: 328.0325.

(*E*)-1-(4-(4,4,5,5-tetramethyl-1,3,2-dioxaborolan-2-yl)phenyl)oct-1-en-4-one (4x)

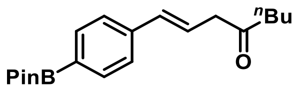

General procedure A was followed with (E)-3-(4-(4,4,5,5-tetramethyl-1,3,2-dioxaborolan-2-yl)phenyl) allyl acetate (60.4 mg, 0.2 mmol, 1.0 equiv), ^{t}BuNC (34 μL, 0.3 mmol, 1.5 equiv) and *n*-butylzinc chloride (0.40 M, 0.75 mL, 0.3 mmol, 1.5 equiv) with a reaction time of 0.5 h. The reaction mixture was purified by flash column chromatography (silica gel, PE:EtOAc = 150:1–100:1–50:1 v/v) to afford **4x** as a pale yellow oil (28.0 mg, 43%), TLC (PE:EtOAc, 20:1 v/v): R_f = 0.29; ^{1}H NMR (400 MHz, $CDCl_3$): δ 7.75 (d, *J* = 8.0 Hz, 2H), 7.36 (d, *J* = 8.4 Hz, 2H), 6.47 (d, *J* = 16.0 Hz, 1H), 6.38 (dt, *J* = 16.0, 6.8 Hz, 1H), 3.32 (d, *J* = 6.8 Hz, 2H), 2.49 (t, *J* = 7.2 Hz, 2H), 1.58 (quin, *J* = 7.6 Hz, 2H), 1.34 (s, 12H), 1.31–1.27 (m, 2H), 0.90 (t, *J* = 7.2 Hz, 3H); ^{13}C NMR (100 MHz, $CDCl_3$): δ 209.1, 139.7, 135.2, 133.7, 125.7, 123.5, 83.9, 47,1, 42.4, 25.9, 25.0, 22.4, 14.0; HRMS (EI): Calcd for $C_{20}H_{29}BO_3$: 328.2210; found: 328.2214.

(*E*)-1-(thiophen-3-yl)oct-1-en-4-one (4y)

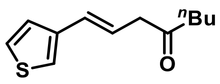

General procedure A was followed with (E)-3-(thiophen-3-yl) allyl acetate (32 μL, 0.2 mmol, 1.0 equiv), ^{t}BuNC (34 μL, 0.3 mmol, 1.5 equiv) and *n*-butylzinc chloride (0.39 M, 0.77 mL, 0.3 mmol, 1.5 equiv) with a reaction time of 0.5 h. The reaction mixture was purified by flash column chromatography (silica gel, PE:EtOAc = 200:1–150:1–100:1 v/v) to afford **4y** as a colorless oil (32.8 mg, 79%), TLC (PE:EtOAc, 20:1 v/v): R_f = 0.47; ^{1}H NMR (400 MHz, $CDCl_3$): δ 7.19 (dd, *J* = 5.2, 3.2 Hz, 1H), 7.13 (dd, *J* = 5.2, 0.8 Hz, 1H), 7.04 (dd, *J* = 2.4, 0.8 Hz, 1H), 6.40 (d, *J* = 16.0 Hz, 1H), 6.08 (dt, *J* = 16.0, 7.2 Hz, 1H), 3.20 (dd, *J* = 7.2, 1.2 Hz, 2H), 2.40 (t, *J* = 7.2 Hz, 2H), 1.50 (quin, *J* = 7.6 Hz, 2H), 1.24 (sext, *J* = 7.6 Hz, 2H), 0.83 (t, *J* = 7.6 Hz, 3H); ^{13}C NMR (100 MHz, $CDCl_3$): δ 209.3, 139.7, 127.9, 126.1, 125.0, 122.1, 121.9, 46.9, 42.3, 25.9, 22.4, 14.0; HRMS (EI): Calcd for $C_{12}H_{16}OS$: 208.0922; found: 208.0923.

(*E*)-1-(furan-2-yl)oct-1-en-4-one (4z)

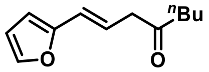

General procedure A was followed with (E)-3-(furan-2-yl) allyl acetate (32 μL, 0.2 mmol, 1.0 equiv), ^{t}BuNC (34 μL, 0.3 mmol, 1.5 equiv) and *n*-butylzinc chloride (0.40 M, 0.75 mL, 0.3 mmol, 1.5 equiv) with a reaction time of 0.5 h. The reaction mixture was purified by flash column chromatography (silica gel, PE:EtOAc = 200:1–150:1 v/v) to afford **4z** as a pale yellow oil (29.1 mg, 76%), TLC (PE:EtOAc, 20:1 v/v): R_f = 0.50; ^{1}H NMR (400 MHz, $CDCl_3$): δ 7.33 (d, *J* = 1.2 Hz, 1H), 6.35 (dd, *J* = 3.2, 1.6 Hz, 1H), 6.29 (d, *J* = 15.6 Hz, 1H), 6.22 (dt, *J* = 16.0, 6.8 Hz, 1H), 6.20 (d, *J* = 3.2 Hz, 1H), 3.27 (d, *J* = 6.4 Hz, 2H), 2.48 (t, *J* = 7.2 Hz, 2H), 1.57 (quin, *J* = 7.6 Hz, 2H), 1.31 (sext, *J* = 7.6 Hz, 2H), 0.90 (t, *J* = 7.2 Hz, 3H); ^{13}C NMR (100 MHz, $CDCl_3$): δ 208.9, 152.5, 142.0, 122.2, 121.0, 111.3, 107.6, 46.8, 42.3, 25.9, 22.4, 14.0; HRMS (EI): Calcd for $C_{12}H_{16}O_2$: 192.1150; found: 192.1149.

(*E*)-1-(1-tosyl-1H-indol-3-yl)oct-1-en-4-one (4aa)

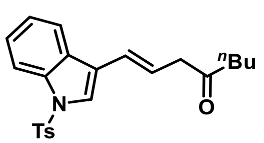

General procedure A was followed with (E)-3-(1-tosyl-1H-indol-3-yl) allyl acetate (73.8 mg, 0.2 mmol, 1.0 equiv), ^{t}BuNC (34 μL, 0.3 mmol, 1.5 equiv) and *n*-butylzinc chloride (0.29 M, 1.03 mL, 0.3 mmol, 1.5 equiv) with a reaction time of 0.5 h. The reaction mixture was purified by flash column chromatography (silica gel, PE:EtOAc = PE:EtOAc = 100:1–50:1–20:1 v/v) to afford **4aa** as a pale yellow oil (63.2 mg, 80%), TLC (PE:EtOAc, 5:1 v/v): R_f = 0.53; ^{1}H NMR (400 MHz, $CDCl_3$): δ 7.98 (d, *J* = 8.0 Hz, 1H), 7.76 (d, *J* = 8.4 Hz, 2H), 7.72 (d, *J* = 7.6 Hz, 1H), 7.56 (s, 1H), 7.24 (td, *J* = 7.2, 1.2 Hz, 1H), 7.26 (td, *J* = 7.6, 1.2 Hz, 1H), 7.21 (d, *J* = 8.0 Hz, 2H), 6.51 (d, *J* = 16.4 Hz, 1H), 6.37 (dt, *J* = 16.0, 7.2 Hz, 1H), 3.35 (dd, *J* = 7.2, 0.8 Hz, 2H), 2.50 (t, *J* = 7.2 Hz, 2H), 2.33 (s, 3H), 1.59 (quin, *J* = 7.6 Hz, 2H), 1.33 (sext, *J* = 7.6 Hz, 2H), 0.91 (t, *J* = 7.2 Hz, 3H); ^{13}C NMR (100 MHz, $CDCl_3$): δ 209.0, 145.1, 135.5, 135.1, 130.0, 129.1, 126.9, 125.0, 124.1, 123.8, 123.7, 123.6, 120.5, 120.4, 113.8, 47.3, 42.4, 25.9, 22.4, 21.6, 14.0; HRMS (EI): Calcd for $C_{23}H_{25}NO_3S$: 395.1555; found: 395.1553.

(*E*)-1-phenyloct-4-en-2-one (4ab)

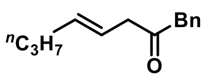

General procedure A was followed with (E)-hex-2-en-1-yl acetate (32 μL, 0.2 mmol, 1.0 equiv), ^{t}BuNC (34 μL, 0.3 mmol, 1.5 equiv) and benzylzinc bromide (0.62 M, 0.48 mL, 0.3 mmol, 1.5 equiv) prepared according to the general procedure used (bromomethyl)benzene with a reaction time of 0.5 h. The reaction mixture was purified by flash column chromatography (silica gel, PE:EtOAc = 200:1–150:1 v/v) to afford **4ab** as a colorless oil (30.9 mg, 76%), TLC (PE:EtOAc, 20:1 v/v): R_f= 0.41; *E*:*Z* = 12:1. ^{1}H NMR (400 MHz, $CDCl_3$): δ 7.27–7.23 (m, 2H), 7.21–7.16 (m, 1H), 7.14–7.10 (m, 2H), 5.51–5.37 (m, 2H), 3.64 (s, 2H), 3.07 (d, *J* = 5.2 Hz, 2H), 1.96–1.91 (m, 2H), 1.30 (sext, *J* = 7.2 Hz, 2H), 0.81 (t, *J* = 7.2 Hz, 3H); ^{13}C NMR (100 MHz, $CDCl_3$): δ 207.0, 135.5, 134.3, 129.6, 128.8, 127.1, 121.9, 49.4, 46.1, 34.8, 22.5, 13.8; HRMS (ESI): $[M+H]^+$ calcd for $C_{14}H_{19}O$: 203.1436; found: 203.1429.

(*E*)-10-phenyldec-7-en-5-one (4ac)

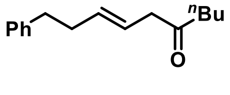

General procedure A was followed with (E)-5-phenylpent-2-en-1-yl acetate (41 μL, 0.2 mmol, 1.0 equiv), ^{t}BuNC (34 μL, 0.3 mmol, 1.5 equiv) and *n*-butylzinc chloride (0.29 M, 1.03 mL, 0.3 mmol, 1.5 equiv) with a reaction time of 0.5 h. The reaction mixture was purified by flash column chromatography (silica gel, PE:EtOAc = 200:1–150:1 v/v) to afford **4ac** as a colorless oil (37.7 mg, 82%), TLC (PE:EtOAc, 20:1 v/v): R_f = 0.52; *E*:*Z* = 13:1. ^{1}H NMR (400 MHz, $CDCl_3$): δ 7.22–7.18 (m, 2H), 7.12–7.08 (m, 3H), 5.54–5.43 (m, 2H), 3.00 (d, *J* = 5.2 Hz, 2H), 2.62 (t, *J* = 7.6 Hz, 2H), 2.32–2.28 (m, 4H), 1.45 (quin, *J* = 7.6 Hz, 2H), 1.22 (sext, *J* = 7.6 Hz, 2H), 0.82 (t, *J* = 7.2 Hz, 3H); ^{13}C NMR (100 MHz, $CDCl_3$): δ 209.8, 141.8, 134.1, 128.5, 128.4, 125.9, 122.9, 46.9, 42.0, 35.7, 34.4, 25.9, 22.4, 14.0; HRMS (EI): Calcd for $C_{16}H_{22}O$: 230.1671; found: 230.1670.

(*E*)-1-cyclohexyloct-1-en-4-one (4ad)

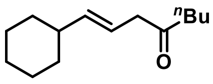

General procedure A was followed with (E)-3-cyclohexylallyl acetate (38 μL, 0.2 mmol, 1.0 equiv), ^{t}BuNC (34 μL, 0.3 mmol, 1.5 equiv) and *n*-butylzinc chloride (0.41 M, 0.73 mL, 0.3 mmol, 1.5 equiv) with a reaction time of 1 h. The reaction mixture was purified by flash column chromatography (silica gel, PE:EtOAc = 200:1–150:1–100:1 v/v) to afford **4ad** as a colorless oil (30.2 mg, 73%), TLC (PE:EtOAc, 20:1 v/v): R_f = 0.49; ^{1}H NMR (400 MHz, $CDCl_3$): δ 5.51–5.41 (m, 2H), 3.06 (d, *J* = 5.2 Hz, 2H), 2.41 (t, *J* = 7.2 Hz, 2H), 1.98–1.92 (m, 1H), 1.72–1.64 (m, 5H), 1.53 (quin, *J* = 7.6 Hz, 2H), 1.29 (sext, *J* = 7.6 Hz, 2H), 1.24–1.11 (m, 3H), 1.09–1.00 (m, 2H), 0.89 (t, *J* = 7.2 Hz, 3H); ^{13}C NMR (100 MHz, $CDCl_3$): δ 210.1, 141.1, 119.7, 47.1, 41.9, 40.8, 33.0, 26.3, 26.1, 26.0, 22.5, 14.0; HRMS (EI): Calcd for $C_{14}H_{24}O$: 208.1827; found: 208.1829.

(*E*)-2-methyl-1-phenyloct-1-en-4-one (4ae)

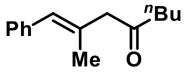

General procedure A was followed with ((E)-2-methyl-3-phenylallyl acetate (37 μL, 0.2 mmol, 1.0 equiv), ^{t}BuNC (34 μL, 0.3 mmol, 1.5 equiv) and *n*-butylzinc chloride (0.45 M, 0.67 mL, 0.3 mmol, 1.5 equiv) with a reaction time of 2 h at 50 °C. The reaction mixture was purified by flash column chromatography (silica gel, 200:1–150:1 v/v) to afford **4ae** as a colorless oil (24.6 mg, 57%), TLC (PE:EtOAc, 20:1 v/v): R_f = 0.50; ^{1}H NMR (400 MHz, $CDCl_3$): δ 7.26 (t, *J* = 7.2 Hz, 2H), 7.19–7.18 (m, 2H), 7.14 (t, *J* = 7.2 Hz, 1H), 6.30 (s, 1H), 3.17 (s, 2H), 2.43 (t, *J* = 7.2 Hz, 2H), 1.80 (d, *J* = 1.2 Hz, 3H), 1.51 (quin, *J* = 7.6 Hz, 2H), 1.25 (sext, *J* = 7.6 Hz, 2H), 0.84 (t, *J* = 7.2 Hz, 3H); ^{13}C NMR (100 MHz, $CDCl_3$): δ 209.4, 137.8, 132.6, 129.5, 129.0, 128.3, 126.6, 54.9, 41.8, 26.0, 22.5, 18.4, 14.0; HRMS (EI): Calcd for $C_{15}H_{20}O$: 216.1514; found: 216.1513.

(*E*)-3-methyl-5-phenylpent-4-en-2-one (4af)

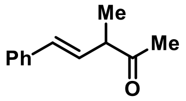

General procedure B was followed with (E)-4-phenylbut-3-en-2-yl acetate (38 μL, 0.2 mmol, 1.0 equiv), ^{t}BuNC (34 μL, 0.3 mmol, 1.5 equiv) and methyl-zinc bromide (0.33 M, 0.90 mL, 0.3 mmol, 1.5 equiv) prepared with a reaction time of 1 h. The reaction mixture was purified by flash column chromatography (silica gel, PE:EtOAc = 200:1–150:1–100:1 v/v) to afford **4af** as a colorless oil (24.0 mg, 69%), TLC (PE:EtOAc, 20:1 v/v): R_f = 0.41; ^{1}H NMR (400 MHz, $CDCl_3$): δ 7.31–7.29 (m, 2H), 7.24 (t, *J* = 7.2 Hz, 2H), 7.18–7.14 (m, 1H), 6.45 (d, *J* = 15.6 Hz, 1H), 6.11 (dd, *J* = 15.6, 8.4 Hz, 1H), 3.29 (quin, *J* = 7.2 Hz, 1H), 2.13 (s, 3H), 1.20 (d, *J* = 6.8 Hz, 3H); ^{13}C NMR (100 MHz, $CDCl_3$): δ 209.6, 136.9, 132.3, 128.9, 128.7, 127.8, 126.4, 51.5, 28.3, 16.3; The spectral data are consistent with those reported in the literature.[22]

(*E*)-3-methyl-1-phenyloct-1-en-4-one (4ag)

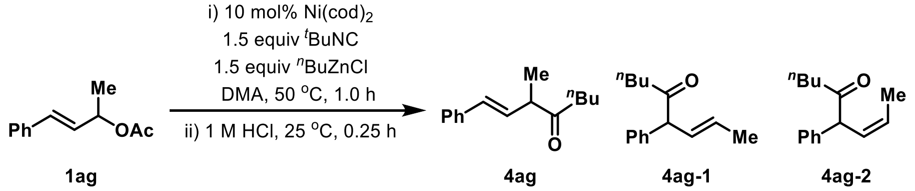

General procedure B was followed with (E)-4-phenylbut-3-en-2-yl acetate (57 μL, 0.3 mmol, 1.0 equiv), (51 μL, 0.45 mmol, 1.5 equiv) and *n*-butylzinc chloride (0.42 M, 1.07 mL, 0.45 mmol, 1.5 equiv) with a reaction time of 1 h. The reaction mixture was purified by flash column chromatography (silica gel, PE:EtOAc = 200:1–150:1 v/v) to afford a mixture of **4ag**, **4ag-1** and **4ag-2** as a colorless oil (45.2 mg, 70%). The ratio of **4ag** to **4ag-1** and **4ag-2** was detected to be 17:2:1 based on the ^{1}H NMR spectrum. Major isomer: TLC (PE:EtOAc, 20:1 v/v): R_f = 0.62; ^{1}H NMR (400 MHz, $CDCl_3$): δ 7.37 (d, *J* = 7.2 Hz, 2H), 7.32 (t, *J* = 7.2 Hz, 2H), 7.24 (t, *J* = 7.2 Hz, 1H), 6.51 (d, *J* = 16.0 Hz, 1H), 6.18 (dd, *J* = 16.0, 8.4 Hz, 1H), 3.37 (quin, *J* = 7.6 Hz, 1H), 2.60–2.44 (m, 2H), 1.56 (quin, *J* = 7.6 Hz, 2H), 1.35–1.29 (m, 2H), 1.27 (d, *J* = 6.8 Hz, 3H), 0.90 (t, *J* = 7.2 Hz, 3H); ^{13}C NMR (100 MHz, $CDCl_3$): δ 211.8, 137.0, 132.0, 129.3, 128.7, 127.7, 126.4, 50.8, 40.8, 25.9, 22.5, 16.5, 14.0; HRMS (EI): Calcd for $C_{15}H_{20}O$: 216.1514; found: 216.1515.

(*E*)-3-ethyl-1-phenyloct-1-en-4-one (4ah)

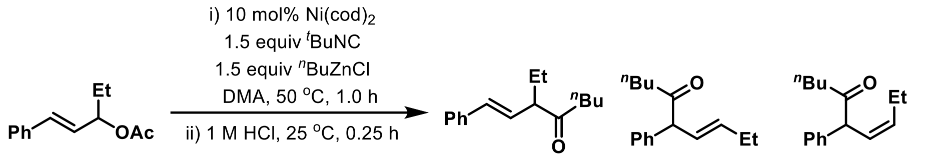

1ah **4ah** **4ah-1** **4ah-2**

General procedure B was followed with (E)-1-phenylpent-1-en-3-yl acetate (38 μL, 0.2 mmol, 1.0 equiv), ^{t}BuNC (34 μL, 0.3 mmol, 1.5 equiv) and *n*-butylzinc chloride (0.36 M, 0.83 mL, 0.3 mmol, 1.5 equiv) with a reaction time of 1 h. The reaction mixture was purified by flash column chromatography (silica gel, PE:EtOAc = 150:1–100:1 v/v) to afford a mixture of **4ah**, **4ah-1** and **4ah-2** as a colorless oil (38.7 mg, 84%). The ratio of **4ah** to **4ah-1** and **4ah-2** was detected to be 8:1:1 based on the ^{1}H NMR spectrum. Major isomer: TLC (PE:EtOAc, 20:1 v/v): R_f = 0.65; ^{1}H NMR (400 MHz, $CDCl_3$): δ 7.37 (d, *J* = 7.2 Hz, 2H), 7.31 (t, *J* = 7.2 Hz, 2H), 7.24 (t, *J* = 7.2 Hz, 1H), 6.49 (d, *J* = 16.0 Hz, 1H), 6.10 (dd, *J* = 16.0, 9.6 Hz, 1H), 3.17 (dt, *J* = 8.8, 7.2 Hz, 1H), 2.58–2.42 (m, 2H), 1.64–1.46 (m, 4H), 1.30 (sext, *J* = 7.2 Hz, 2H), 0.91 (t, *J* = 7.2 Hz, 3H), 0.89 (t, *J* = 7.2 Hz, 3H); ^{13}C NMR (100 MHz, $CDCl_3$): δ 211.6, 137.0, 133.0, 128.7, 128.1, 127.7, 126.4, 58.8, 41.7, 25.8, 24.7, 22.5, 14.0, 12.0; HRMS (EI): Calcd for $C_{16}H_{22}O$: 230.1671; found: 230.1670.

(*S*,*E*)-10,14-dimethylpentadeca-7,13-dien-5-one (4ai)

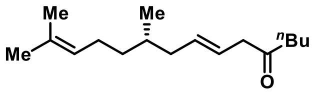

General procedure A was followed with (S,E)-5,9-dimethyldeca-2,8-dien-1-yl acetate (51 μL, 0.2 mmol, 1.0 equiv), ^{t}BuNC (34 μL, 0.3 mmol, 1.5 equiv) and *n*-butylzinc chloride (0.41 M, 0.73 mL, 0.3 mmol, 1.5 equiv) with a reaction time of 0.5 h. The reaction mixture was purified by flash column chromatography (silica gel, PE:EtOAc = 200:1–150:1 v/v) to afford **4ai** as a colorless oil (35.1 mg, 70%), TLC (PE:EtOAc, 20:1 v/v): R_f = 0.67; ^{1}H NMR (400 MHz, $CDCl_3$): δ 5.59–5.45 (m, 2H), 5.10–5.06 (m, 1H), 3.09 (d, *J* = 5.2 Hz, 2H), 2.42 (t, *J* = 7.6 Hz, 2H), 2.09–2.02 (m, 1H), 2.00–1.93 (m, 2H), 1.91–1.84 (m, 1H), 1.67 (s, 3H), 1.59 (s, 3H), 1.54 (quin, *J* = 7.6 Hz, 2H),

1.50–1.44 (m, 1H), 1.35–1.25 (m, 3H), 1.17–1.08 (m, 1H), 0.89 (t, *J* = 7.2 Hz, 3H), 0.86 (d, *J* = 6.4 Hz, 3H); ^{13}C NMR (100 MHz, $CDCl_3$): δ 210.0, 133.8, 131.3, 124.9, 123.3, 47.1, 42.0, 40.1, 36.7, 32.7, 25.9, 25.9, 25.7, 22.5, 19.5, 17.8, 14.0; HRMS (EI): Calcd for $C_{17}H_{30}O$: 250.2297; found: 250.2296.

(*R*,*E*)-9-(6-methoxynaphthalen-2-yl)dec-7-en-5-one (4aj)

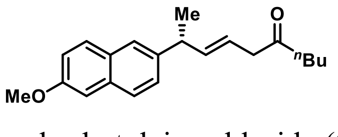

General procedure B was followed with (*R*,*E*)-4-(6-methoxynaphthalen-2-yl)pent-2-en-1-yl acetate (56.8 mg, 0.2 mmol, 1.0 equiv), *t*BuNC (34 µL, 0.3 mmol, 1.5 equiv) and *n*-butylzinc chloride (0.39 M, 0.77 mL, 0.3 mmol, 1.5 equiv) with a reaction time of 1 h. The reaction mixture was purified by flash column chromatography (silica gel, PE:EtOAc = 200:1–150:1–100:1 v/v) to afford **4aj** as a colorless oil (43.4 mg, 70%), TLC (PE:EtOAc, 20:1 v/v): R_f = 0.43; ^{1}H NMR (400 MHz, $CDCl_3$): δ 7.69 (d, *J* = 8.4 Hz, 2H), 7.56 (s, 1H), 7.32 (dd, *J* = 8.4, 1.6 Hz, 1H), 7.15–7.12 (m, 2H), 5.79 (ddt, *J* = 15.2, 6.8, 1.2 Hz, 1H), 5.63 (dtd, *J* = 15.2, 7.2, 1.2 Hz, 1H), 3.91 (s, 3H), 3.62 (quin, *J* = 6.8 Hz, 1H), 3.14 (d, *J* = 6.8 Hz, 2H), 2.42 (t, *J* = 7.2 Hz, 2H), 1.55 (quin, *J* = 7.6 Hz, 2H), 1.45 (d, *J* = 6.8 Hz, 3H), 1.30 (sext, *J* = 7.6 Hz, 2H), 0.90 (t, *J* = 7.2 Hz, 3H); ^{13}C NMR (100 MHz, $CDCl_3$): δ 209.7, 157.4, 140.9, 139.6, 133.3, 129.2, 129.2, 127.0, 126.8, 125.1, 121.3, 118.8, 105.7, 55.4, 46.8, 42.3, 42.1, 25.9, 22.4, 21.2, 14.0; HRMS (EI): Calcd for $C_{21}H_{26}O_2$: 310.1933; found: 310.1935.

(*E*)-9-(4-isobutylphenyl)dec-7-en-5-one (4ak)

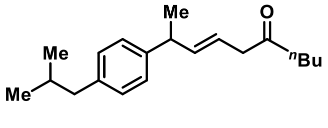

General procedure B was followed with (E)-4-(4-isobutylphenyl)pent-2-en-1-yl acetate (55 µL, 0.2 mmol, 1.0 equiv), *t*BuNC (34 µL, 0.3 mmol, 1.5 equiv) and *n*-butylzinc chloride (0.39 M, 0.77 mL, 0.3 mmol, 1.5 equiv) with a reaction time of 0.5 h. The reaction mixture was purified by flash column chromatography (silica gel, PE:EtOAc = 200:1–150:1 v/v) to afford **4ak** as a colorless oil (42.0 mg, 73%), TLC (PE:EtOAc, 20:1 v/v): R_f = 0.53; ^{1}H NMR (400 MHz, $CDCl_3$): δ 7.12–7.05 (m, 4H), 5.70 (ddt, *J* = 15.6, 6.8, 1.2 Hz, 1H), 5.56 (dtd, *J* = 15.6, 6.8, 1.2 Hz, 1H), 3.44 (quin, *J* = 6.8 Hz, 1H), 3.10 (d, *J* = 6.8 Hz, 2H), 2.43 (d, *J* = 7.2 Hz, 2H), 2.40 (t, *J* = 7.2 Hz, 2H), 1.88–1.78 (m, 1H), 1.53 (quin, *J* = 7.6 Hz, 2H), 1.34 (d, *J* = 7.2 Hz, 3H), 1.28 (sext, *J* = 7.6 Hz, 2H), 0.89 (d, *J* = 6.8 Hz, 6H), 0.88 (t, *J* = 7.2 Hz, 3H); ^{13}C NMR (100 MHz, $CDCl_3$): δ 209.8, 142.9, 139.8, 139.6, 129.3, 126.9, 120.9, 46.8, 45.1, 42.0, 30.3, 25.9, 22.5, 22.4, 21.2, 14.0; HRMS (EI): Calcd for $C_{20}H_{30}O$: 286.2297; found: 286.2298.

(7*E*,16*Z*)-pentacosa-7,16-dien-5-one (4al)

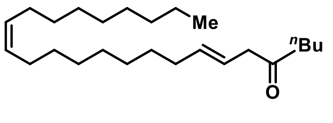

General procedure B was followed with (2E,11Z)-icosa-2,11-dien-1-yl acetate (78 µL, 0.2 mmol, 1.0 equiv), *t*BuNC (34 µL, 0.3 mmol, 1.5 equiv) and *n*-butylzinc chloride (0.42 M, 0.71 mL, 0.3 mmol, 1.5 equiv) with a reaction time of 0.5 h. The reaction mixture was purified by flash column chromatography (silica gel, PE:EtOAc = 200:1–150:1 v/v) to afford **4al** as a colorless oil (51.2 mg, 71%), TLC (PE:EtOAc, 20:1 v/v): R_f = 0.64;

^{1}H NMR (400 MHz, CDCl3): δ 5.57–5.46 (m, 2H), 5.38–5.30 (m, 2H), 3.08 (d, *J* = 5.6 Hz, 2H), 2.42 (t, *J* = 7.6 Hz, 2H), 2.04–1.98 (m, 6H), 1.54 (quin, *J* = 7.6 Hz, 2H), 1.31–1.25 (m, 24H), 0.89 (t, *J* = 7.2 Hz, 3H), 0.88 (t, *J* = 7.2 Hz, 3H); ^{13}C NMR (100 MHz, CDCl3): δ 210.0, 135.3, 130.1, 130.0, 122.0, 47.0, 42.0, 32.7, 32.1, 29.9, 29.9, 29.8, 29.8, 29.7, 29.5, 29.5, 29.4, 29.4, 29.3, 27.4, 26.0, 22.8, 22.5, 14.3, 14.0; HRMS (EI): Calcd for $C_{25}H_{46}O$: 362.3549; found: 362.3546.

(*R*,*E*)-8-((3*R*,5*R*,6*S*,8*S*,9*S*,10*R*,13*R*,14*S*,17*R*)-3,6-dimethoxy-10,13-dimethylhexadecahydro-1H-cyclopenta[a]phenanthren-17-yl)-1-phenylnon-4-en-2-one (4am)

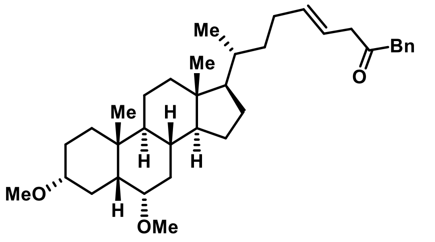

General procedure A was followed with (R,E)-6-((3R,5R,6S,8S,9S,10R,13R,14S,17R)-3,6-dimethoxy-10,13-dimethylhexadecahydro-1H-cyclopenta-[a]phenanthren-17-yl) hept-2-en-1-yl acetate (49.4 mg, 0.104 mmol, 1.0 equiv), ^{t}BuNC (18 μL, 0.156 mmol, 1.5 equiv) and benzylzinc bromide (0.62 M, 0.25 mL, 0.156 mmol, 1.5 equiv) prepared according to the general procedure used (bromomethyl)benzene with a reaction time of 0.5 h. The reaction mixture was purified by flash column chromatography (silica gel, PE:EtOAc = 50:1–30:1–20:1 v/v) to afford **4am** as a pale yellow oil (35.4 mg, 64%), TLC (PE:EtOAc, 5:1 v/v): R_f = 0.50; ^{1}H NMR (400 MHz, CDCl3): δ 7.40–7.36 (m, 2H), 7.34–7.28 (m, 2H), 7.25 (d, *J* = 7.2 Hz, 1H), 5.61–5.49 (m, 2H), 3.76 (s, 2H), 3.60–3.55 (m, 1H), 3.41 (s, 3H), 3.35 (s, 3H), 3.26–3.20 (m, 1H), 3.18 (d, *J* = 4.8 Hz, 2H), 2.15–1.95 (m, 4H), 1.89–1.73 (m, 6H), 1.66–1.60 (m, 1H), 1.48–1.39 (m, 6H), 1.19–1.07 (m, 9H), 0.96 (s, 3H), 0.95 (d, *J* = 4.4 Hz, 3H), 0.68 (s, 3H); ^{13}C NMR (100 MHz, CDCl3): δ 207.0, 136.1, 134.3, 129.6, 128.8, 127.1, 121.4, 80.3, 76.8, 56.3, 56.3, 55.8, 55.7, 49.4, 46.1, 45.0, 43.0, 40.2, 40.1, 36.1, 35.8, 35.5, 35.5, 34.9, 31.8, 29.5, 28.8, 28.4, 27.1, 25.6, 24.3, 23.8, 20.9, 18.6, 12.1; HRMS (EI): Calcd for $C_{36}H_{54}O_3$: 534.4073; found: 534.4071.

(*E*)-1-(1-(4-chlorobenzoyl)-5-methoxy-2-methyl-1H-indol-3-yl)non-2-en-5-one (4an)

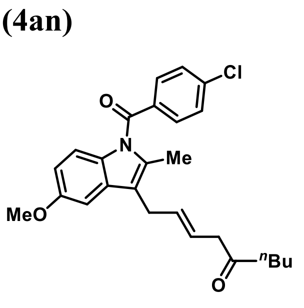

General procedure A was followed with 1-(1-(4-chlorobenzoyl)-5-methoxy-2-methyl-1H-indol-3-yl)but-3-en-2-yl acetate (82.2 mg, 0.2 mmol, 1.0 equiv), ^{t}BuNC (34 μL, 0.3 mmol, 1.5 equiv) and *n*-butylzinc chloride (0.36 M, 0.83 mL, 0.3 mmol, 1.5 equiv) with a reaction time of 0.5 h. The reaction mixture was purified by flash column chromatography (silica gel, PE:EtOAc = 100:1–50:1–25:1 v/v) to afford **4an** as a yellow oil (62.3 mg, 71%), TLC (PE:EtOAc, 10:1 v/v): R_f = 0.39; ^{1}H NMR (400 MHz, CDCl3): δ 7.65 (dt, *J* = 8.8, 2.4 Hz, 2H), 7.46 (dt, *J* = 8.8, 2.4 Hz, 2H), 6.91 (d, *J* = 2.8 Hz, 1H), 6.89 (d, *J* = 9.2 Hz, 1H), 6.65 (dd, *J* = 8.8, 2.4 Hz, 1H), 5.70–5.60 (m, 2H), 3.82 (s, 3H), 3.40 (d, *J* = 3.2 Hz, 2H), 3.11 (d, *J* = 4.4 Hz, 2H), 2.39 (t, *J* = 7.2 Hz, 2H), 2.31 (s, 3H), 1.52 (quin, *J* = 7.6 Hz, 2H), 1.27 (sext, *J* = 7.6 Hz, 2H), 0.87 (t, *J* = 7.6 Hz, 3H); ^{13}C NMR (100 MHz, CDCl3): δ 209.4, 168.4, 156.0, 139.2,

134.5, 131.6, 131.2, 131.1, 131.1, 129.2, 123.5, 117.4, 115.1, 111.3, 101.7, 55.8, 46.5, 42.2, 27.4, 25.9, 13.9, 13.4; HRMS (EI): Calcd for $C_{26}H_{28}ClNO_3$: 437.1758; found: 437.1760.

(*E*)-11-(((*R*)-2,5,7,8-tetramethyl-2-((4*R*,8*R*)-4,8,12-trimethyltridecyl)chroman-6-yl)oxy)undec-7-en-5-one (4ao)

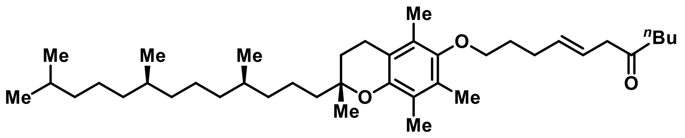

General procedure A was followed with (E)-6-(((R)-2,5,7,8-tetramethyl-2-((4R,8R)-4,8,12-trimethyltridecyl)chroman-6-yl)oxy)hex-2-en-1-yl acetate (114.1 mg, 0.2 mmol, 1.0 equiv), ^{t}BuNC (34 μL, 0.3 mmol, 1.5 equiv) and *n*-butylzinc chloride (0.44 M, 0.68 mL, 0.3 mmol, 1.5 equiv) with a reaction time of 1 h. The reaction mixture was purified by flash column chromatography (silica gel, PE:EtOAc = 200:1–150:1 v/v) to afford **4ao** as a colorless oil (59.8 mg, 50%), TLC (PE:EtOAc, 20:1 v/v): R_f = 0.53; ^{1}H NMR (400 MHz, $CDCl_3$): δ 5.65–5.55 (m, 2H), 3.63 (t, *J* = 6.4 Hz, 2H), 3.11 (d, *J* = 3.6 Hz, 2H), 2.57 (t, *J* = 6.8 Hz, 2H), 2.43 (t, *J* = 7.2 Hz, 2H), 2.31–2.26 (m, 2H), 2.16 (s, 3H), 2.11 (s, 3H), 2.08 (s, 3H), 1.87 (quin, *J* = 6.8 Hz, 2H), 1.83–1.71 (m, 2H), 1.60–1.49 (m, 6H), 1.42–1.35 (m, 3H), 1.34–1.25 (m, 8H), 1.23 (s, 3H), 1.16–1.01 (m, 8H), 0.90 (t, *J* = 7.2 Hz, 3H), 0.87 (d, *J* = 6.8 Hz, 6H), 0.84 (d, *J* = 6.8 Hz, 6H); ^{13}C NMR (100 MHz, $CDCl_3$): δ 209.8, 148.4, 147.8, 134.4, 127.9, 125.9, 122.9, 122.8, 117.6, 74.9, 72.4, 46.9, 42.1, 40.2 (d, *J* = 3.8 Hz), 39.5, 37.7, 37.6, 37.5, 37.4, 32.9 (dd, *J* = 9.7, 1.9 Hz), 31.4 (d, *J* = 4.8 Hz), 30.0, 29.5, 28.1, 26.0, 25.0 (d, *J* = 1.4 Hz), 24.6, 24.0, 22.9, 22.8, 22.5, 21.2, 20.8, 19.9, 19.8, 19.8, 14.0, 12.9, 12.0, 11.9; HRMS (EI): Calcd for $C_{40}H_{68}O_3$: 596.5168; found: 596.5173.

Supplementary Notes

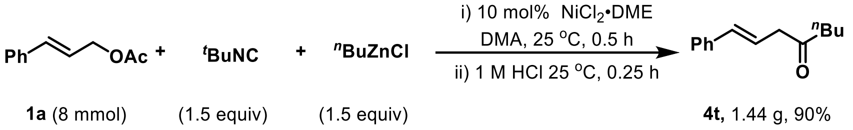

An oven-dried Schlenk tube charged with $NiCl_2$·DME (175.8 mg, 0.8 mmol, 10 mol%) was evacuated and backfilled with N_2 (This process was repeated for three times). DMA (8.0 mL, 1 M) was added into the reaction mixture. To this solution was subsequently added allylic acetate **1a** (1.36 mL, 8 mmol, 1.0 equiv), ^{t}BuNC (1.36 mL, 12 mmol, 1.5 equiv) and ^{n}BuZnCl (30 mL, 12 mmol, 1.5 equiv). The tube was equipped with a balloon filled with N_2 at 25 °C for 0.5 h. The mixture was added 1 M HCl aq. (10 mL) and stirred at room temperature for 0.25 h. The mixture was then extracted three times with EtOAc and separated organic layer was washed with brine, dried over anhydrous Na_2SO_4, and concentrated under reduced pressure. The reaction mixture was purified by flash column chromatography (silica gel, PE:EtOAc = 200:1–150:1 v/v) to afford **4t** as a pale yellow oil (1.44 g, 90%).

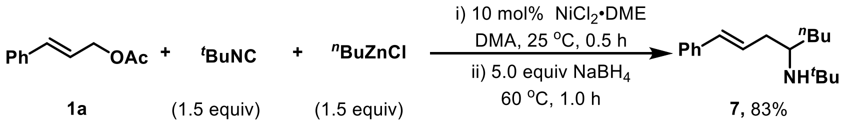

An oven-dried Schlenk tube charged with $NiCl_2$·DME (6.6 mg, 0.03 mmol, 10 mol%) was evacuated and backfilled with N_2 (This process was repeated for three times). DMA (3 mL, 0.1 M) was added into the reaction mixture. To this solution was subsequently added allylic acetate **1a** (51 µL, 0.3 mmol, 1.0 equiv), ^{t}BuNC (51 µL, 0.45 mmol, 1.5 equiv) and ^{n}BuZnCl (1.80 mL, 0.45 mmol, 1.5 equiv). The tube was equipped with a balloon filled with N_2 at 25 °C for 0.5 h. The mixture was then added $NaBH_4$ (56.7 mg, 1.5 mmol, 5.0 equiv) and stirred at 60 °C for 1 h. The reaction was then quenched with H_2O, the aqueous layer was extracted three times with EtOAc and separated organic layer was washed with brine, dried over Na_2SO_4 and concentrated under reduced pressure. The crude residue was purified by column chromatography (silica gel, PE:EA = 20:1–10:1–5:1 v/v) to afford the desired product **7** as a colorless oil (64.3 mg, 83%), TLC (EtOAc:MeOH, 20:1 v/v): R_f = 0.42; ^{1}H NMR (400 MHz, $CDCl_3$): δ 7.37–7.34 (m, 2H), 7.30 (t, J = 8.0 Hz, 2H), 7.20 (tt, J = 7.2, 1.6 Hz, 1H), 6.41 (d, J = 16.0 Hz, 1H), 6.21 (dt, J = 15.6, 7.2 Hz, 1H), 2.69 (quin, J = 6.0 Hz, 1H), 2.39–2.32 (m, 1H), 2.29–2.21 (m, 1H), 1.38–1.29 (m, 6H), 1.10 (s, 9H), 0.91 (t, J = 7.2 Hz, 3H); ^{13}C NMR (100 MHz, $CDCl_3$): δ 137.8, 132.1, 128.6, 128.5, 127.1, 126.1, 51.9, 51.2, 41.0, 37.3, 30.2, 28.7, 23.1, 14.3; HRMS (ESI): $[M+H]^+$ calcd for $C_{18}H_{30}N$: 260.2378; found: 260.2385.

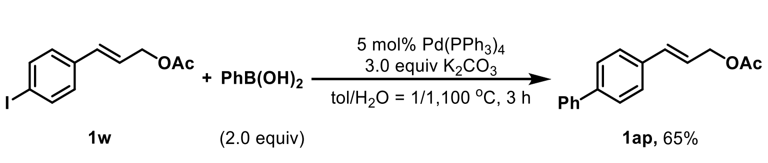

An oven-dried Schlenk tube containing a stirring bar was charged with $Pd(PPh_3)_4$

(11.5 mg, 0.01 mmol, 5 mol%), K_2CO_3 (82.9 mg, 0.6 mmol, 3.0 equiv), **1w** (60.4 mg, 0.2 mmol, 1.0 equiv), $PhB(OH)_2$ (48.8 mg, 0.4 mmol, 2.0 equiv). Then the Schlenk tube was evacuated and backfilled with N_2 (This process was repeated for three times). tol:H_2O (1 mL:1 mL) was then added and the tube was equipped with a balloon filled with N_2 at 100 °C for 3 h. The reaction mixture was quenched with sat. aq. NH_4Cl and was filtered through Celite. The aqueous layer was extracted three times with EtOAc and separated organic layer was washed with brine, dried over Na_2SO_4 and concentrated under reduced pressure. The crude residue was purified by column chromatography (silica gel, PE:EA = 200:1–150:1–100:1 v/v) to afford the desired product **1ap** as a white solid (32.5 mg, 64%), TLC (PE:EtOAc, 20:1 v/v): R_f = 0.48; ^{1}H NMR (400 MHz, $CDCl_3$): δ 7.62–7.57 (m, 4H), 7.48–7.43 (m, 4H), 7.35 (tt, *J* = 7.2, 1.2 Hz, 1H), 6.70 (d, *J* = 15.6 Hz, 1H), 6.34 (dt, *J* = 16.0, 6.4 Hz, 1H), 4.76 (dd, *J* = 6.4, 1.2 Hz, 2H), 2.12 (s, 3H); ^{13}C NMR (100 MHz, $CDCl_3$): δ 171.0, 141.0, 140.7, 135.3, 133.9, 128.9, 127.5, 127.4, 127.2, 127.1, 123.4, 65.2, 21.2.

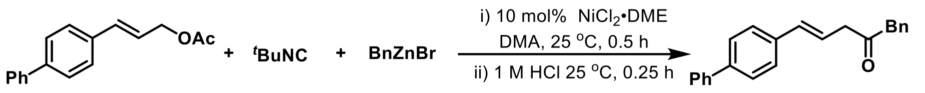

S1 (1.5 equiv) (1.5 equiv) **4ap**, 89%

An oven-dried Schlenk tube charged with $NiCl_2$·DME (2.6 mg, 0.012 mmol, 10 mol%) was evacuated and backfilled with N_2 (This process was repeated for three times). DMA (1.2 mL, 0.1 M) was added into the reaction mixture. To this solution was subsequently added allylic acetate **S1** (29.3 mg, 0.116 mmol, 1.0 equiv), ^{t}BuNC (20 μL, 0.174 mmol, 1.5 equiv) and BnZnBr (0.28 mL, 0.174 mmol, 1.5 equiv). The tube was equipped with a balloon filled with N_2 at 25 °C for 0.5 h. The mixture was added 1 M HCl aq. (1.2 mL) and stirred at room temperature for 0.25 h. The mixture was then extracted three times with EtOAc and separated organic layer was washed with brine, dried over anhydrous Na_2SO_4, and concentrated under reduced pressure to yield the crude product, which was purified by column chromatography (silica gel, PE:EA = 200:1–150:1 v/v) to afford **4ap** as a pale yellow solid (32.2 mg, 89%), mp: 102–103 °C. TLC (PE:EtOAc, 20:1 v/v): R_f = 0.30; ^{1}H NMR (400 MHz, $CDCl_3$): δ 7.52–7.49 (m, 2H), 7.46 (dt, *J* = 8.0, 1.6 Hz, 2H), 7.37–7.32 (m, 4H), 7.29–7.23 (m, 3H), 7.20 (tt, *J* = 7.2, 1.6 Hz, 1H), 7.16–7.14 (m, 2H), 6.37 (d, *J* = 16.0 Hz, 1H), 6.23 (dt, *J* = 16.0, 6.8 Hz, 1H), 3.69 (s, 2H), 3.30 (dd, *J* = 7.2, 1.2 Hz, 2H); ^{13}C NMR (100 MHz, $CDCl_3$): δ 206.2, 140.8, 140.4, 136.0, 134.1, 133.6, 129.7, 128.9, 128.9, 127.4, 127.3, 127.2, 127.0, 126.8, 122.1, 49.8, 46.2; HRMS (EI): Calcd for $C_{23}H_{20}O$: 312.1514; found: 312.1515.

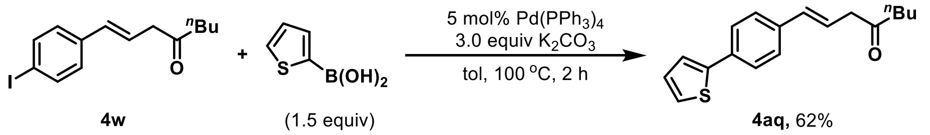

4w (1.5 equiv) **4aq**, 62%

An oven-dried Schlenk tube containing a stirring bar was charged with $Pd(PPh_3)_4$ (10.7 mg, 0.009 mmol, 5 mol%), K_2CO_3 (77 mg, 0.555 mmol, 3.0 equiv), **4w** (60.9 mg, 0.185 mmol, 1.0 equiv), thiophen-2-ylboronic acid (35.6 mg, 0.278 mmol, 1.5 equiv).

Then the Schlenk tube was evacuated and backfilled with N_2 (This process was repeated for three times). tol (1.8 mL, 0.1 M) was then added and the tube was equipped with a balloon filled with N_2 at 100 °C for 2 h. The reaction mixture was quenched with sat. aq. NH_4Cl and was filtered through Celite. The aqueous layer was extracted three times with EtOAc and separated organic layer was washed with brine, dried over Na_2SO_4 and concentrated under reduced pressure. The crude residue was purified by column chromatography (silica gel, PE:EA = 200:1–150:1–100:1 v/v) to afford the desired product **4aq** as a pale yellow solid (32.5 mg, 62%), mp: 117–118 °C. TLC (PE:EtOAc, 20:1 v/v): R_f = 0.43; ^{1}H NMR (400 MHz, $CDCl_3$): δ 7.56 (dt, *J* = 8.4, 2.0 Hz, 2H), 7.37 (dt, *J* = 8.4, 1.6 Hz, 2H), 7.31 (dd, *J* = 3.6, 1.2 Hz, 1H), 7.27 (dd, *J* = 5.2, 1.2 Hz, 1H), 7.08 (dd, *J* = 5.2, 3.6 Hz, 1H), 6.47 (d, *J* = 16.0 Hz, 1H), 6.34 (dt, *J* = 16.0, 6.8 Hz, 1H), 3.33 (dd, *J* = 7.2, 1.2 Hz, 2H), 2.50 (t, *J* = 7.2 Hz, 2H), 1.59 (quin, *J* = 7.6 Hz, 2H), 1.34 (quin, *J* = 7.2 Hz, 2H), 0.92 (t, *J* = 7.2 Hz, 3H); ^{13}C NMR (100 MHz, $CDCl_3$): δ 209.1, 144.2, 136.2, 133.7, 133.1, 128.2, 126.9, 126.1, 124.9, 123.1, 122.5, 47.1, 42.4, 26.0, 22.5, 14.0; HRMS (EI): Calcd for $C_{18}H_{20}OS$: 284.1235; found: 284.1236.

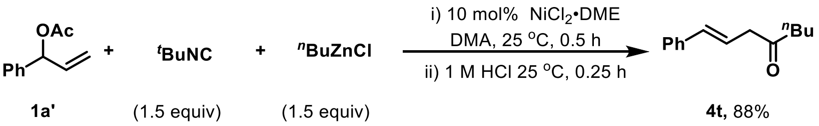

An oven-dried Schlenk tube charged with $NiCl_2$·DME (4.4 mg, 0.02 mmol, 10 mol%) was evacuated and backfilled with N_2 (This process was repeated for three times). DMA (2 mL, 0.1 M) was added into the reaction mixture. To this solution was subsequently added allylic acetate **1ap** (34 μL, 0.2 mmol, 1.0 equiv), ^{t}BuNC (34 μL, 0.3 mmol, 1.5 equiv) and ^{n}BuZnCl (0.72 mL, 0.3 mmol, 1.5 equiv). The tube was equipped with a balloon filled with N_2 at 25 °C for 0.5 h. The mixture was added 1 M HCl aq. (2 mL) and stirred at room temperature for 0.25 h. The mixture was then extracted three times with EtOAc and separated organic layer was washed with brine, dried over anhydrous Na_2SO_4, and concentrated under reduced pressure to yield the crude product, which was purified by column chromatography (silica gel, PE:EA = 200:1–150:1 v/v) to afford **4t** as a colorless oil (35.7 mg, 88%).

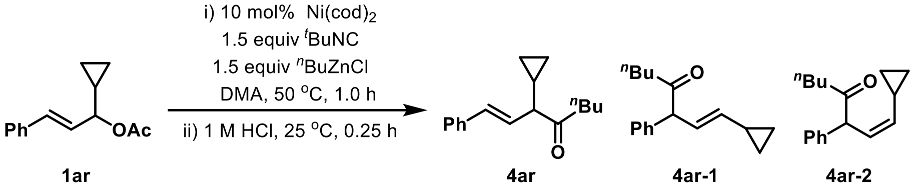

An oven-dried Schlenk tube charged with $Ni(cod)_2$ (5.6 mg, 0.02 mmol, 10 mol%) (weighed in the glove box), DMA (2 mL, 0.1 M) was added into the reaction mixture. To this solution was subsequently added allylic acetate **1ar** (42 μL, 0.2 mmol, 1.0 equiv), ^{t}BuNC (34 μL, 0.3 mmol, 1.5 equiv) and ^{n}BuZnCl (0.75 mL, 0.3 mmol, 1.5 equiv). The tube was equipped with a balloon filled with N_2 at 50 °C for 1 h. The mixture was added 1 M HCl aq. (2 mL) and stirred at room temperature for 0.25 h. The mixture was then extracted three times with EtOAc and separated organic layer was washed with brine, dried over anhydrous Na_2SO_4, and concentrated under reduced pressure to yield the crude product, which was purified by silica gel flash column chromatography (PE:EA = PE–200:1 v/v) to afford a mixture of **4ar**, **4ar-1** and **4ar-2** as a pale yellow oil (29.4 mg, 61%). The ratio of **4ar** to **4ar-1** and **4ar-2** was detected to be 11:1:1 based on the ^{1}H NMR spectrum. Major isomer: TLC (PE:EtOAc, 20:1 v/v): R_f = 0.53; ^{1}H NMR (400 MHz, $CDCl_3$): δ 7.39–7.36 (m, 2H), 7.33–7.29 (m, 2H), 7.25–7.20 (m, 1H), 6.47 (d, J = 16.0 Hz, 1H), 6.25 (dd, J = 16.0, 8.4 Hz, 1H), 2.62–2.40 (m, 3H), 1.57 (quin, J = 7.6 Hz, 2H), 1.36–1.29 (m, 2H), 1.18–1.10 (m, 1H), 0.90 (t, J = 7.2 Hz, 3H), 0.70–0.55 (m, 2H), 0.35–0.18 (m, 2H); ^{13}C NMR (100 MHz, $CDCl_3$): δ 211.3, 137.0, 132.3, 128.7, 127.7, 127.2, 126.4, 61.4, 41.3, 25.8, 22.5, 14.1, 13.0, 4.4, 3.7; HRMS (EI): Calcd for $C_{17}H_{22}O$: 242.1671; found: 242.1669.

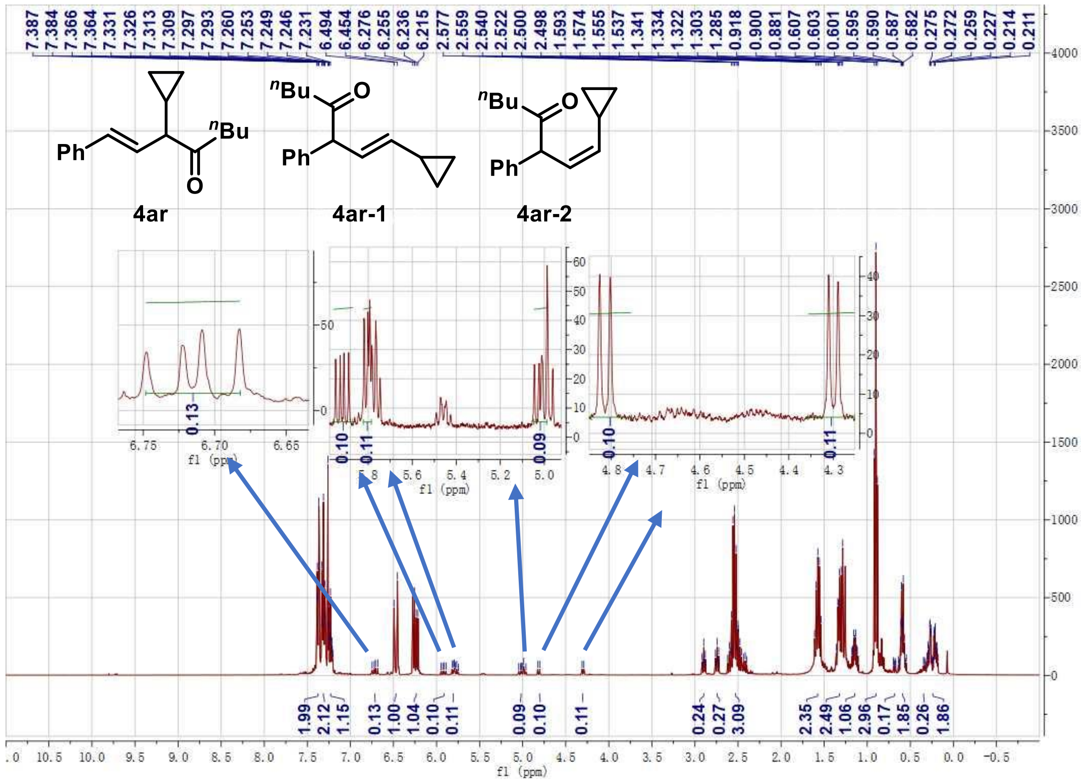

Supplementary Figure 1. ^{1}**H NMR spectrum of a isomer mixture of 4ar in** $CDCl_3$

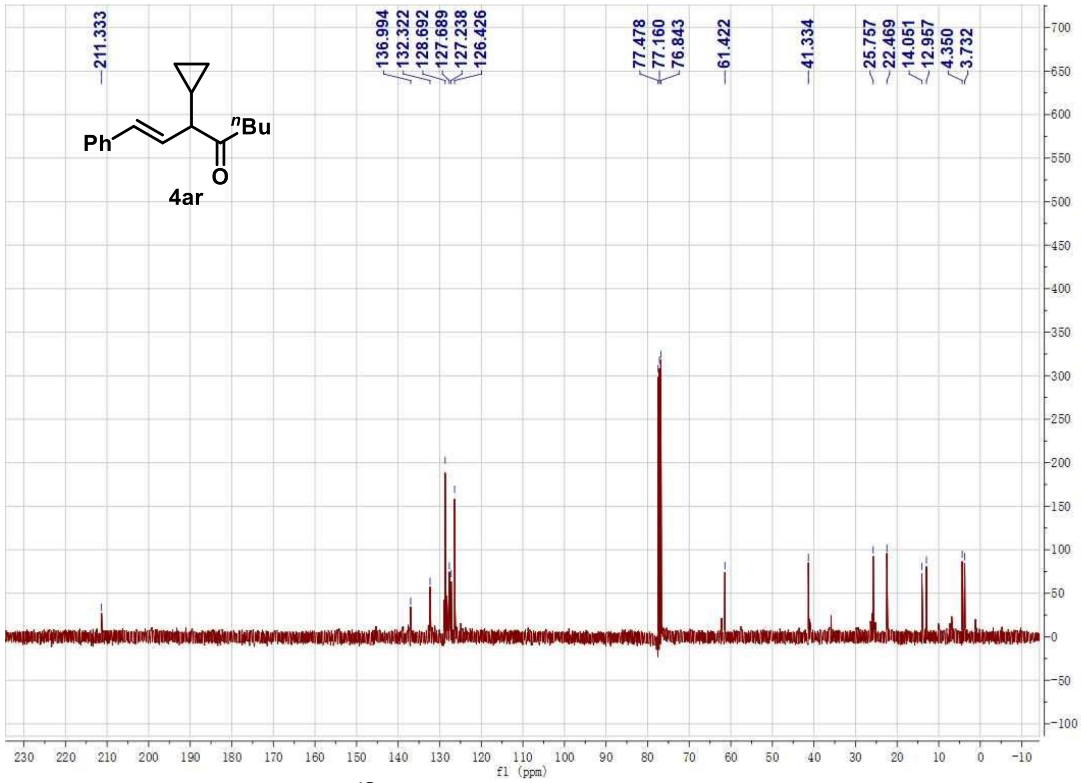

Supplementary Figure 2. ^{13}**C NMR spectrum of a isomer mixture of 4ar in** $CDCl_3$

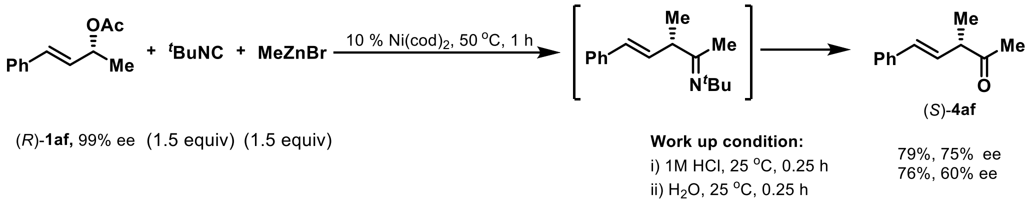

An oven-dried Schlenk tube charged with Ni(cod)$_2$ (5.6 mg, 0.02 mmol, 10 mol%) (weighed in the glove box), DMA (2 mL, 0.1 M) was added into the reaction mixture. To this solution was subsequently added allylic acetate (*R*)-**1af** (38 μL, 0.2 mmol, 1.0 equiv), ^{t}BuNC (34 μL, 0.3 mmol, 1.5 equiv) and MeZnBr (0.91 mL, 0.3 mmol, 1.5 equiv). The tube was equipped with a balloon filled with N_2 at 50 ºC for 1 h.

work up conditions:

i) The mixture was added 1 M HCl aq. (2 mL) and stirred at room temperature for 0.25 h. The mixture was then extracted three times with EtOAc and separated organic layer was washed with brine, dried over anhydrous Na_2SO_4, and concentrated under reduced pressure to yield the crude product, which was purified by silica gel flash column chromatography (PE:EA = 200:1–150:1 v/v) to afford (*S*)-**4af** as a colorless oil (27.4 mg, 79%). R_f = 0.41 (PE:EtOAc = 20:1 v/v). HPLC analysis (Daicel Chiralpak, OJ-H, n-hexane:i-propanol = 99:1 v/v, 1.0 mL:min, 254 nm, retention time: 16.5 min (major), 18.8 min (minor) indicated 75% ee. $[\alpha]_D^{20}$ = + 163.6 (c=1, $CHCl_3$), [lit.[23]: $[\alpha]_D^{20}$ = + 76.3 (c=1, $CHCl_3$).

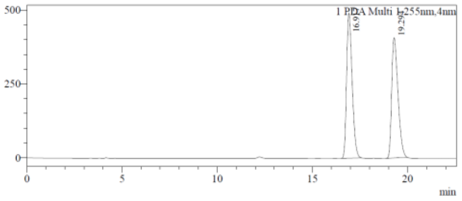

Peak Table

PDA Ch1 255nm

Peak#	Ret. Time	Peak End	Height	Area	Area%
1	16.917	17.579	490280	9887866	51.473
2	19.294	19.989	406592	9321895	48.527
Total			896872	19209761	100.000

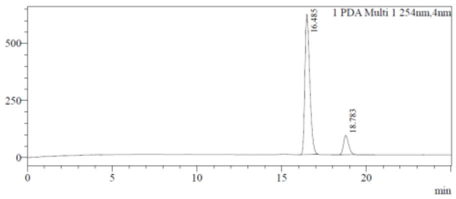

Peak Table

PDA Ch1 254nm

Peak#	Ret. Time	Peak End	Height	Area	Area%
1	16.485	17.163	614211	12266587	87.344
2	18.783	19.339	84562	1777435	12.656
Total			698772	14044023	100.000

Supplementary Figure 3. HPLC chromatogram for compound (*S*)-4af

ii) The mixture was added H_2O (2 mL) and stirred at room temperature for 0.25 h. The mixture was then extracted three times with EtOAc and separated organic layer was washed with brine, dried over anhydrous Na_2SO_4, and concentrated under reduced pressure to yield the crude product, which was purified by silica gel flash column chromatography (PE:EA = 200:1–150:1 v/v) to afford (*S*)-**4af** as a colorless oil (26.3 mg, 76%). R_f = 0.41 (PE:EtOAc = 20:1 v/v). HPLC analysis (Daicel Chiralpak, OJ-H, n-hexane :i-propanol = 99:1(v/v), 1.0 mL:min, 254 nm, retention time: 16.2 min (major), 18.4 min (minor) indicated 60% ee.

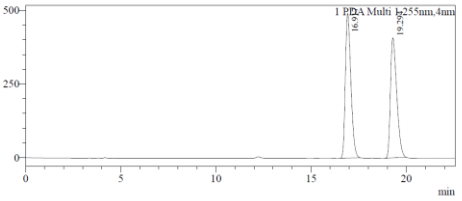

Peak Table

PDA Ch1 255nm

Peak#	Ret. Time	Peak End	Height	Area	Area%
1	16.917	17.579	490280	9887866	51.473
2	19.294	19.989	406592	9321895	48.527
Total			896872	19209761	100.000

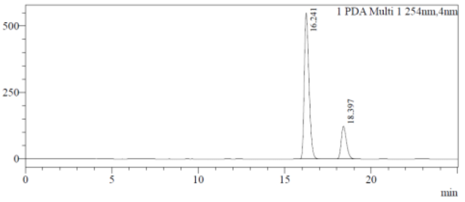

Peak Table

PDA Ch1 254nm

Peak#	Ret. Time	Peak End	Height	Area	Area%
1	16.241	17.163	548246	10369142	79.928
2	18.397	19.381	123383	2603894	20.072
Total			671629	12973036	100.000

Supplementary Figure 4. HPLC chromatogram for compound (*S*)-4af

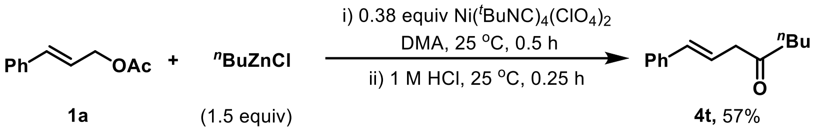

An oven-dried Schlenk tube charged with Ni(^{t}BuNC)$_4$(ClO$_4$)$_2$[24] (44.1 mg, 0.075 mmol, 0.38 equiv) was evacuated and backfilled with N_2 (This process was repeated for three times). DMA (2 mL, 0.1 M) was added into the reaction mixture. To this solution was subsequently added allylic acetate **1a** (34 µL, 0.2 mmol, 1.0 equiv) and ^{n}BuZnCl (0.97 mL, 0.3 mmol, 1.5 equiv). The tube was equipped with a balloon filled with N_2 at 25 °C for 0.5 h. The mixture was added 1 M HCl aq. (2 mL) and stirred at room temperature for 0.25 h. The mixture was then added EtOAc and afforded **4t** with corrected 57% GC yield.

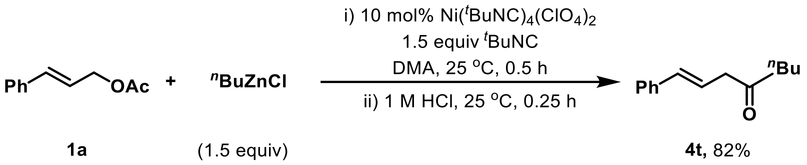

An oven-dried Schlenk tube charged with Ni(^{t}BuNC)$_4$(ClO$_4$)$_2$[24] (11.8 mg, 0.02 mmol, 10 mol%) was evacuated and backfilled with N_2 (This process was repeated for three times). DMA (2 mL, 0.1 M) was added into the reaction mixture. To this solution was subsequently added allylic acetate **1a** (34 µL, 0.2 mmol, 1.0 equiv), ^{t}BuNC (34 µL, 0.3 mmol, 1.5 equiv) and ^{n}BuZnCl (0.97 mL, 0.3 mmol, 1.5 equiv). The tube was equipped with a balloon filled with N_2 at 25 °C for 0.5 h. The mixture was added 1 M HCl aq. (2 mL) and stirred at room temperature for 0.25 h. The mixture was then added EtOAc and afforded **4t** with 82% corrected GC yield.

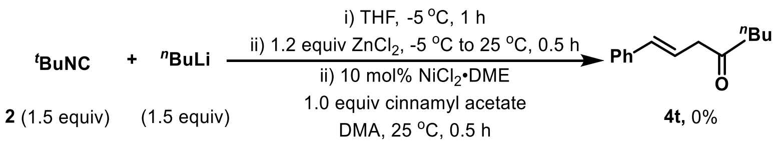

To a -5 °C stirred solution of ^{t}BuNC (68 µL, 0.60 mmol, 1.5 equiv) in THF (1 mL,

0.6 M) under N_2 was added ^{n}BuLi (2.4 M, 0.25 mL, 0.60 mmol, 1.5 equiv). The reaction mixture was stirred at -5 °C for 1 h to give an orange mixture that was added $ZnCl_2$ (1 M, 0.72 mL, 0.72 mmol, 1.2 equiv) at -5 °C and was stirred at room temperature for 0.5 h to give an yellow mixture. The mixture was then transferred to a stirred solution of $NiCl_2$·DME (8.8 mg, 0.04 mmol, 10 mol%) and cinnamyl acetate (68 μL, 0.40 mmol, 1.5 equiv) in DMA (4 mL, 0.1 M) and stirred at 25 °C for 0.5 h. The mixture was added 1 M HCl aq. (4 mL) and stirred at room temperature for 0.25 h and no product **4t** was afforded.

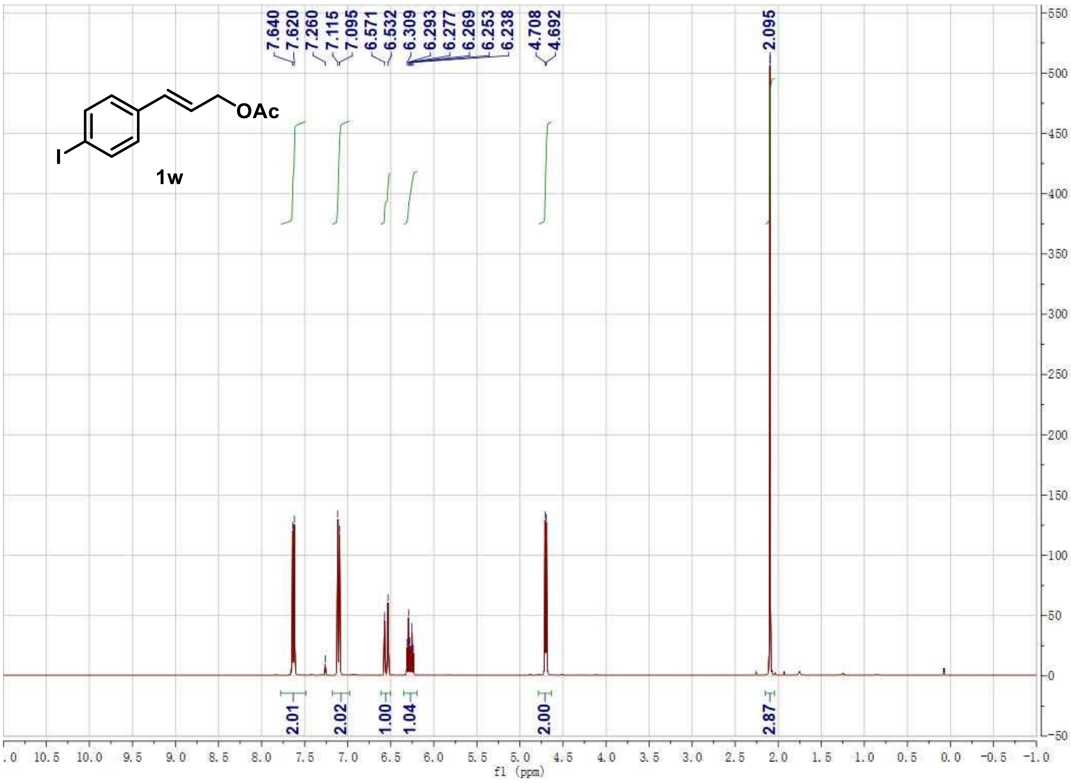

Supplementary Figure 5. ^{1}H NMR (400 MHz, $CDCl_3$) spectrum of 1w

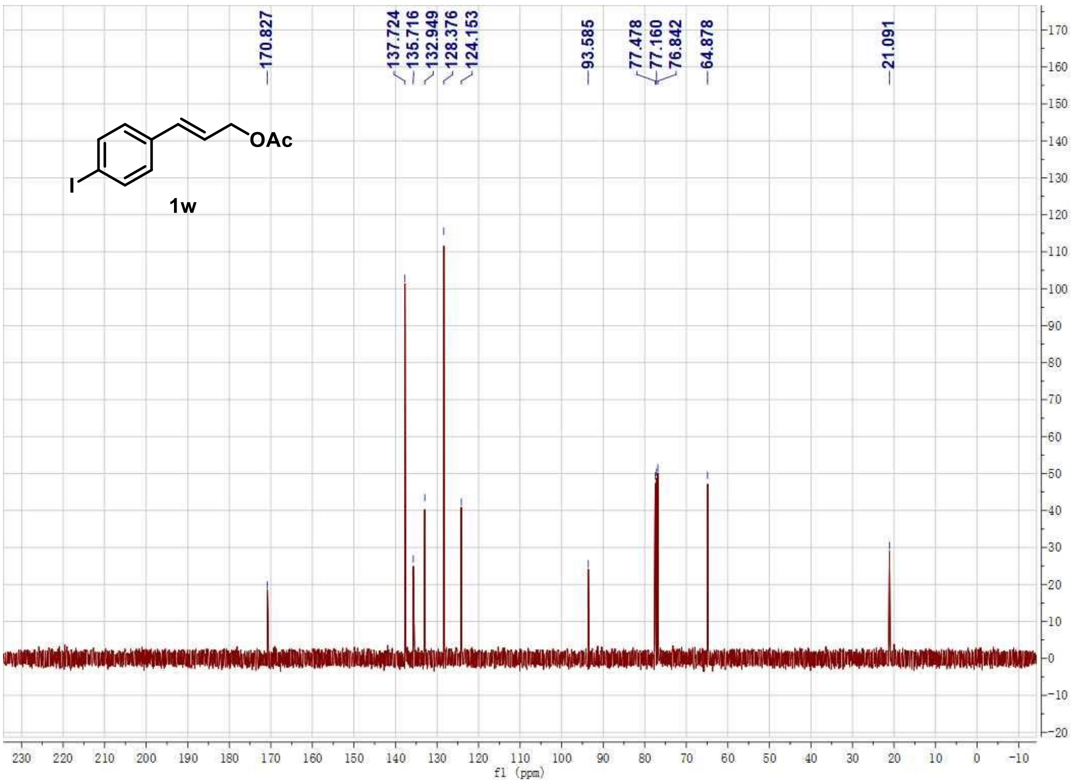

Supplementary Figure 6. ^{13}C NMR (100 MHz, $CDCl_3$) spectrum of 1w

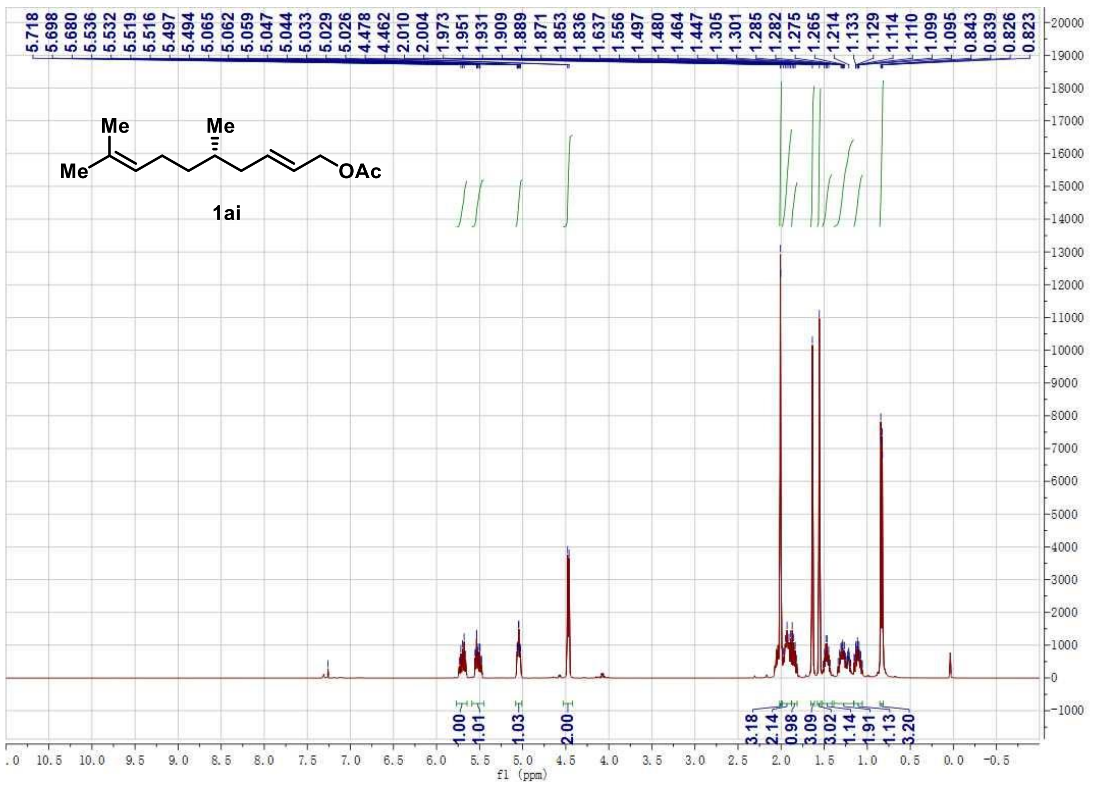

Supplementary Figure 7. ^{1}H NMR (400 MHz, CDCl$_3$) spectrum of 1ai

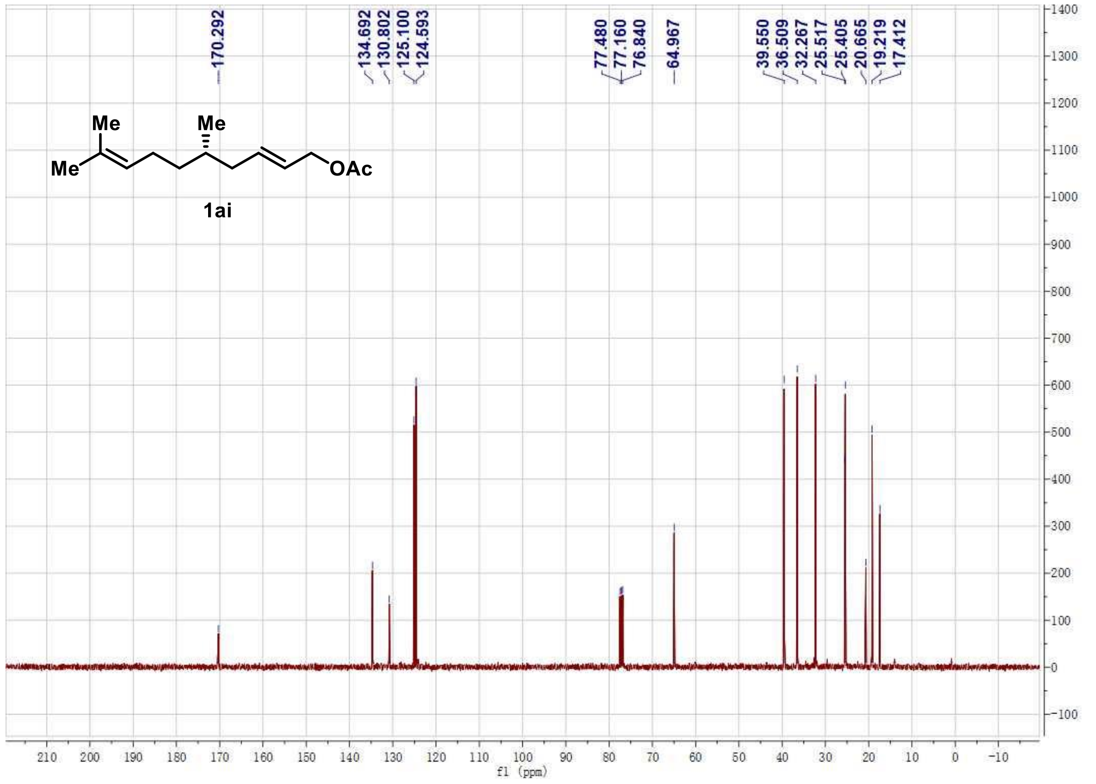

Supplementary Figure 8. ^{13}C NMR (100 MHz, CDCl$_3$) spectrum of 1ai

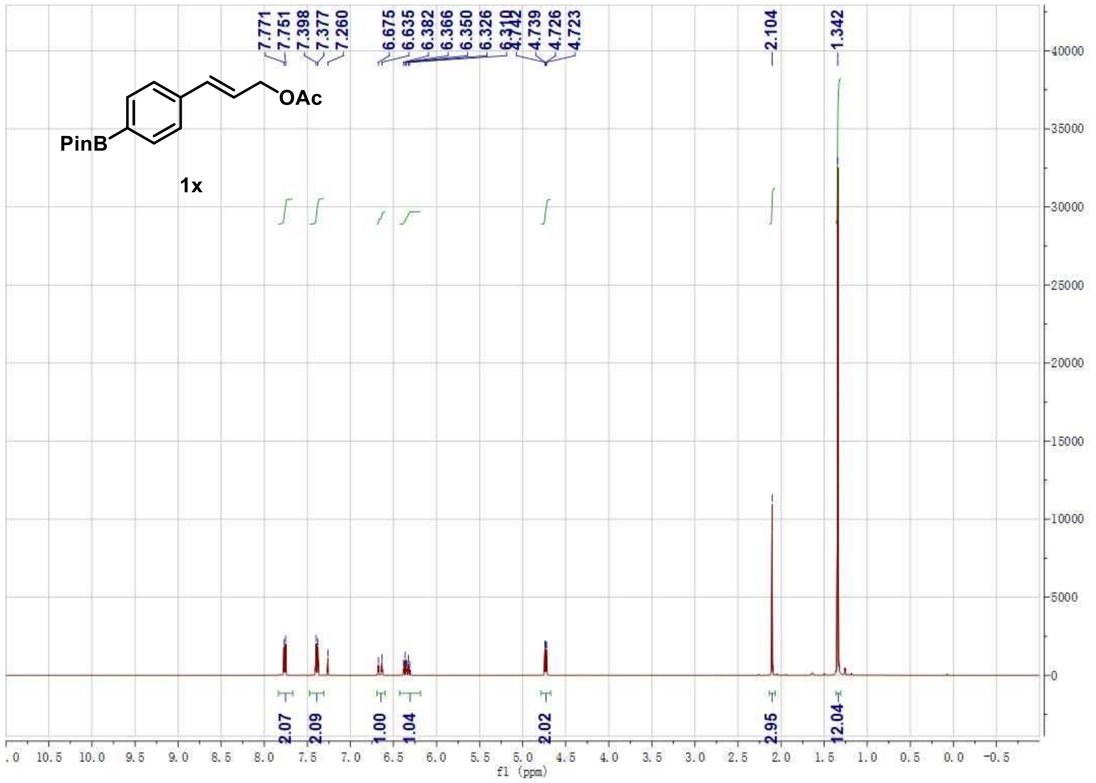

Supplementary Figure 9. **^{1}H NMR (400 MHz, CDCl$_3$) spectrum of 1x**

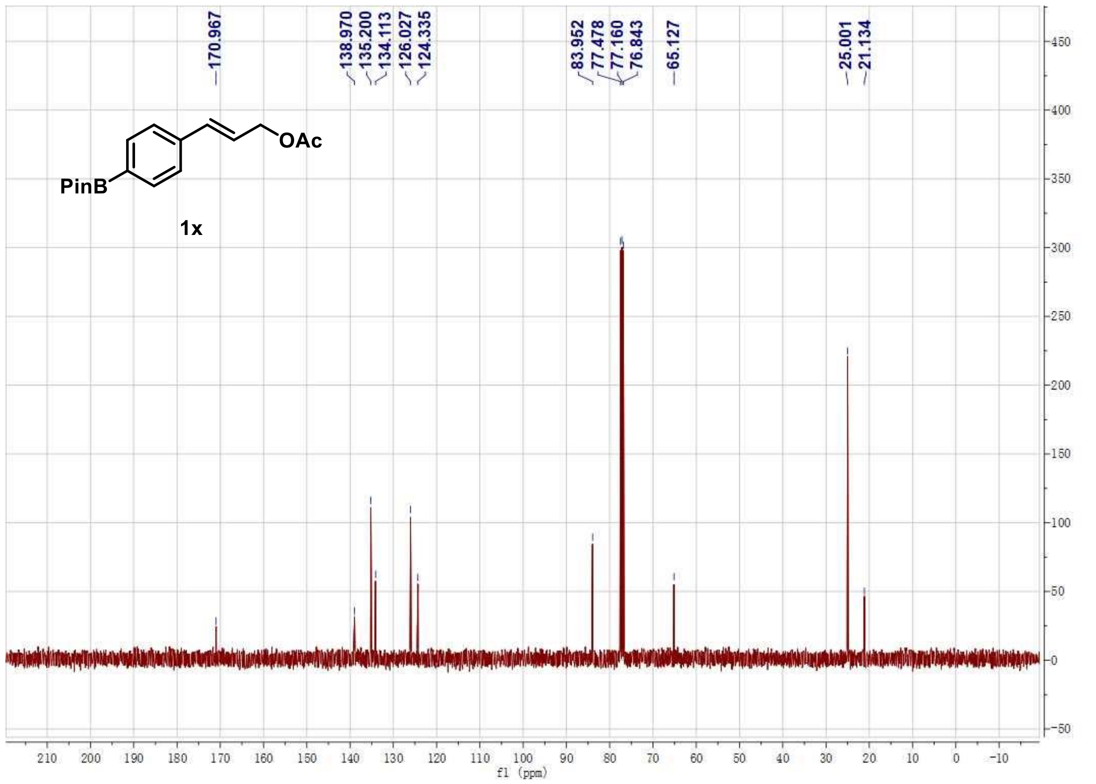

Supplementary Figure 10. **^{13}C NMR (100 MHz, CDCl$_3$) spectrum of 1x**

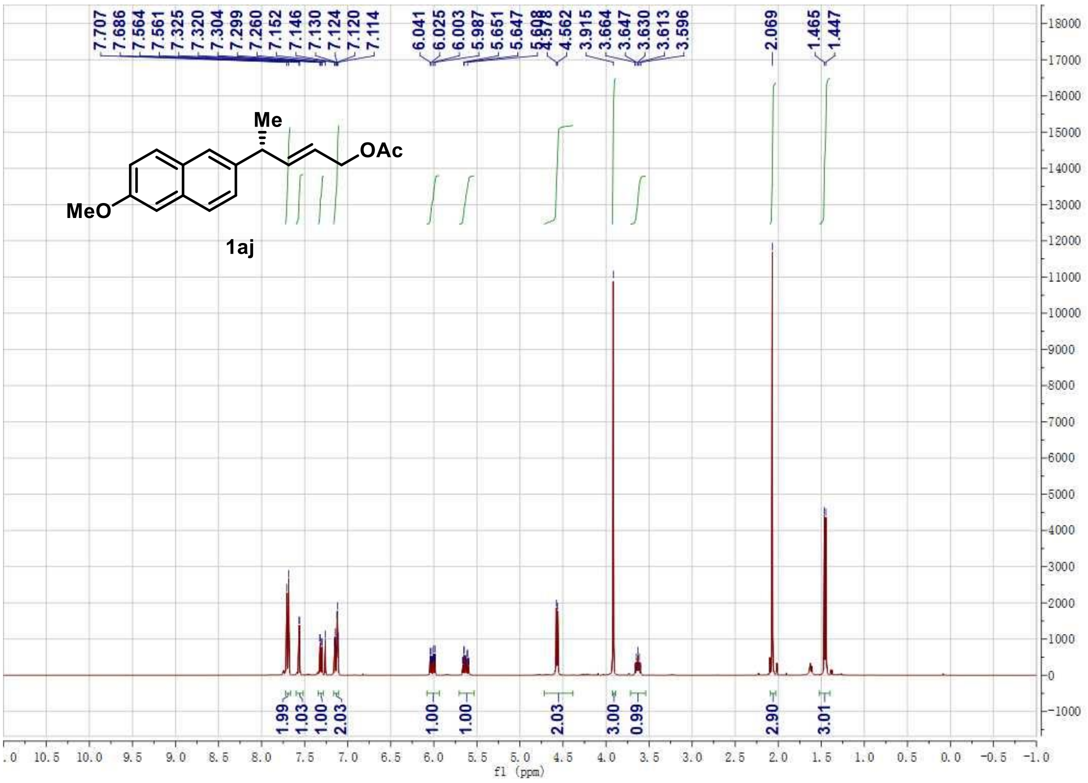

Supplementary Figure 11. ^{1}H NMR (400 MHz, $CDCl_3$) spectrum of 1aj

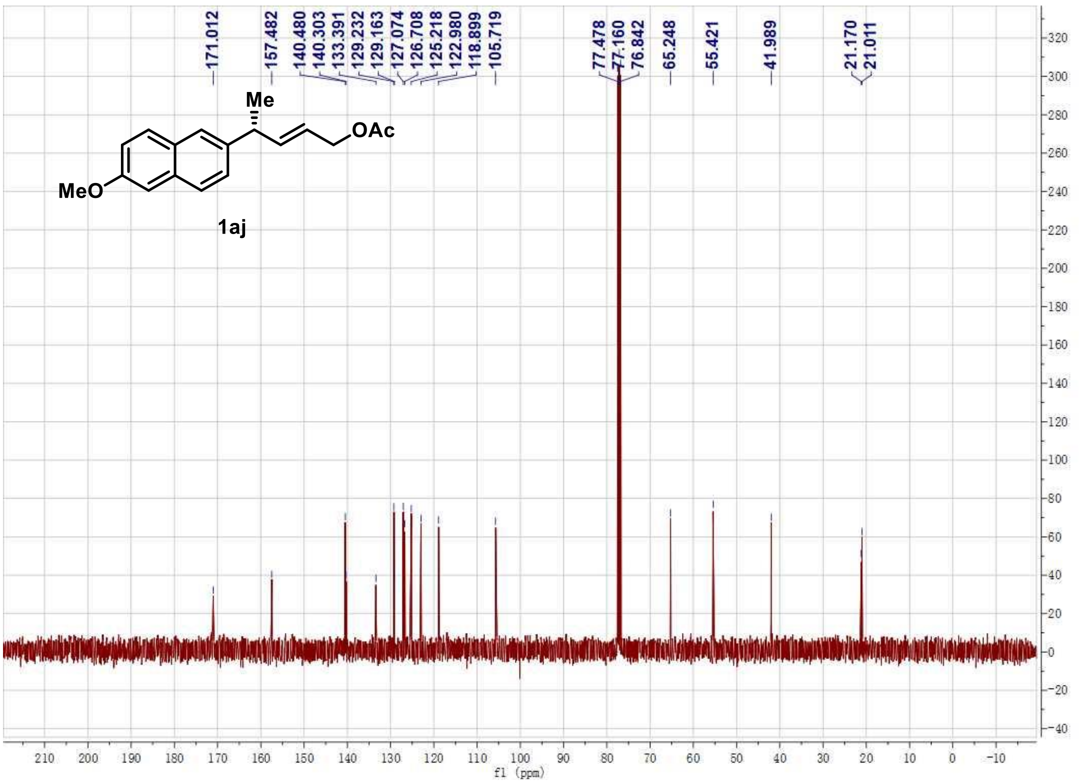

Supplementary Figure 12. ^{13}C NMR (100 MHz, $CDCl_3$) spectrum of 1aj

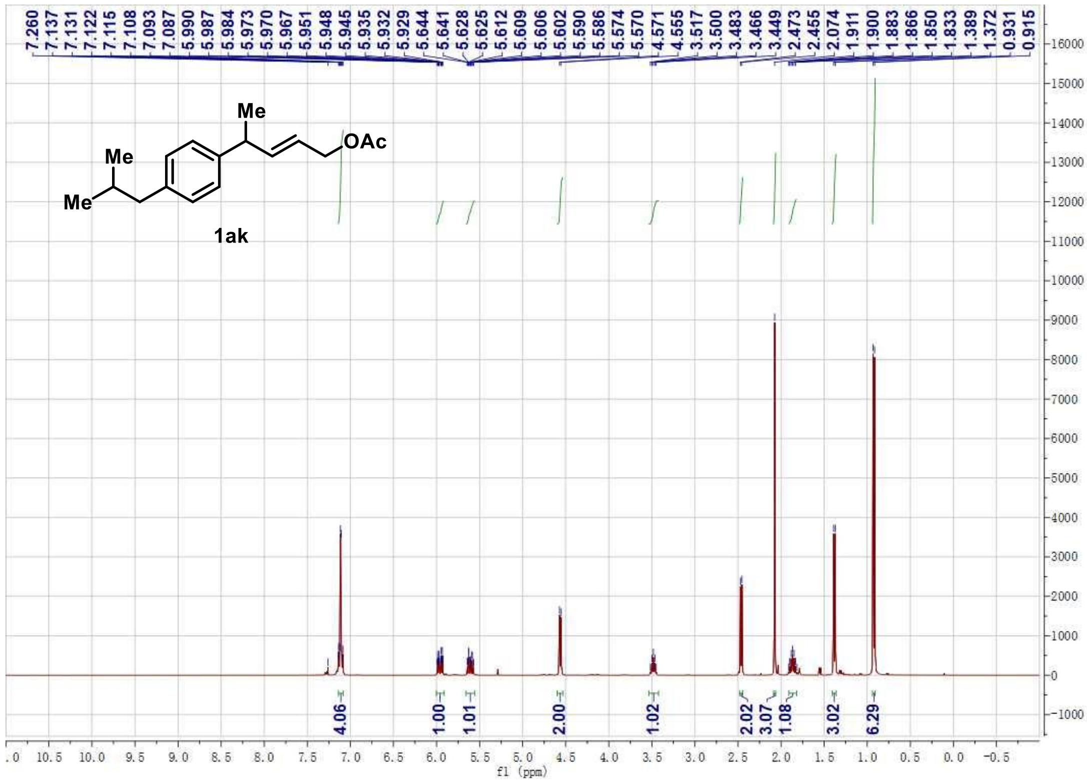

Supplementary Figure 13. ^{1}H NMR (400 MHz, $CDCl_3$) spectrum of 1ak

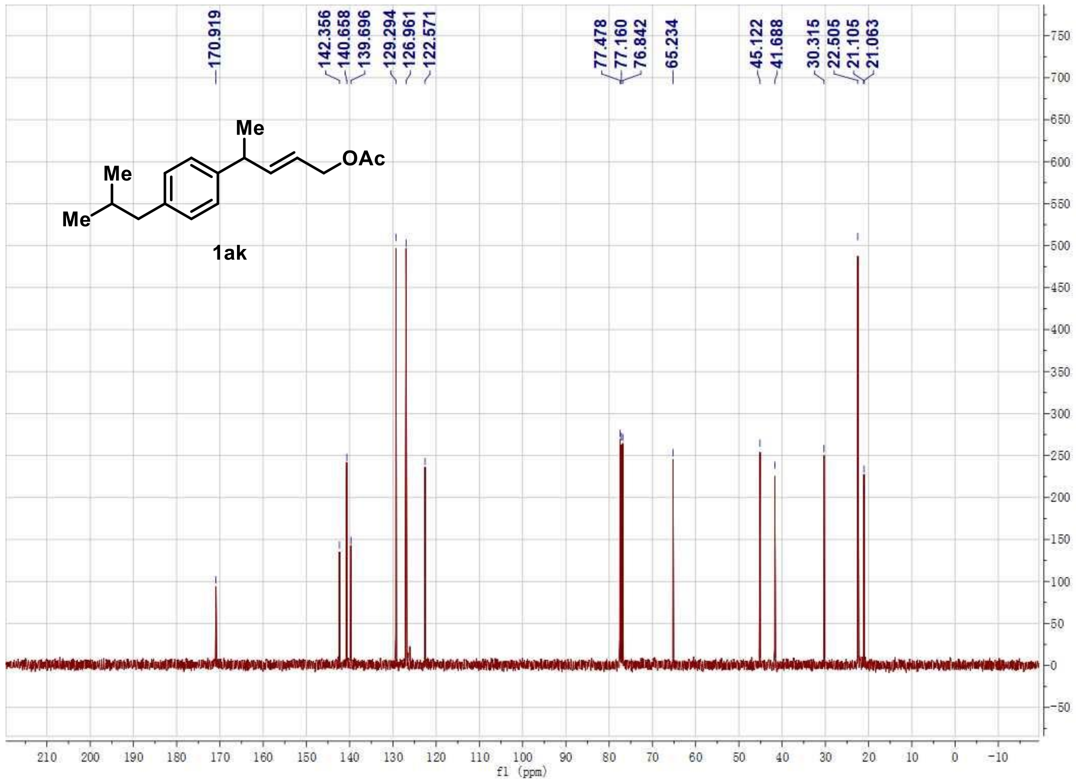

Supplementary Figure 14. ^{13}C NMR (100 MHz, $CDCl_3$) spectrum of 1ak

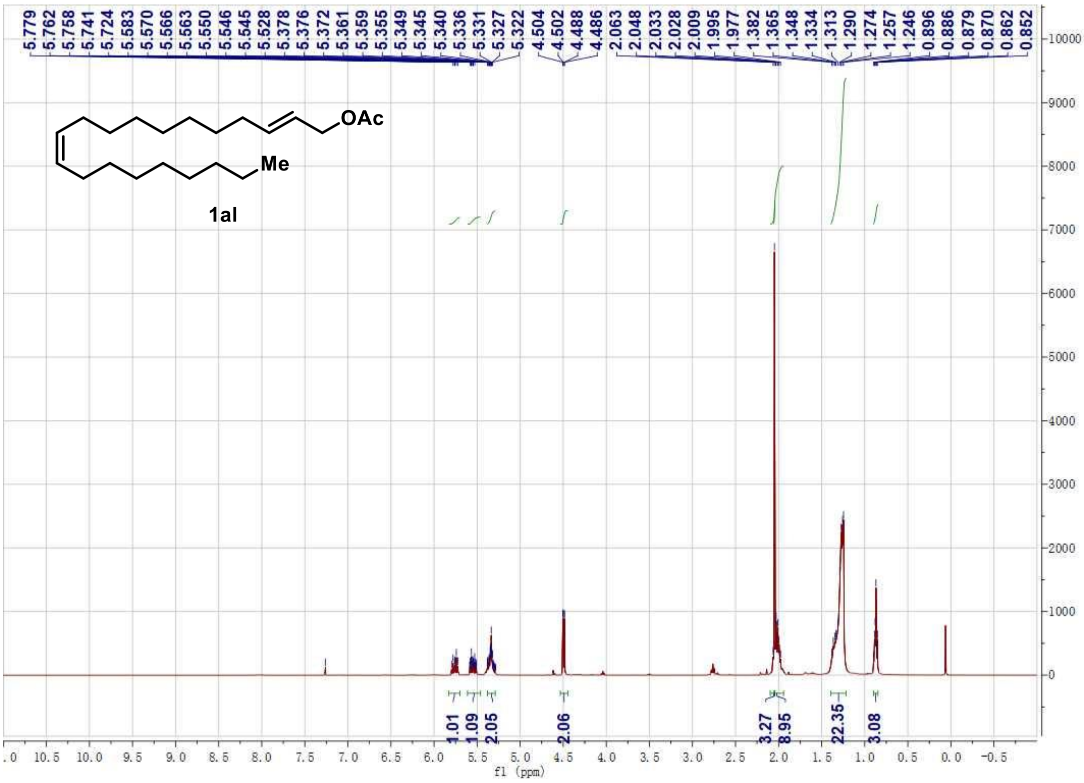

Supplementary Figure 15. ^{1}H NMR (400 MHz, $CDCl_3$) spectrum of 1al

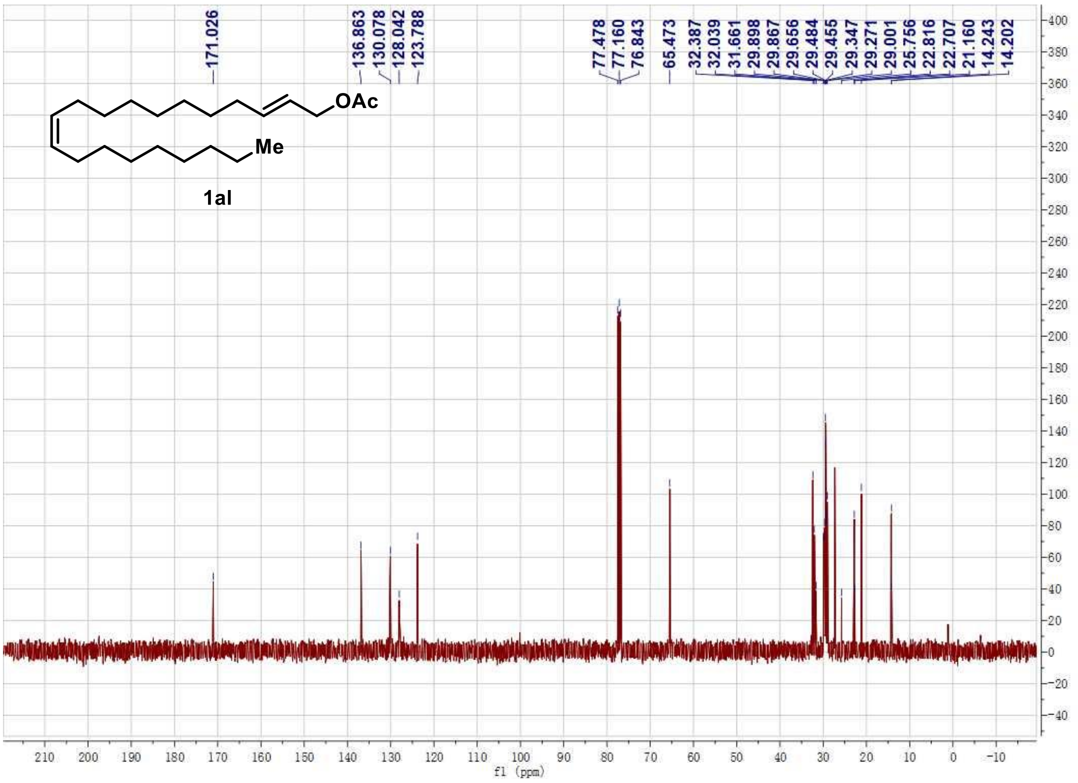

Supplementary Figure 16. ^{13}C NMR (100 MHz, $CDCl_3$) spectrum of 1al

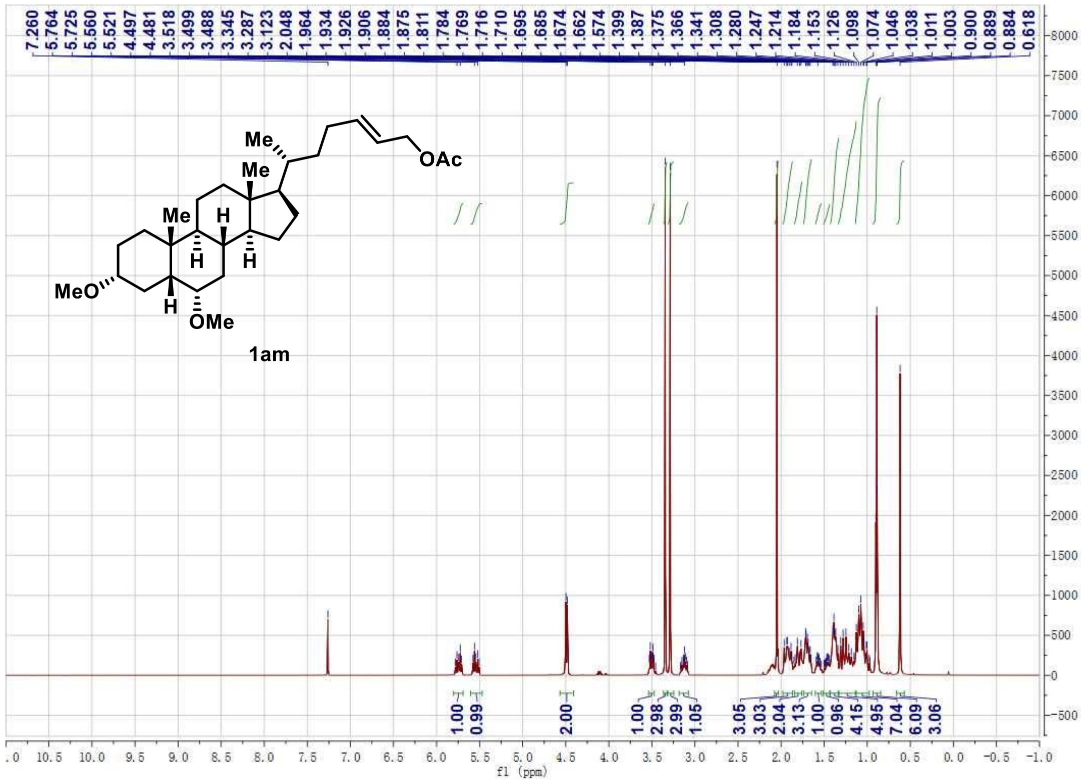

Supplementary Figure 17. ^{1}H NMR (400 MHz, $CDCl_3$) spectrum of 1am

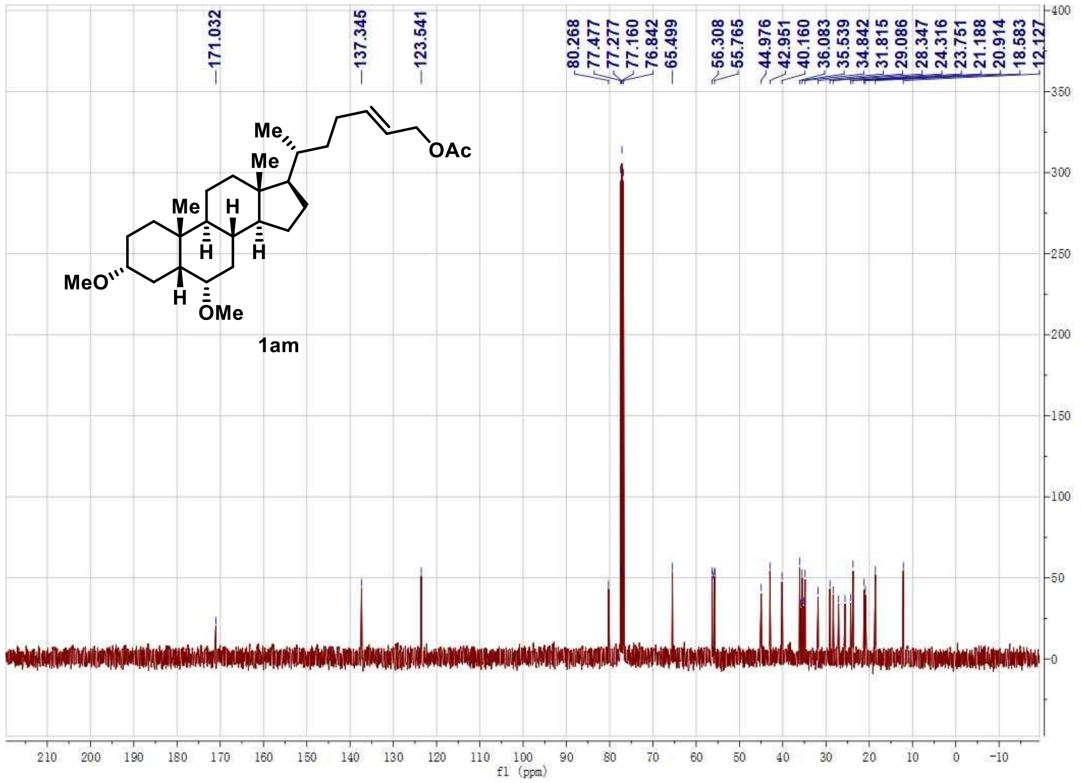

Supplementary Figure 18. ^{13}C NMR (100 MHz, $CDCl_3$) spectrum of 1am

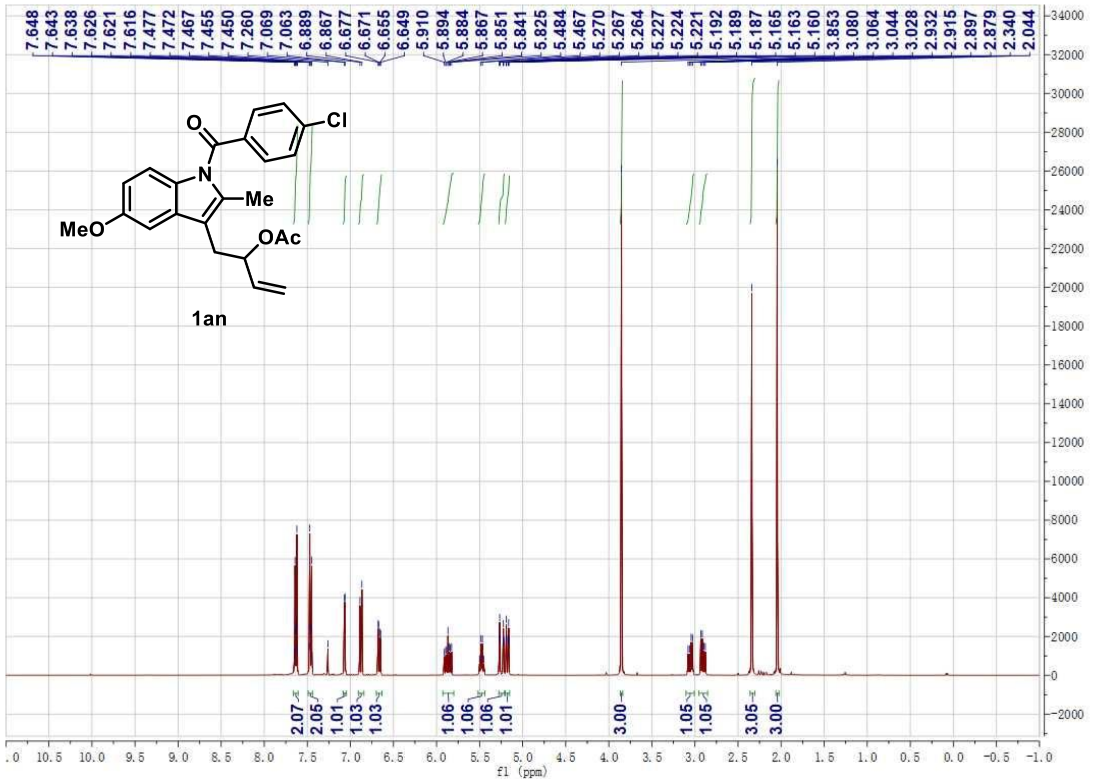

Supplementary Figure 19. ^{1}H NMR (400 MHz, $CDCl_3$) spectrum of 1an

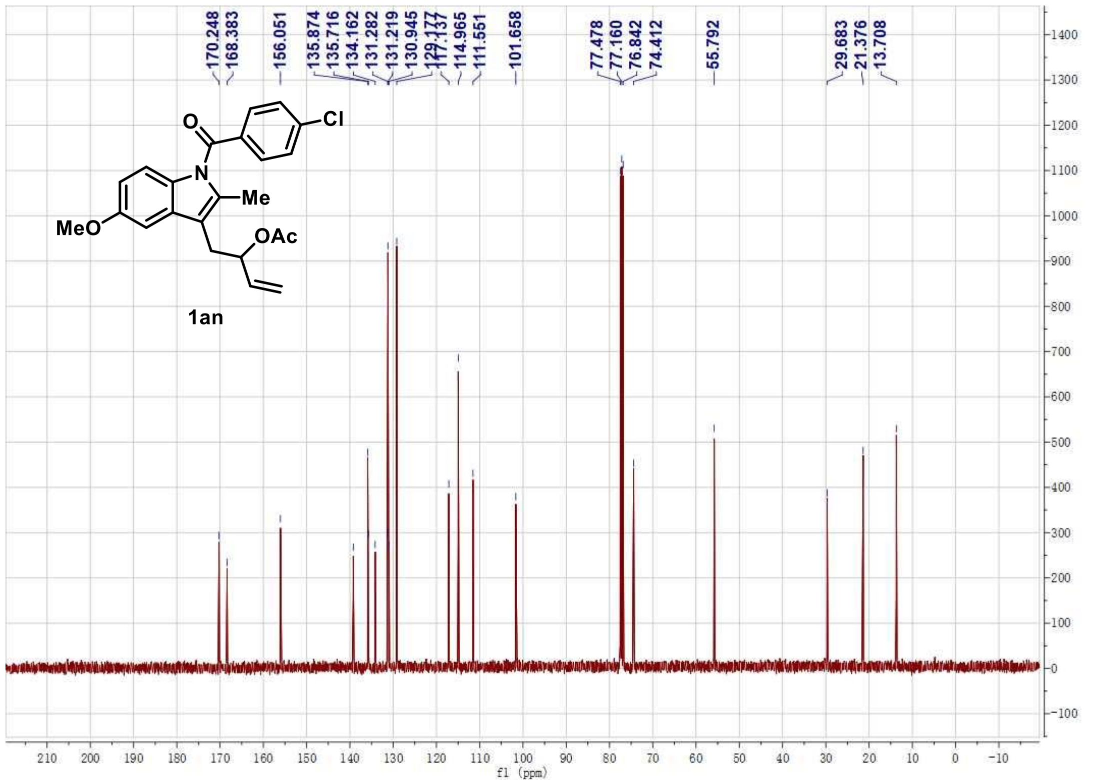

Supplementary Figure 20. ^{13}C NMR (100 MHz, $CDCl_3$) spectrum of 1an

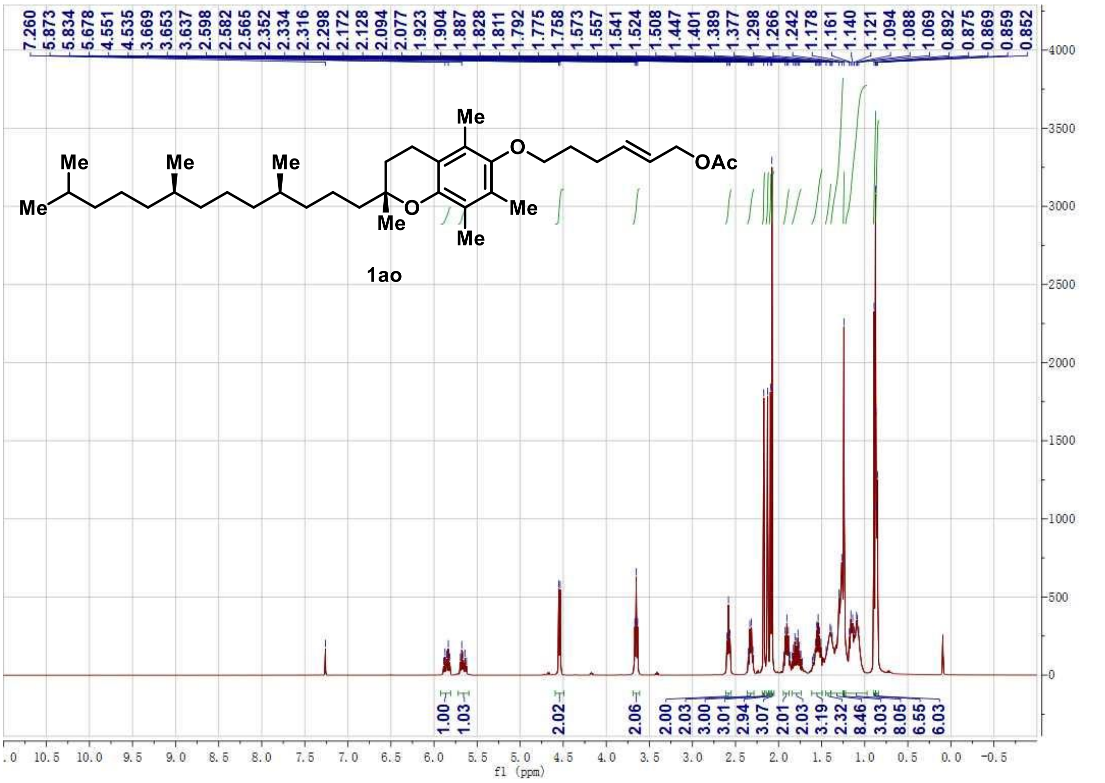

Supplementary Figure 21. ^{1}H NMR (400 MHz, CDCl3) spectrum of 1ao

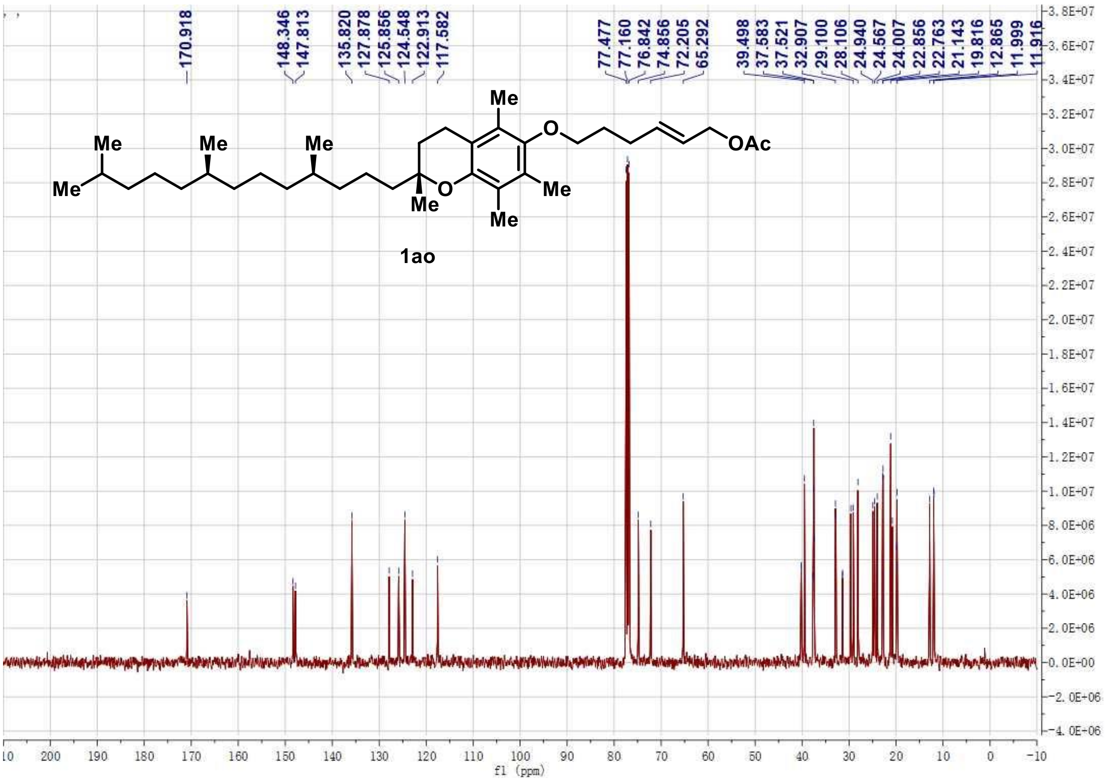

Supplementary Figure 22. ^{13}C NMR (100 MHz, CDCl3) spectrum of 1ao

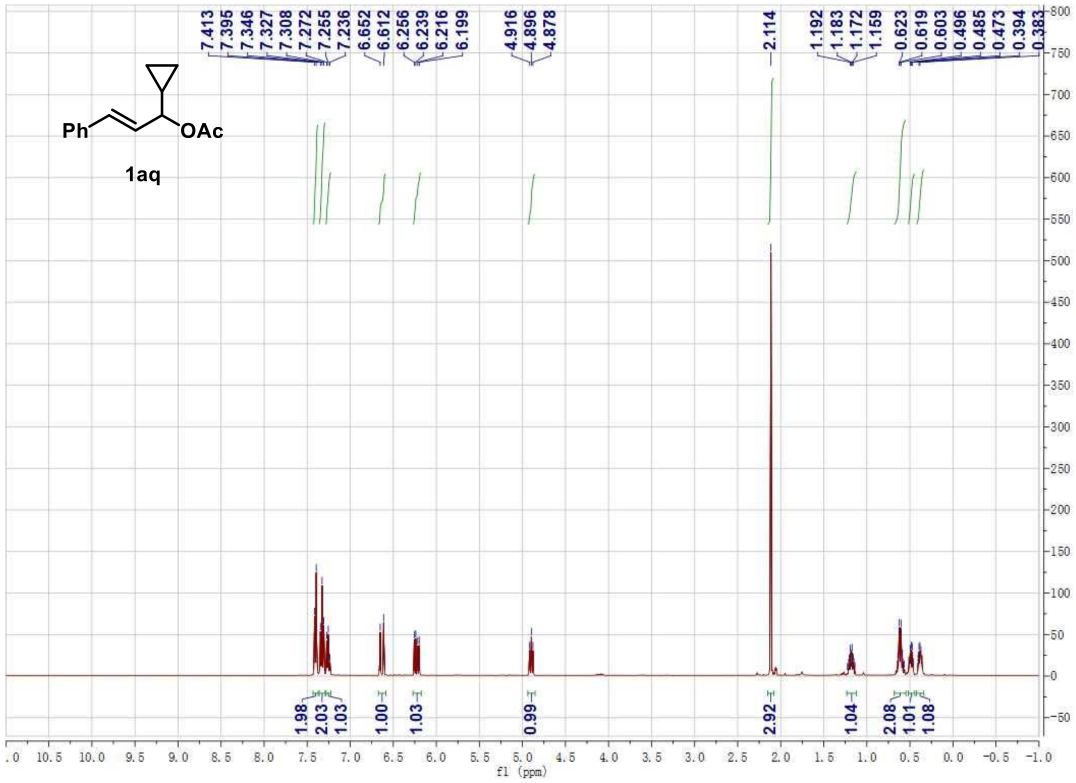

Supplementary Figure 23. ¹H NMR (400 MHz, CDCl₃) spectrum of 1aq

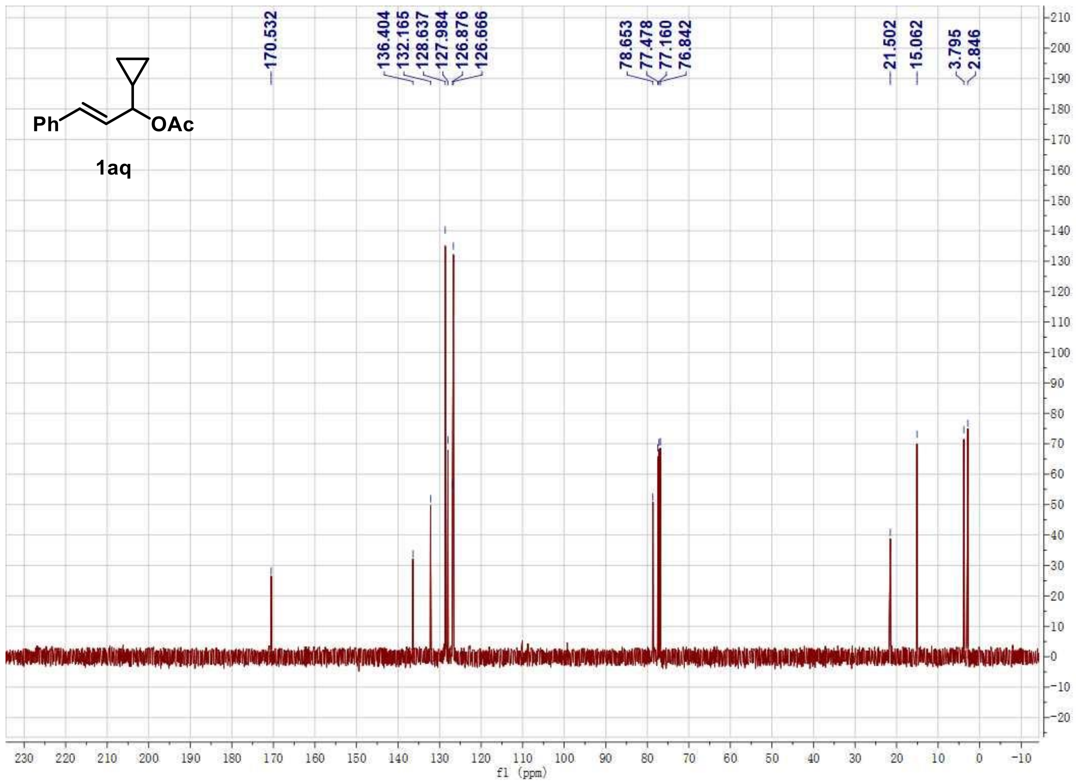

Supplementary Figure 24. ¹³C NMR (100 MHz, CDCl₃) spectrum of 1aq

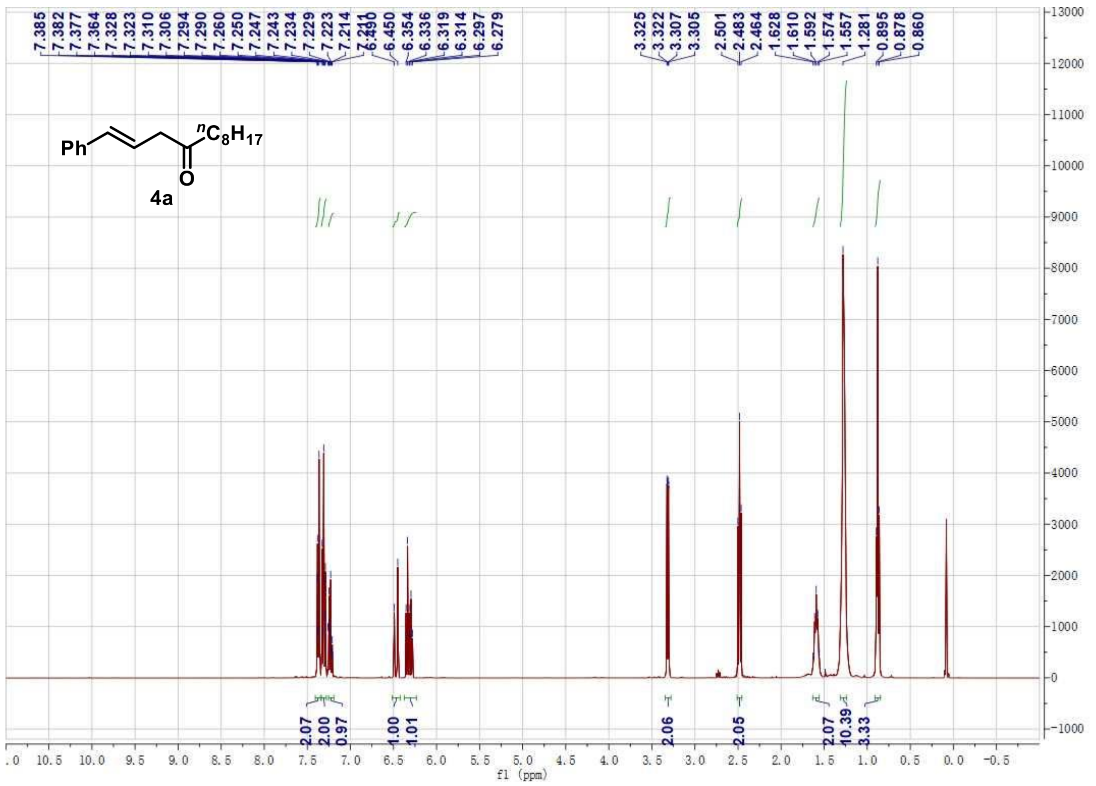

Supplementary Figure 25. ^{1}H NMR (400 MHz, $CDCl_3$) spectrum of 4a

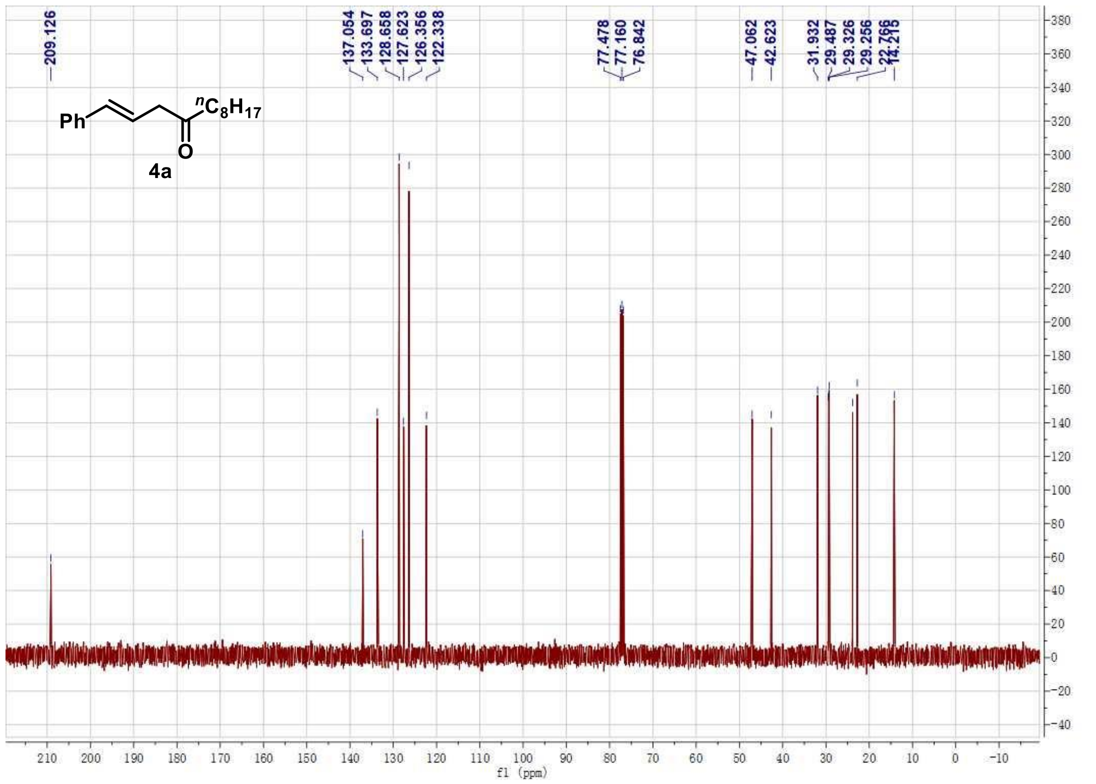

Supplementary Figure 26. ^{13}C NMR (100 MHz, $CDCl_3$) spectrum of 4a

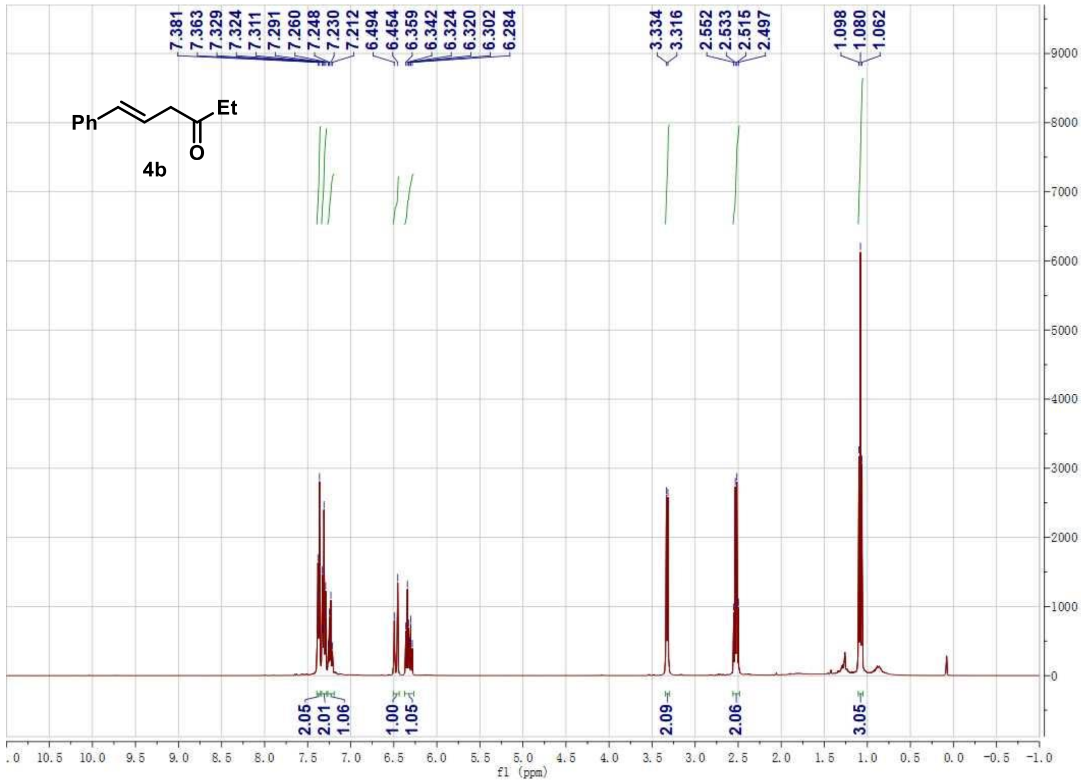

Supplementary Figure 27. ^{1}**H NMR (400 MHz, CDCl$_3$) spectrum of 4b**

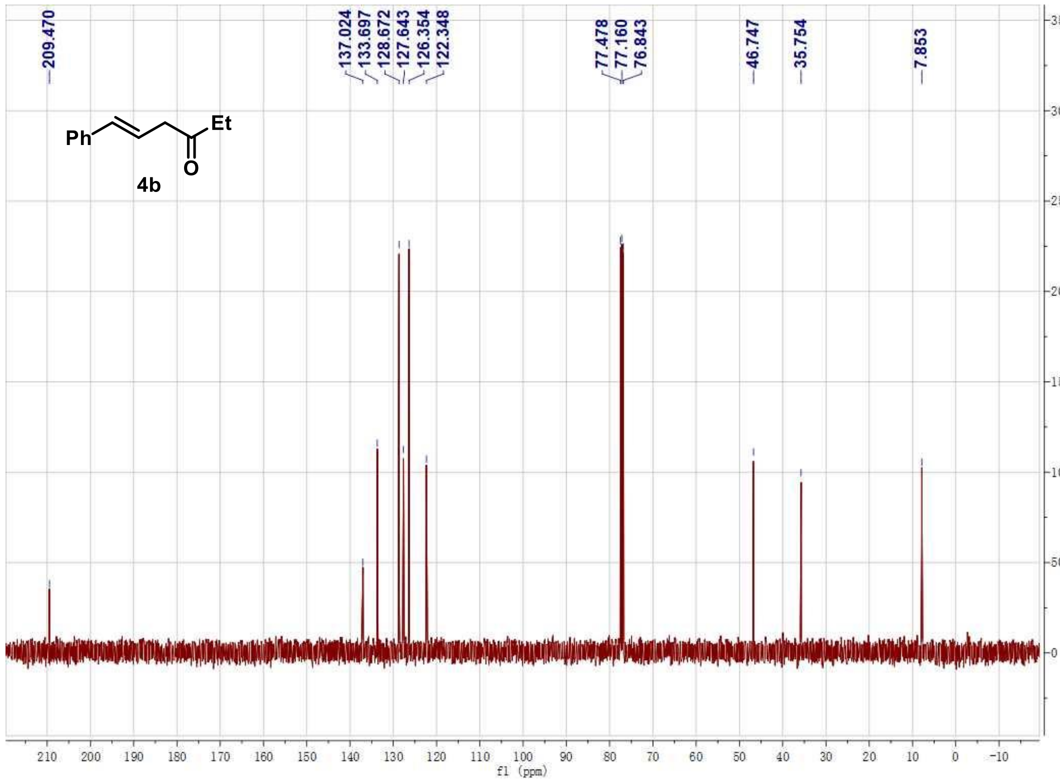

Supplementary Figure 28. ^{13}**C NMR (100 MHz, CDCl$_3$) spectrum of 4b**

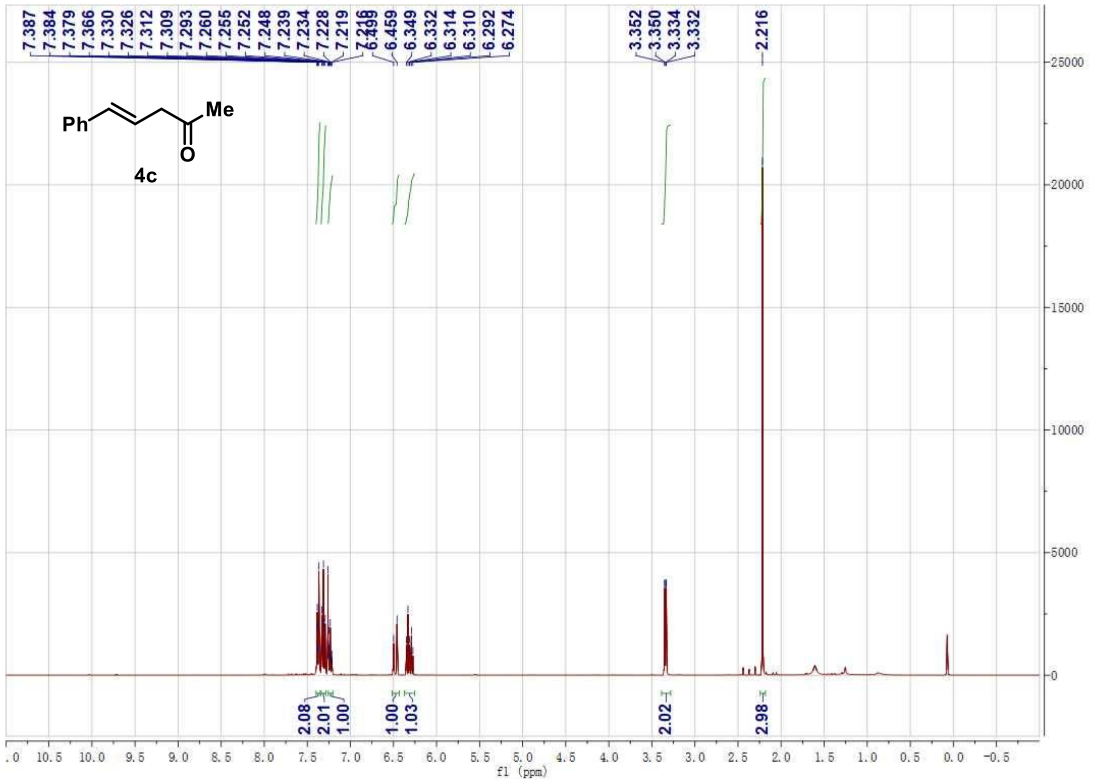

Supplementary Figure 29. ^{1}H NMR (400 MHz, CDCl$_3$) spectrum of 4c

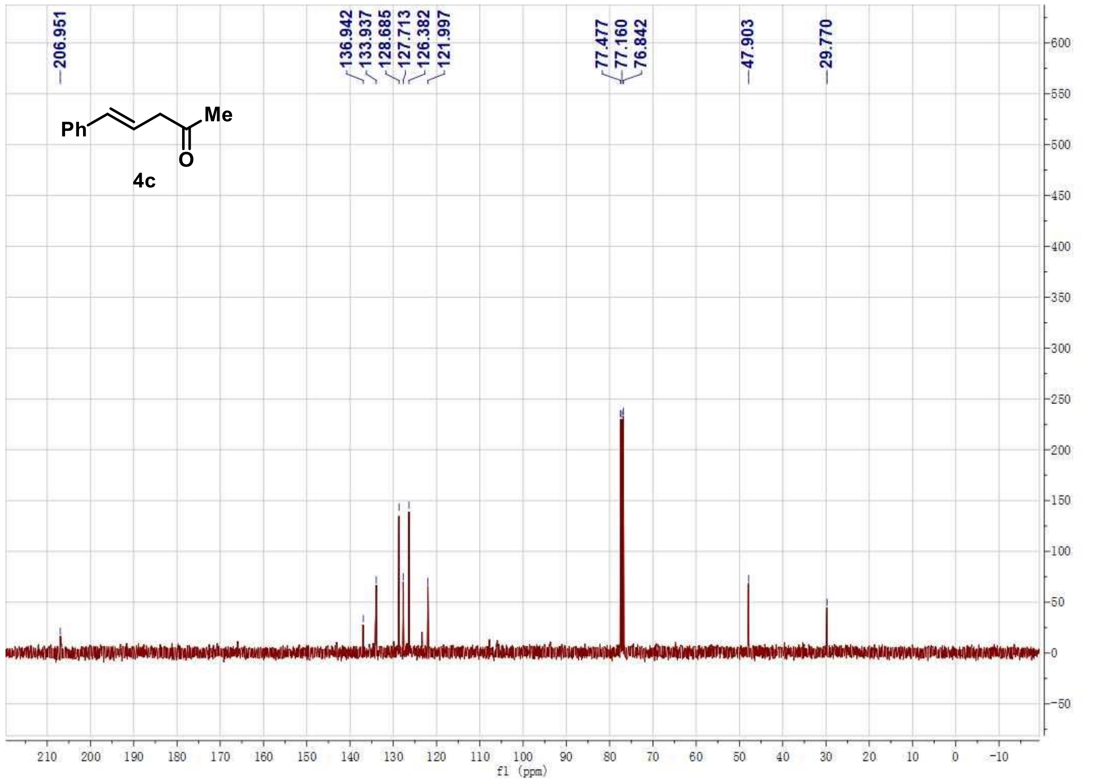

Supplementary Figure 30. ^{13}C NMR (100 MHz, CDCl$_3$) spectrum of 4c

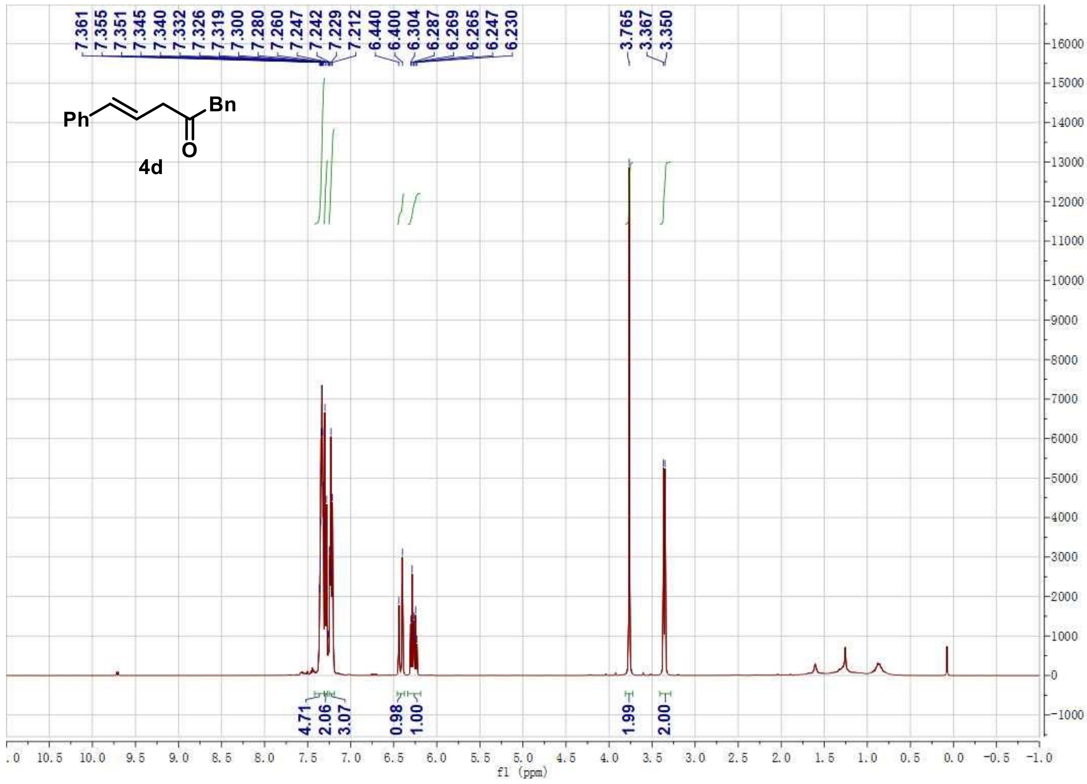

Supplementary Figure 31. ^{1}H NMR (400 MHz, CDCl$_3$) spectrum of 4d

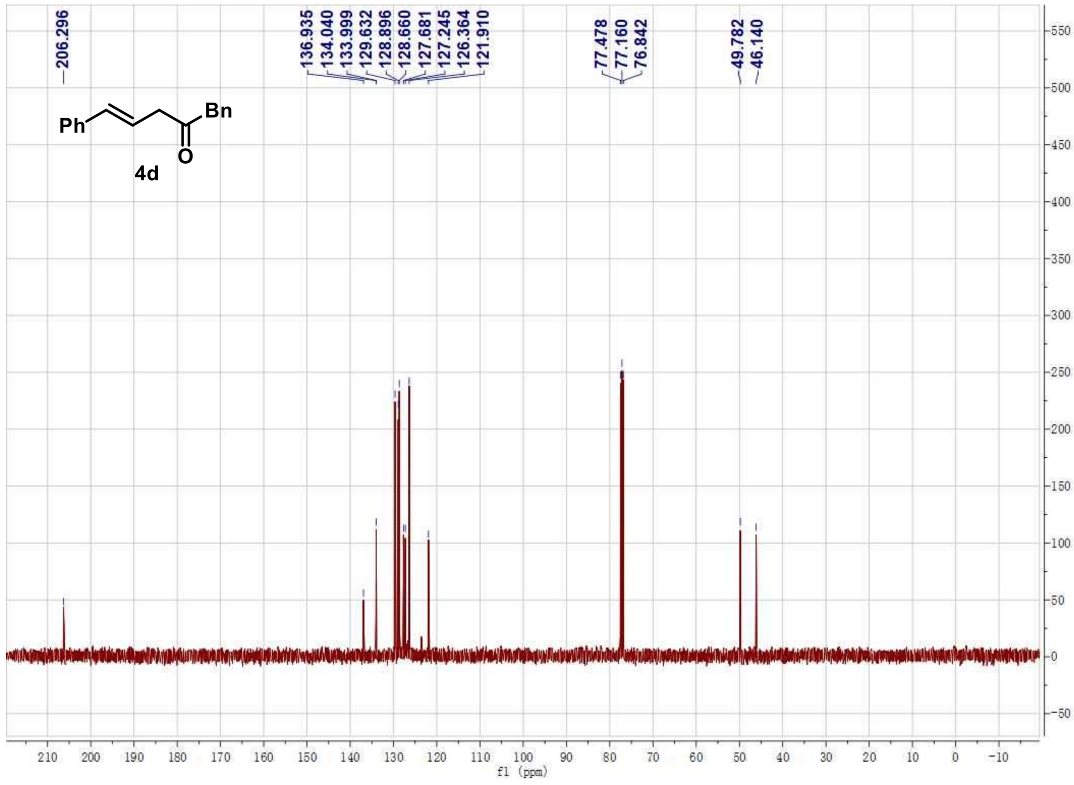

Supplementary Figure 32. ^{13}C NMR (100 MHz, CDCl$_3$) spectrum of 4d

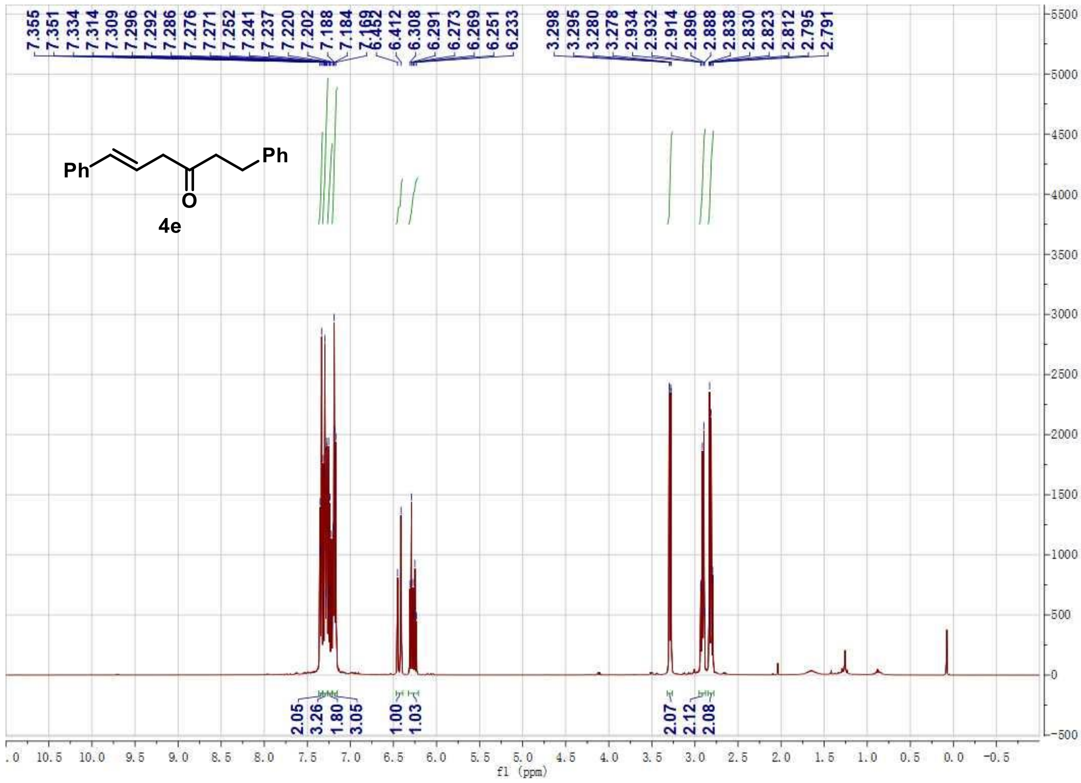

Supplementary Figure 33. ^{1}H NMR (400 MHz, $CDCl_3$) spectrum of 4e

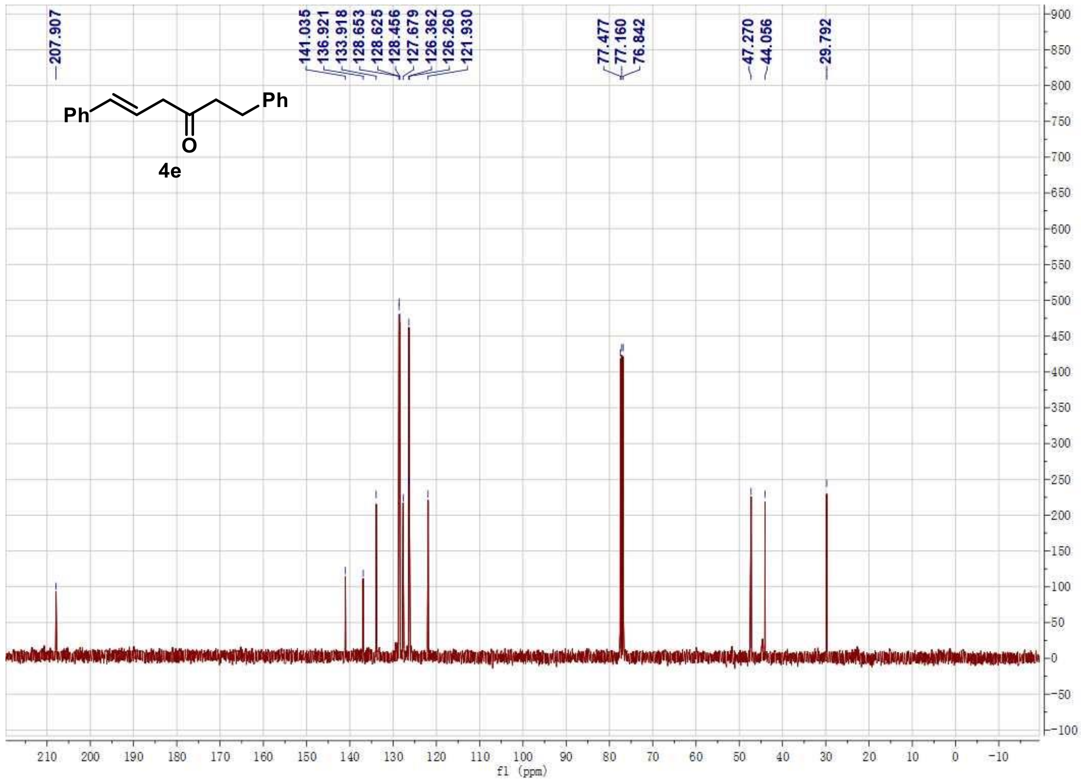

Supplementary Figure 34. ^{13}C NMR (100 MHz, $CDCl_3$) spectrum of 4e

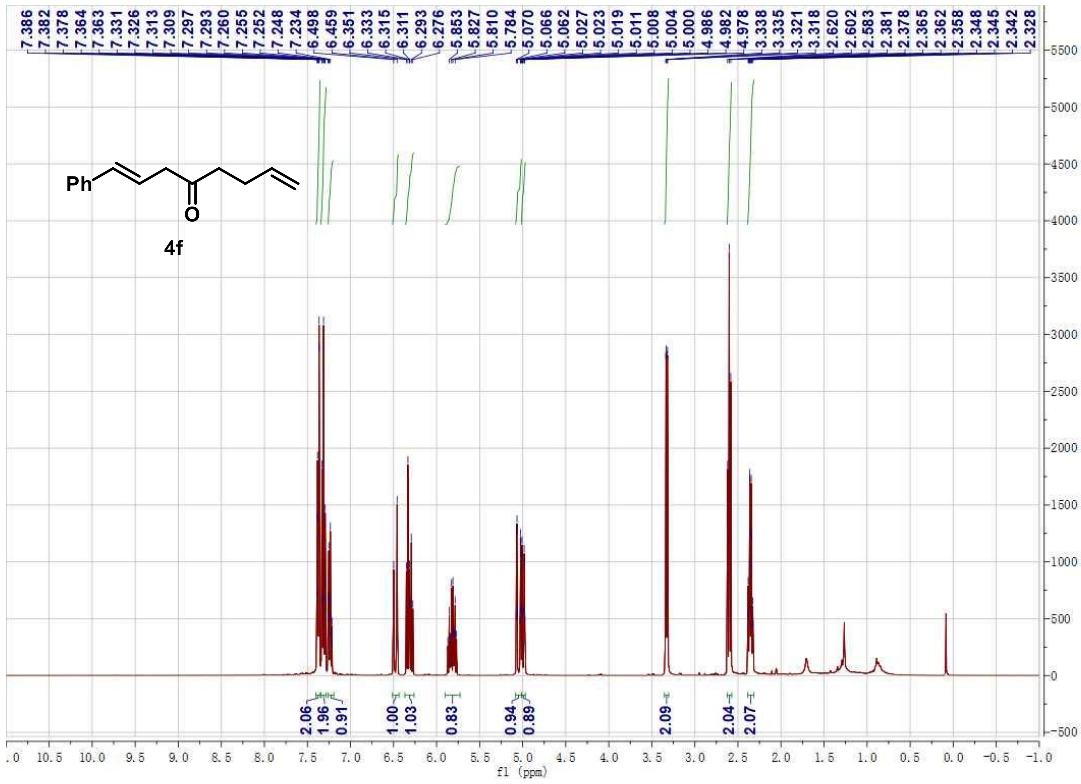

Supplementary Figure 35. ^{1}H NMR (400 MHz, $CDCl_3$) spectrum of 4f

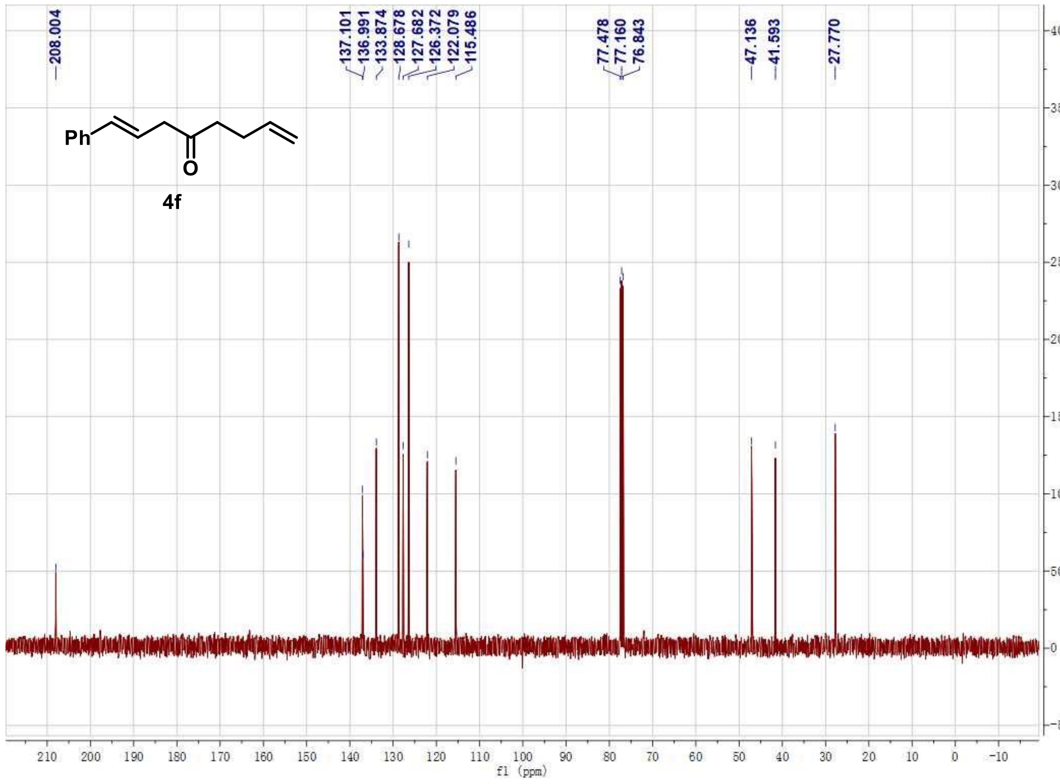

Supplementary Figure 36. ^{13}C NMR (100 MHz, $CDCl_3$) spectrum of 4f

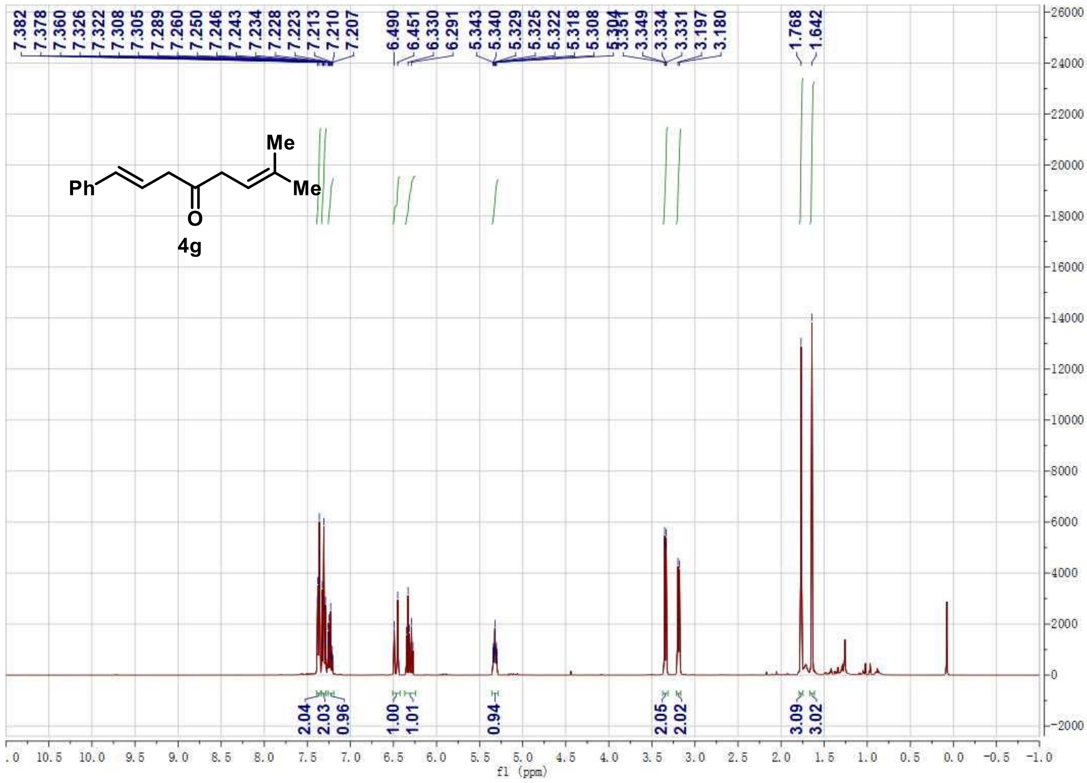

Supplementary Figure 37. ^{1}H NMR (400 MHz, $CDCl_3$) spectrum of 4g

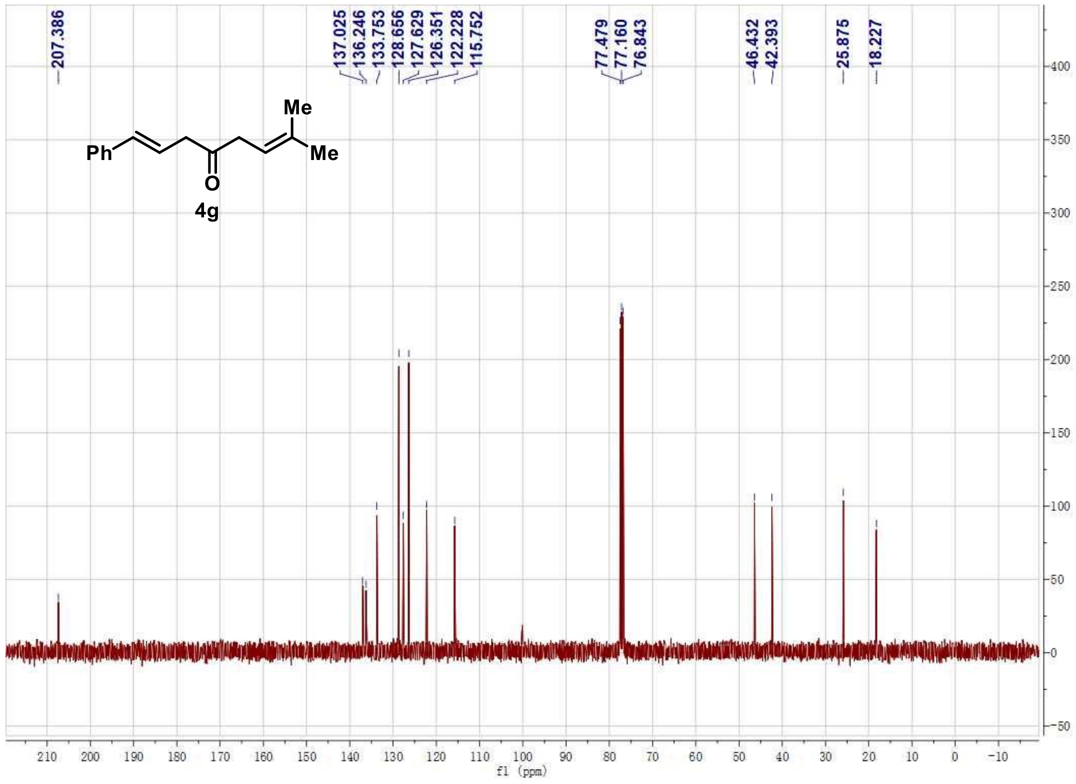

Supplementary Figure 38. ^{13}C NMR (100 MHz, $CDCl_3$) spectrum of 4g

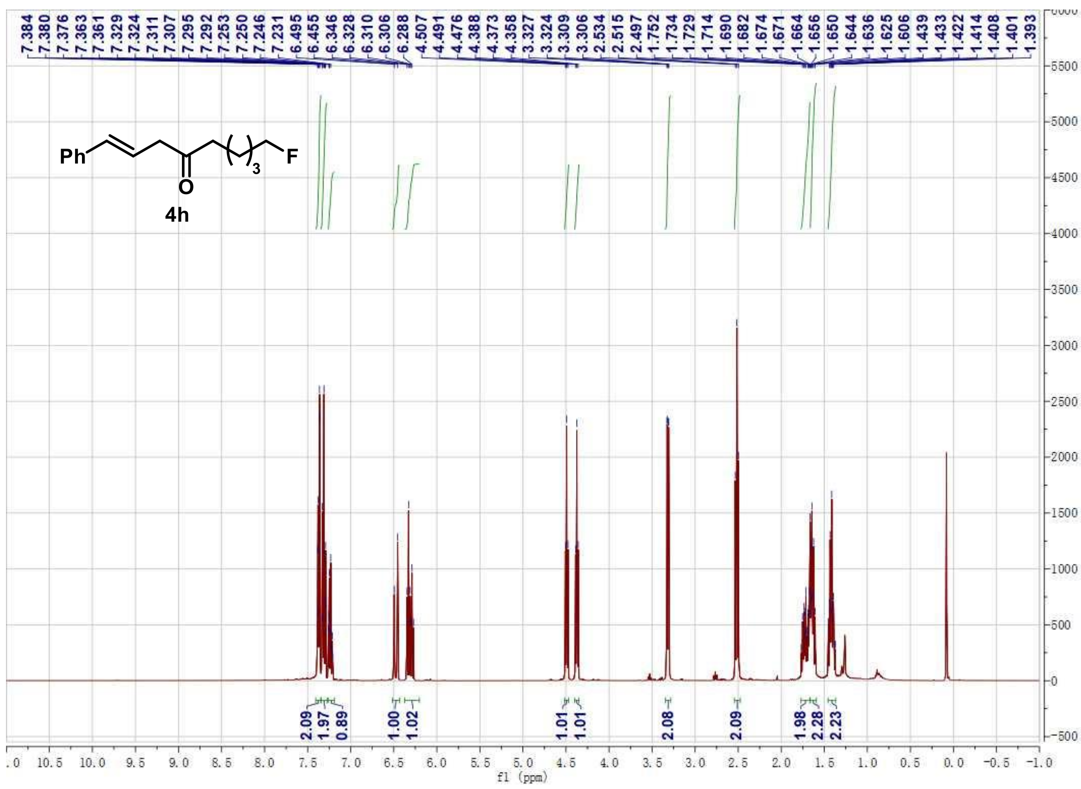

Supplementary Figure 39. ^{1}H NMR (400 MHz, $CDCl_3$) spectrum of 4h

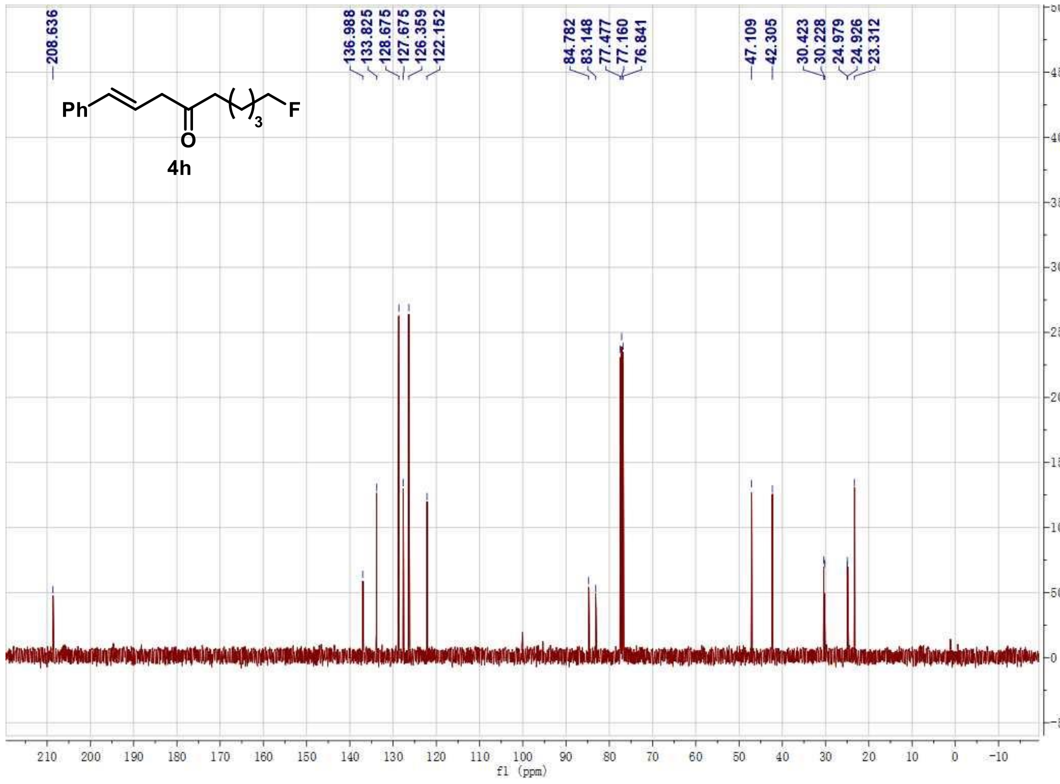

Supplementary Figure 40. ^{13}C NMR (100 MHz, $CDCl_3$) spectrum of 4h

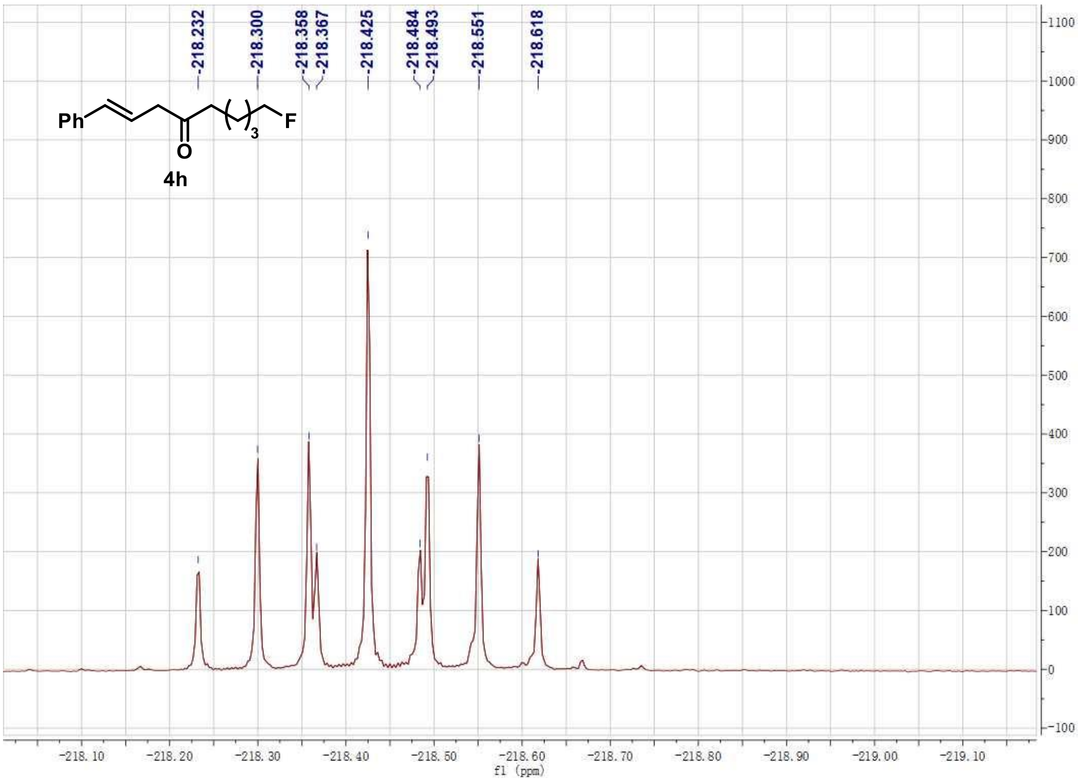

Supplementary Figure 41. ^{19}F NMR (376MHz, CDCl$_3$) spectrum of 4h

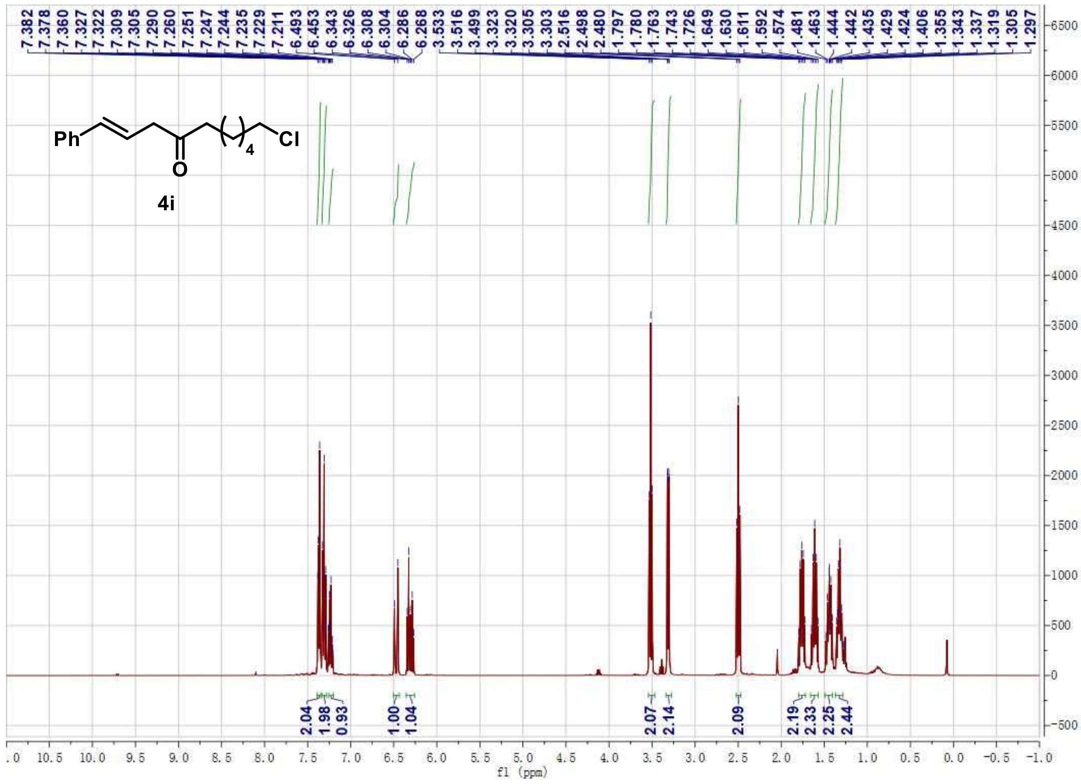

Supplementary Figure 42. ^{1}H NMR (400 MHz, $CDCl_3$) spectrum of 4i

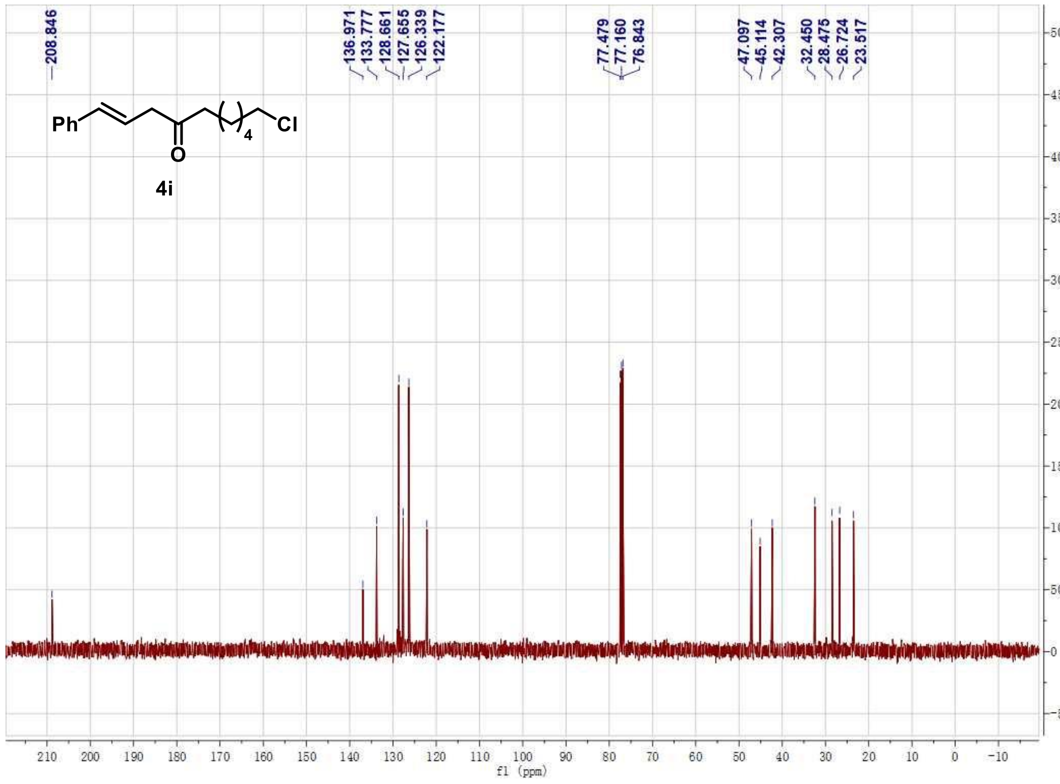

Supplementary Figure 43. ^{13}C NMR (100 MHz, $CDCl_3$) spectrum of 4i

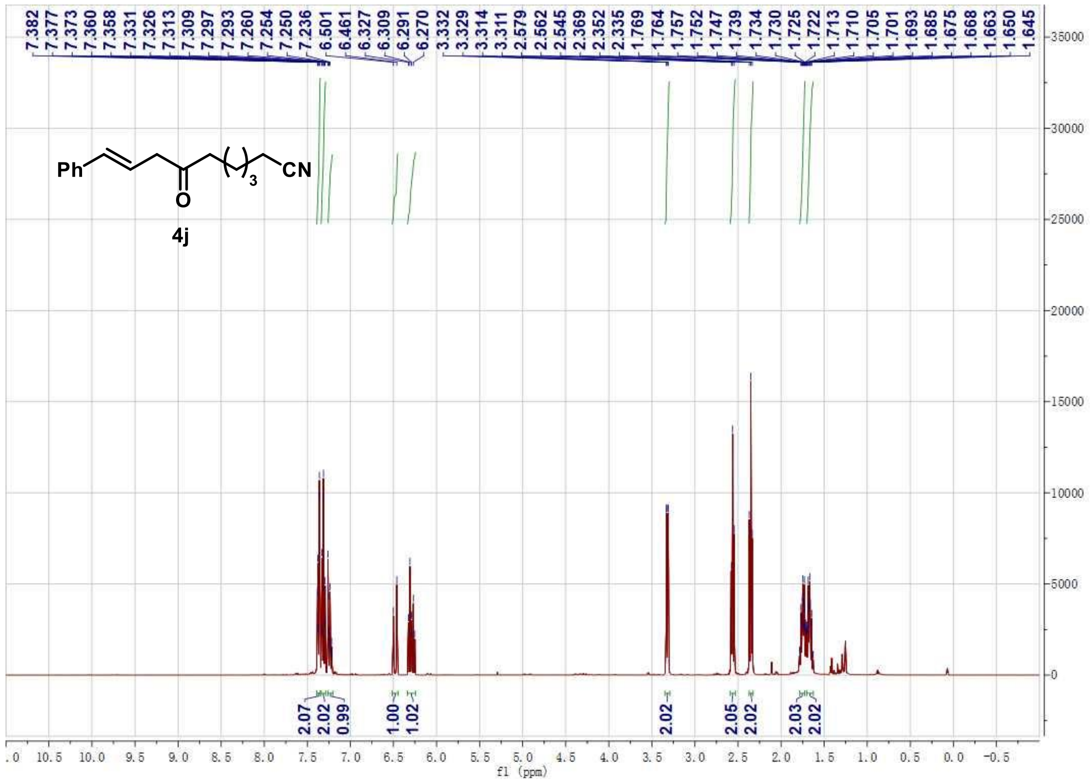

Supplementary Figure 44. ^{1}H NMR (400 MHz, $CDCl_3$) spectrum of 4j

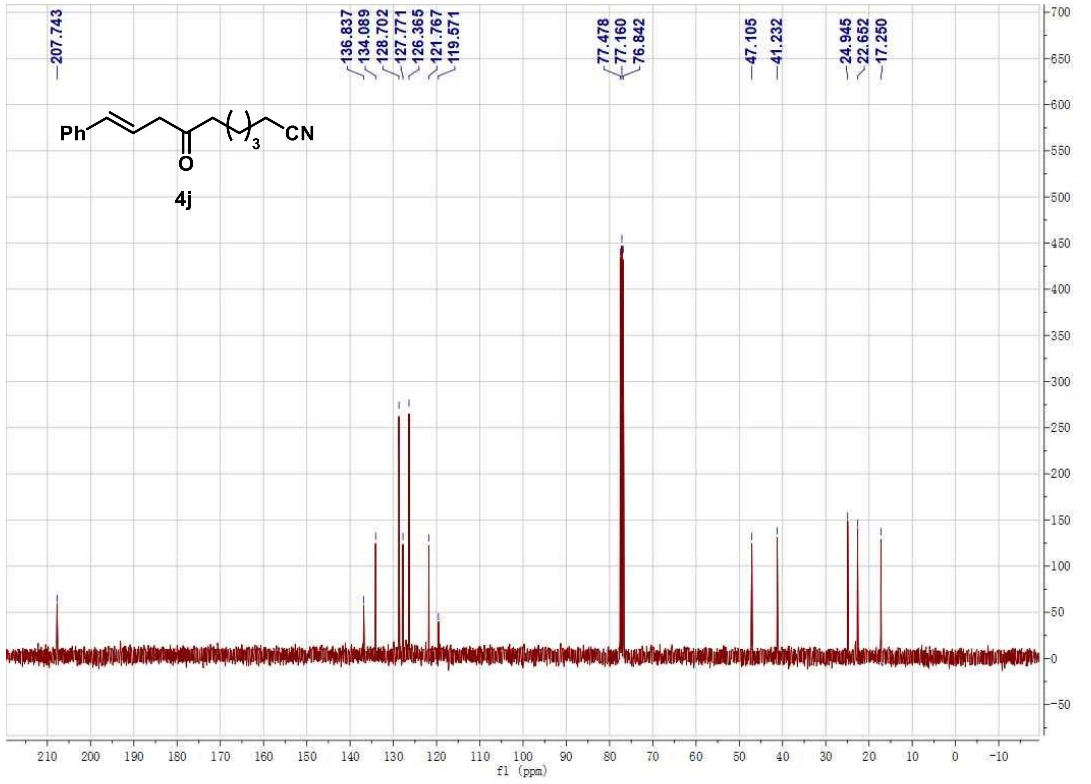

Supplementary Figure 45. ^{13}C NMR (100 MHz, $CDCl_3$) spectrum of 4j

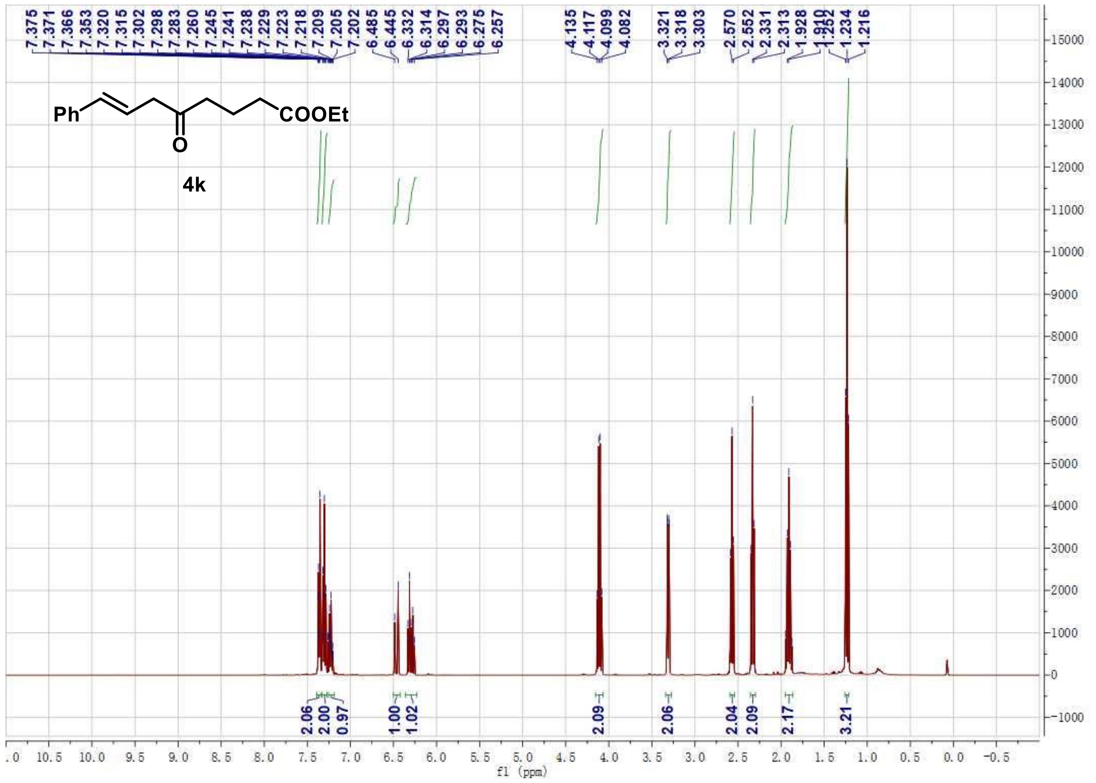

Supplementary Figure 46. ^{1}H NMR (400 MHz, $CDCl_3$) spectrum of 4k

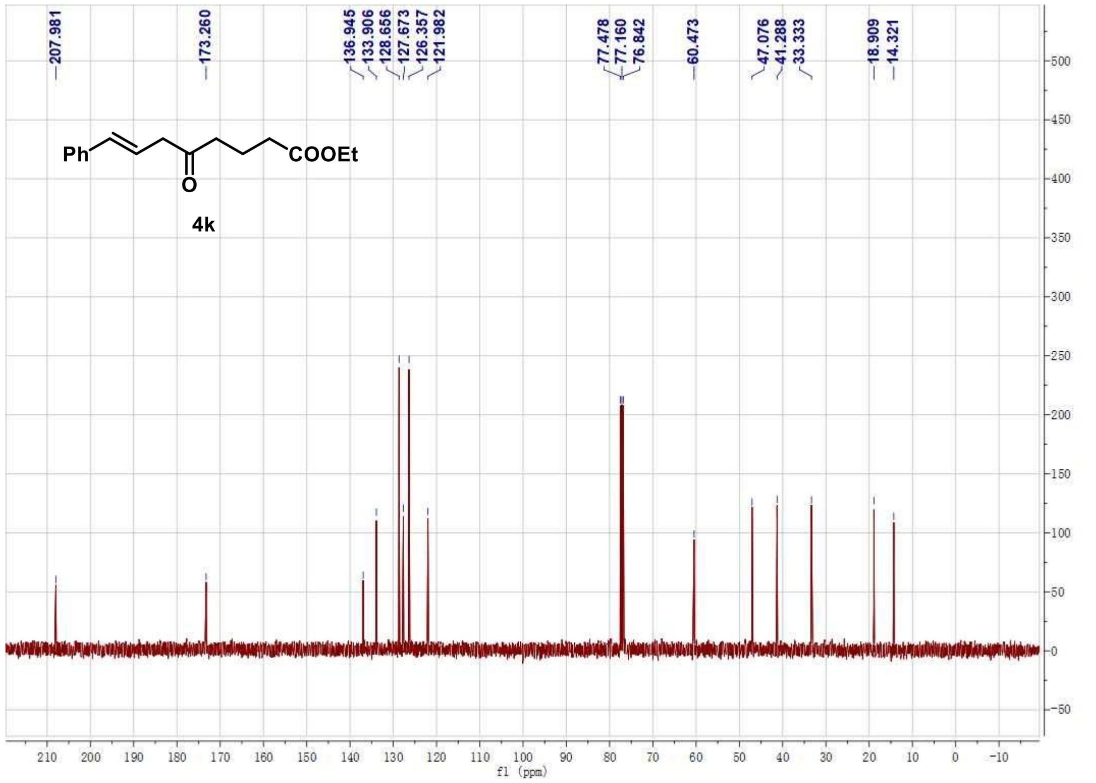

Supplementary Figure 47. ^{13}C NMR (100 MHz, $CDCl_3$) spectrum of 4k

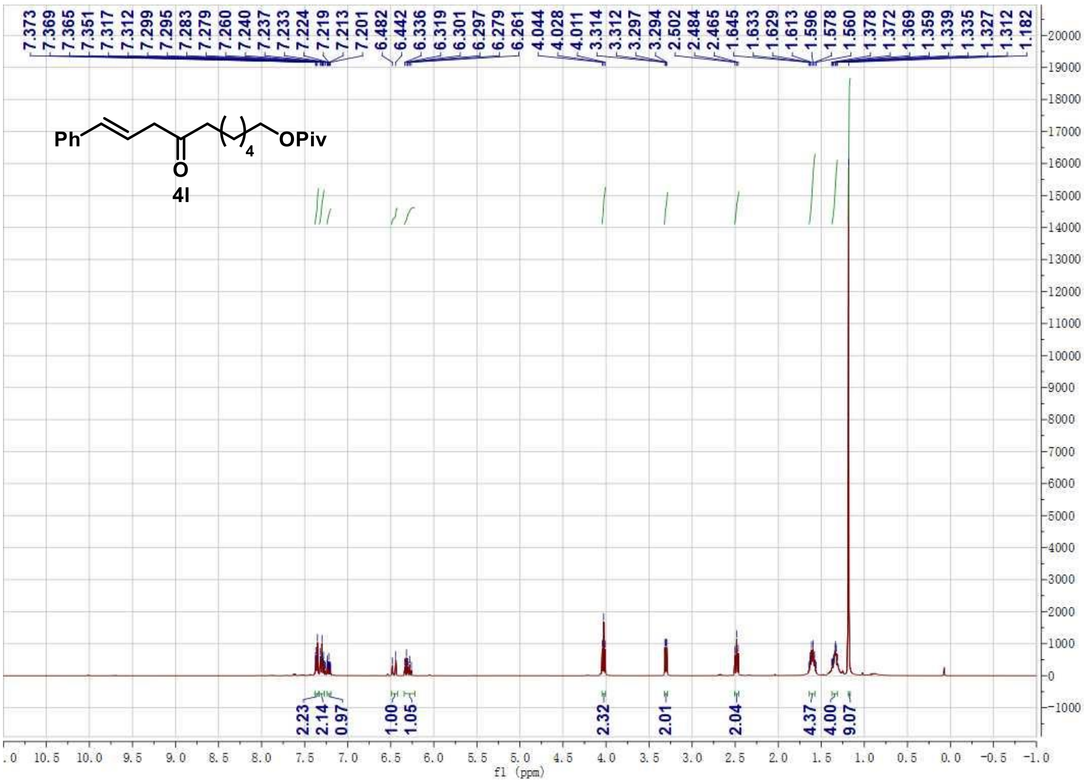

Supplementary Figure 48. ^{1}H NMR (400 MHz, $CDCl_3$) spectrum of 4l

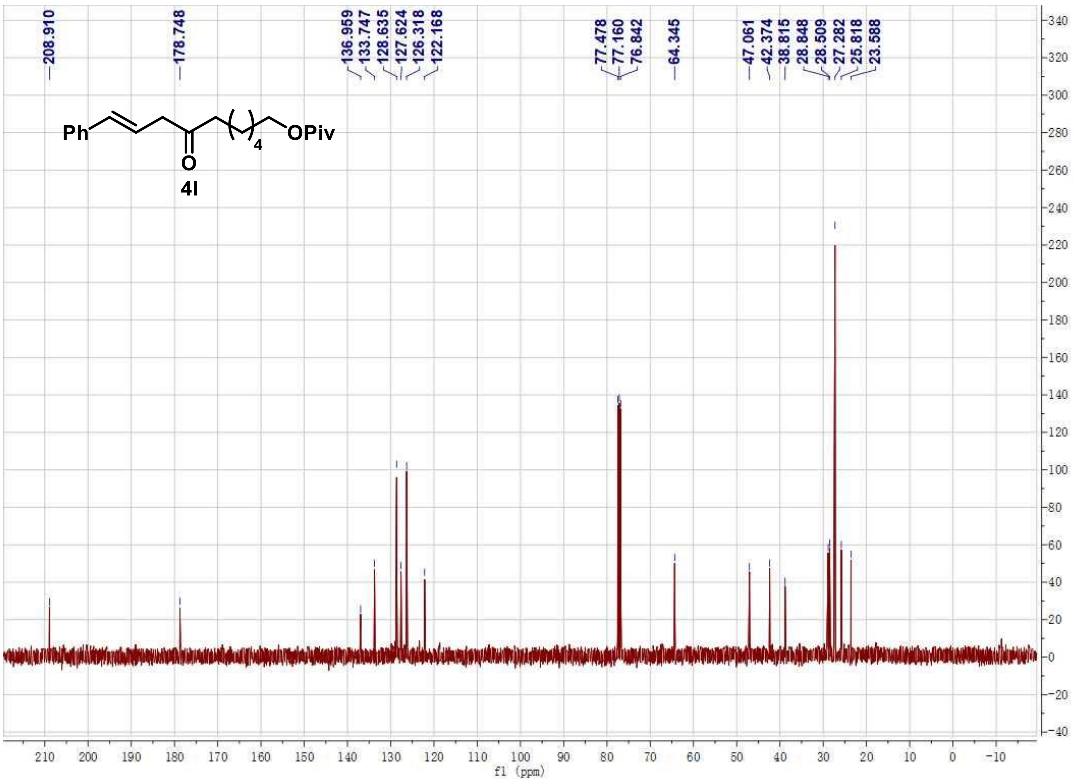

Supplementary Figure 49. ^{13}C NMR (100 MHz, $CDCl_3$) spectrum of 4l

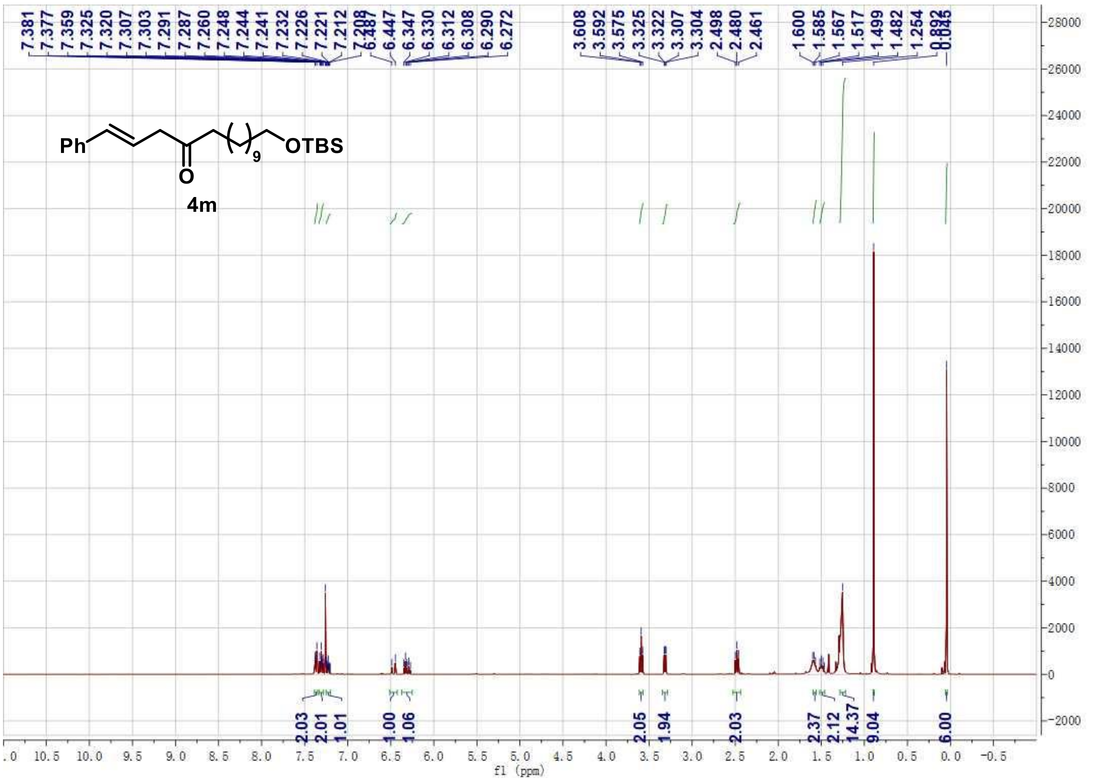

Supplementary Figure 50. ^{1}H NMR (400 MHz, $CDCl_3$) spectrum of 4m

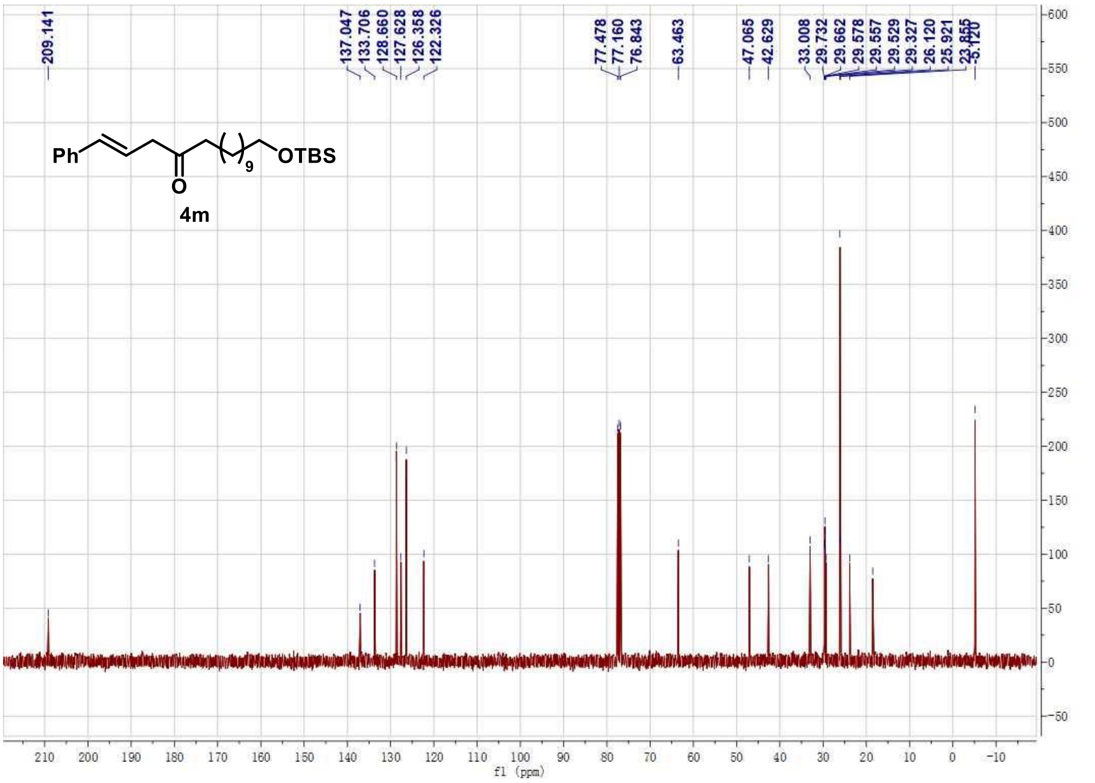

Supplementary Figure 51. ^{13}C NMR (100 MHz, $CDCl_3$) spectrum of 4m

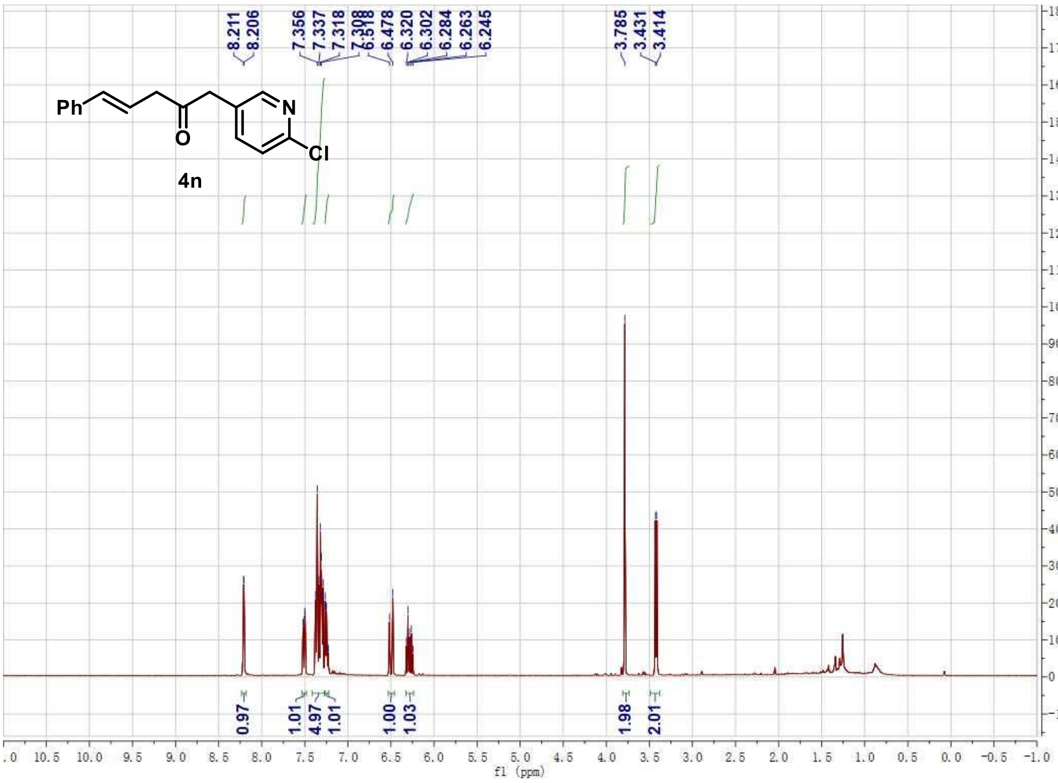

Supplementary Figure 52. **^{1}H NMR (400 MHz, CDCl3) spectrum of 4n**

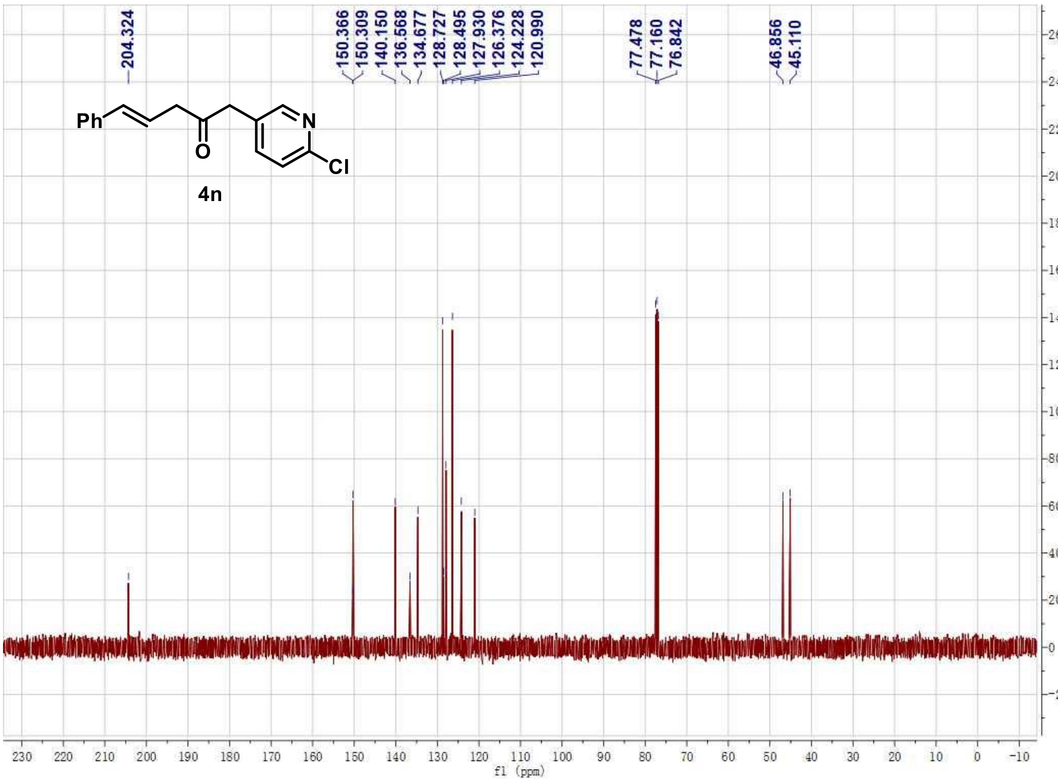

Supplementary Figure 53. **^{13}C NMR (100 MHz, CDCl3) spectrum of 4n**

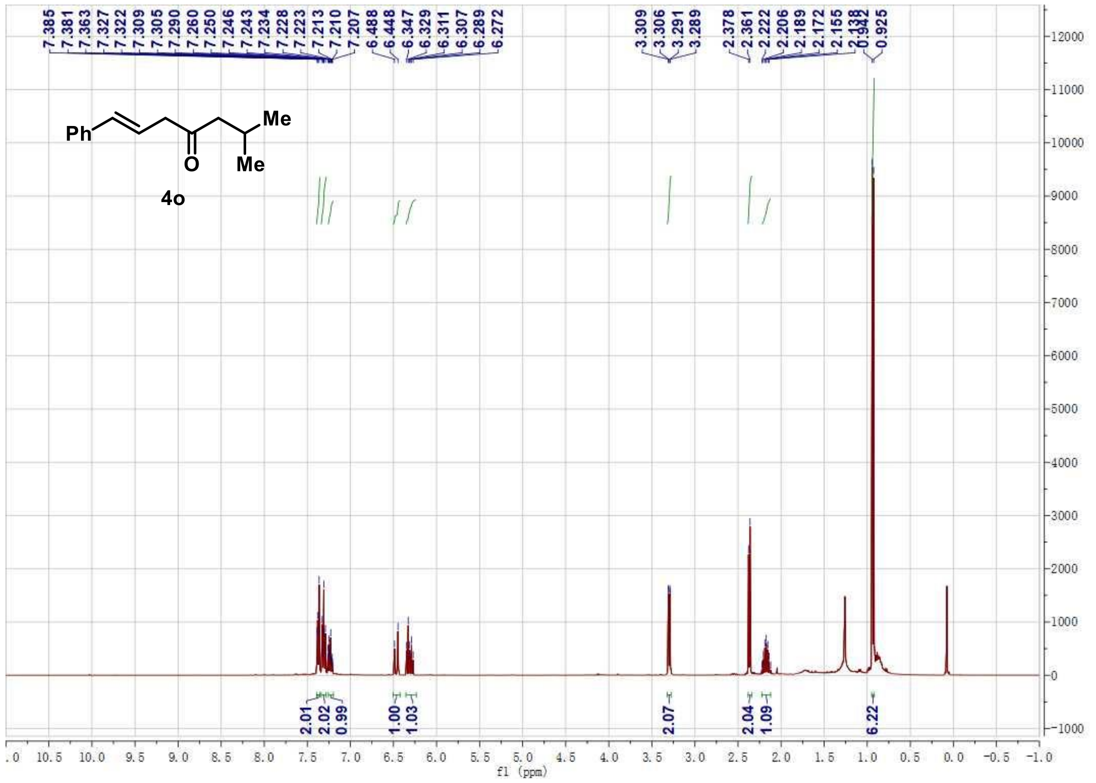

Supplementary Figure 54. ^{1}H NMR (400 MHz, $CDCl_3$) spectrum of 4o

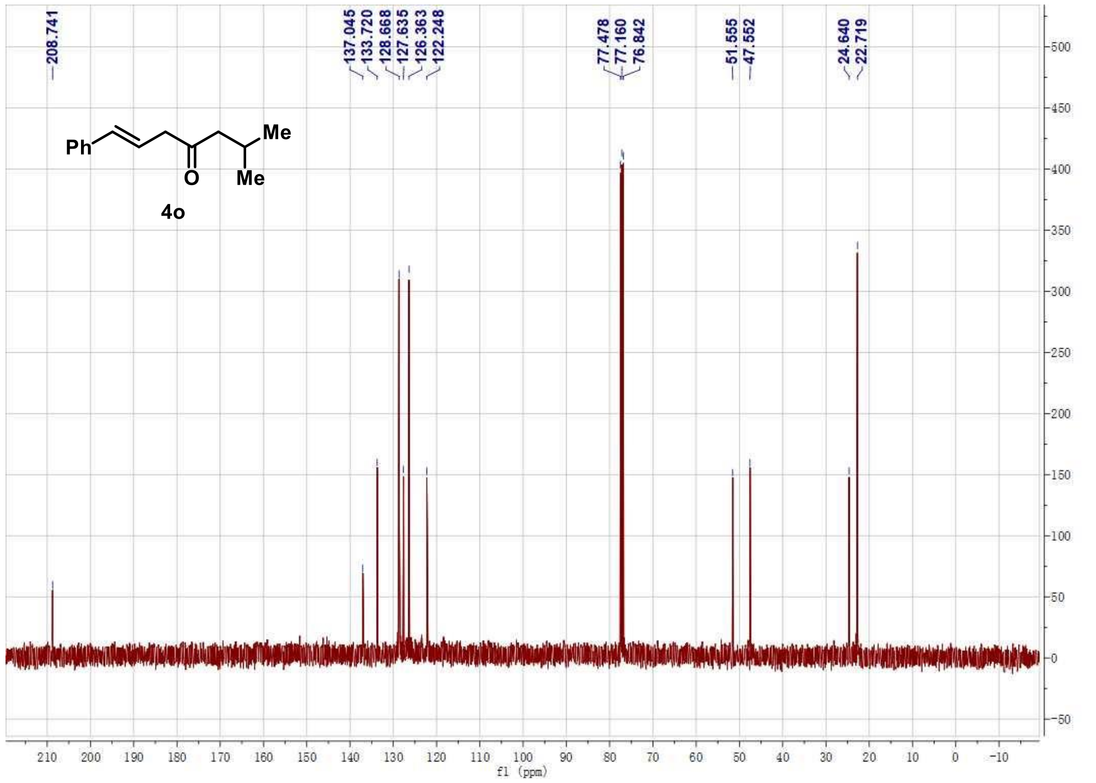

Supplementary Figure 55. ^{13}C NMR (100 MHz, $CDCl_3$) spectrum of 4o

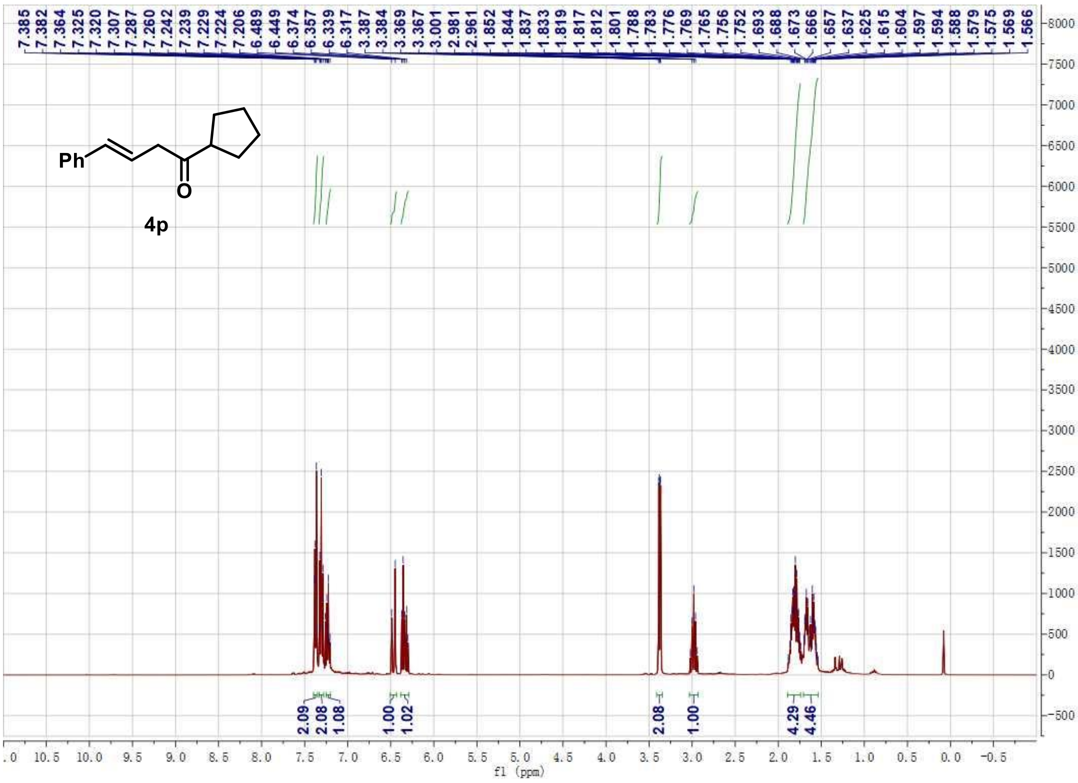

Supplementary Figure 56. ^{1}H NMR (400 MHz, $CDCl_3$) spectrum of 4p

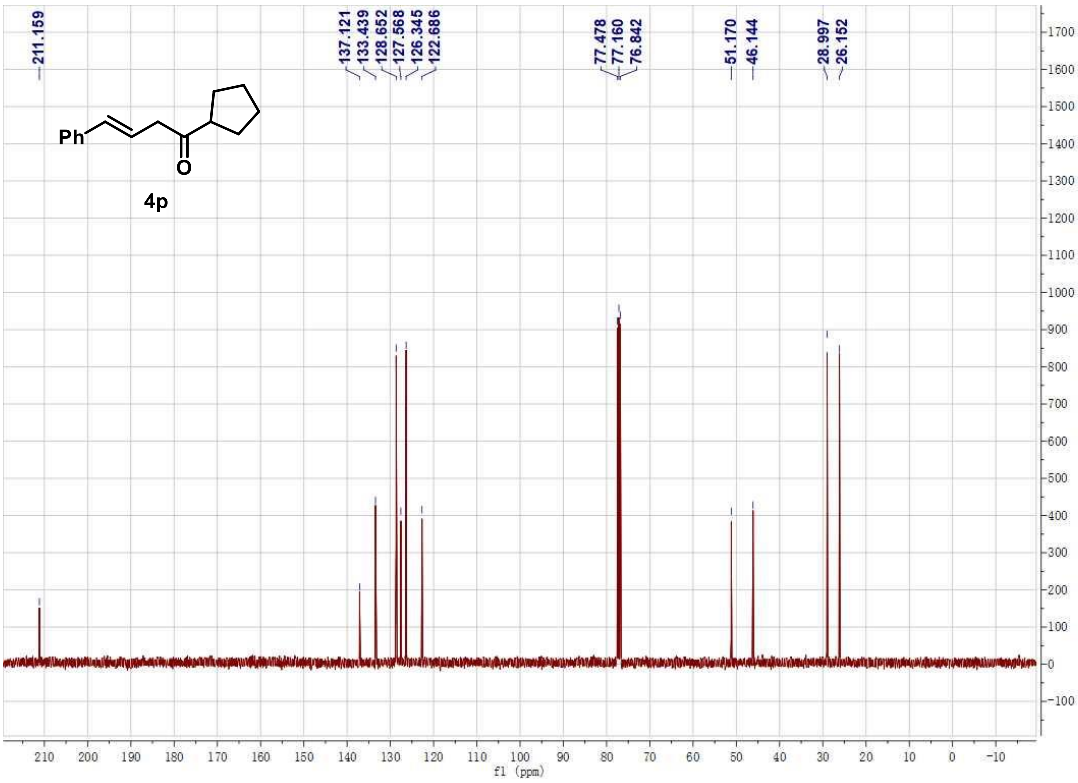

Supplementary Figure 57. ^{13}C NMR (100 MHz, $CDCl_3$) spectrum of 4p

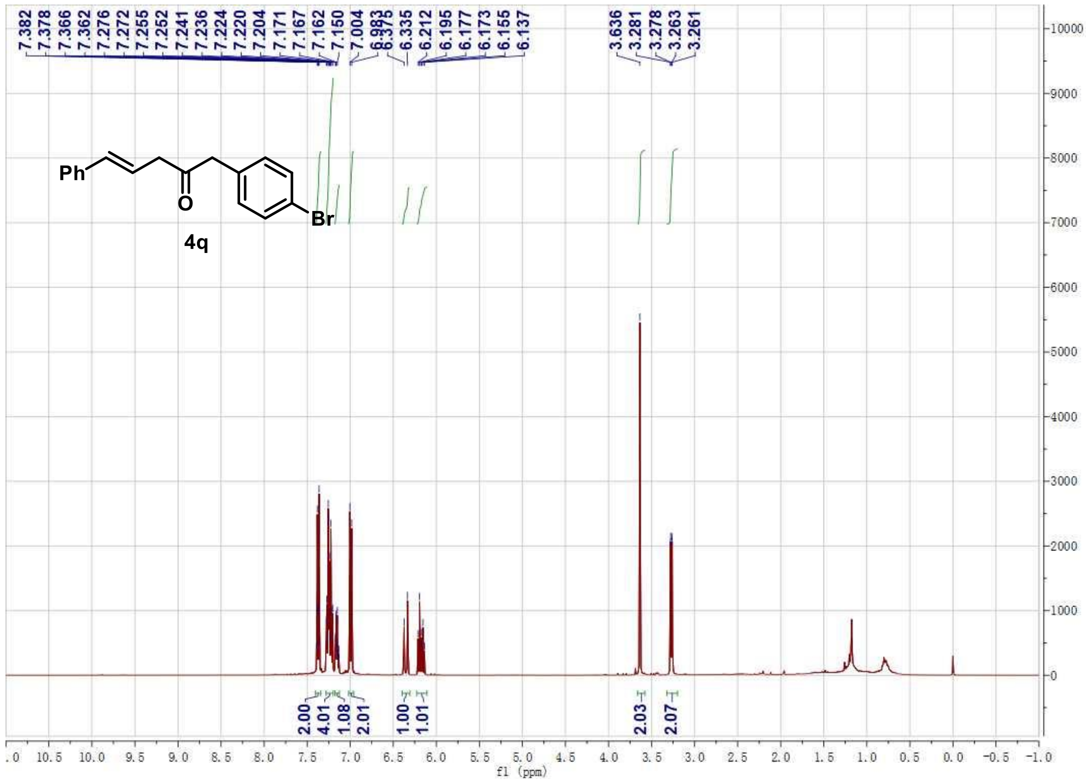

Supplementary Figure 58. ^{1}H NMR (400 MHz, $CDCl_3$) spectrum of 4q

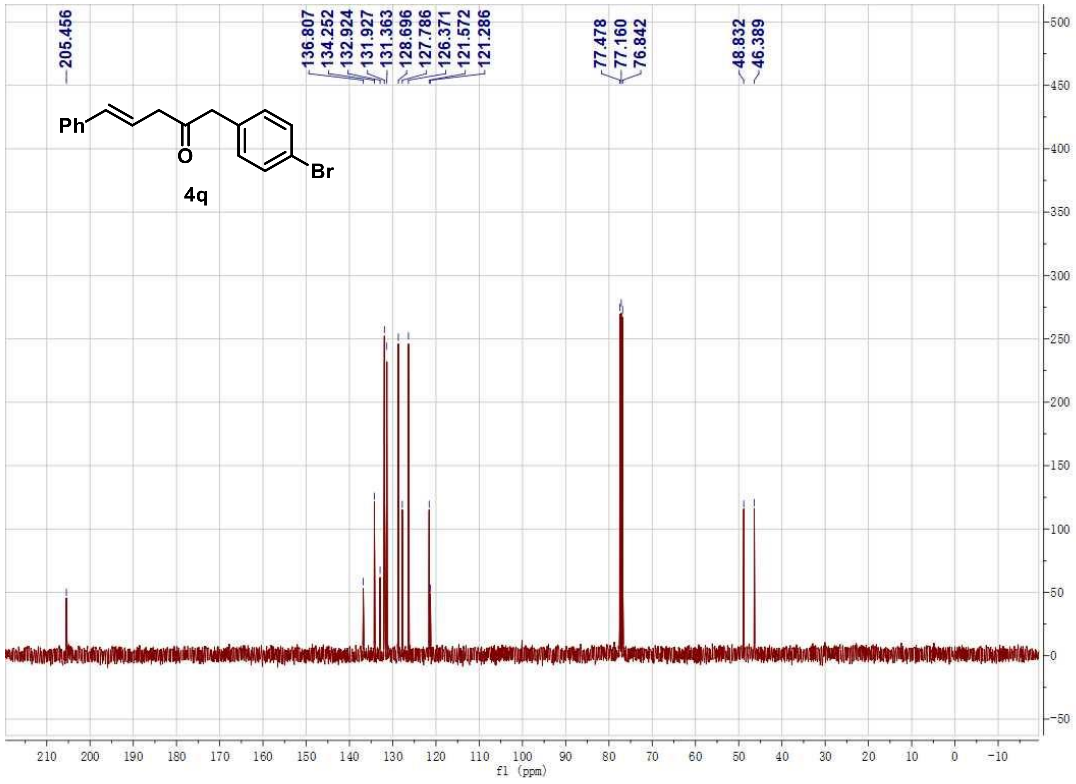

Supplementary Figure 59. ^{13}C NMR (100 MHz, $CDCl_3$) spectrum of 4q

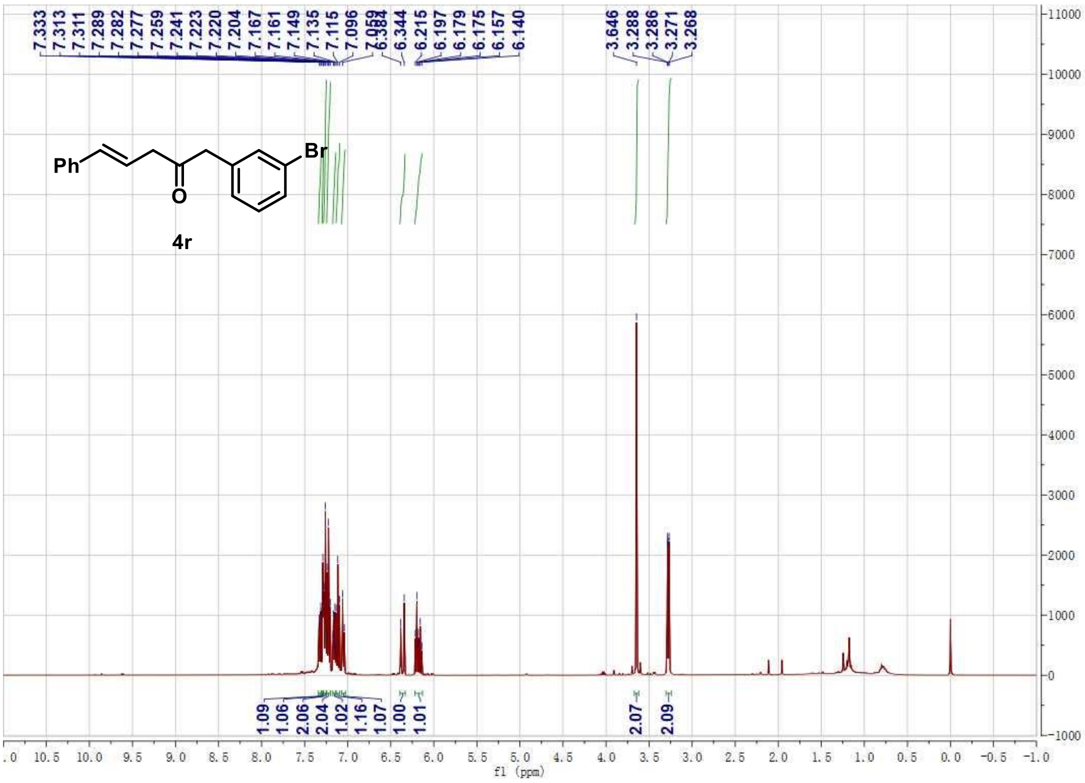

Supplementary Figure 60. ^{1}**H NMR (400 MHz, CDCl$_3$) spectrum of 4r**

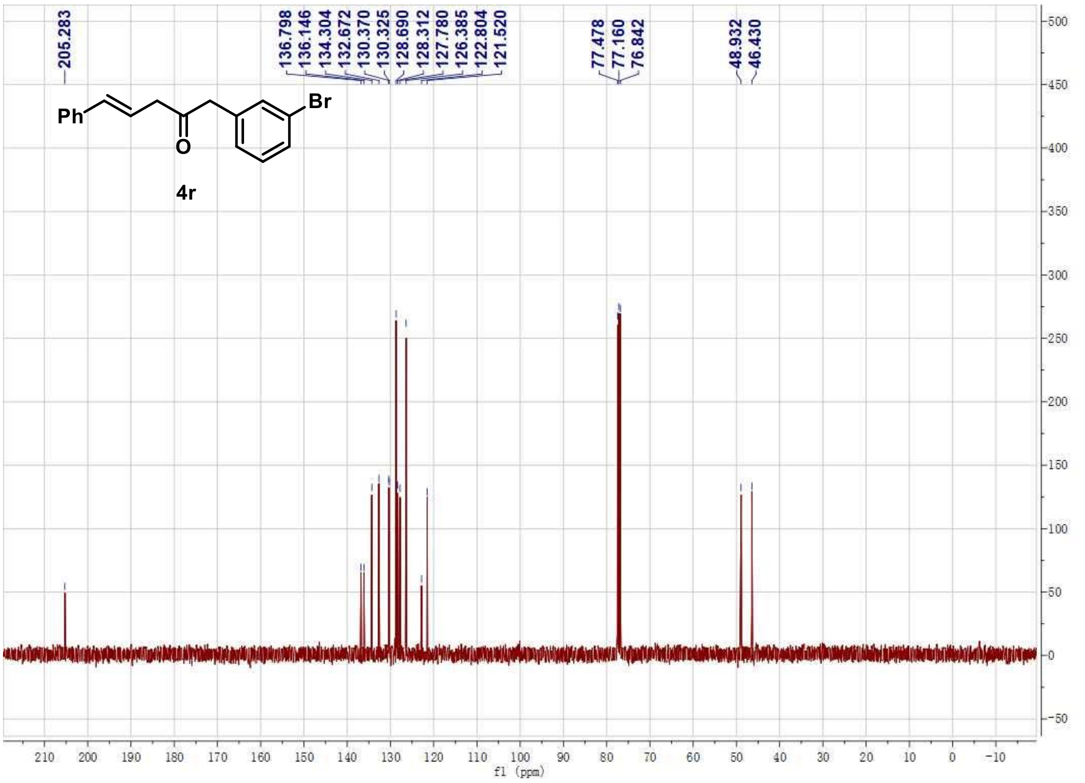

Supplementary Figure 61. ^{13}**C NMR (100 MHz, CDCl$_3$) spectrum of 4r**

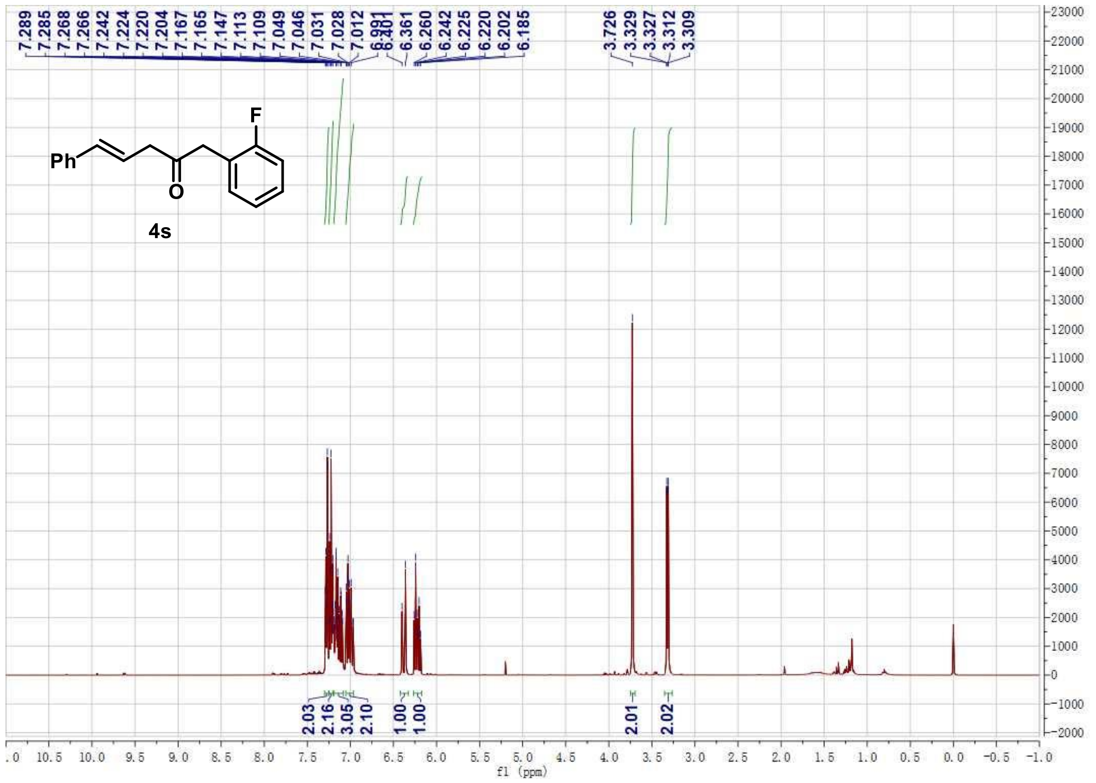

Supplementary Figure 62. ¹H NMR (400 MHz, CDCl₃) spectrum of 4s

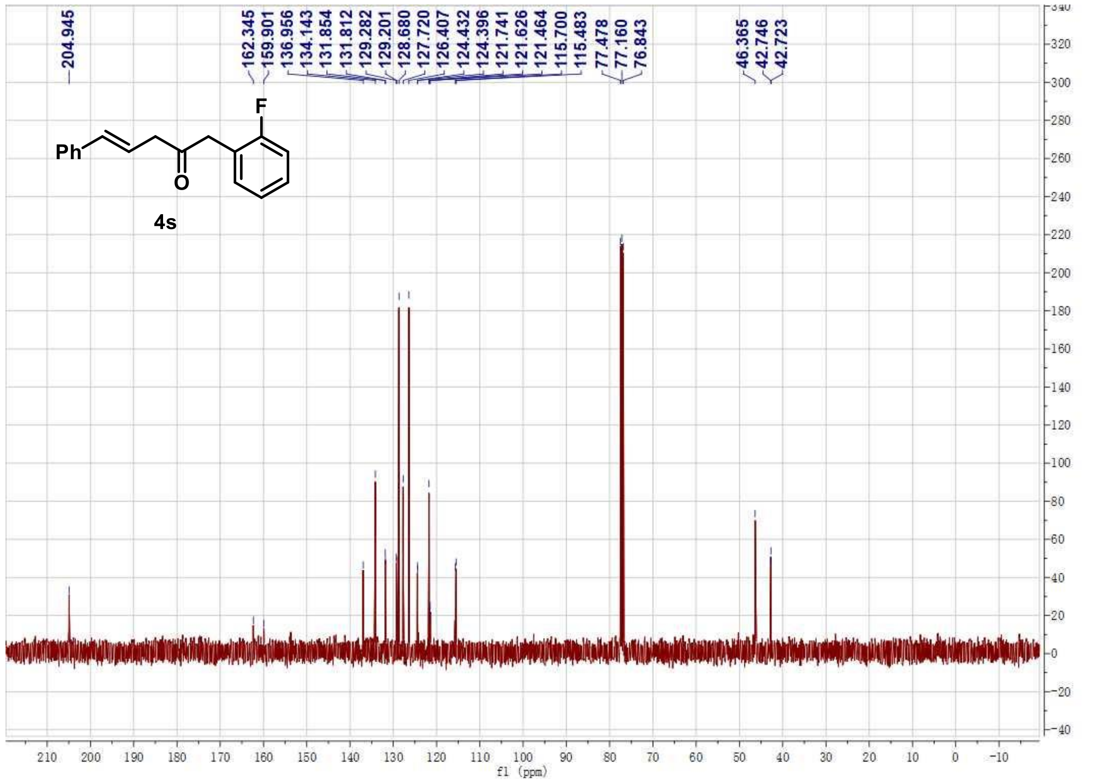

Supplementary Figure 63. ¹³C NMR (100 MHz, CDCl₃) spectrum of 4s

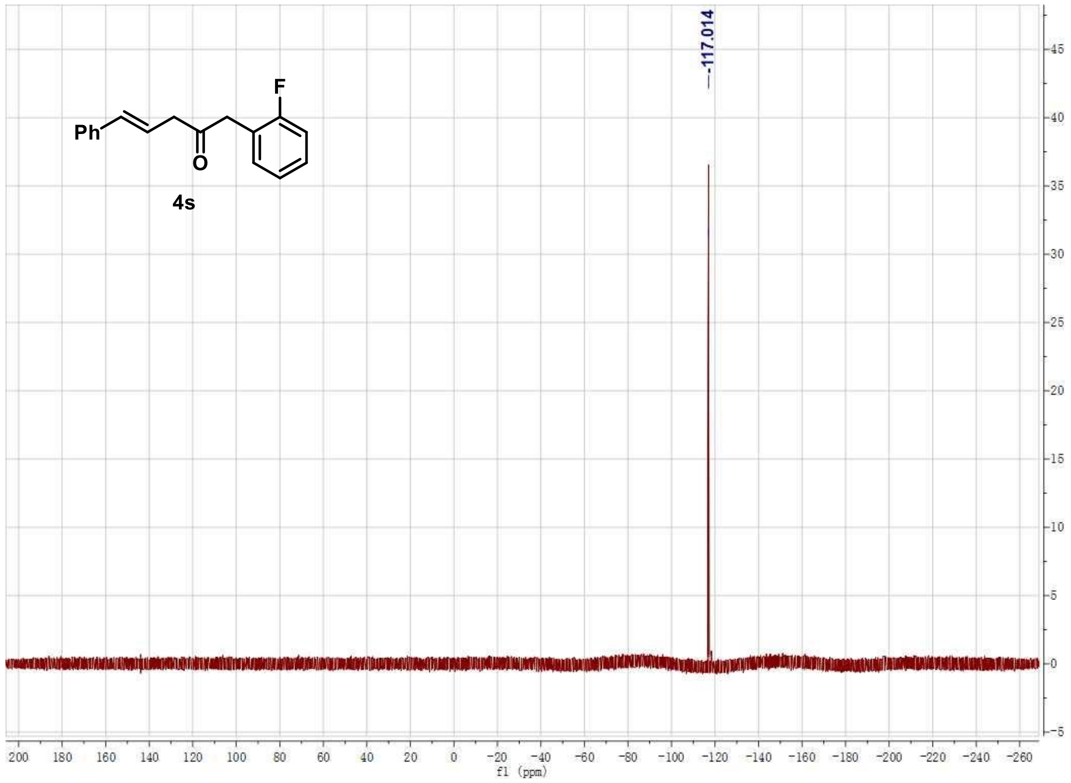

Supplementary Figure 64. ^{19}F NMR (376 MHz, $CDCl_3$) spectrum of 4s

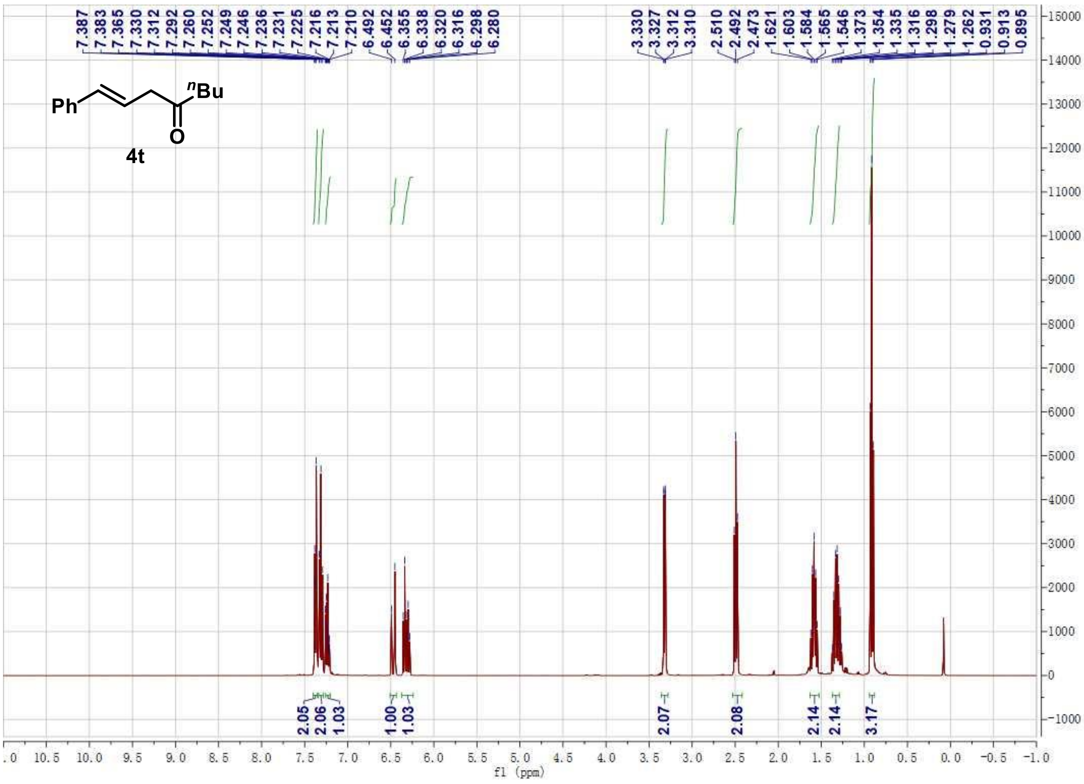

Supplementary Figure 65. ^{1}H NMR (400 MHz, $CDCl_3$) spectrum of 4t

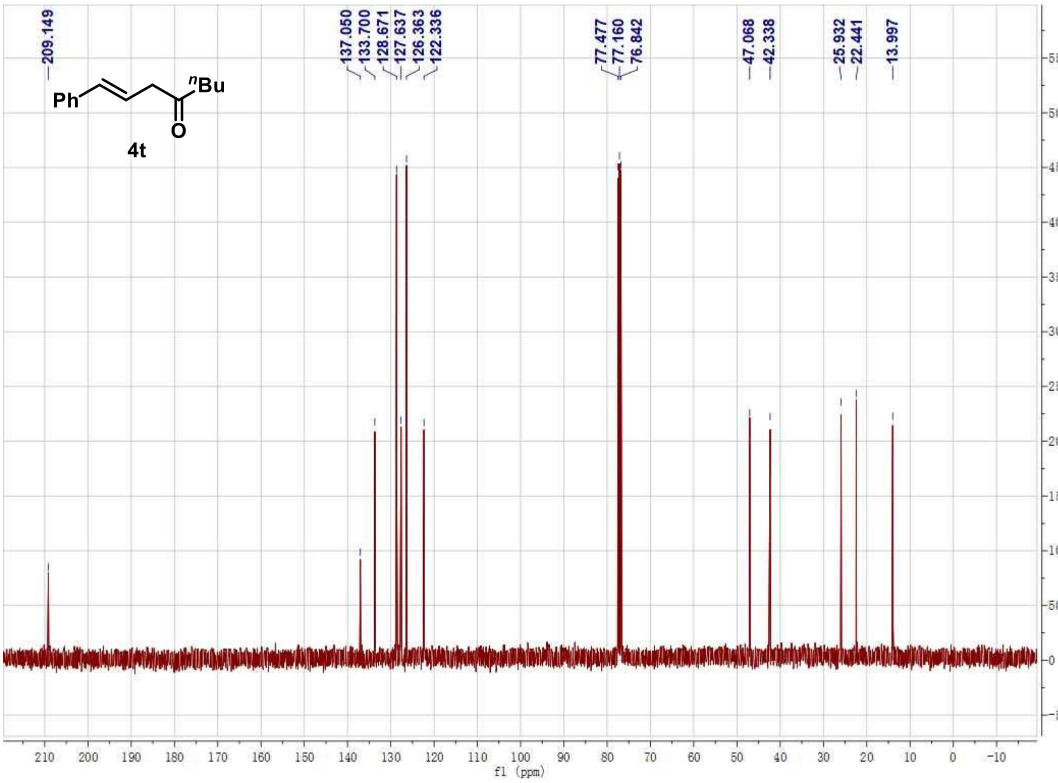

Supplementary Figure 66. ^{13}C NMR (100 MHz, $CDCl_3$) spectrum of 4t

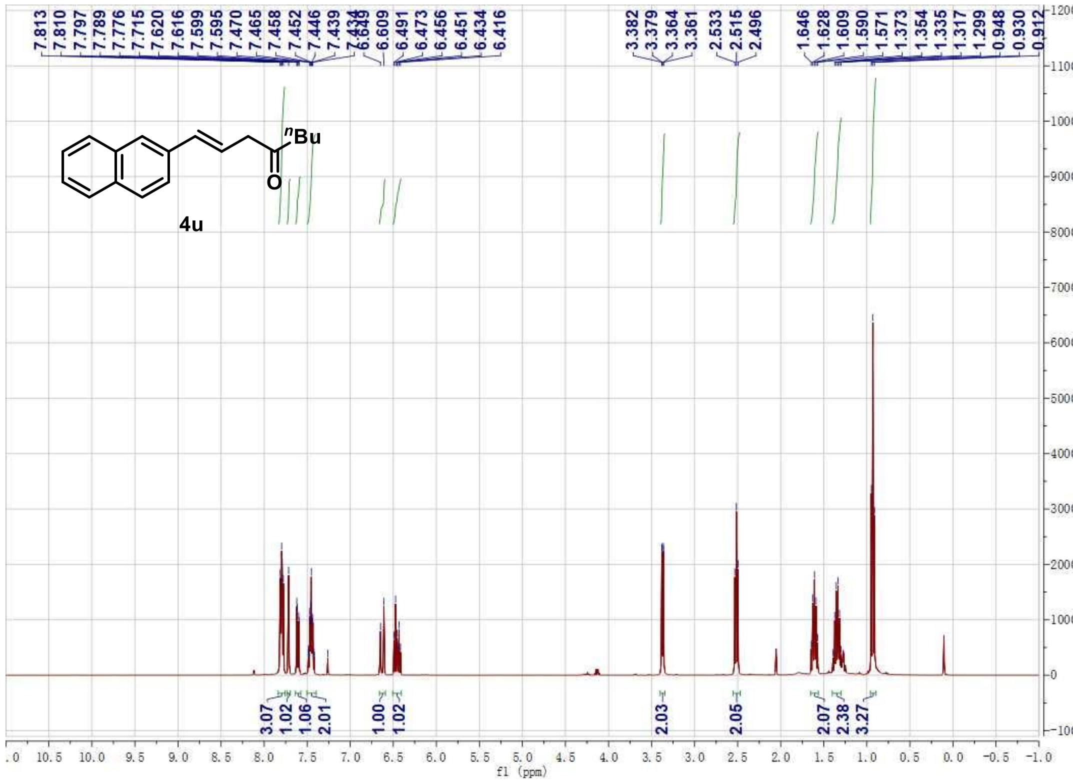

Supplementary Figure 67. ^{1}H NMR (400 MHz, $CDCl_3$) spectrum of 4u

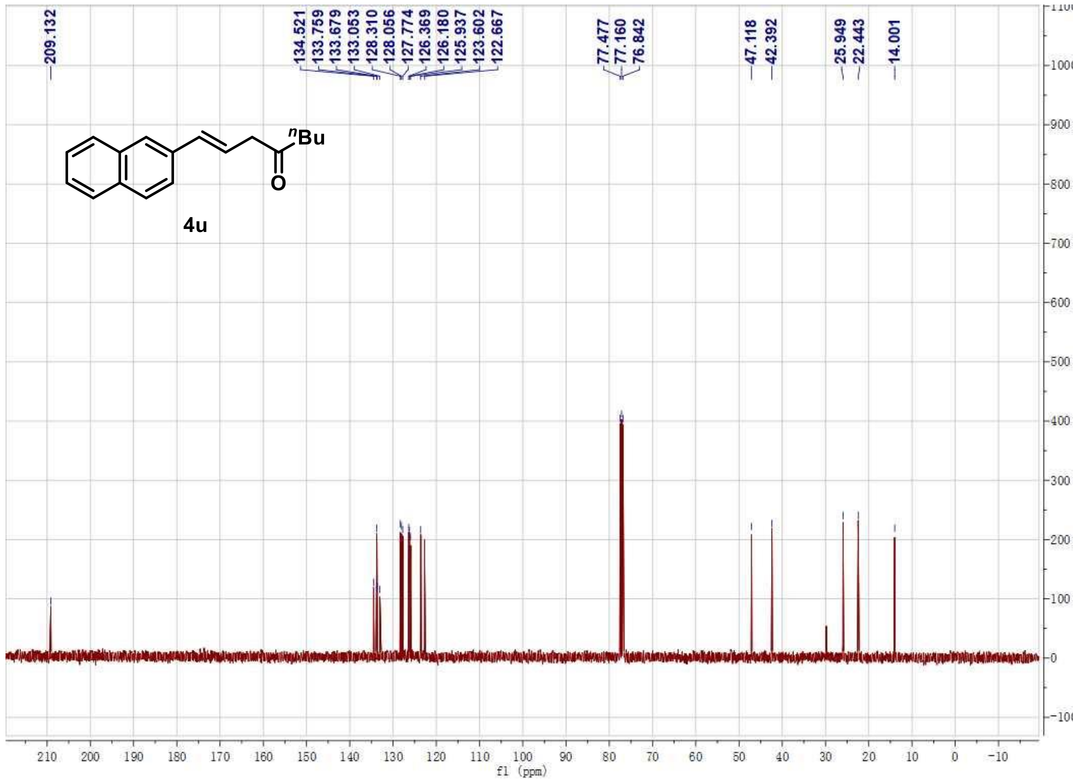

Supplementary Figure 68. ^{13}C NMR (100 MHz, $CDCl_3$) spectrum of 4u

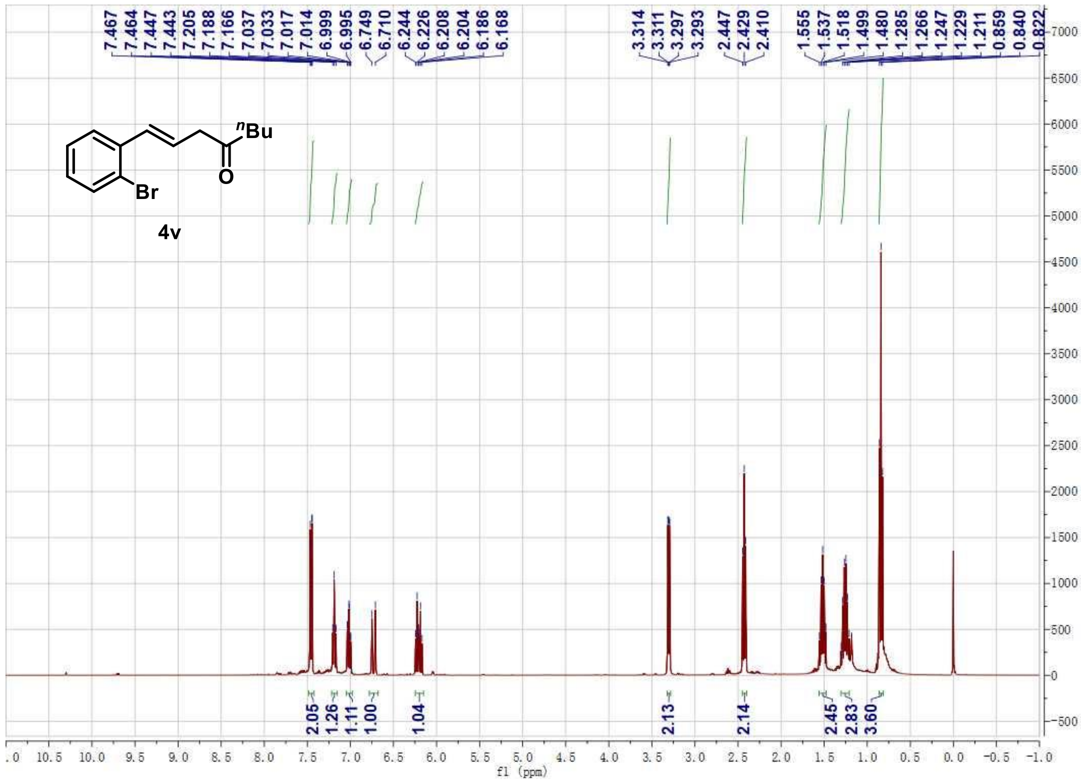

Supplementary Figure 69. ^{1}H NMR (400 MHz, CDCl$_3$) spectrum of 4v

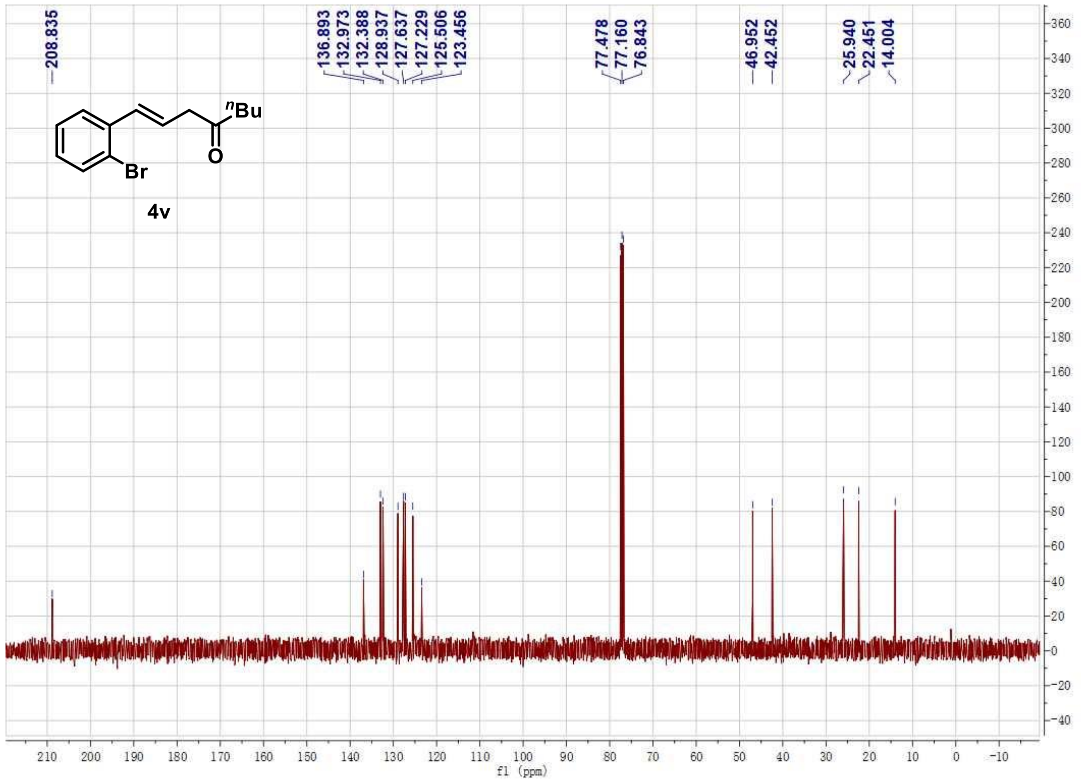

Supplementary Figure 70. ^{13}C NMR (100 MHz, CDCl$_3$) spectrum of 4v

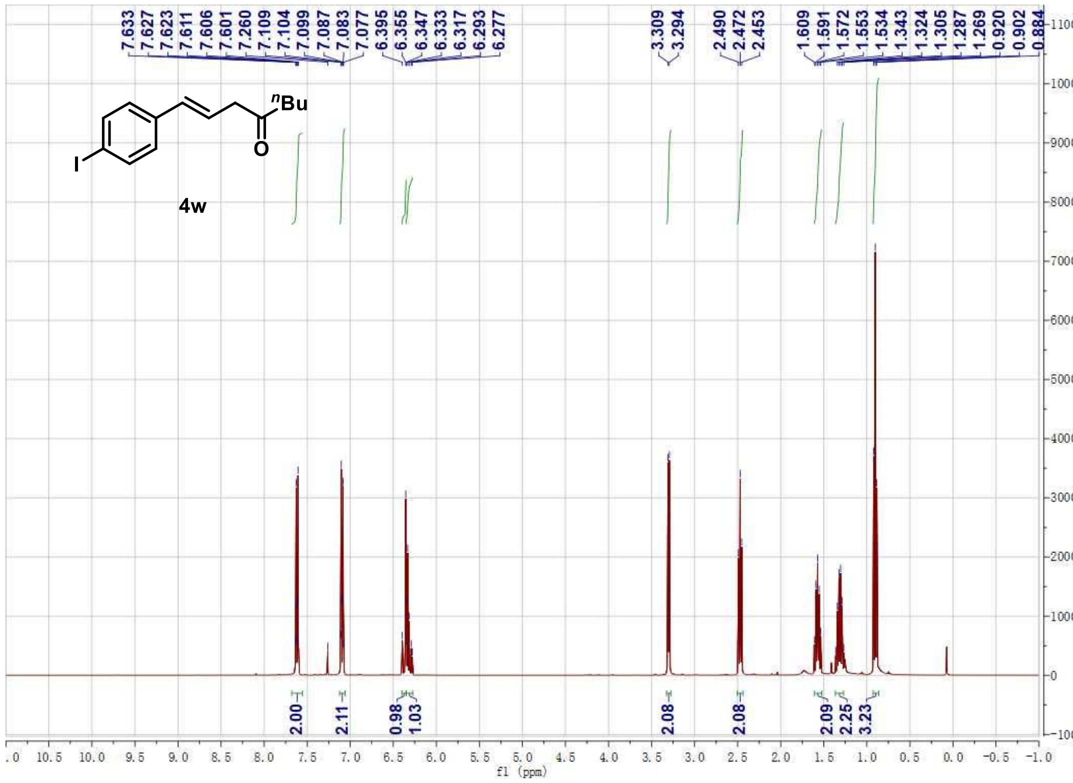

Supplementary Figure 71. ^{1}H NMR (400 MHz, $CDCl_3$) spectrum of 4w

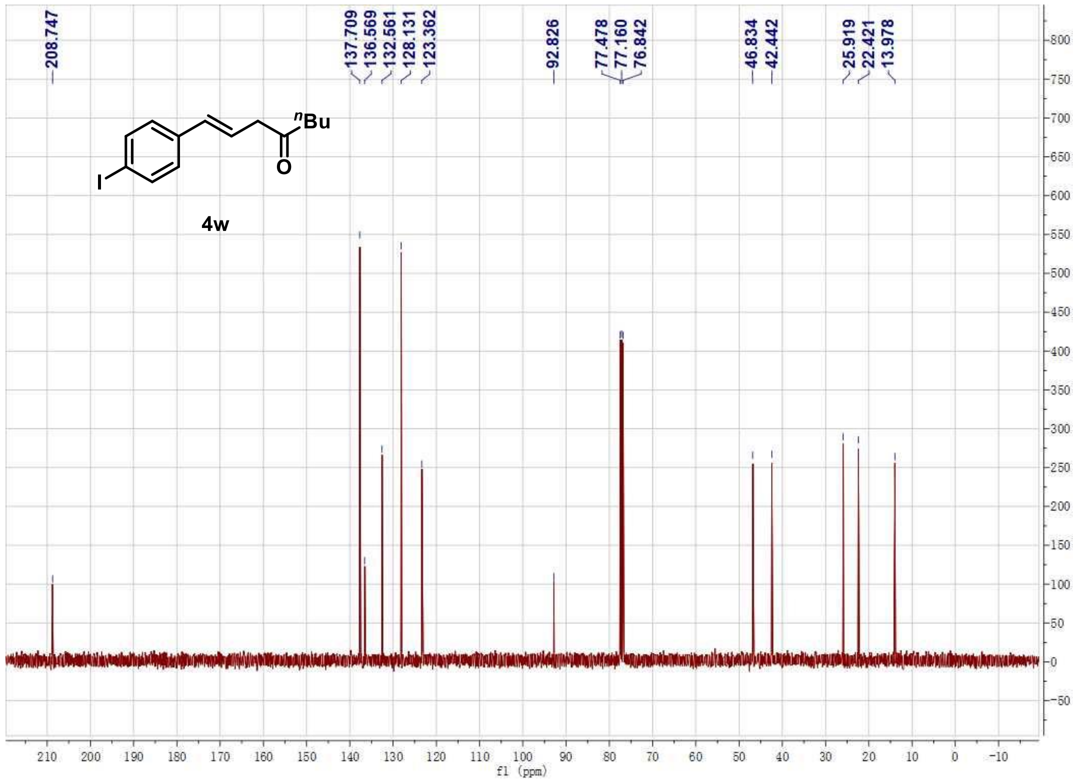

Supplementary Figure 72. ^{13}C NMR (100 MHz, $CDCl_3$) spectrum of 4w

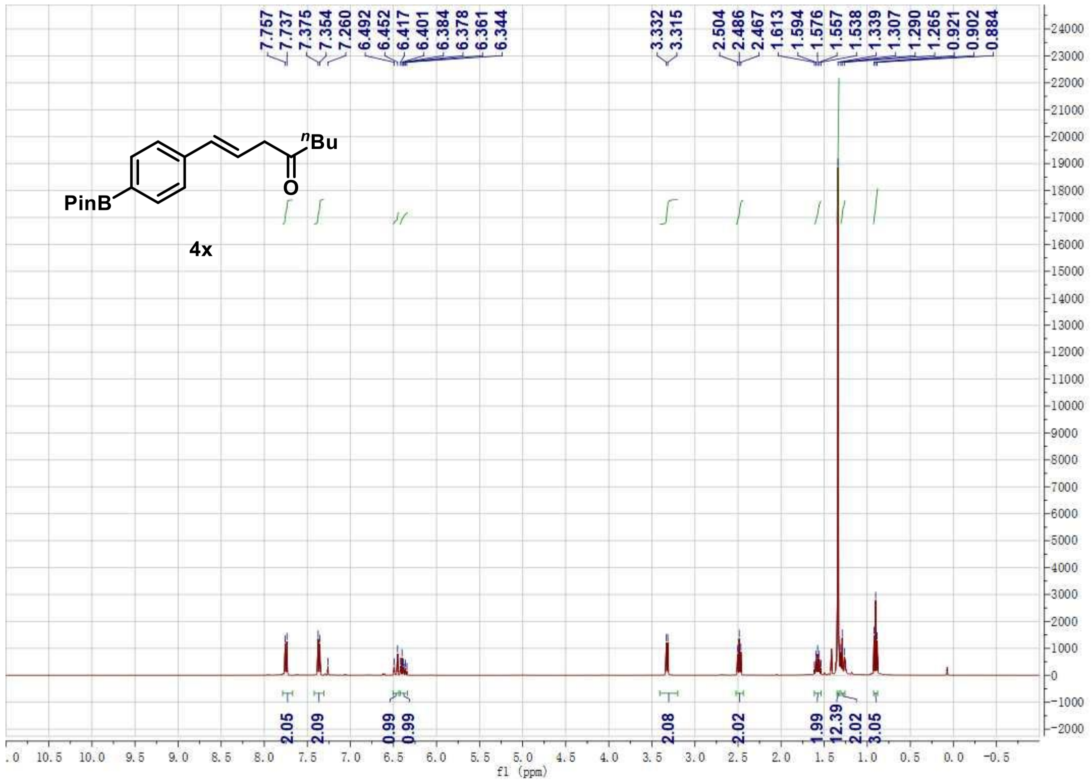

Supplementary Figure 73. ^{1}H NMR (400 MHz, $CDCl_3$) spectrum of 4x

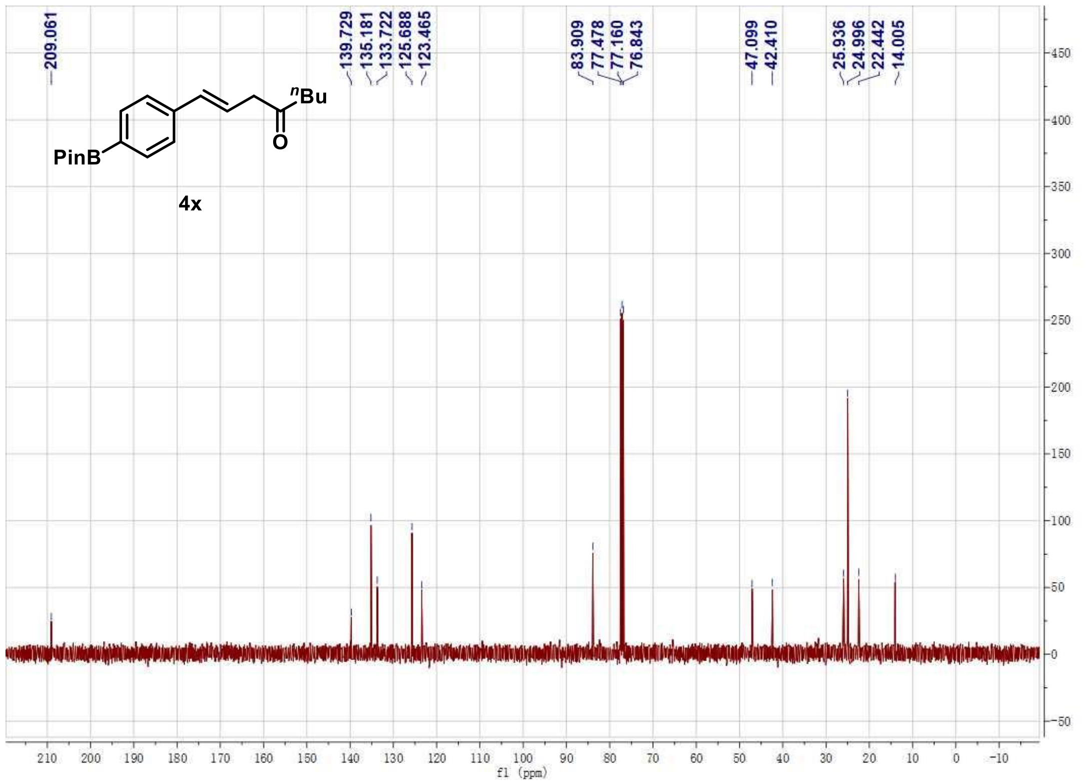

Supplementary Figure 74. ^{13}C NMR (100 MHz, $CDCl_3$) spectrum of 4x

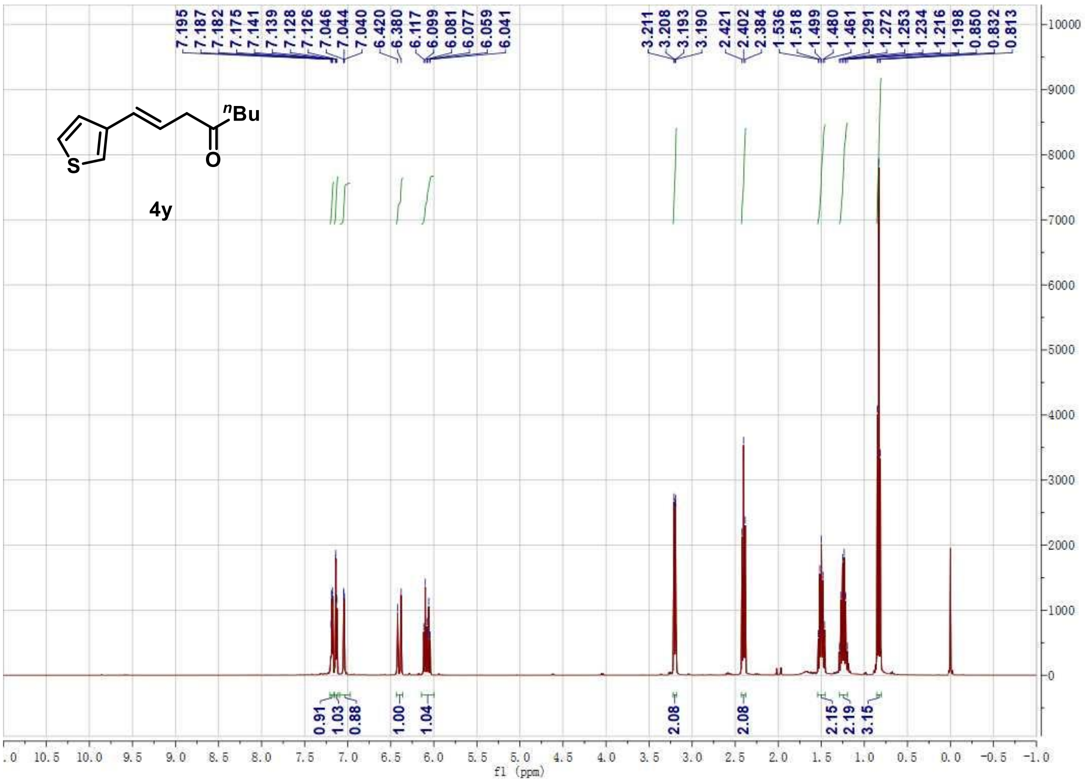

Supplementary Figure 75. ^{1}H NMR (400 MHz, $CDCl_3$) spectrum of 4y

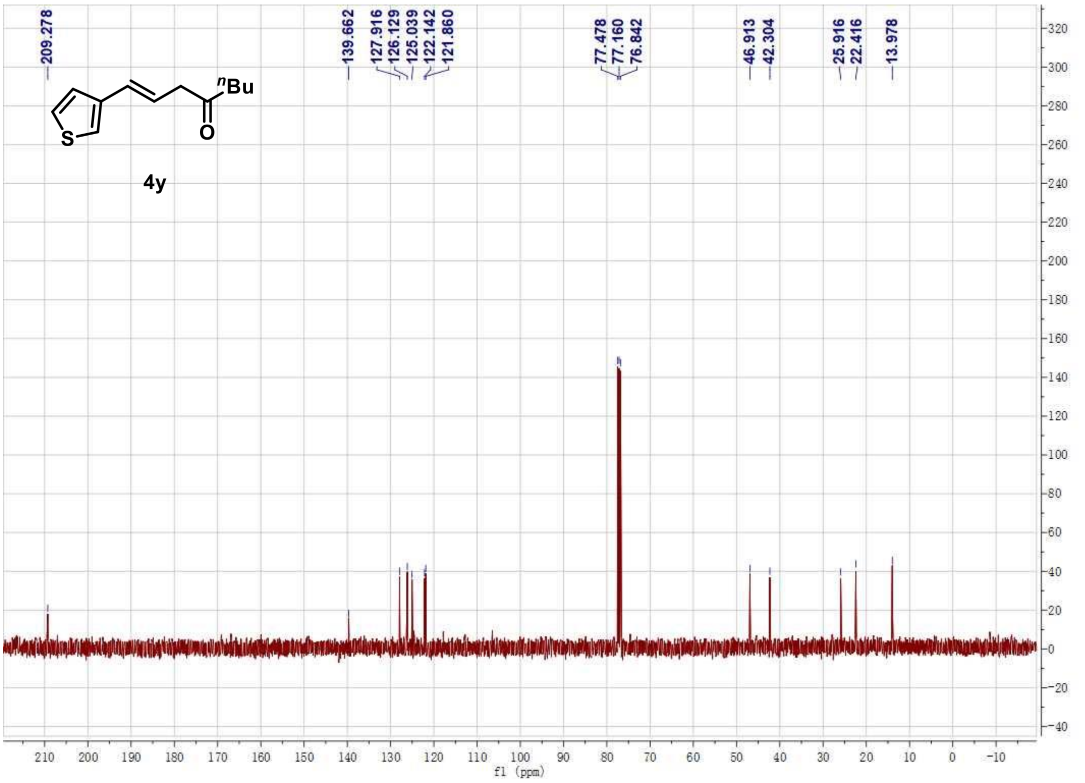

Supplementary Figure 76. ^{13}C NMR (100 MHz, $CDCl_3$) spectrum of 4y

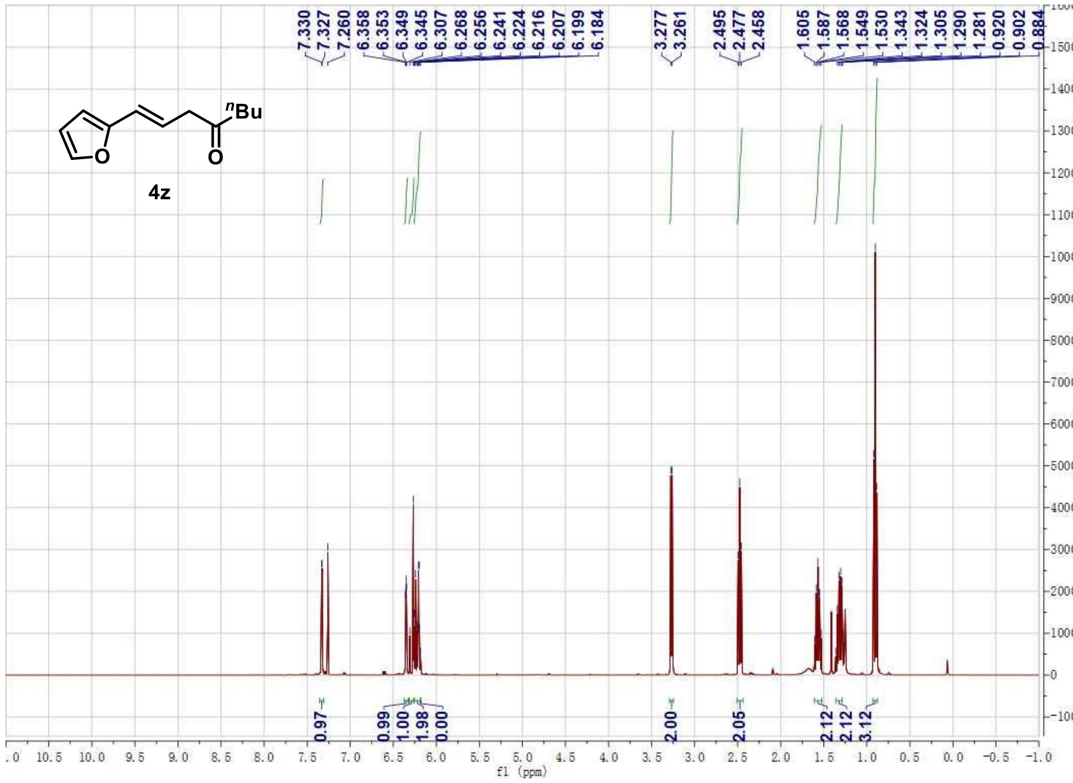

Supplementary Figure 77. ^{1}H NMR (400 MHz, CDCl$_3$) spectrum of 4z

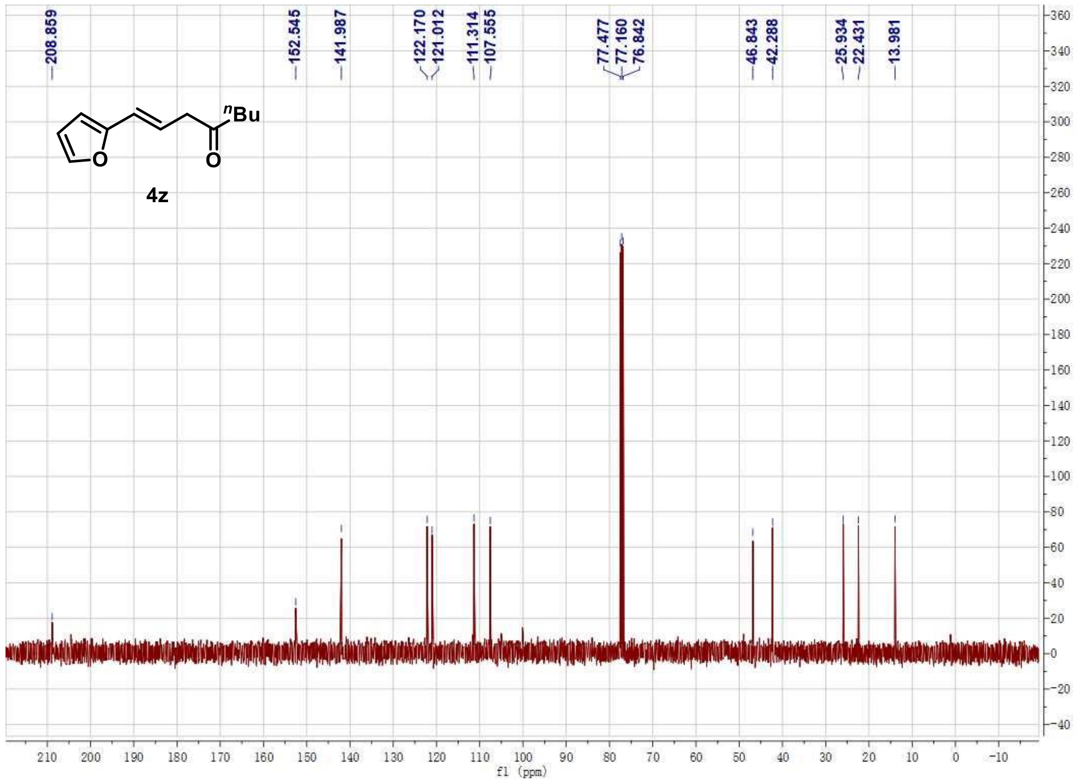

Supplementary Figure 78. ^{13}C NMR (100 MHz, CDCl$_3$) spectrum of 4z

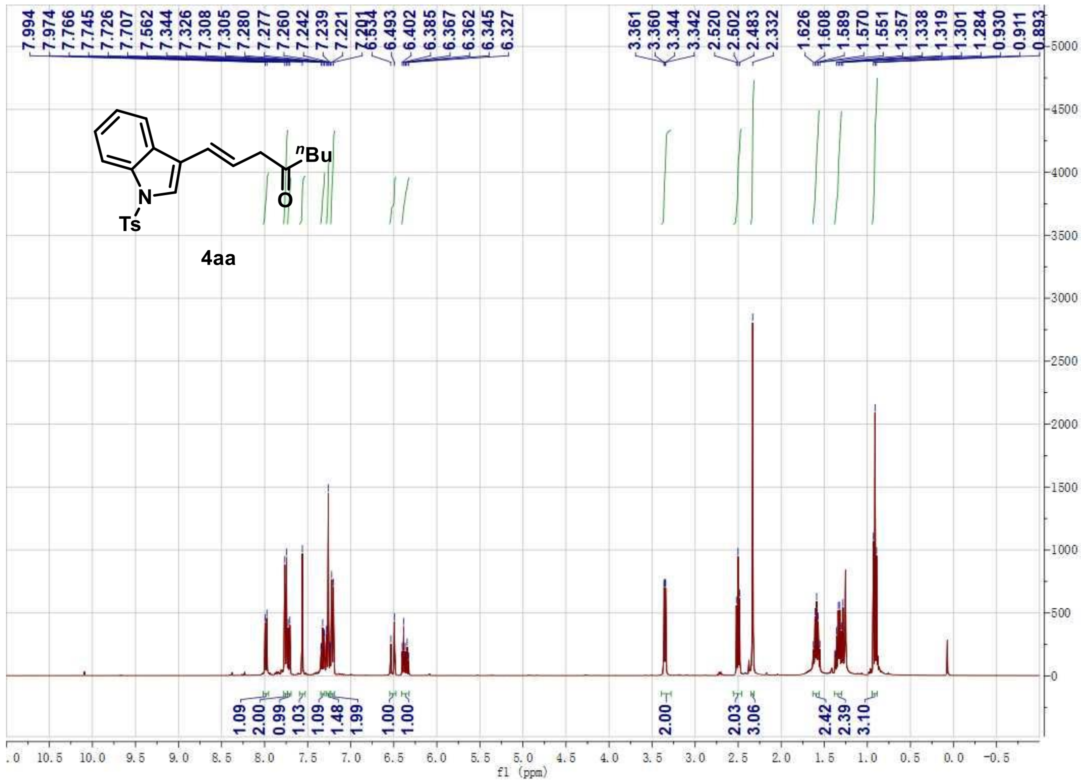

Supplementary Figure 79. ^{1}H NMR (400 MHz, $CDCl_3$) spectrum of 4aa

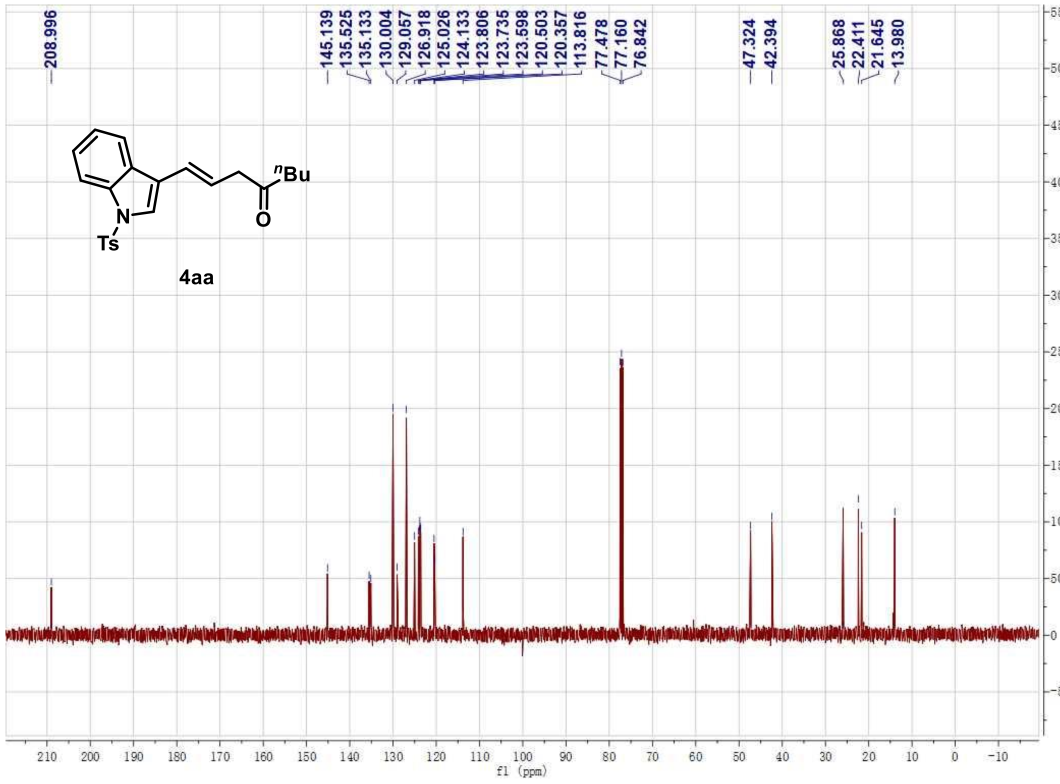

Supplementary Figure 80. ^{13}C NMR (100 MHz, $CDCl_3$) spectrum of 4aa

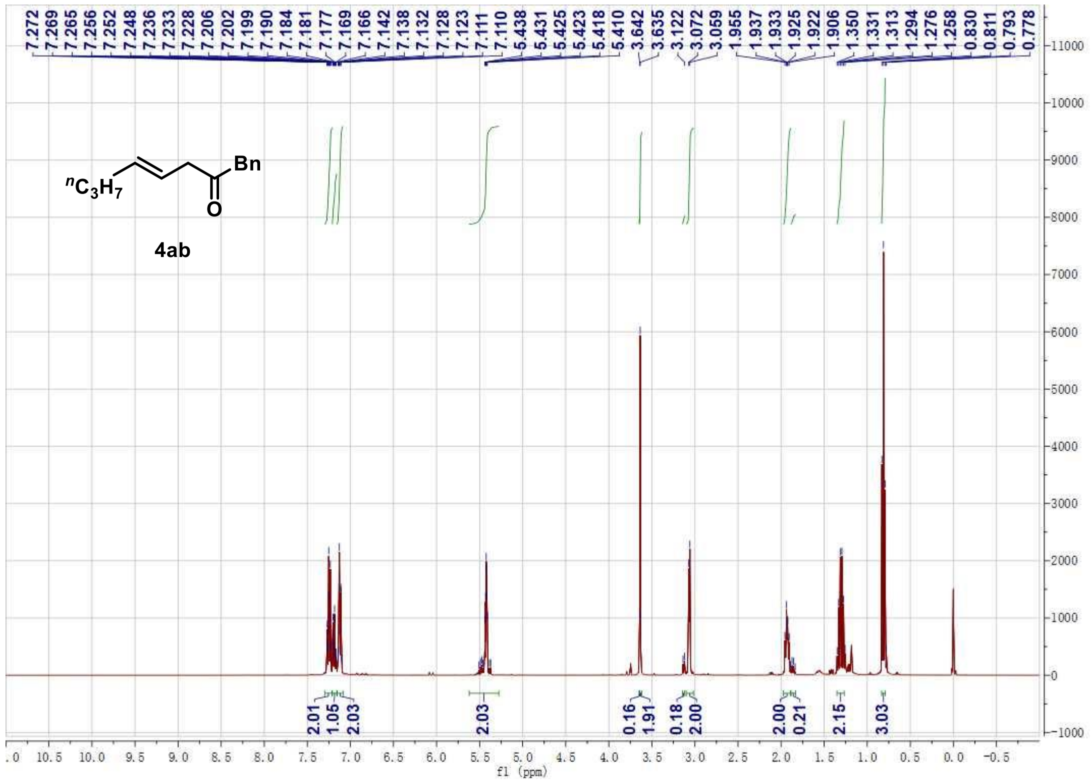

Supplementary Figure 81. ^{1}H NMR (400 MHz, $CDCl_3$) spectrum of 4ab

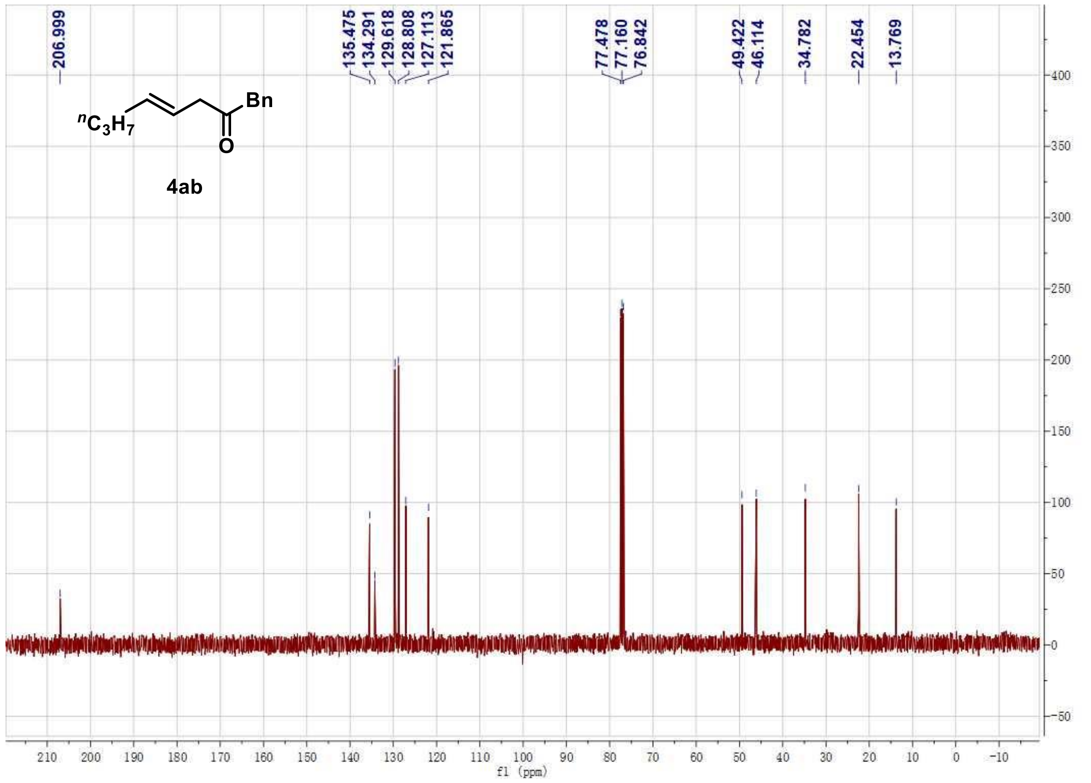

Supplementary Figure 82. ^{13}C NMR (100 MHz, $CDCl_3$) spectrum of 4ab

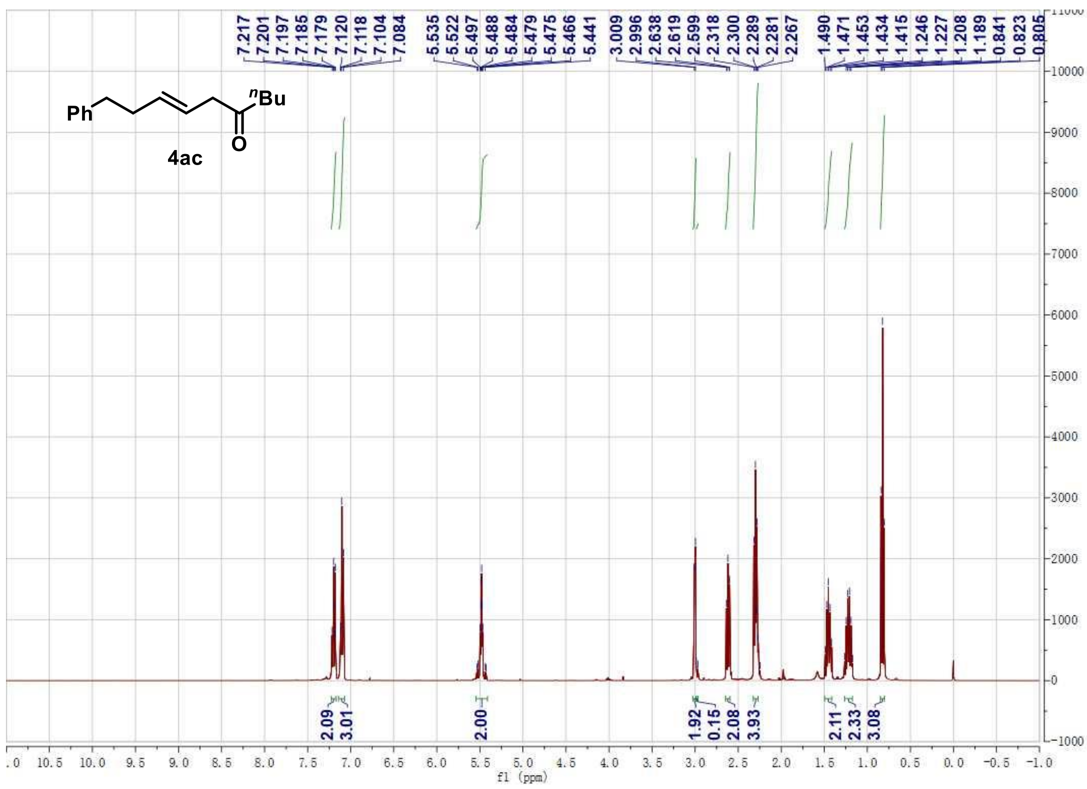

Supplementary Figure 83. ^{1}H NMR (400 MHz, $CDCl_3$) spectrum of 4ac

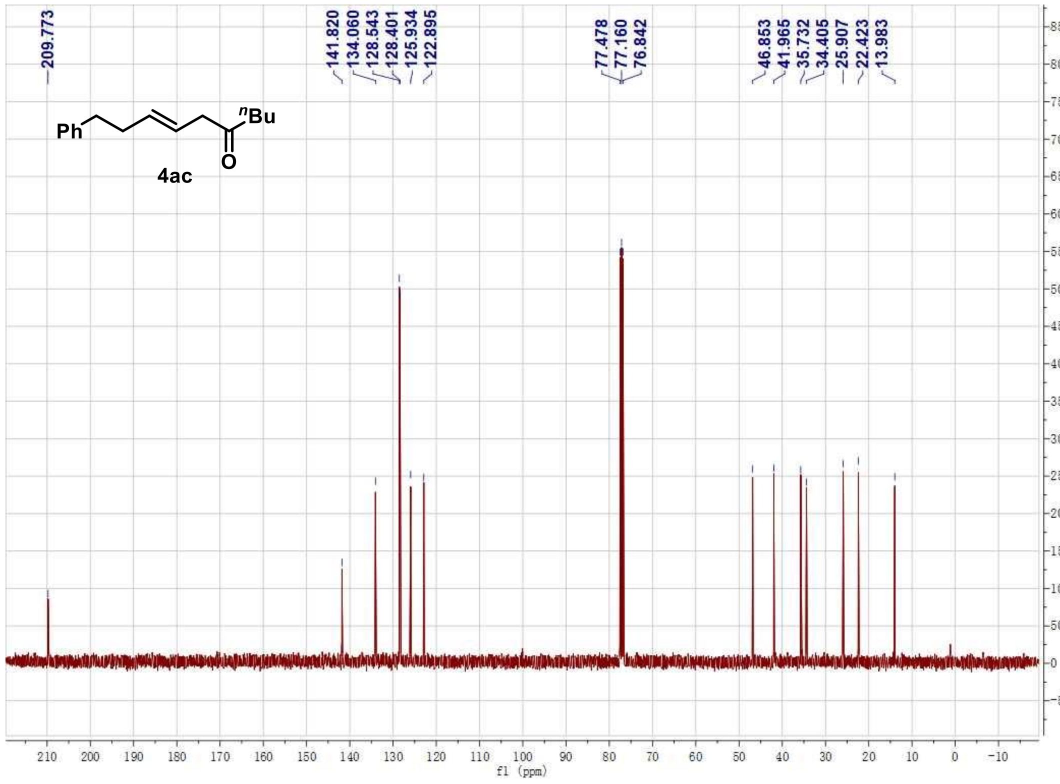

Supplementary Figure 84. ^{13}C NMR (100 MHz, $CDCl_3$) spectrum of 4ac

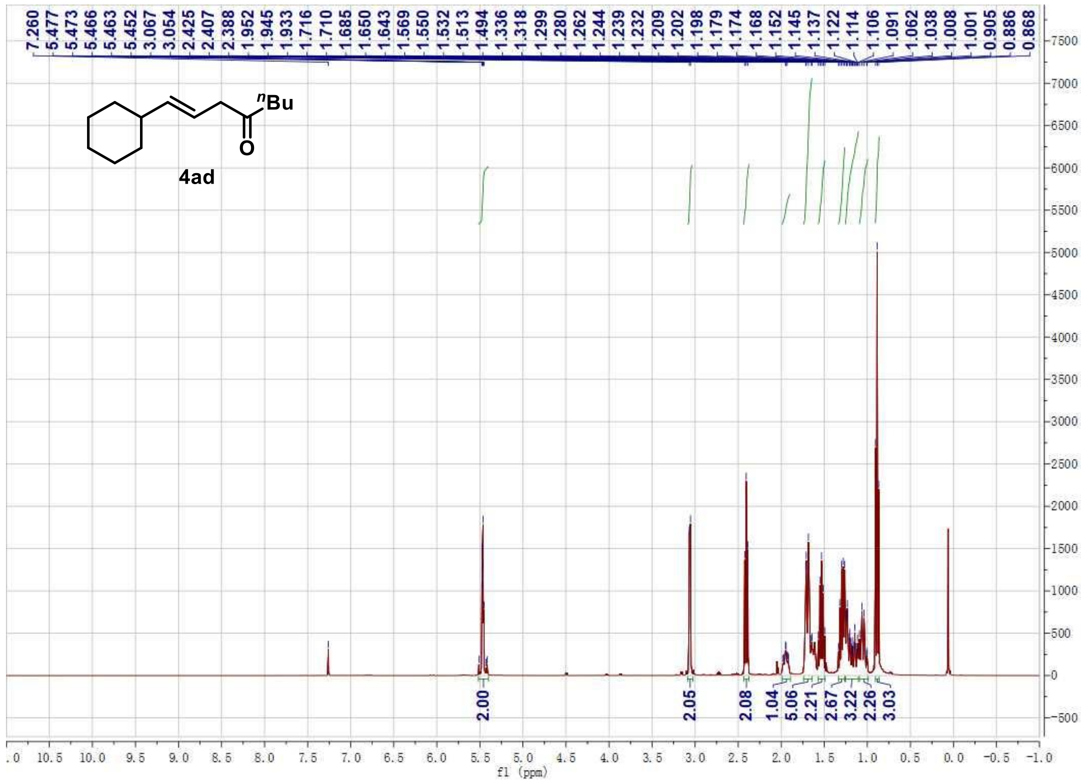

Supplementary Figure 85. ^{1}H NMR (400 MHz, $CDCl_3$) spectrum of 4ad

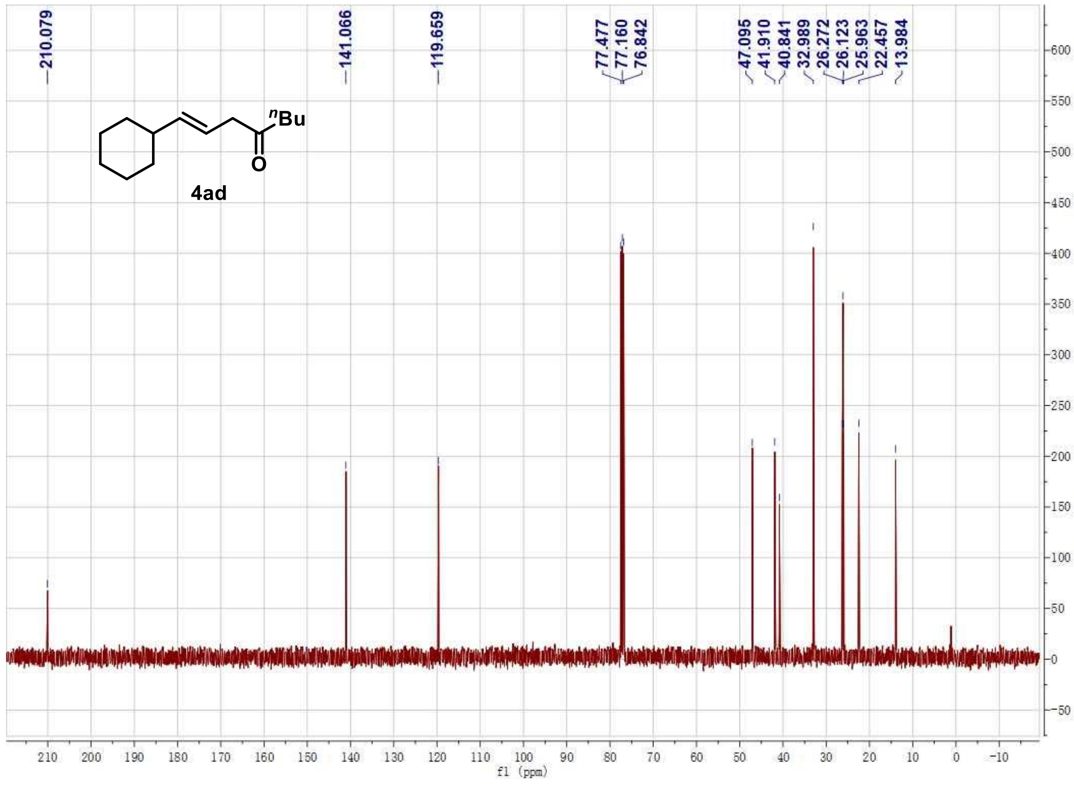

Supplementary Figure 86. ^{13}C NMR (100 MHz, $CDCl_3$) spectrum of 4ad

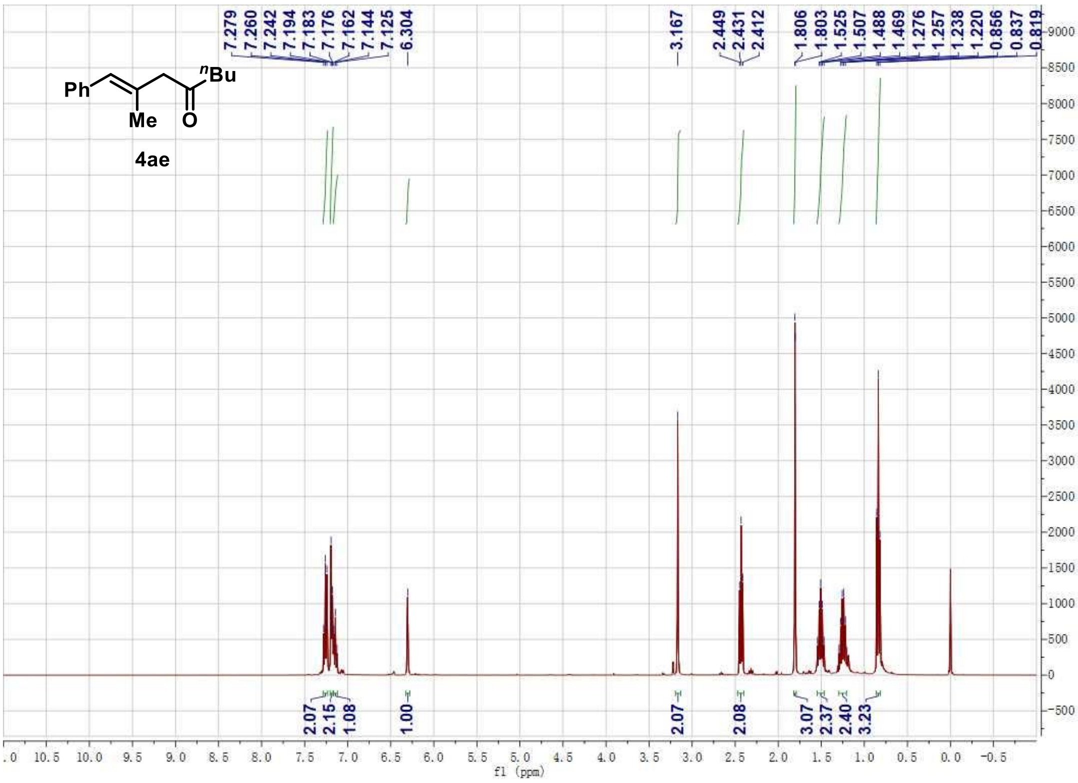

Supplementary Figure 87. ^{1}H NMR (400 MHz, $CDCl_3$) spectrum of 4ae

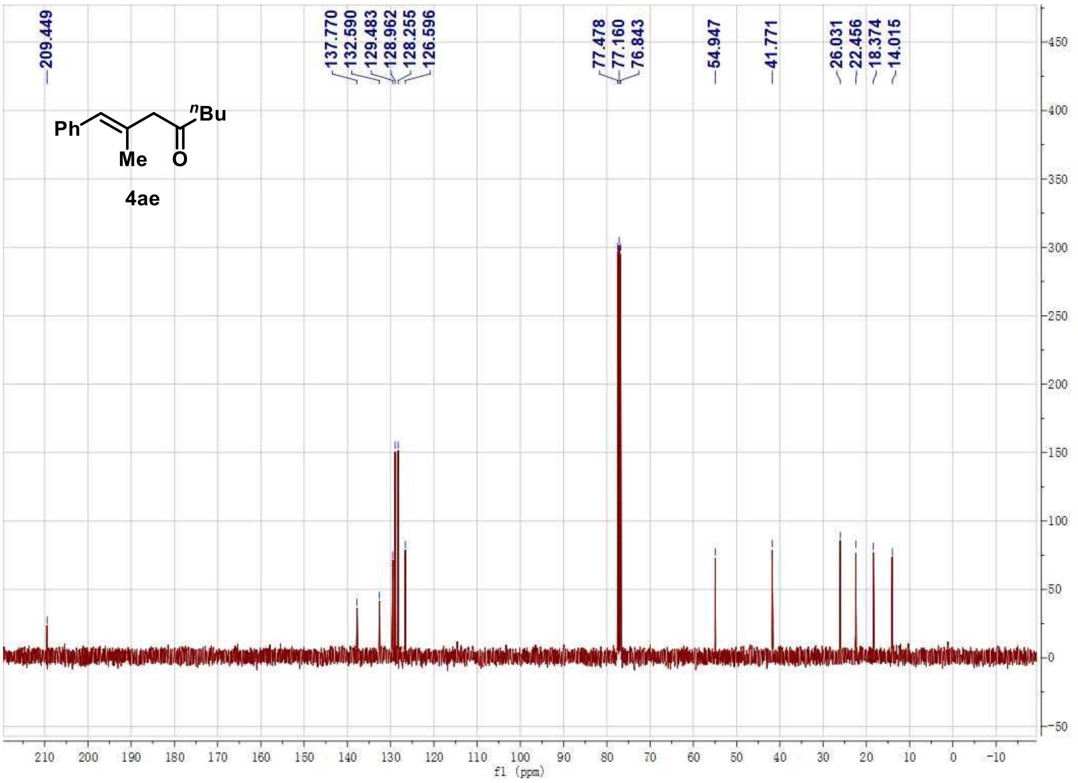

Supplementary Figure 88. ^{13}C NMR (100 MHz, $CDCl_3$) spectrum of 4ae

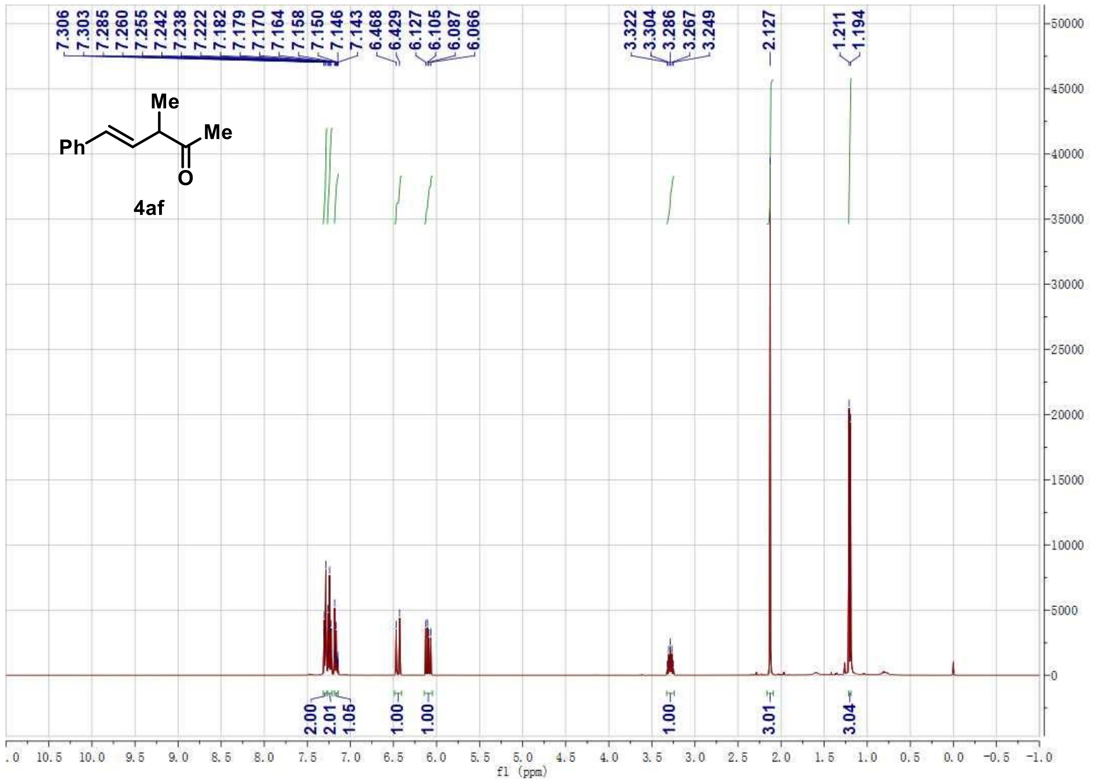

Supplementary Figure 89. **^{1}H NMR (400 MHz, $CDCl_3$) spectrum of 4af**

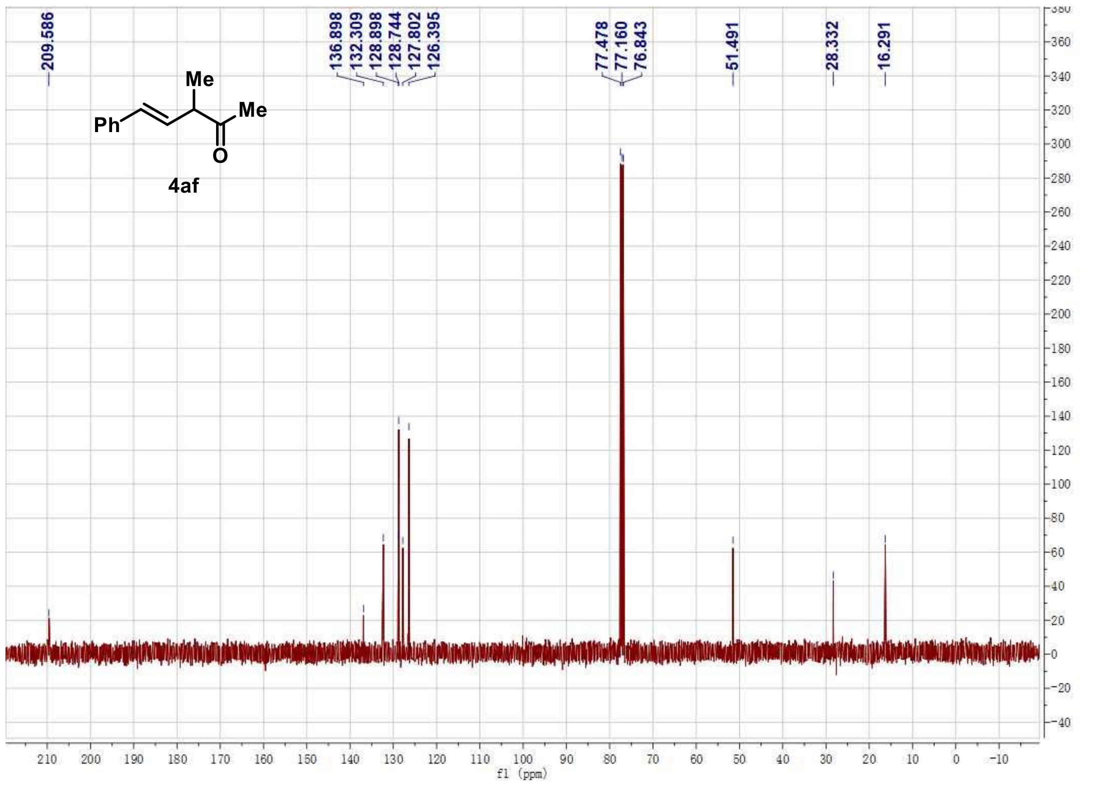

Supplementary Figure 90. **^{13}C NMR (100 MHz, $CDCl_3$) spectrum of 4af**

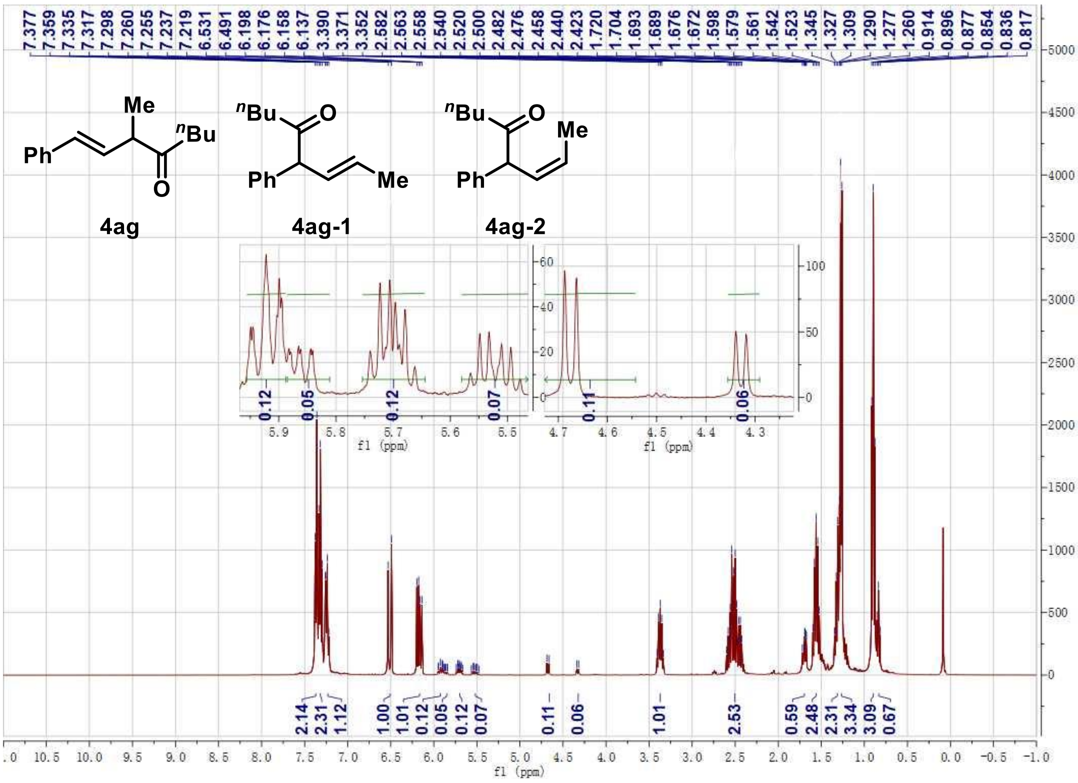

Supplementary Figure 91. ^{1}H NMR (400 MHz, $CDCl_3$) spectrum of 4ag and isomers

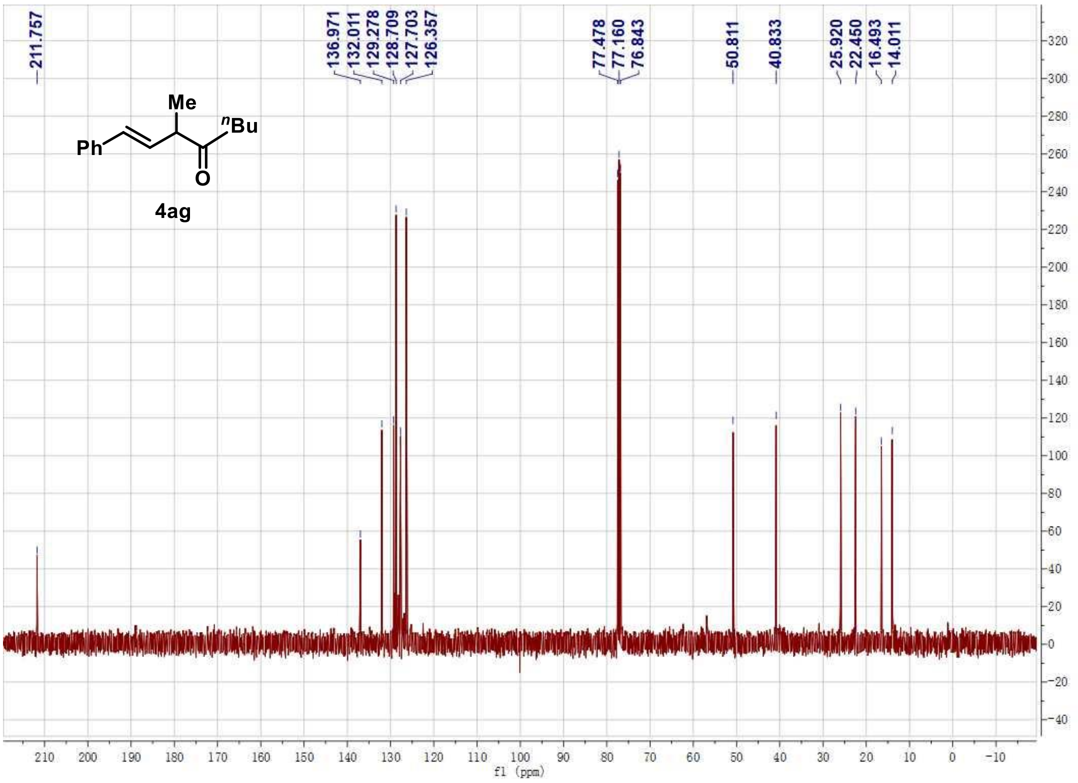

Supplementary Figure 92. ^{13}C NMR (100 MHz, $CDCl_3$) spectrum 4ag

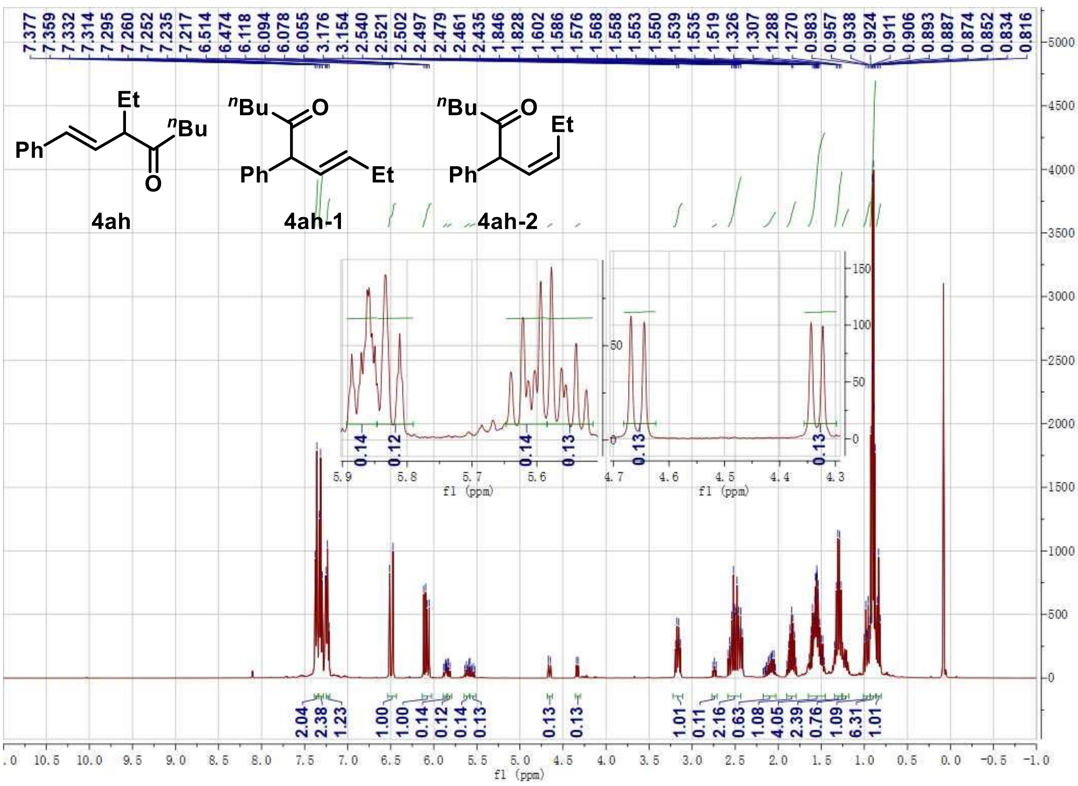

Supplementary Figure 93. ^{1}**H NMR (400 MHz, CDCl$_3$) spectrum of 4ah and isomers**

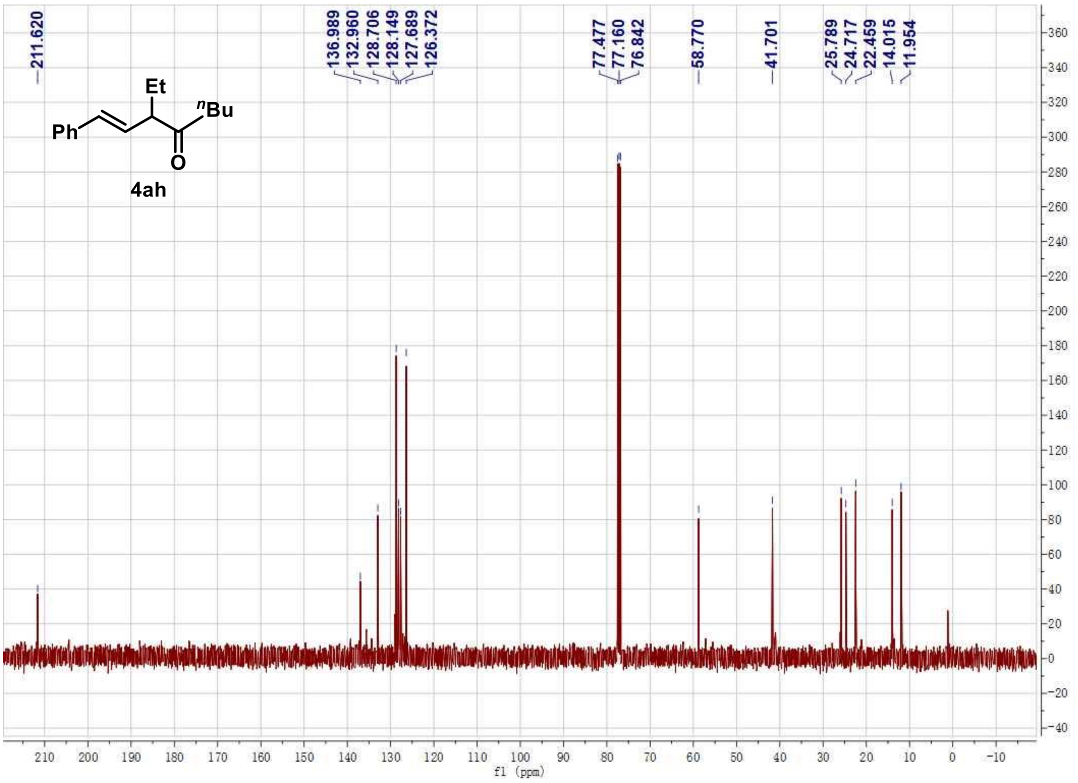

Supplementary Figure 94. ^{13}**C NMR (100 MHz, CDCl$_3$) spectrum 4ah**

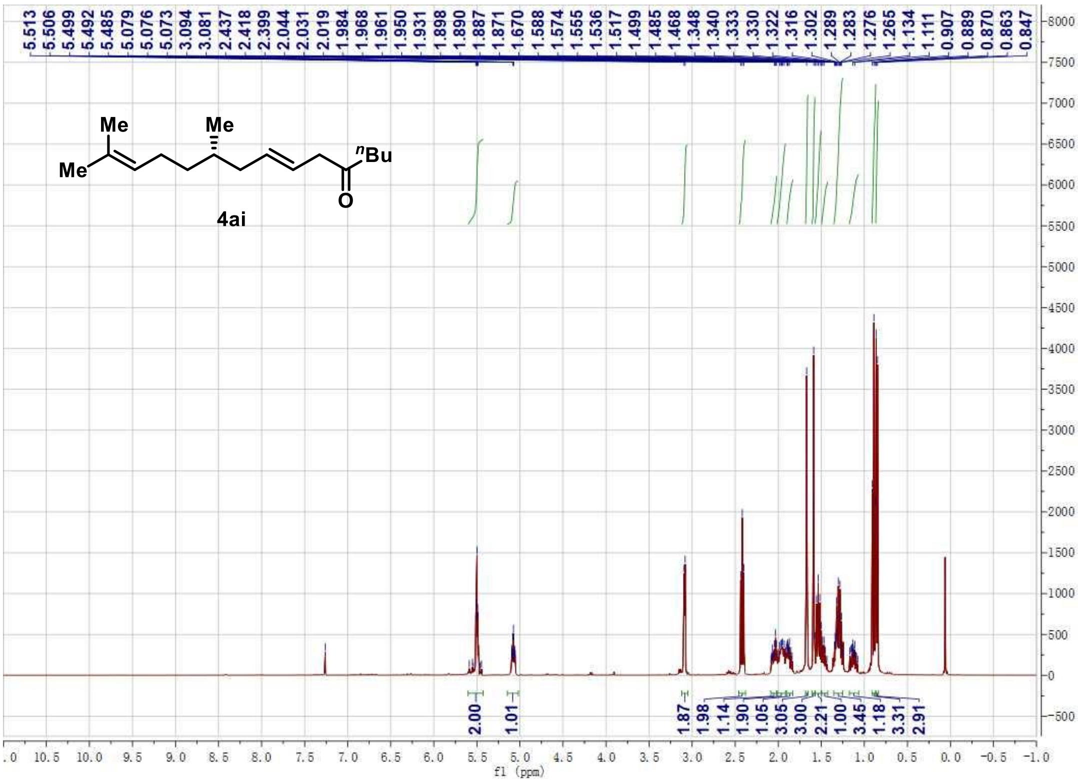

Supplementary Figure 95. ^{1}H NMR (400 MHz, $CDCl_3$) spectrum of 4ai

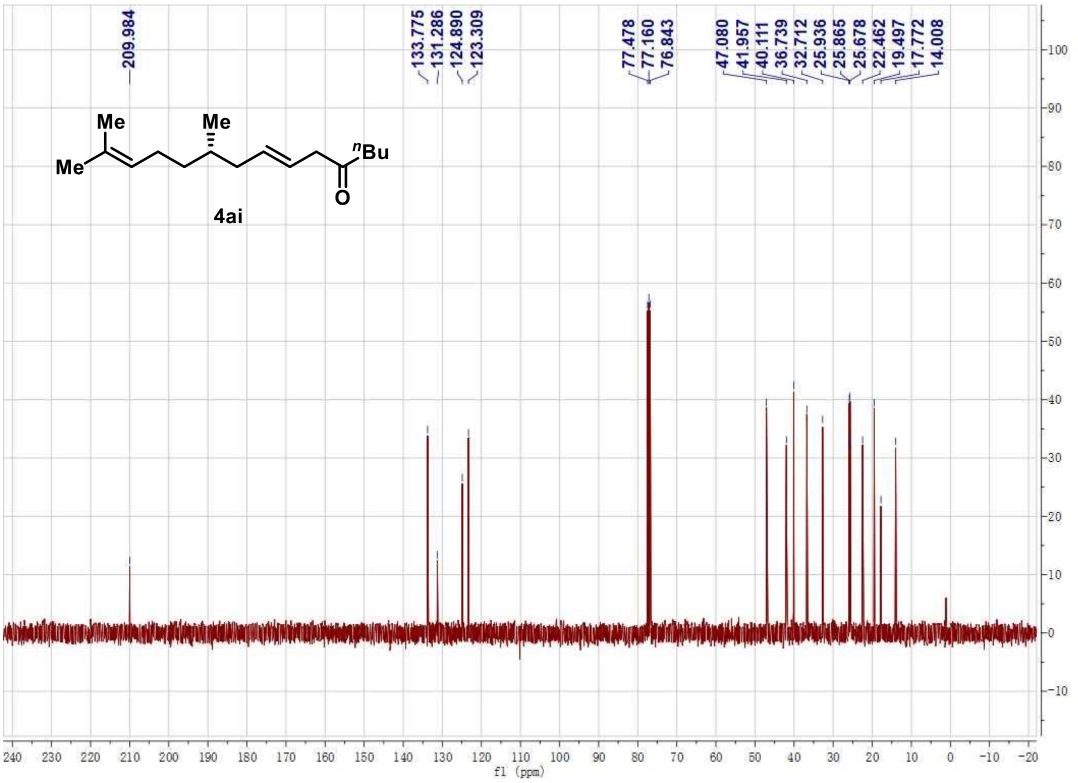

Supplementary Figure 96. ^{13}C NMR (100 MHz, $CDCl_3$) spectrum 4ai

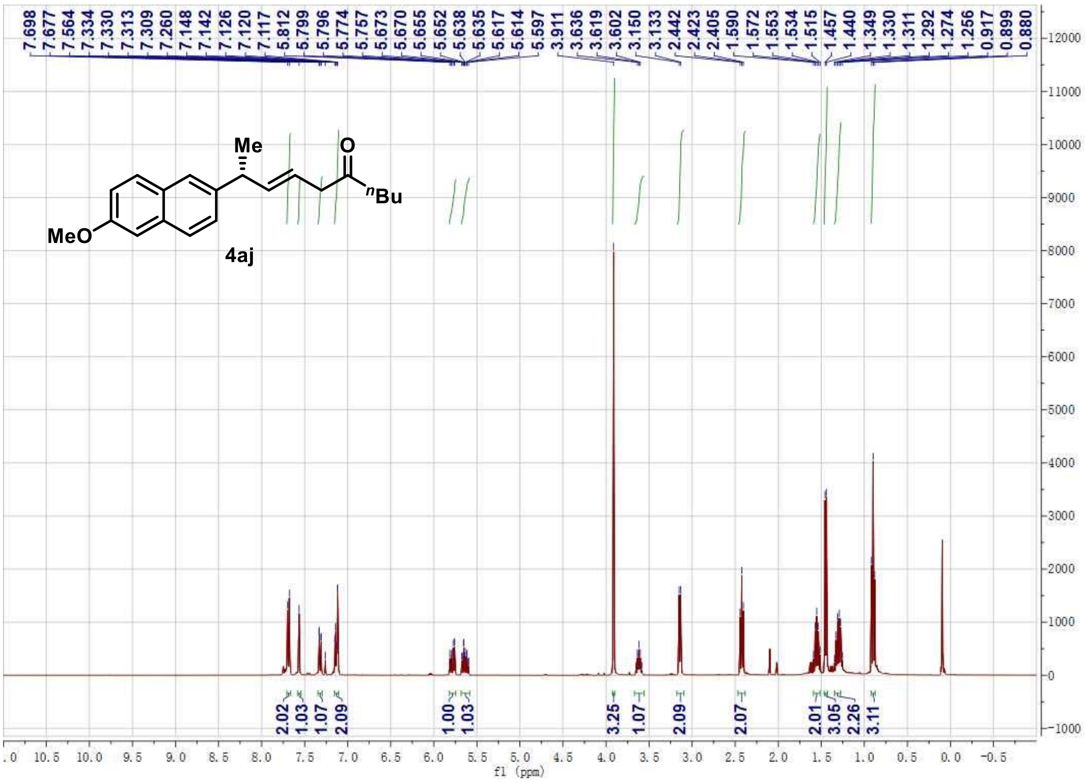

Supplementary Figure 97. ^{1}H NMR (400 MHz, $CDCl_3$) spectrum of 4aj

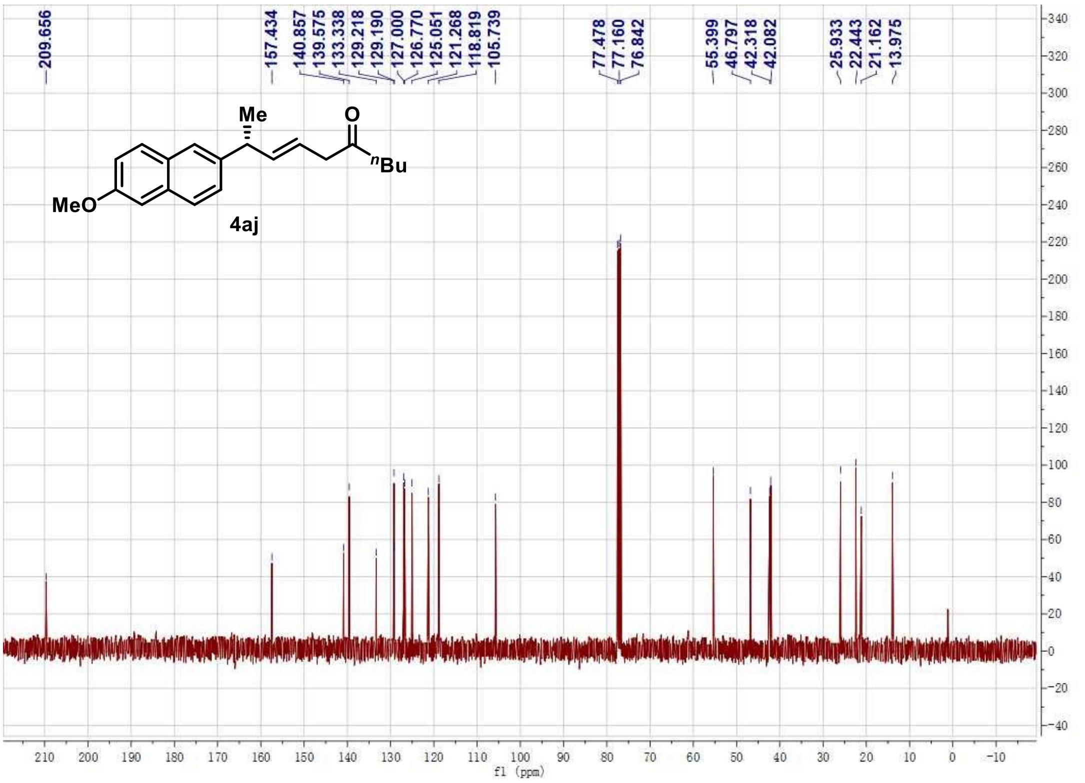

Supplementary Figure 98. ^{13}C NMR (100 MHz, $CDCl_3$) spectrum 4aj

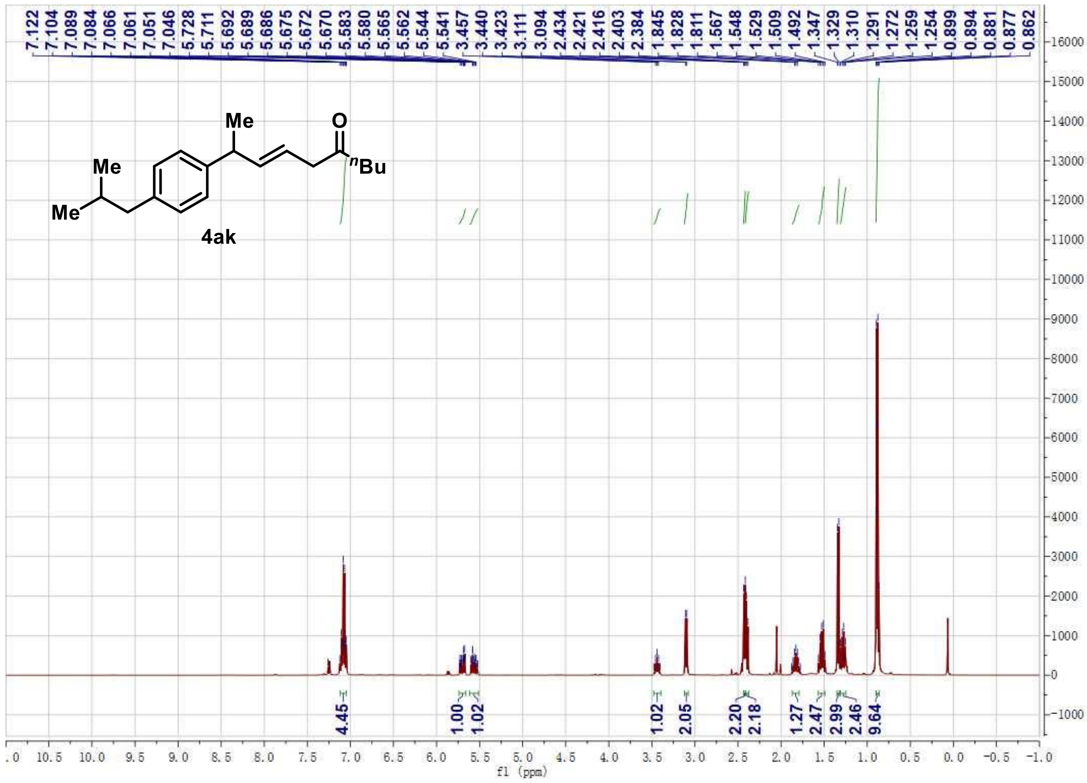

Supplementary Figure 99. ^{1}H NMR (400 MHz, $CDCl_3$) spectrum of 4ak

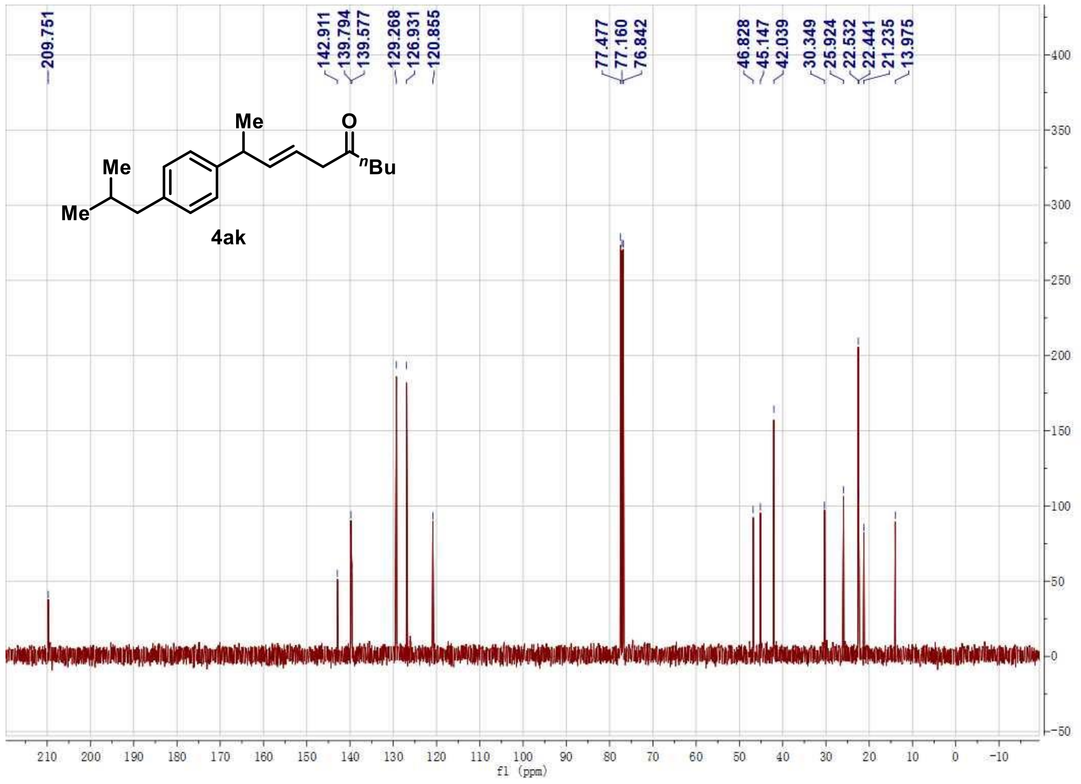

Supplementary Figure 100. ^{13}C NMR (100 MHz, $CDCl_3$) spectrum 4ak

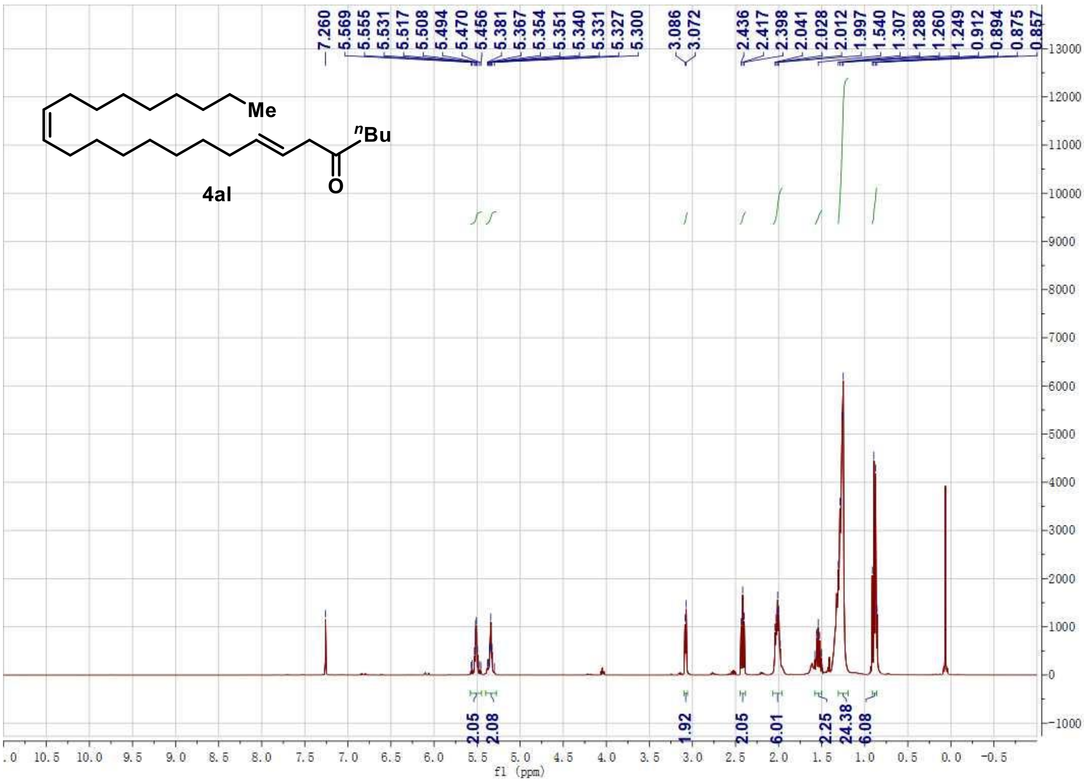

Supplementary Figure 101. ^{1}H **NMR (400 MHz, $CDCl_3$) spectrum of 4al**

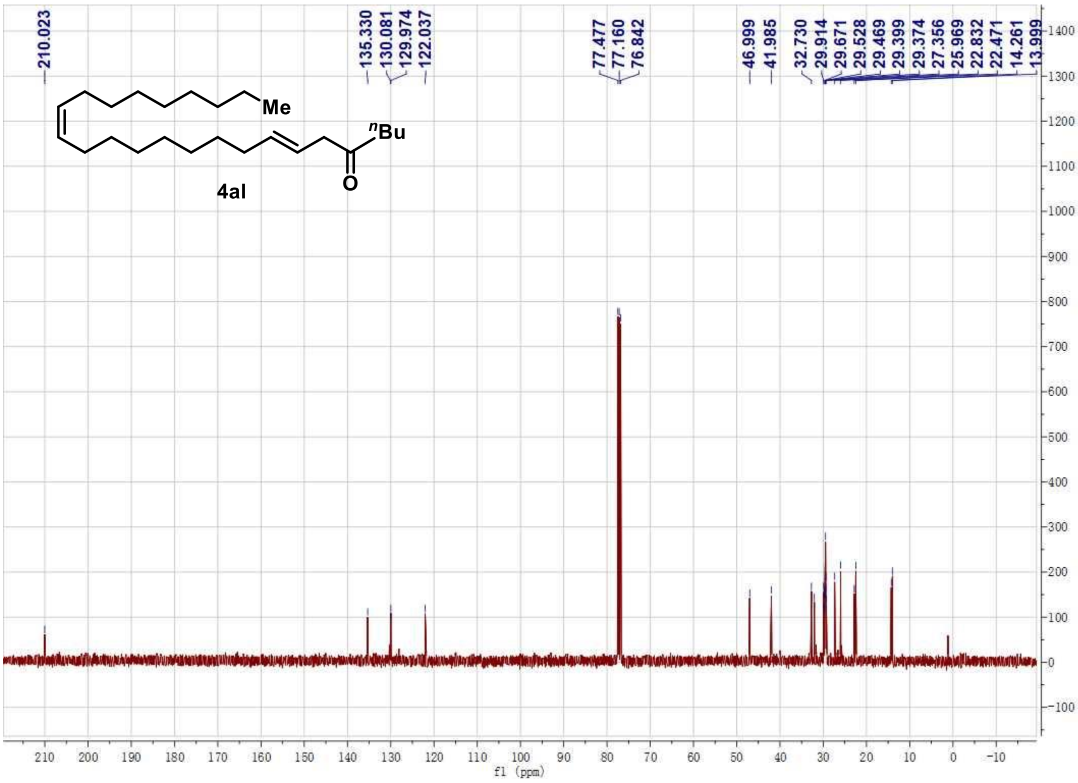

Supplementary Figure 102. ^{13}C **NMR (100 MHz, $CDCl_3$) spectrum 4al**

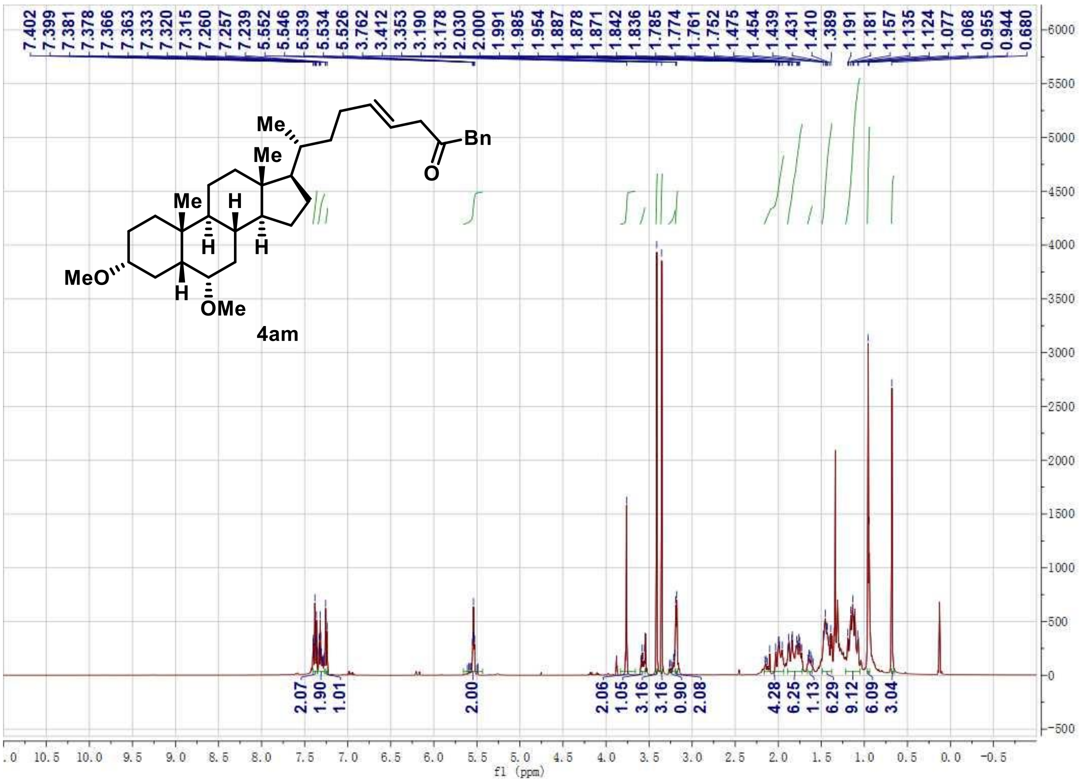

Supplementary Figure 103. ^{1}H NMR (400 MHz, $CDCl_3$) spectrum of 4am

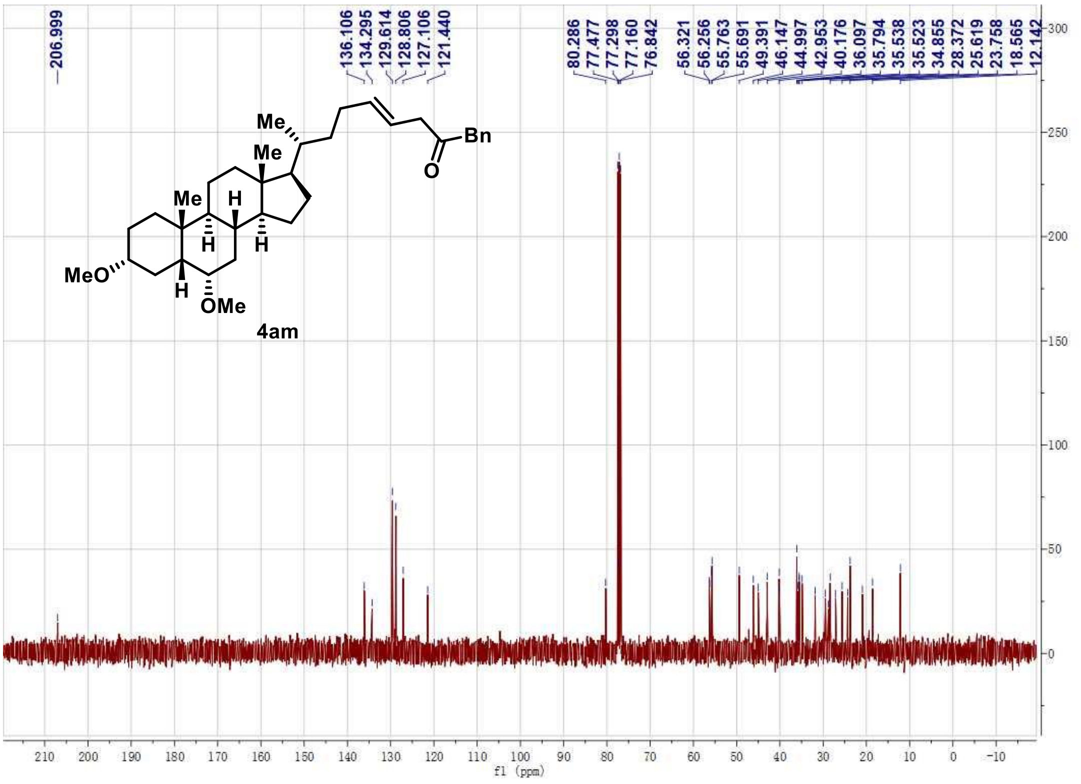

Supplementary Figure 104. ^{13}C NMR (100 MHz, $CDCl_3$) spectrum 4am

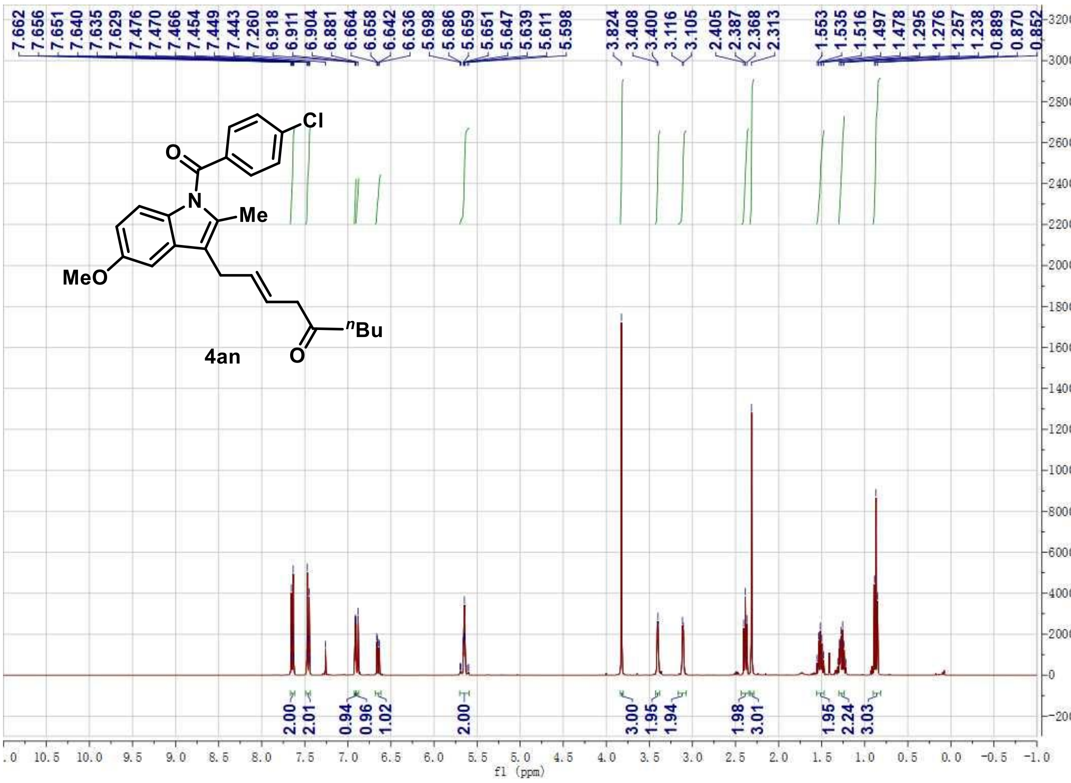

Supplementary Figure 105. ^{1}H NMR (400 MHz, $CDCl_3$) spectrum of 4an

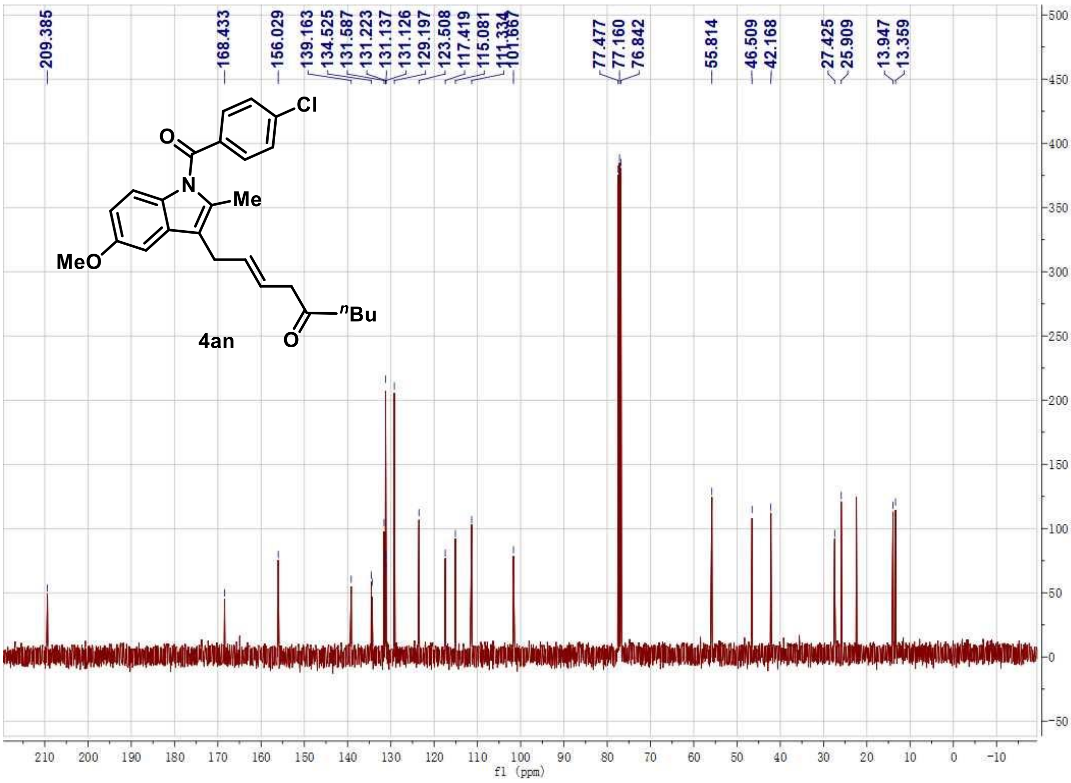

Supplementary Figure 106. ^{13}C NMR (100 MHz, $CDCl_3$) spectrum 4an

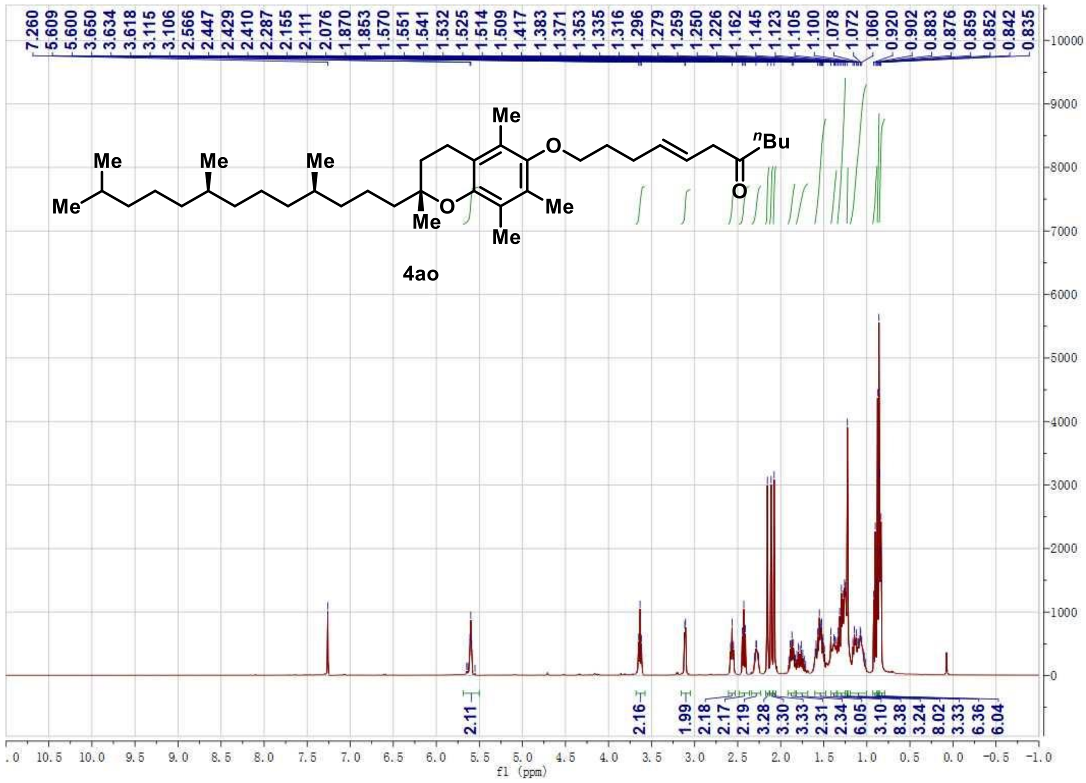

Supplementary Figure 107. ^{1}**H NMR (400 MHz, CDCl**$_3$**) spectrum of 4ao**

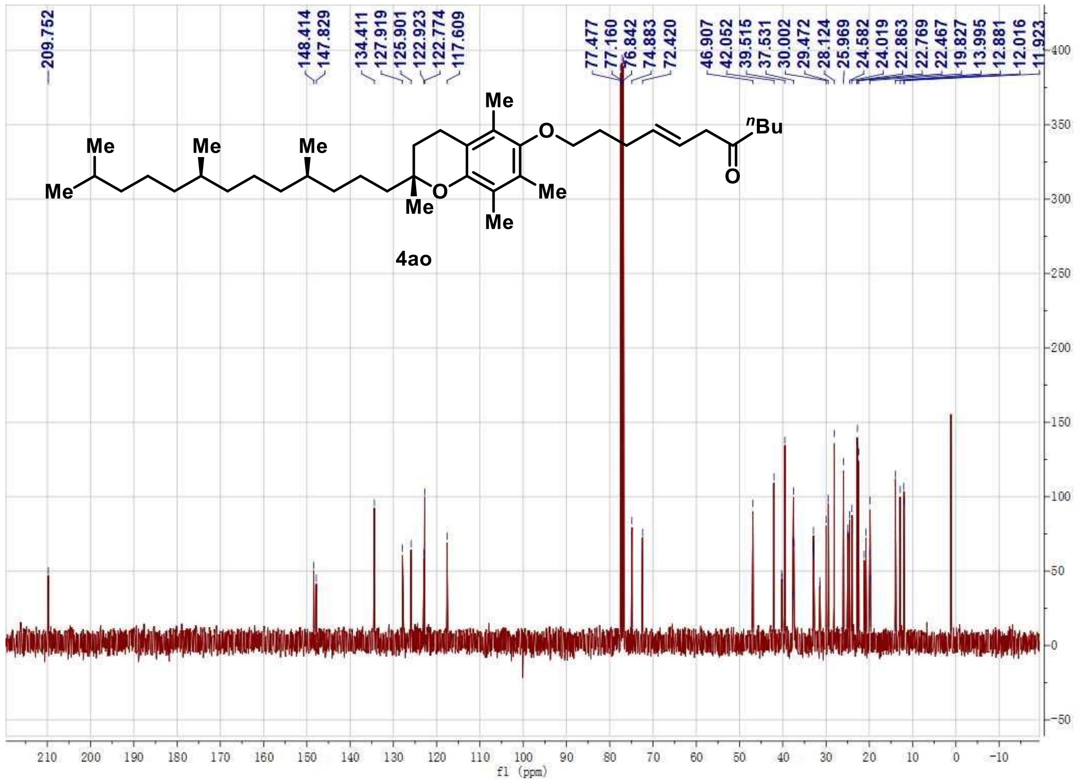

Supplementary Figure 108. ^{13}**C NMR (100 MHz, CDCl**$_3$**) spectrum 4ao**

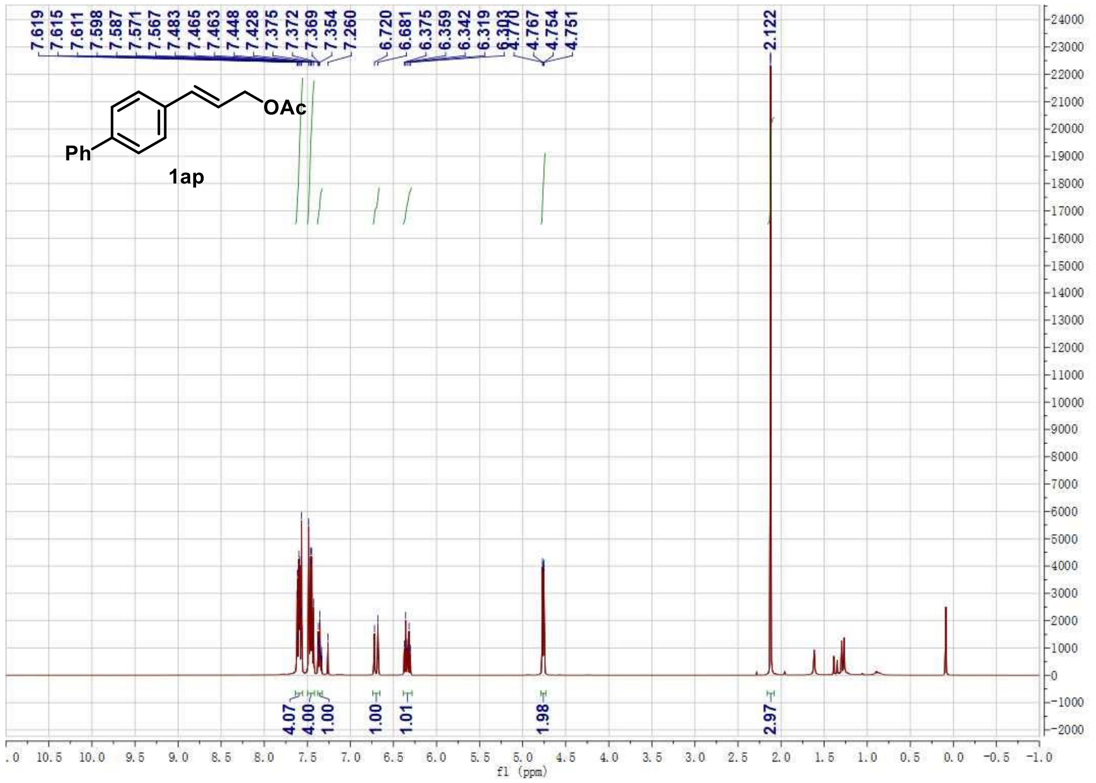

Supplementary Figure 109. ^{1}H NMR (400 MHz, CDCl$_3$) spectrum of 1ap

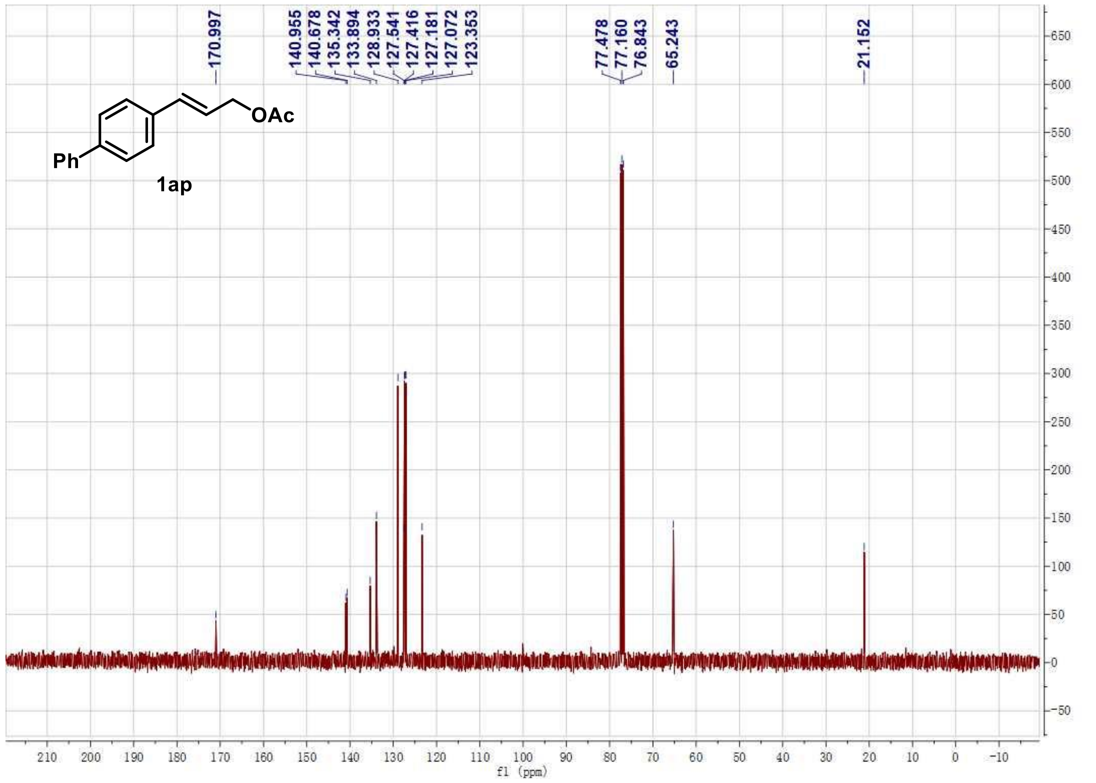

Supplementary Figure 110. ^{13}C NMR (100 MHz, CDCl$_3$) spectrum 1ap

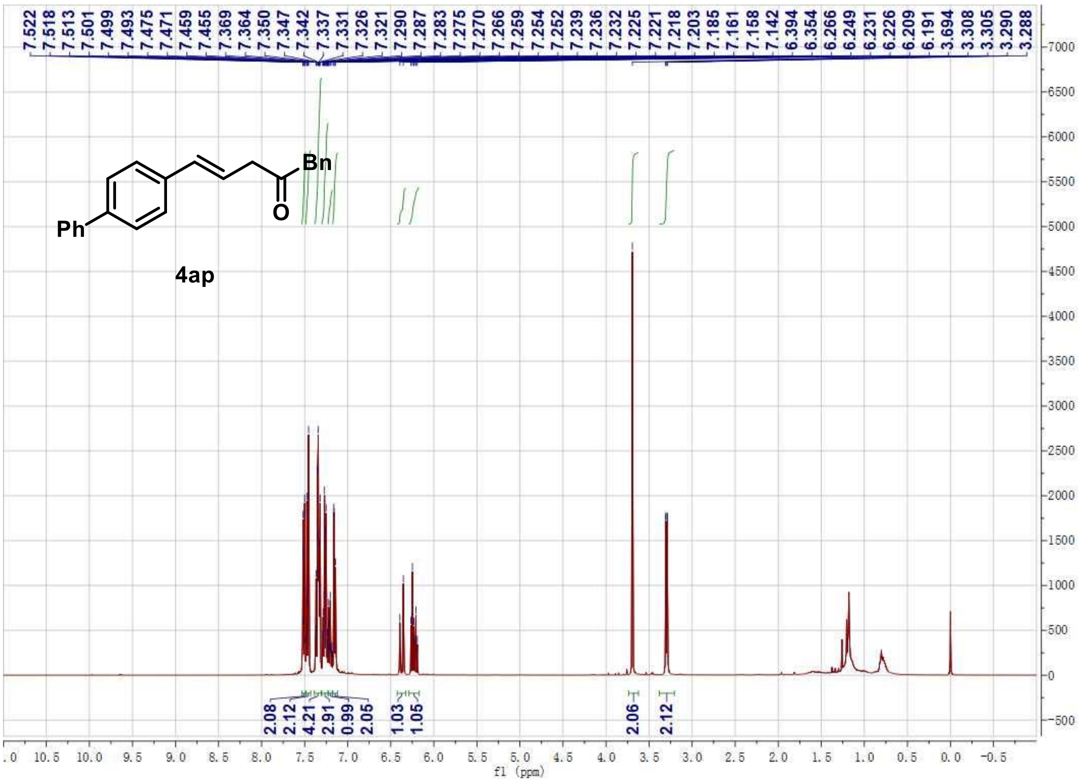

Supplementary Figure 111. ^{1}H NMR (400 MHz, CDCl$_3$) spectrum of 4ap

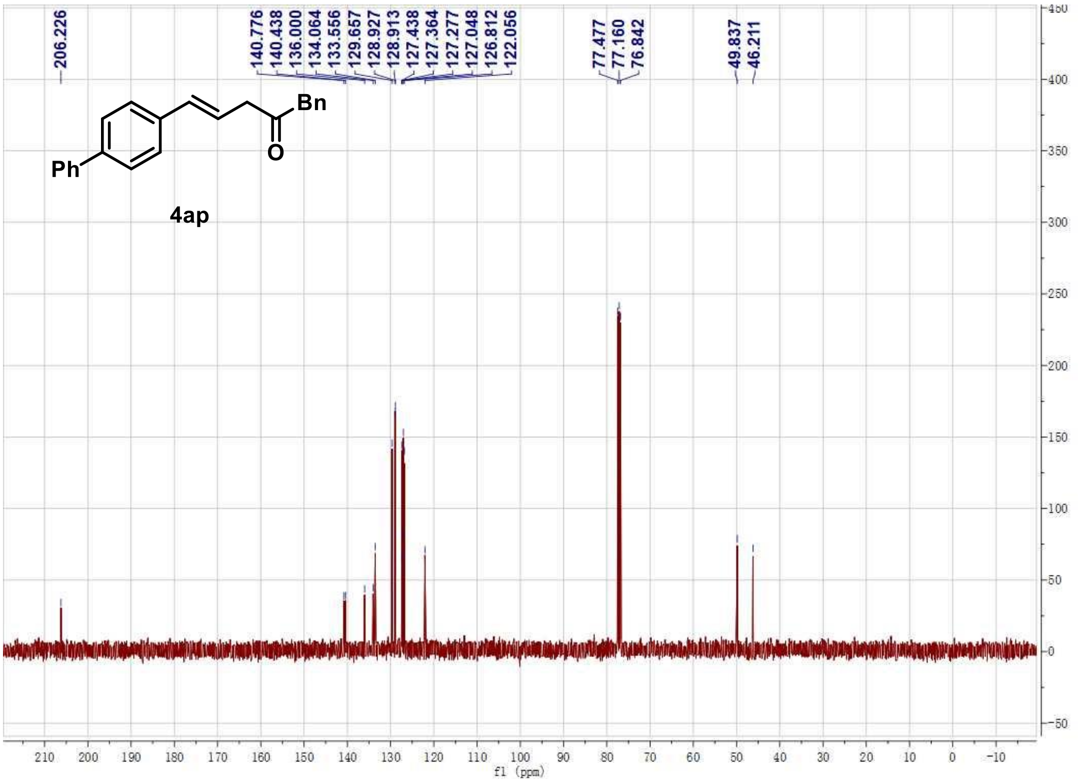

Supplementary Figure 112. ^{13}C NMR (100 MHz, CDCl$_3$) spectrum 4ap

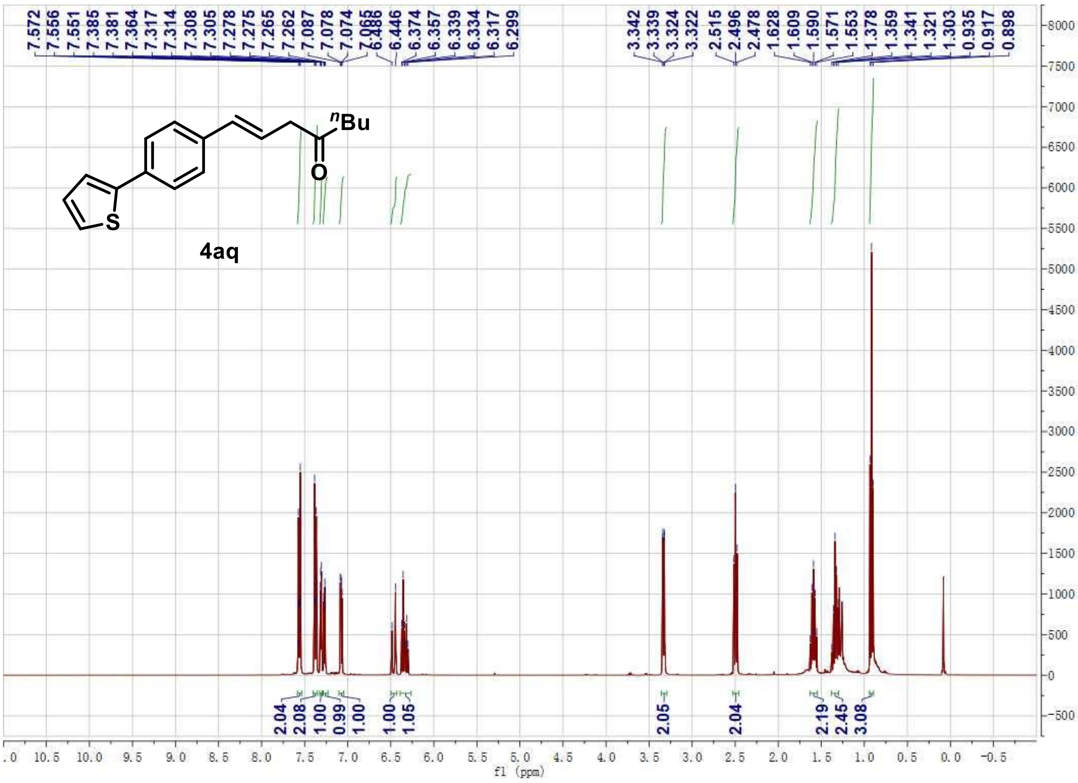

Supplementary Figure 113. ^{1}**H NMR (400 MHz, CDCl**$_3$**) spectrum of 4aq**

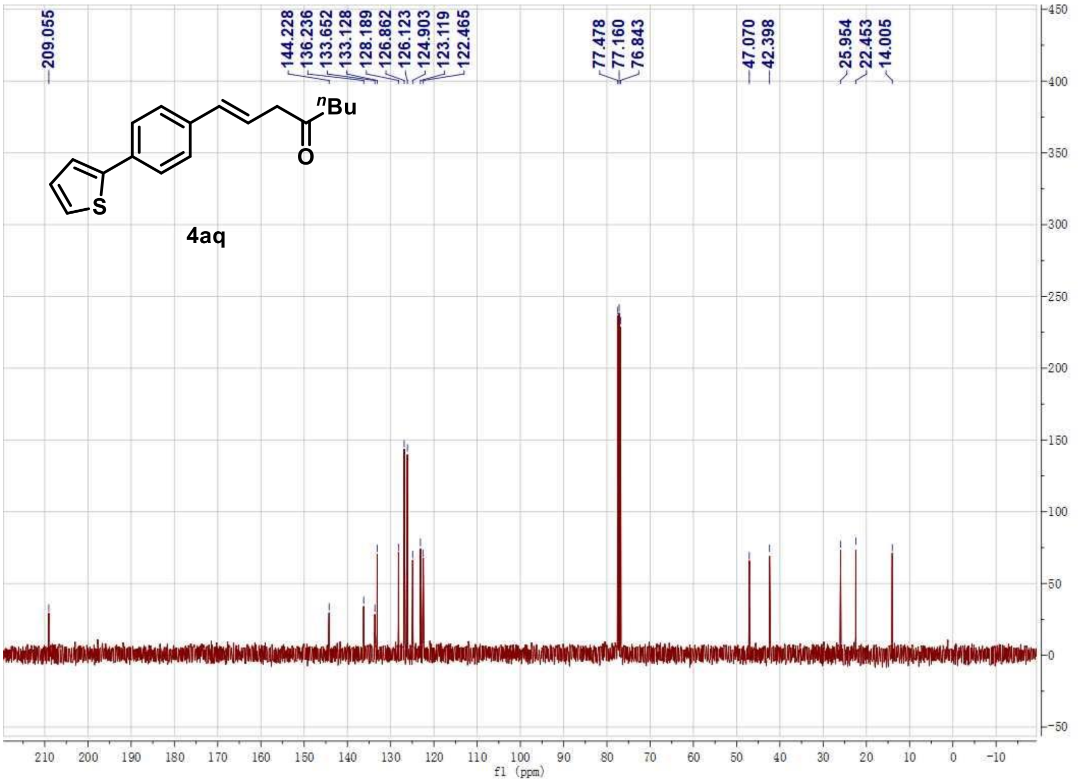

Supplementary Figure 114. ^{13}**C NMR (100 MHz, CDCl**$_3$**) spectrum 4aq**

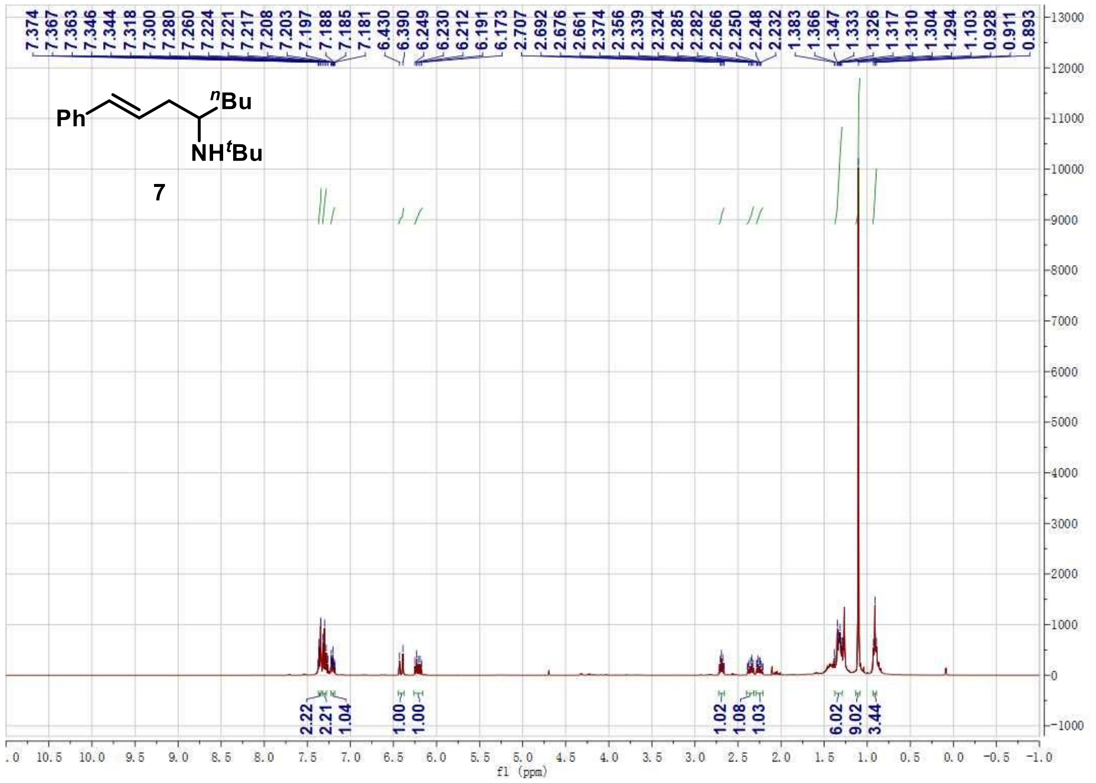

Supplementary Figure 115. **^{1}H NMR (400 MHz, CDCl$_3$) spectrum of 7**

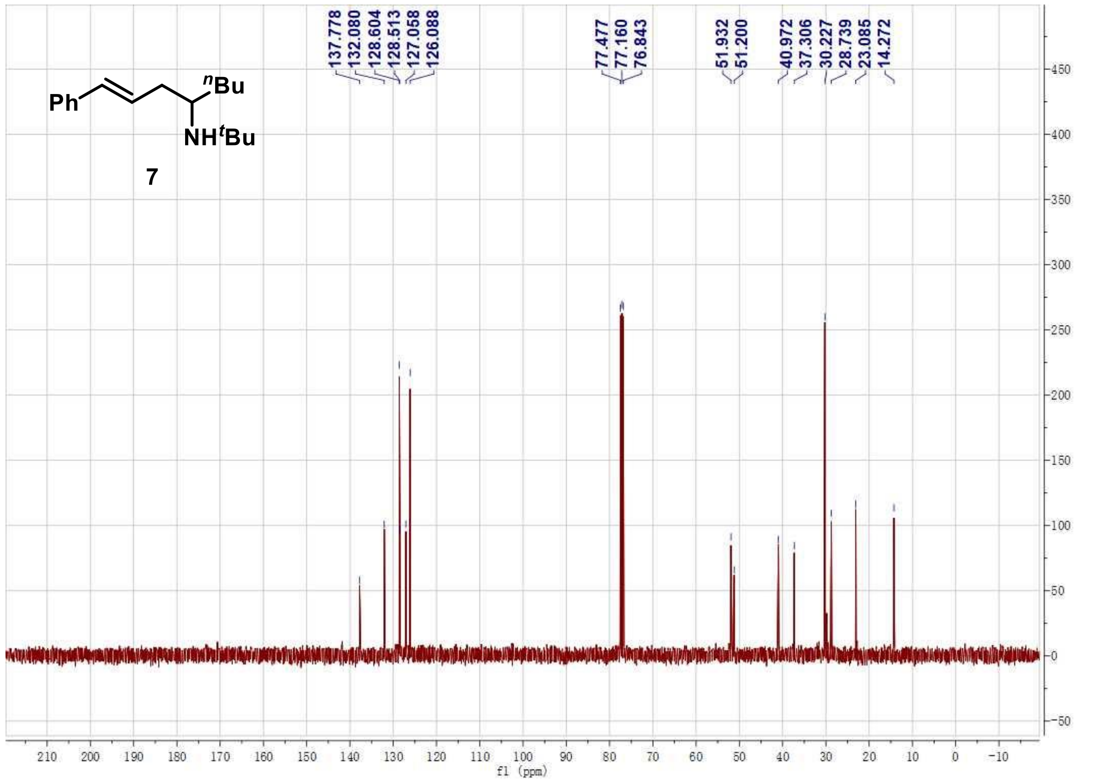

Supplementary Figure 116. **^{13}C NMR (100 MHz, CDCl$_3$) spectrum 7**

Supplementary References

[1] Krasovskiy, A., Knochel, P. Convenient Titration Method for Organometallic Zinc, Magnesium, and Lanthanide- Reagents. *Synthesis* **5**, 890–891 (2006).

[2] Krätzschmar, F., Kaßel, M., Delony, D. & Breder, A. Selenium-Catalyzed C(sp^3)–H Acyloxylation: Application in the Expedient Synthesis of Isobenzofuranones. *Chem. Eur. J.* **21**, 7030–7034 (2015).

[3] Song, T., Arseniyadis, S. & Cossy, J. Highly Enantioselective, Base-Free Synthesis of α-Quaternary Succinimides through Catalytic Asymmetric Allylic Alkylation. *Chem. Eur. J.* **24**, 8076–8080 (2018).

[4] Iwasaki, M., Kobayashi, Y., Li, J. P., Matsuzaka, H., Ishii, Y. & Hidai, M. Construction of polycyclic compounds by cyclocarbonylation. 6. Palladium-catalyzed cyclocarbonylation of 3-(heteroaryl)allyl acetates. *J. Org. Chem.* **56**, 1922–1927 (1991).

[5] Ueno, S., Hartwig, J. F. Direct, Iridium-Catalyzed Enantioselective and Regioselective Allylic Etherification with Aliphatic Alcohols. *Angew. Chem. Int. Ed.* **47**, 1928–1931 (2008).

[6] Bartels, B., García-Yebra, C., Rominger, F. & Helmchen, G. Iridium-Catalysed Allylic Substitution: Stereochemical Aspects and Isolation of Ir^{III} Complexes Related to the Catalytic Cycle. *Eur. J. Inorg. Chem.* **2002**, 2569–2586 (2002).

[7] Ding, F., William, R., Wang, F. & Liu, X.-W. Triflimide-catalyzed allyl–allyl cross-coupling: a metal-free allylic alkylation. *Chem. Commun.* **48**, 8709–8711 (2012).

[8] Le, H., Batten, A. & Morken, J. P. Catalytic Stereospecific Allyl–Allyl Cross-Coupling of Internal Allyl Electrophiles with AllylB(pin). *Org. Lett.* **16**, 2096–2099 (2014).

[9] Xia, C., Shen, J., Liu, D. & Zhang, W. Synthesis of Chiral α,β-Unsaturated γ-Amino Esters via Pd-Catalyzed Asymmetric Allylic Amination. *Org. Lett.* **19**, 4251–4254 (2017).

[10] Miura, M., Koike, T., Ishihara, T., Sakamoto, S., Okada, M., Ohta, M. & Tsukamoto, S.-i. One-Pot Preparation of Unsymmetrical Biaryls via Suzuki Cross-Coupling Reaction of Aryl Halide using Phase-Transfer Catalyst in a Biphasic Solvent System. *Synth. Commun.* **37**, 667–674 (2007).

[11] Richmond, E., Ling, K. B., Duguet, N., Manton, L. B., Çelebi-Ölçüm, N., Lam, Y.-H., Alsancak, S., Slawin, A. M. Z., Houk, K. N. & Smith, A. D. An asymmetric pericyclic cascade approach to 3-alkyl-3-aryloxindoles: generality, applications and mechanistic investigations. *Org. Biomol. Chem.* **13**, 1807–1817 (2015).

[12] Tassano, E., Faber, K. & Hall, M. Biocatalytic Parallel Interconnected Dynamic Asymmetric Disproportionation of α-Substituted Aldehydes: Atom-Efficient Access to Enantiopure (*S*)-Profens and Profenols. *Adv. Synth. Catal.* **360**, 2742–2751 (2018).

[13] Twidle, A. M., Suckling, D. M., Seal, A. G., Fedrizzi, B., Pilkington, L. I. & Barker, D. Identification of in situ flower volatiles from kiwifruit (Actinidia chinensis var. deliciosa) cultivars and their male pollenisers in a New Zealand orchard.

Phytochemistry **141**, 61–69 (2017).

[14] Tatamidani, H., Kakiuchi, F. & Chatani, N. A New Ketone Synthesis by Palladium-Catalyzed Cross-Coupling Reactions of Esters with Organoboron Compounds. *Org. Lett.* **6**, 3597–3599 (2004).

[15] Obora, Y., Ogawa, Y., Imai, Y., Kawamura, T. & Tsuji, Y. Palladium Complex Catalyzed Acylation of Allylic Esters with Acylsilanes. *J. Am. Chem. Soc.* **123**, 10489–10493 (2001).

[16] Izquierdo, J., Rodríguez, S. & González, F. V. Regioselective Ring Opening and Isomerization Reactions of 3,4-Epoxyesters Catalyzed by Boron Trifluoride. *Org. Lett.* **13**, 3856–3859 (2011).

[17] Akula, P. S., Hong, B.-C. & Lee, G.-H. Catalyst- and Substituent-Controlled Switching of Chemoselectivity for the Enantioselective Synthesis of Fully Substituted Cyclobutane Derivatives via 2 + 2 Annulation of Vinylogous Ketone Enolates and Nitroalkene. *Org. Lett.* **20**, 7835–7839 (2018).

[18] Chang, S., Yoon, J. & Brookhart, M. Carbon-Carbon Bond Forming Reactions of .eta.3-Allyl Iron Tricarbonyl Anions with Carbon Electrophiles. *J. Am. Chem. Soc.* **116**, 1869–1879 (1994).

[19] Zhuo, L.-G., Yao, Z.-K. & Yu, Z.-X. Synthesis of Z-Alkenes from Rh(I)-Catalyzed Olefin Isomerization of β,γ-Unsaturated Ketones. *Org. Lett.* **15**, 4634–4637 (2013).

[20] Yasui, K., Fugami, K., Tanaka, S. & Tamaru, Y. Unsymmetrical Ketone Synthesis via a Three-Component Connection Reaction of Organozincs, Allylating Agents, and Carbon Monoxide. *J. Org. Chem.* **60**, 1365–1380 (1995).

[21] Wakeham, R. J., Baillie, R. A., Patrick, B. O., Legzdins, P. & Rosenfeld, D. C. Selective Functionalization of a Variety of Hydrocarbon C(sp^3)–H Bonds Initiated by Cp*W(NO)(CH_2CMe_3)(η^3-$CH_2CHCHPh$) *Organometallics* **36,** 39–52 (2017).

[22] Chapado, L., Linares-Palomino, P. J., Badía, C., Salido, S., Nogueras, M., Sánchez, A. & Altarejos, J. Synthesis and Olfactory Evaluation of Bulky Moiety-Modified Analogues to the Sandalwood Odorant Polysantol. *Molecules* **14**, 2780–2800 (2009).

[23] Fronza, G., Fuganti, C., Högberg, H.-E., Pedrocchi-Fantoni, G. & Servi, S. Chiral α-Methyl-homoallylic Alcohols from Yeast-Generated Precursors. Synthesis of (4R,5S) Sitophilure. *Chem. Lett.* **17**, 385–388 (1988).

[24] Croom, A., Tarallo, T. & Weck, M. End-group functionalization and postpolymerization modification of helical poly(isocyanide)s. *J. Polym. Sci., Part A: Polym. Chem.* **54**, 2766–2773 (2016).
